# Palladium-Catalyzed Oxidative Acyloxylation/Carbocyclization of Allenynes**

**DOI:** 10.1002/anie.201208718

**Published:** 2013-02-05

**Authors:** Youqian Deng, Jan-E Bäckvall

**Affiliations:** * Department of Organic Chemistry, Arrhenius Laboratory, Stockholm UniversitySE-106 91 Stockholm (Sweden) E-mail: jeb@organ.su.se

**Keywords:** allenynes, carboxylic acids, cyclization, oxidation, palladium

Palladium(II)-catalyzed oxidative carbocyclizations represent an important class of reactions, which have provided powerful and atom-economical approaches to carbo- and heterocycles.^1^–^5^ In particular, oxidative carbocyclization strategies have been efficiently applied to total synthesis.^3^ As a continuation of our research on the palladium-catalyzed oxidative carbocyclizations of dienallenes^4^ and enallenes,^5^ we recently developed palladium-catalyzed arylating or borylating oxidative carbocyclizations of allenynes^6^ by using the corresponding arylboronic acid or B_2_pin_2_.^7^

In connection with our previous studies on acetoxylation(hydroxylation)/carbocyclizations of dienallenes (Scheme 1 a),^4^ we envisioned an oxidative acetoxylation/carbocyclization of allenynes **1** in the presence of HOAc/LiOAc, with a simple Pd^II^ salt as the catalyst (Scheme 1 b). Based on the previously proposed mechanism,^4^, ^5^ the reaction was expected to be initiated by allene attack on Pd^II^ through allylic C–H bond cleavage followed by alkyne insertion to give acetoxylated triene product **2**. However, the reaction took an unexpected path, and herein we report on a palladium-catalyzed oxidative acyloxylation/carbocyclization of allenynes **1** to give acyloxylated vinylallenes **3** (Scheme 1 b). An aerobic version of this transformation was also realized by using catalytic amounts of *p*-benzoquinone together with cobalt salophen.

**Scheme 1:**
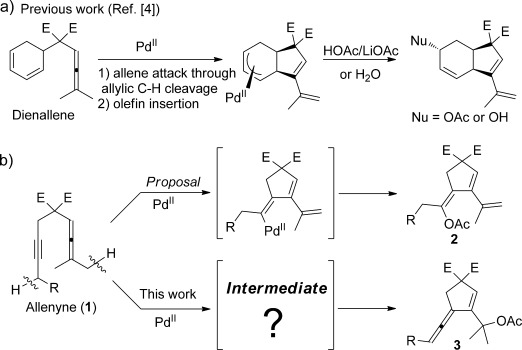
Palladium-catalyzed oxidative acetoxylation/carbocyclization of dienallenes and allenynes (E=CO_2_Me).

In our preliminary study, we chose allenyne **1 a** with a pentyl group on the triple bond as the model substrate to study the oxidative carbocyclization in the presence of HOAc/LiOAc. To our surprise, treatment of **1 a** with Pd(OAc)_2_ (5 mol %), LiOAc⋅2 H_2_O (2 equiv), and *p*-benzoquinone (BQ; 1.2 equiv) at 60 °C in HOAc gave an acetoxylated vinylallene product **3 aa** in 61 % yield along with dimer **4 a** in 10 % yield (Table 1, entry 1). The reaction in the absence of LiOAc⋅2 H_2_O also proceeded smoothly to give **3 aa** in 63 % yield and **4 a** in 10 % yield (Table 1, entry 2, defined as method A), whereas the replacement of acetic acid with acetone as the solvent resulted in a complicated mixture (entry 3). In a solvent study, acetone was found to work as solvent in the presence of acetic acid (5 equiv) for this transformation (Table 1, entry 4, defined as method B). Compared with 60 °C, room temperature or a higher temperature (e.g. 80 °C) were found to give inferior yields of **3 aa** (compare entries 2, 5 and 6 in Table 1). When Pd(OAc)_2_ was replaced with Pd(OOCCF_3_)_2_ or [Pd(acac)_2_], the yield of carbocyclized product **3 aa** decreased (Table 1, entries 7 and 8). No conversion was observed with PdCl_2_ or [PdCl_2_(MeCN)_2_] as the catalyst, and all the starting material **1 a** was recovered in these cases (Table 1, entries 9 and 10). A control experiment without palladium under otherwise the same reaction conditions showed no conversion of **1 a** according to ^1^H NMR spectroscopy. The effect of palladium catalyst loading was also investigated. A lower loading (2 mol %) of Pd(OAc)_2_ gave only 86 % conversion of **1 a** with a yield of **3 aa** of only 50 % (Table 1, entry 11). A higher catalyst loading (10 mol %) also resulted in a lower yield of **3 aa** (Table 1, entry 12).

**Table 1:** Screening of reaction conditions in the palladium-catalyzed oxidative carbocyclization of allenyne 1 a with acetic acid. 
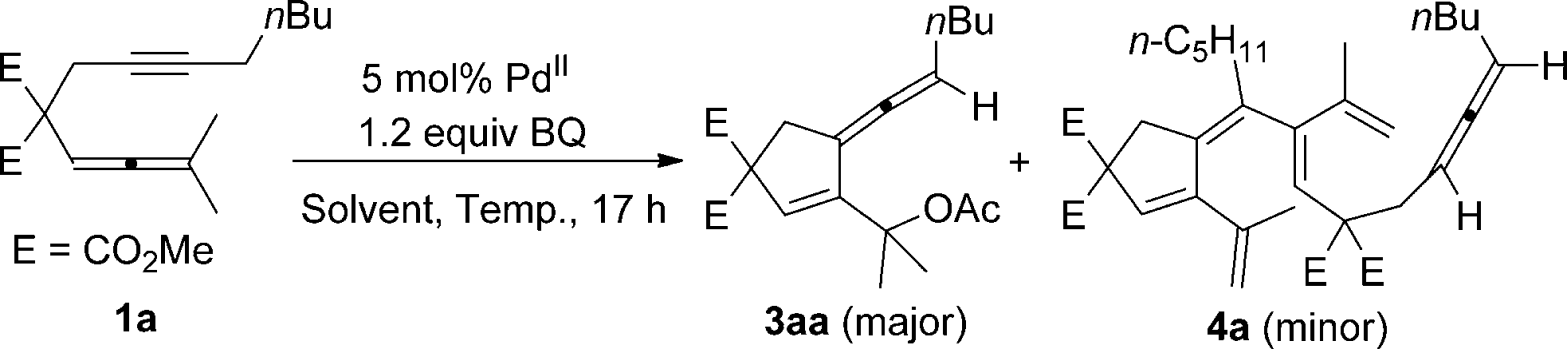

Entry	Pd^II^	Solvent	*T* [°C]	Yield of **3 aa** [%]^a^	Yield of **4 a** [%]^a^
1^b^	Pd(OAc)_2_	HOAc	60	61	10
2	Pd(OAc)_2_	HOAc	60	63(63^c^)	10
3^b^	Pd(OAc)_2_	acetone	60	0	0
4^d^	Pd(OAc)_2_	acetone	60	62(60^c^)	6
5	Pd(OAc)_2_	HOAc	25	<4	0
6	Pd(OAc)_2_	HOAc	80	42	10
7	Pd(OOCCF_3_)_2_	HOAc	60	52	14
8	[Pd(acac)_2_]	HOAc	60	46	8
9	PdCl_2_	HOAc	60	0	0
10	[PdCl_2_(MeCN)_2_]	HOAc	60	0	0
11^e^,^f^	Pd(OAc)_2_	HOAc	60	50	9
12^g^	Pd(OAc)_2_	HOAc	60	38	10

a Yield determined by NMR spectroscopy with anisole as the internal standard.

b LiOAc⋅2H_2_O (2 equiv) was added.

c Yield of isolated product.

d HOAc (5 equiv) was added.

e 2 mol % Pd(OAc)_2_ was used.

f 14 % of **1 a** was recovered.

g 10 mol % Pd(OAc)_2_ was used.

With the optimized conditions in hand, we investigated the scope of allenynes in the presence of acetic acid (Table 2). When both methyl groups on the terminal carbon atom of the allene moiety of **1 a** were replaced by pentamethylene (forming the cyclohexylidene group) (**1 b**), the reaction with acetic acid gave the cyclized vinylallene product **3 ba** in 66 % yield with method A. By altering one methyl group on the allene to an ethyl group, the unsymmetrical allenyne **1 c** displayed a similar reactivity. The reaction of allenyne having an ethyl group (**1 d**) on the triple bond also reacted smoothly to afford product **3 da** in 52 % yield by employing method B. Methyl-substituted allenyne **1 e** gave terminal allene product **3 ea** in 39 % yield. Moreover, the reactions of allenynes bearing two hydroxy or ether groups (**1 g** and **1 h**) instead of the carbomethoxy groups provided the corresponding products **3 ga** and **3 ha** in good yields. Even the allenynes (**1 i** and **1 j**) with the ether as the tether group also worked well and afforded six-membered ring products **3 ia** and **3 ja** in moderate yields, respectively.

**Table 2:** Allenyne scope.^a^ 
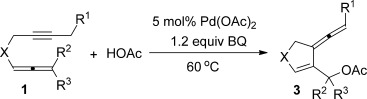

Entry	Allenyne	Product	Yield [%]
1	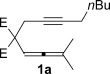	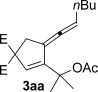	63 (method A) 60 (method B) 62 (method C)
2	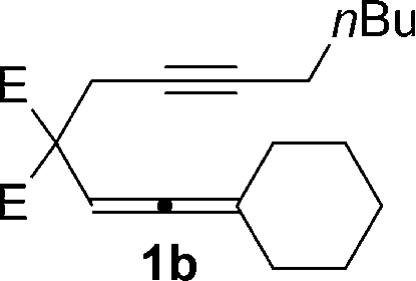	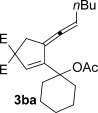	66 (method A) 67 (method C)
3	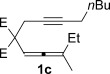	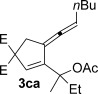	66 (method A) 51 (method C)
4	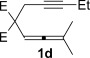	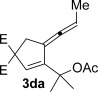	52 (method B)
5^b^	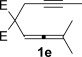	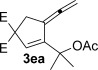	39 (method A)
6	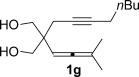	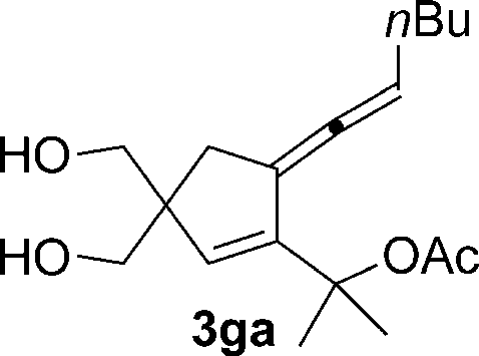	60 (method B)
7	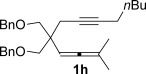	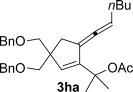	70 (method B)
8^c^	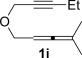	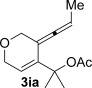	51 (method A)
9^c^	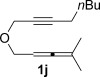	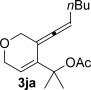	52 (method A)

a Reaction method A: Pd(OAc)_2_ (5 mol %), BQ (1.2 equiv), allenyne (1.0 equiv), HOAc, 60 °C, 17 h; method B: Pd(OAc)_2_ (5 mol %), BQ (1.2 equiv), HOAc (5.0 equiv), allenyne (1.0 equiv), acetone, 60 °C, 17 h; method C: Pd(OAc)_2_ (5 mol %), [Co(salophen)] (5 mol %), BQ (20 mol %), HOAc (5.0 equiv), allenyne (1.0 equiv), acetone, 1 atm O_2_, 60 °C, 18 h.

b Reaction time: 23 h.

c Reaction time: 5 h. E=CO_2_Me.

In addition, the reaction of allenyne (**1 f**) with a phenyl substitution on the alkyne gave no acetoxylation product, but afforded cycloisomerization product **5 f** (8 %),^6^ dimerization products **6 f** (4 %) and **7 f** (29 %); the reaction may be initiated by an allylic C–H bond cleavage on the allene side (Scheme 2).

**Scheme 2:**
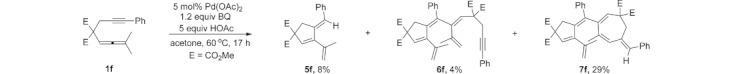
The reaction of phenyl-substituted allenyne **1 f**.

Furthermore, the scope of the reaction with respect to the carboxylic acid coupling partner was also studied by using allenyne **1 b** (Scheme 3). In addition to acetic acid, aliphatic carboxylic acids such as propionic acid or butyric acid reacted smoothly by employing method A to give the cyclized vinylallene products **3 bb** (65 %) and **3 bc** (74 %), respectively. Moreover, benzoic acid and other functionalized aromatic carboxylic acids bearing methoxy, fluorine, or chlorine groups were also tolerated under the oxidative procedure giving the corresponding carbocyclization products in good yields (64–84 %). Interestingly, only trace amounts (<1 %) of dimerization product **4 b** (formed by dimerization of **1 b** through the mechanism shown in Scheme 6) were observed in the reactions in Scheme 3.

**Scheme 3:**
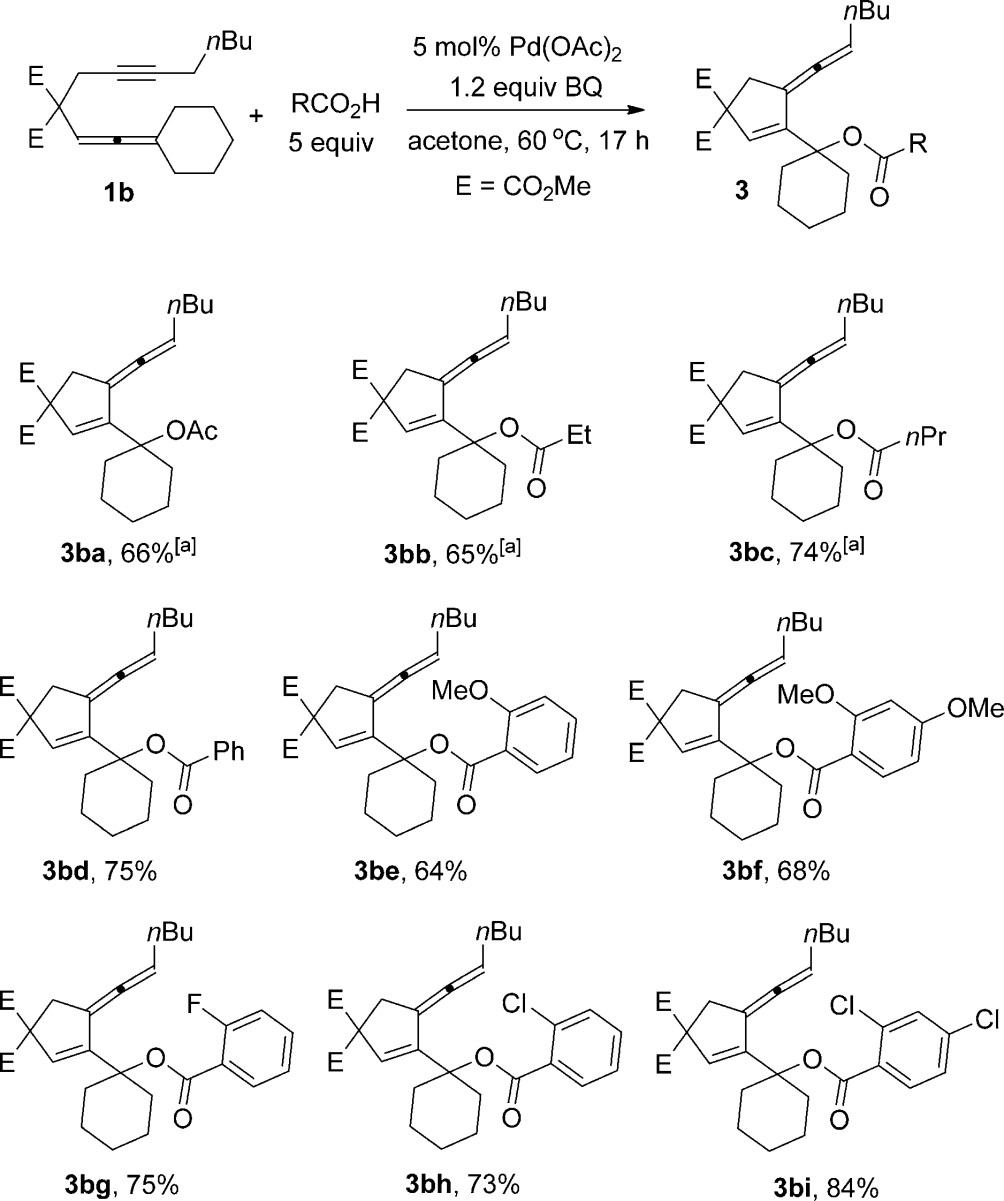
Carboxylic acid scope. [a] Method A was used.

Oxidation processes utilizing molecular oxygen have attracted considerable attention in recent years,^8^ and therefore the oxidative carbocyclization in Table 2 and Scheme 3 was studied under various aerobic conditions. It was found that the combination of cocatalyst [Co(salophen)]^9^ with molecular oxygen (balloon) in the presence of catalytic amounts of BQ (20 mol %) permits the efficient reoxidation of Pd^0^ to Pd^II^ and makes it possible to use O_2_ as the oxidant in the acetoxylation/carbocyclization of allenynes (Table 2, entries 1–3 with method C. For details, please see Scheme S1 in the Supporting Information).

The synthetic potential of the acyloxylated allene products was demonstrated by a few transformations of the representative product **3 aa**. Acetoxyallene **3 aa** was first converted to 3,4-allenol **8** through hydrolysis^10^ (Scheme 4). Under different cyclization conditions,^11^ the prepared 3,4-allenol **8** was subsequently cyclized to various dihydropyran-fused bicyclic skeletons such as **9** (81 %),11a **10** (82 %),11b and **11** (85 %),11c respectively.

**Scheme 4:**
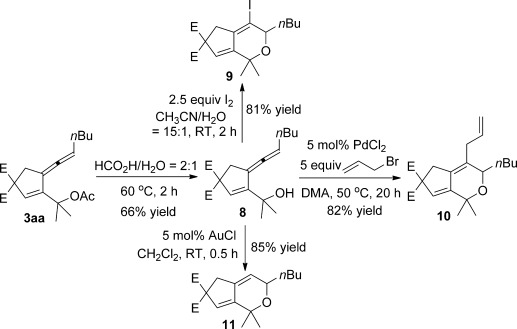
Application of the acyloxylated allene product **3 aa**.

To gain some insight into the reaction mechanism, the deuterium kinetic isotope effect (KIE) was determined from the experiment where a 1:1 mixture of **1 a** and [D_2_]-**1 a** was allowed to react in acetic acid under the reaction conditions used in Table 2 [Eq. (1)]. The product ratio **3 aa**/[D_1_]-**3 aa** at 13 % yield (ca. 35 % conv.) was 4.8:1, and from this ratio the KIE was determined to *k*_H_/*k*_D_=5.5.^12^ Furthermore, the intrinsic KIE from intramolecular competition was determined by the use of [D_1_]-**1 a** as the allenyne substrate. In this case *k*_H_/*k*_D_=6.1 [Eq. (2)]. Parallel kinetic experiments using **1 a** and [D_2_]-**1 a** provided an intermolecular KIE (*k*_H_/*k*_D_, from initial rate) value of 5.1 [Eqs. (3) and (4)]. These results indicate that the propargylic C–H bond cleavage is the rate-determining step in the reaction.^13^


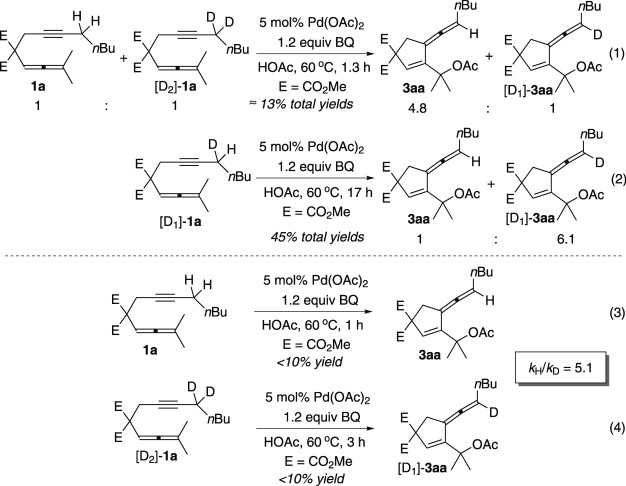


Two control experiments with the deuterium-labeled allenynes [D_6_]-**1 a** and [D_2_]-**1 a** were carried out under the standard conditions. Allenyne [D_6_]-**1 a** gave an increased yield (80 %) of acetoxylated vinylallene ([D_6_]-**3 aa**) compared to the undeuterated allene, whereas the yield of the corresponding dimer products decreased to 2 % (Scheme 5 a). In contrast, the allenyne [D_2_]-**1 a** gave only 20 % yield of acetoxylated vinylallene along with an increased yield (16 %) of the corresponding dimers (Scheme 5 b).

**Scheme 5:**
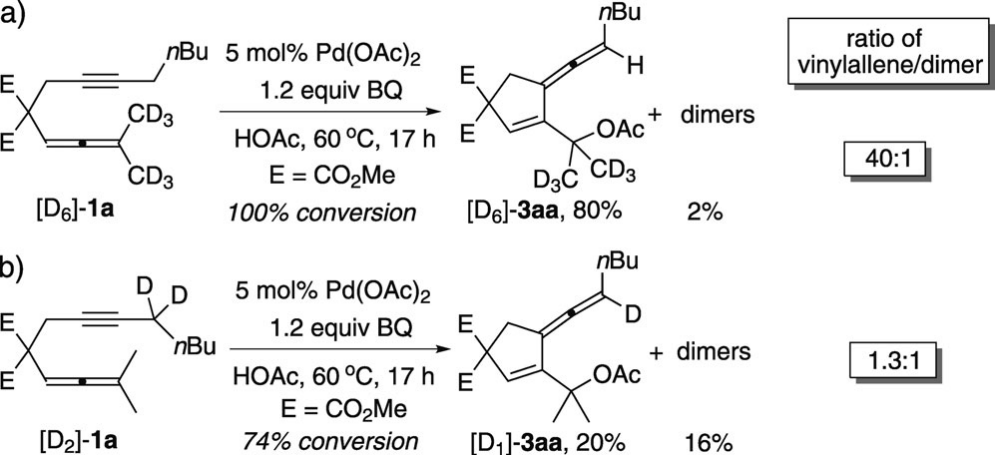
The control reactions of allenynes [D_6_]-**1 a** (a) and [D_2_]-**1 a** (b).

A control experiment replacing the allenyne by an enyne, dimethyl-2-(3′-methylbut-2′-enyl)-2-(pent-2′-ynyl)malonate, was also carried out under the standard conditions of Table 2 using method A. No formation of the corresponding cyclized allene products was observed, which shows that the allene moiety in the substrate is crucial for the oxidative transformation (Scheme S2 in the Supporting Information).

On the basis of these experimental findings, we propose the mechanism shown in Scheme 6. π-Complex formation of **1** with Pd(OAc)_2_ to give chelate **A** and subsequent rearrangement involving a propargylic C–H bond cleavage would produce vinylpalladium intermediate **B**. Intramolecular vinylpalladation of the allene moiety would generate (π-allyl)palladium intermediate **D**, which is attacked by an acetate nucleophile (coordinated or external)^14^ to give **3**. Competing allene attack in **A** through allylic C–H bond cleavage4d and subsequent alkyne insertion would generate intermediate **E**. Reaction of **E** with another molecule of allenyne **1** through insertion of the vinyl–Pd bond of **E** into the allene moiety of **1** would give the π-allyl species **F**, which would yield dimers (**4**, **6**, and **7**; for details, see the Supporting Information). Also, a mechanism involving a pallada(IV)cyclopentene^7^ intermediate **C** could be possible, which would generate intermediates **D** and **E** through β-H elimination and subsequent loss of HOAc leading to product **3** and dimeric by-products, respectively. Although β-H elimination in electron-deficient Pd^IV^ intermediates is considered to be less likely,^15^ β-H elimination from less electron-deficient Pd^IV^ intermediate **C** may occur.

**Scheme 6:**
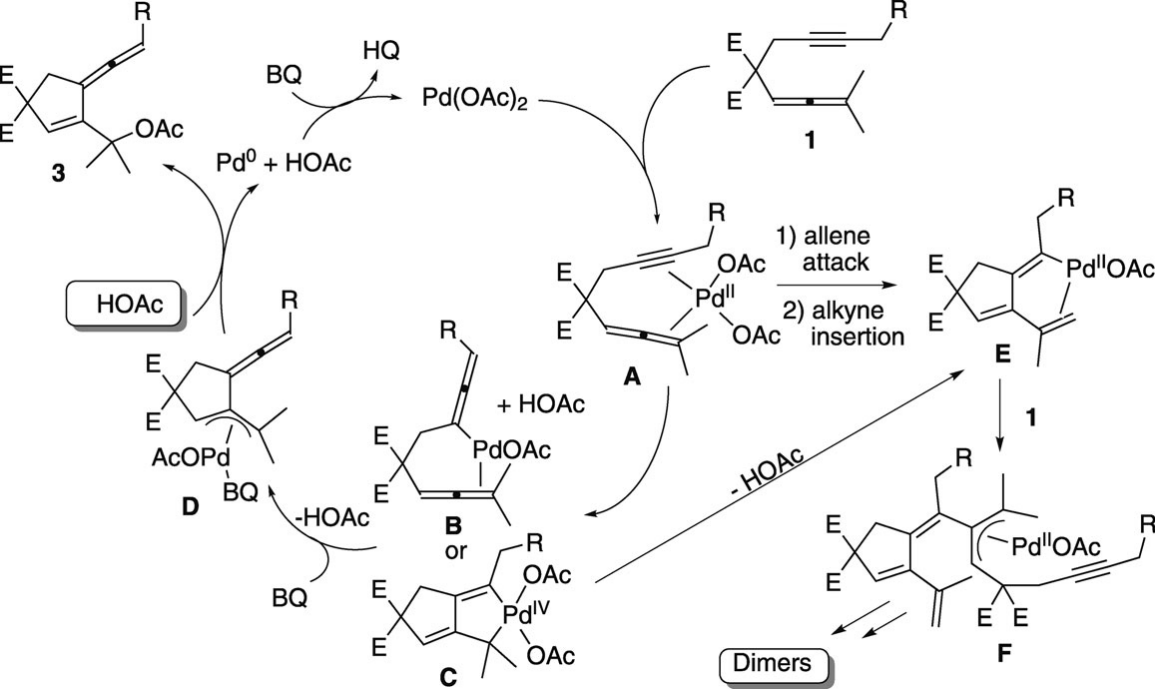
Plausible mechanisms for the palladium-catalyzed oxidative acetoxylation/carbocyclization of allenyne **1**.

One could also consider a mechanism through acetoxypalladation of the terminal C=C double bond of the allene, followed by insertion of the alkyne into the newly generated vinyl–Pd bond and subsequent β-H elimination to give acetoxyallene **3** (for a detailed mechanism, see the Supporting Information). However, with this mechanism one would not obtain any significant change of the ratio between vinylallene **3** and dimers with dideuterated species [D_2_]-**1 a** compared to nondeuterated **1 a**, since with this mechanism the ratio between the competing pathways leading to **3** and dimers would be determined in the first step without any possible isotope effect (see the Supporting Information). The low ratio of 1.3:1 between [D_1_]-**3 aa** and dimers from [D_2_]-**1 a** (Scheme 5 b) therefore rules out this mechanism. In contrast, the two mechanisms proposed in Scheme 6 (via intermediates **B** and **C**, respectively) are in agreement with the results observed in Equations (1)–(4) and Scheme 5.

In summary, we have developed a novel palladium-catalyzed oxidative carbocyclization of allenynes in the presence of various carboxylic acids, providing access to potentially synthetically useful acyloxylated vinylallenes. During this carbocyclization a new C–C bond, a new C–O bond, and a new allene structure are formed. Furthermore, an aerobic version of this transformation using a catalytic amount of BQ was developed to enhance the utility of this method. According to the results of deuterium labeling experiments, we propose that the reaction of the allenynes proceeds through competing propargylic and allylic C–H bond cleavage pathways or via a pallada(IV)cyclopentene intermediate with competing β-eliminations. Further studies on the mechanism and synthetic application of this reaction are ongoing.

## Experimental Section

Typical experimental procedure for palladium-catalyzed oxidative acyloxylation/carbocyclization of allenyne **1**: To a mixture of BQ (26.2 mg, 0.24 mmol) and Pd(OAc)_2_ (2.4 mg, 0.01 mmol) were added **1 b** (69.5 mg, 0.20 mmol) and HOAc (0.4 mL) at room temperature. The reaction was stirred at 60 °C for 17 h. After full consumption of starting material **1 b**, as monitored by TLC, the reaction was cooled to room temperature, diluted with Et_2_O (20 mL), and quenched with H_2_O (5 mL). The organic phase was separated and the aqueous phase was extracted with Et_2_O (2×20 mL). The combined organic layers were washed with H_2_O and dried over anhydrous Na_2_SO_4_. Evaporation and column chromatography on silica gel (pentane/ethyl acetate=10:1) afforded **3 ba** (53.3 mg, 66 %) as a liquid; ^1^H NMR (400 MHz, CDCl_3_): *δ*=5.74 (d, *J*=1.2 Hz, 1 H), 5.34–5.24 (m, 1 H), 3.73 (s, 3 H), 3.72 (s, 3 H), 3.18 (d, *J*=3.6 Hz, 2 H), 2.46–2.38 (m, 1 H), 2.36–2.24 (m, 1 H), 2.08–1.98 (m, 2 H), 1.97 (s, 3 H), 1.66–1.46 (m, 7 H), 1.45–1.18 (m, 5 H), 0.89 ppm (t, *J*=7.2 Hz, 3 H); ^13^C NMR (100 MHz, CDCl_3_): *δ*=198.1, 170.8, 170.7, 169.1, 149.4, 125.3, 104.2, 95.0, 79.7, 63.5, 52.9, 52.8, 36.6, 34.8, 33.6, 31.2, 29.2, 25.4, 22.1, 21.6, 21.52, 21.50, 13.9 ppm; HRMS (ESI): calc. for C_23_H_32_NaO_6_ [*M*+Na]^+^: 427.2091; found: 427.2091.

## Supplementary material

As a service to our authors and readers, this journal provides supporting information supplied by the authors. Such materials are peer reviewed and may be re-organized for online delivery, but are not copy-edited or typeset. Technical support issues arising from supporting information (other than missing files) should be addressed to the authors.
