# Supplementary material for: Palladium-Catalyzed Oxidative Acyloxylation/Carbocyclization of Allenynes
Source: Angew Chem Int Ed Engl. 2013 Feb 5;52(11):3217–21. doi: 10.1002/anie.201208718 (PMC3601422; doi:10.1002/anie.201208718)
Supplement: Supplementary file 1 [file anie0052-3217-SD1.pdf]

Angewandte Chemie
Eine Zeitschrift der Gesellschaft Deutscher Chemiker

Supporting Information
© Wiley-VCH 2013
69451 Weinheim, Germany

# Palladium-Catalyzed Oxidative Acyloxylation/Carbocyclization of Allenynes**

*Youqian Deng and Jan-E. Bäckvall**

anie_201208718_sm_miscellaneous_information.pdf

# Contents


| | |
|---|---|
| General remarks | S2 |
| Preparation of allenynes | S3 |
| General procedure for oxidative acyloxylation/carbocyclization | S13 |
| The reaction of phenyl-substituted allenyne **1f** | S22 |
| General procedure for aerobic acetoxylation/carbocyclization | S24 |
| Application of product **3aa** | S25 |
| Kinetic isotope effect experiments | S28 |
| Detailed mechanism for the formation of products **4**, **6**, and **7** | S34 |
| Control experiment using enyne **12** | S35 |
| References | S36 |
| Copies of spectra | S37 |

## Experimental Section

### *General remarks*

Unless otherwise noted, all reagents were used as received from the commercial suppliers. HOAc was commercially available from Aldrich. $Pd(OAc)_2$ was obtained from Pressure Chemicals and used without further purification. Palladium-catalyzed cyclizations were performed without any efforts to exclude moisture. Dry solvents ($Et_2O$, THF) were obtained from a VAC Solvent Purifier. Reactions were monitored using thin-layer chromatography ($SiO_2$). TLC plates were visualized with UV light (254 nm) or $KMnO_4$ stain. Flash chromatography was carried out with 60Å (particle size 35-70 μm) normal flash silica gel. NMR spectra were recorded at 400 MHz ($^1H$) or 500 MHz ($^1H$) and at 100 MHz ($^{13}C$) or 125 MHz ($^{13}C$), respectively. Chemical shifts (δ) are reported in ppm, using the residual solvent peak in $CDCl_3$ ($\delta_H$ = 7.26 and $\delta_C$ = 77.0 ppm) as internal standard, and coupling constants (*J*) are given in Hz. HRMS were recorded using ESI-TOF techniques.

***General procedure for preparation of starting materials***

All bromoallenes were prepared according to the procedure published by Landor[1a] with minor modifications.[1b]

Dimethyl propargylmalonate is commercially available from Aldrich. Other alkyl substituted dimethyl propargylmalonates were prepared as described in the literature.[2]

Allenynes **1a-f** were prepared as previously described.[3]

**1. Synthesis of allenyne [D$_2$]-1a.**

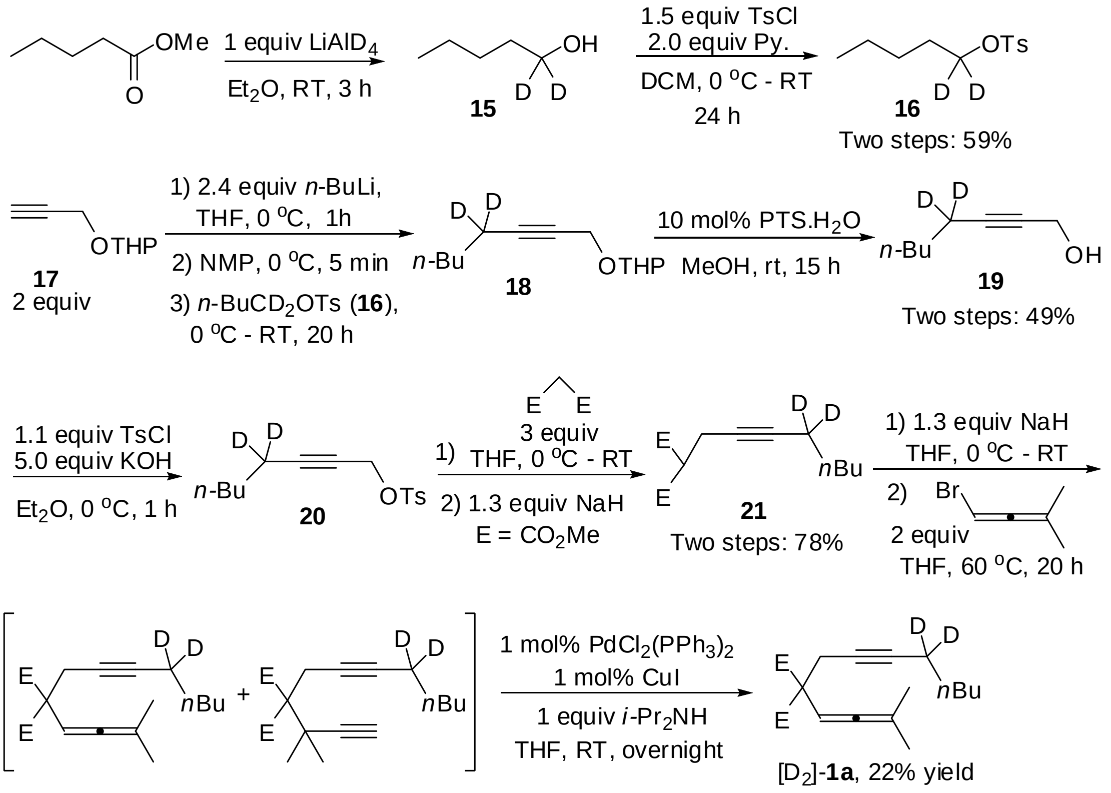


*[D$_2$]-Pentyl 4-methylbenzenesulfonate (**16**)*[4]

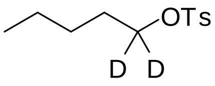


A solution of methyl pentanoate (13.810 g, 119.0 mmol) in dry Et$_2$O (100 mL) was added dropwise to a stirred suspension of LiAlD$_4$ (5 g, 119.0 mmol) in dry Et$_2$O (200

mL) at 0 °C over 15 min. The mixture was stirred for another 3 h at rt and carefully quenched with $D_2O$ (10 mL). The resulting mixture was filtered to remove the precipitate and the filtrate was dried over $Na_2SO_4$. Evaporation afforded crude **15** (8.443 g). The alcohol **15** (8.443 g, ~93.8 mmol) was dissolved in $CH_2Cl_2$ and cooled in an ice bath (0 °C). Pyridine (14.820 g, 187.6 mmol) was then added, followed by *p*-toluenesulfonyl chloride (26.817 g, 140.7 mmol) in small portions with constant stirring. The reaction mixture was warmed to rt and stirred for 24 h. Ether (200 mL) and water (50 mL) were added and the organic layer was washed successively with 2 N HCl, 5% $NaHCO_3$, and water and then dried ($Na_2SO_4$). Evaporation and column chromatography on silica gel (pentane/ethyl ether = 50:1) afforded **16** (17.107 g, 59% yield for two steps): liquid; $^1$H NMR (500 MHz, $CDCl_3$): 7.79 (d, *J* = 8.5 Hz, 2H), 7.34 (d, *J* = 8.5 Hz, 2H), 2.45 (s, 3H), 1.64-1.56 (m, 2H), 1.40-1.18 (m, 4H), 0.85 (t, *J* = 7.0 Hz, 3H); $^{13}$C NMR (125 MHz, $CDCl_3$): δ 144.6, 133.3, 129.8, 127.9, 28.3, 27.4, 22.0, 21.6, 13.8; HRMS (ESI): calc. for $C_{12}H_{16}D_2NaO_3S$ $[M+Na]^+$: 267.0994; found: 267.0997.

*[D₂]-Oct-2-yn-1-ol (**19**)*[5]

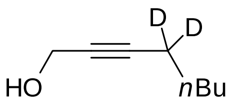


To a solution of THP ether **17** (8.424 g, 60.0 mmol) in 20 mL of THF was added *n*-BuLi (29 mL, 72.5 mmol, 2.5 M in hexane) at 0 °C over 15 min. After the resulting slurry was stirred at 0 °C for another 45 min, 40 mL of *N*-methyl-2-pyrrolidone (NMP) was added at 0 °C and the mixture was stirred for 5 min. Then a solution of **16** (7.365 g, 30.0 mmol) in 20 mL of NMP was added dropwise at 0 °C over 10 min. The reaction mixture was allowed to react at rt, stirred for 20 h, and quenched with 20 mL of water, extracted with diethyl ether (3 x 40 mL). The combined organic layer was washed with water. After drying over *anhydrous* $Na_2SO_4$, the extract was concentrated and the followed bulb-to-bulb distillation removed most of the residual **17** and afforded crude **18** (4.454 g, containing 5% of **17**).

To a solution of THP ether **18** (4.454 g, ~21 mmol) in 21 mL of MeOH was added

PTS·$H_2O$ (399.2 mg, 2.1 mmol) at rt. The mixture was stirred at rt for 15 h. After full consumption of starting material **18**, as monitored by TLC, MeOH was removed *in vacuo*. The resulting mixture was purified by column chromatography on silica gel (pentane/ethyl acetate = 10:1) affording **19** (1.910 g, 49% total yields for two steps): liquid; $^1$H NMR (500 MHz, $CDCl_3$): 4.25 (d, $J$ = 6.0 Hz, 2H), 1.52-1.42 (m, 3H), 1.40-1.22 (m, 4H), 0.90 (t, $J$ = 7.0 Hz, 3H); $^{13}$C NMR (125 MHz, $CDCl_3$): δ 86.7, 78.3, 51.4, 31.0, 28.1, 22.2, 13.9.

*[$D_2$]-Dimethyl 2-(oct-2-ynyl)malonate (**21**)*[6]

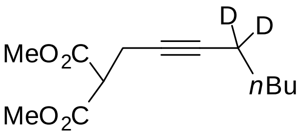


To a solution of **19** (1.910 g, 14.9 mmol) and TsCl (3.129 g, 16.4 mmol) in 50 mL of $Et_2O$ at 0 °C was added in portions over 10 min powdered KOH (4.188 g, 74.8 mmol). The resulting slurry was stirred at 0 °C for 1 h, then quenched with $H_2O$ (20 mL). The aqueous layer was extracted with ether (2 x 30 mL). The combined organic layer was washed with $H_2O$ (10 mL), dried over $Na_2SO_4$, concentrated, giving the crude tosylate (**20**) for next step without further purification.

To an ice cooled suspension of NaH (880.0 mg, 22.0 mmol, 60% purity) in THF (50 mL), a solution of dimethyl malonate (5.941 g, 45.0 mmol) in THF (10 mL) was added dropwise over a period of 10 min. After removal of the ice bath, the mixture was stirred at rt for 50 min. Then crude tosylate (**20**) in THF (10 mL) was added dropwise over a period of 10 min at 0 °C and the mixture was stirred at 50 °C for an additional 20 h. The mixture was concentrated, diluted with ether (50 mL), and quenched with $H_2O$ (20 mL). The phases were separated and the aqueous layer was extracted with diethyl ether (2 x 30 mL). The combined organic layers were washed with $H_2O$ (20 mL), dried ($Na_2SO_4$) and the solvent was removed under reduced pressure. The crude product was purified by column chromatography (pentane/ethyl acetate = 30:1) to yield **21** (2.810 g, 78% total yields for two steps): liquid; $^1$H NMR (500 MHz, $CDCl_3$): 3.74 (s, 6H), 3.55 (t, $J$ = 8.0 Hz, 1H), 2.73 (d, $J$ = 8.0 Hz, 2H),

1.44-1.36 (m, 2H), 1.32-1.21 (m, 4H), 0.88 (t, *J* = 7.0 Hz, 3H); $^{13}$C NMR (125 MHz, $CDCl_3$): δ 168.6, 82.7, 75.4, 52.6, 51.6, 30.8, 28.2, 22.2, 18.9, 13.9; HRMS (ESI): calc. for $C_{13}H_{18}D_2NaO_4$ $[M+Na]^+$: 265.1379; found: 265.1364.

*[D₂]-Dimethyl 2-(3-methylbuta-1,2-dienyl)-2-(oct-2-ynyl)malonate ([D₂]-**1a**)*

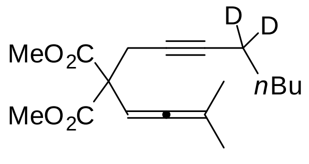


To a suspension of NaH (60% in mineral oil, 580.9 mg, 14.5 mmol) in anhydrous THF (60 ml) was added a solution of **21** (2.403 g, 9.9 mmol) in anhydrous THF (5 ml) at 0 ºC. After the addition, the mixture was stirred for another 20 min at rt. Then a solution of 1-bromo-3,3-dimethylallene (3.246 g, 22.1 mmol) in anhydrous THF (5 ml) was added at rt and the resulting mixture was stirred at 60 $^{o}$C for 20 h. After the reaction was complete as monitored by TLC, it was cooled to room temperature. Most of the solvent was removed under vacuum, and then the reaction mixture was diluted with 50 mL of $Et_2O$ and quenched with 10 mL of water. The organic layer was separated, and the aqueous layer was extracted with diethyl ether (2 x 20 mL). The combined organic layers were dried over $Na_2SO_4$, concentrated. After a short column, a crude mixture of allenyne ([$D_2$]-**1a**) and the corresponding isomeric diyne were obtained and used for the next step without further purification.

To a solution of allenyne ([$D_2$]-**1a**) and the corresponding diyne in THF (11 mL) at rt were added $PdCl_2(PPh_3)_2$ (78.2 mg, 0.11 mmol), CuI (22.0 mg, 0.12 mmol), and *i*-$Pr_2NH$ (1.1510 g, 11.4 mmol). The resulting mixture was stirred at rt for 17 h. (By using this typical Sonogashira coupling condition, the terminal alkyne could be completely converted to the corresponding dimerization product while the allenyne is still stable under this condition. Therefore, the allenyne could be separated easily because of the large difference in polarity between allenyne and dimer).[3b] Evaporation, column chromatography directly on silica gel (pentane/ethyl ether = 50/1), and bulb-to-bulb distillation (in order to remove the residual Pd or Cu) afforded [$D_2$]-**1a** (662.8 mg, 22%): liquid; $^1$H NMR (500 MHz, $CDCl_3$): δ 5.59-5.53 (m, 1H), 3.74 (s,

6H), 2.84 (s, 2H), 1.71 (d, *J* = 3.0 Hz, 6H), 1.46-1.38 (m, 2H), 1.36-1.22 (m, 4H), 0.89 (t, *J* = 7.0 Hz, 3H); $^{13}$C NMR (125 MHz, $CDCl_3$): δ 201.5, 170.0, 100.5, 88.0, 82.8, 74.9, 58.2, 52.8, 30.9, 28.4, 24.9, 22.2, 20.0, 14.0; HRMS (ESI): calc. for $C_{18}H_{24}D_2NaO_4$ $[M+Na]^+$: 331.1849; found: 331.1841.

## 2. Synthesis of allenyne [$D_1$]-la.

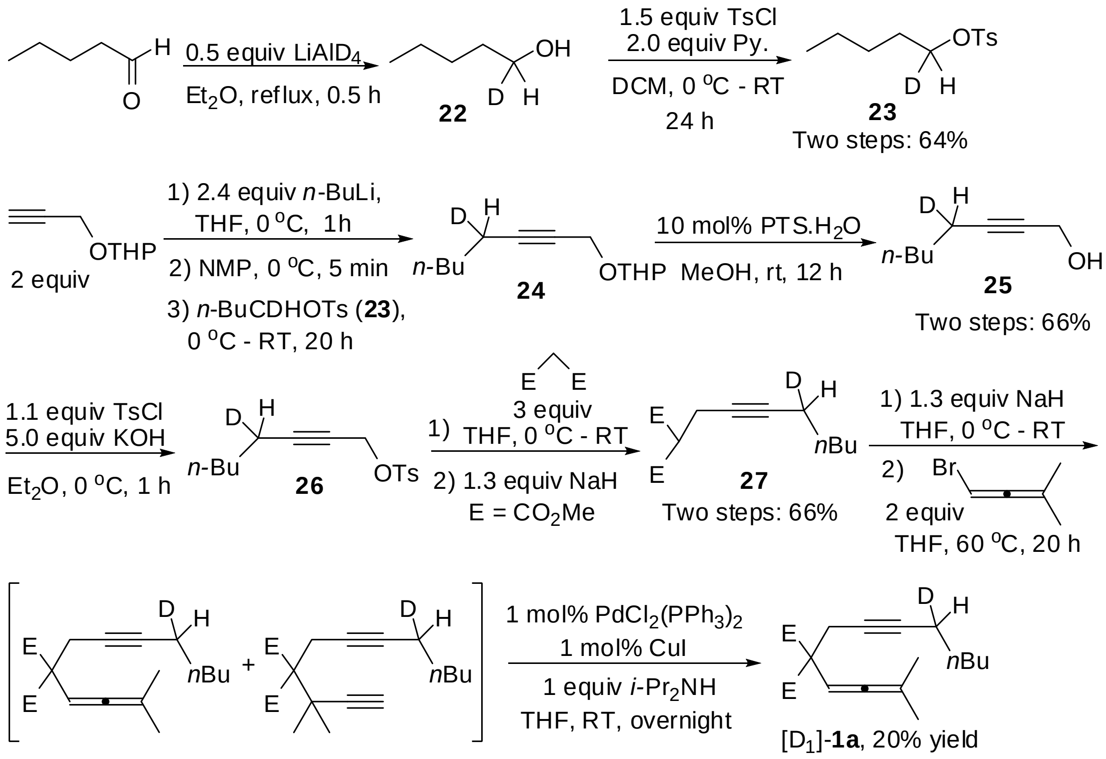


The allenyne [$D_1$]-**la** was prepared according to the procedures for the preparation of [$D_2$]-**1a**.

*[$D_1$]-Pentyl 4-methylbenzenesulfonate* (**23**)

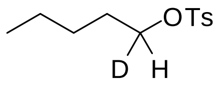

Liquid. $^1$H NMR (500 MHz, $CDCl_3$): 7.79 (d, *J* = 8.0 Hz, 2H), 7.34 (d, *J* = 8.0 Hz, 2H), 4.00 (t, *J* = 6.5 Hz, 1H), 2.45 (s, 3H), 1.64-1.56 (m, 2H), 1.40-1.18 (m, 4H), 0.85 (t, *J* = 7.0 Hz, 3H); $^{13}$C NMR (125 MHz, $CDCl_3$): δ 144.6, 133.3, 129.8, 127.9, 70.4 (t, *J* = 23.8 Hz), 28.4, 27.4, 22.0, 21.6, 13.8; HRMS (ESI): calc. for $C_{12}H_{17}DNaO_3S$ $[M+Na]^+$: 266.0932; found: 266.0934.

*[D$_1$]-Oct-2-yn-1-ol (**25**)*

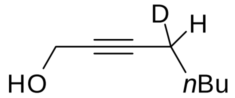


Liquid. $^1$H NMR (500 MHz, $CDCl_3$): 4.24 (dd, $J_1$ = 6.0 Hz, $J_2$ = 1.5 Hz, 2H), 2.22-2.14 (m, 1H), 1.65 (t, $J$ = 6.0 Hz, 1H), 1.52-1.44 (m, 2H), 1.40-1.22 (m, 4H), 0.91 (t, $J$ = 7.0 Hz, 3H); $^{13}$C NMR (125 MHz, $CDCl_3$): δ 86.6, 78.3, 51.4, 31.0, 28.2, 22.2, 18.4 (t, $J$ = 20.0 Hz), 13.9.

*[D$_1$]-Dimethyl 2-(oct-2-ynyl)malonate (**27**)*

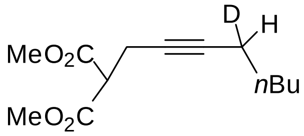


Liquid. $^1$H NMR (500 MHz, $CDCl_3$): 3.75 (s, 6H), 3.56 (t, $J$ = 8.0 Hz, 1H), 2.74 (dd, $J_1$ = 8.0 Hz, $J_2$ = 2.0 Hz, 2H), 2.12-2.04 (m, 1H), 1.44-1.38 (m, 2H), 1.36-1.21 (m, 4H), 0.89 (t, $J$ = 7.0 Hz, 3H); $^{13}$C NMR (125 MHz, $CDCl_3$): δ 168.6, 82.7, 75.4, 52.7, 51.6, 30.9, 28.4, 22.2, 18.9, 18.3 (t, $J$ = 20.0 Hz), 13.9; HRMS (ESI): calc. for $C_{13}H_{19}DNaO_4$ $[M+Na]^+$: 264.1317; found: 264.1333.

*[D$_1$]-Dimethyl 2-(3-methylbuta-1,2-dienyl)-2-(oct-2-ynyl)malonate ([D$_1$]-**1a**)*

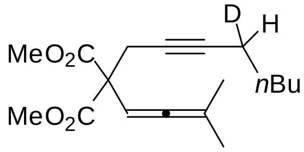


Liquid. $^1$H NMR (500 MHz, $CDCl_3$): δ 5.59-5.53 (m, 1H), 3.73 (s, 6H), 2.83 (d, $J$ = 2.5 Hz, 2H), 2.10-2.02 (m, 1H), 1.70 (d, $J$ = 2.5 Hz, 6H), 1.44-1.36 (m, 2H), 1.35-1.20 (m, 4H), 0.88 (t, $J$ = 7.0 Hz, 3H); $^{13}$C NMR (125 MHz, $CDCl_3$): δ 201.5, 170.0, 100.4, 88.0, 82.8, 74.8, 58.1, 52.7, 30.9, 28.5, 24.9, 22.2, 20.0, 18.3 (t, $J$ = 20.0 Hz), 13.9; HRMS (ESI): calc. for $C_{18}H_{25}DNaO_4$ $[M+Na]^+$: 330.1786; found: 330.1780.

**3. Synthesis of allenyne [D$_6$]-la.**

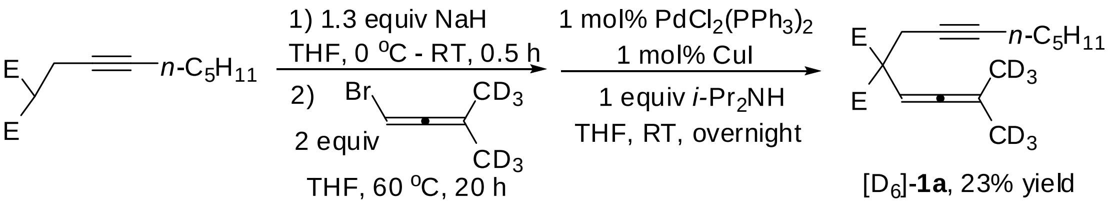


The allenyne [$D_6$]-**la** was prepared according to the procedure for the preparation of [$D_2$]-**1a**.

*[$D_6$]-Dimethyl 2-(3-methylbuta-1,2-dienyl)-2-(oct-2-ynyl)malonate ([$D_6$]-**la**)*

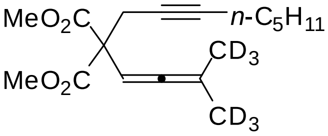

Liquid. $^1$H NMR (500 MHz, $CDCl_3$): δ 5.56 (s, 1H), 3.74 (s, 6H), 2.84 (t, *J* = 2.5 Hz, 2H), 2.14-2.05 (m, 2H), 1.48-1.38 (m, 2H), 1.36-1.22 (m, 4H), 0.88 (t, *J* = 7.0 Hz, 3H); $^{13}$C NMR (125 MHz, $CDCl_3$): δ 201.5, 170.0, 100.2, 88.0, 82.9, 74.8, 58.1, 52.8, 30.9, 28.6, 24.9, 22.2, 18.6, 14.0; HRMS (ESI): calc. for $C_{18}H_{20}D_6NaO_4$ $[M+Na]^+$: 335.2100; found: 335.2095.

**4. Synthesis of allenynes 1g and 1h.**[7]

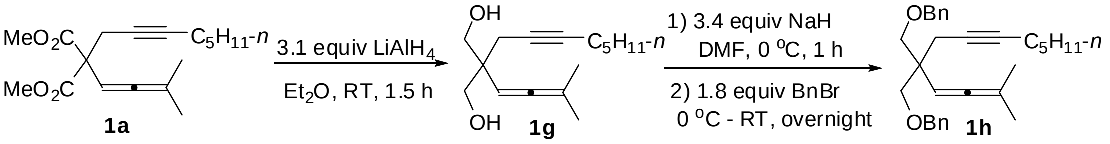


*2-(3-Methylbuta-1,2-dienyl)-2-(oct-2-ynyl)propane-1,3-diol (**1g**)*

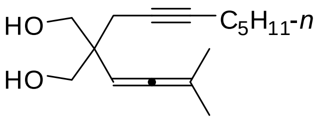

To a suspension of $LiAlH_4$ (354 mg, 9.3 mmol) in anhydrous $Et_2O$ (30 ml) was added a solution of **1a** (913 mg, 3.0 mmol) in anhydrous $Et_2O$ (20 ml) at rt. After the addition, the mixture was stirred for another 1.5 h at rt and carefully quenched with $H_2O$ (5 mL). The resulting mixture was extracted with diethyl ether (2 x 30 mL). The combined organic layers were washed with $H_2O$ (20 mL), dried ($Na_2SO_4$) and the solvent was removed under reduced pressure. The crude product was purified by

column chromatography (pentane/ethyl acetate = 4:1) to yield **1g** (512 mg, 69% yield): liquid; $^1$H NMR (500 MHz, $CDCl_3$): δ 4.97-4.92 (m, 1H), 3.67 (bs, 4H), 2.33 (t, *J* = 2.5 Hz, 2H), 2.27 (bs, 2H), 2.16-2.10 (m, 2H), 1.71 (d, *J* = 3.0 Hz, 6H), 1.50-1.42 (m, 2H), 1.38-1.26 (m, 4H), 0.88 (t, *J* = 7.0 Hz, 3H); $^{13}$C NMR (125 MHz, $CDCl_3$): δ 201.6, 98.0, 90.6, 83.0, 76.3, 67.5, 44.7, 31.1, 28.7, 23.4, 22.2, 20.6, 18.7, 14.0; HRMS (ESI): calc. for $C_{16}H_{26}D_2NaO_2$ $[M+Na]^+$: 273.1825; found: 273.1816.

*(2-(3-Methylbuta-1,2-dienyl)-2-(oct-2-ynyl)propane-1,3-diyl)bis(oxy)bis(methylene)dibenzene (**1h**)*

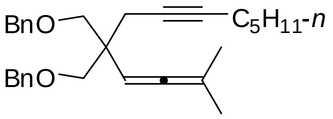


To a suspension of NaH (60% in mineral oil, 108.8 mg, 2.7 mmol) in anhydrous DMF (3 ml) was added a solution of **1g** (200 mg, 0.8 mmol) in anhydrous DMF (2 ml) at 0 ºC. After the addition, the mixture was stirred for another 1 h at 0 ºC. Then BnBr (170 μL, 1.4 mmol) was added at 0 ºC and the resulting mixture was stirred at rt for 13 h. Then the reaction mixture was diluted with 20 mL of $Et_2O$ and quenched with 5 mL of water. The organic layer was separated, and the aqueous layer was extracted with diethyl ether (2 x 20 mL). The combined organic layers were washed with $H_2O$ (10 mL), dried ($Na_2SO_4$) and the solvent was removed under reduced pressure. The crude product was purified by column chromatography (pentane/diethyl ether = 100:1) to yield **1h** (284 mg, 92% yield): liquid; $^1$H NMR (500 MHz, $CDCl_3$): 7.40-7.20 (m, 10H), 5.18-5.12 (m, 1H), 4.56 (s, 4H), 3.56 (d, *J* = 9.0 Hz, 2H), 3.54 (d, *J* = 9.0 Hz, 2H), 2.43 (t, *J* = 2.5 Hz, 2H), 2.19-2.12 (m, 2H), 1.73 (d, *J* = 3.0 Hz, 6H), 1.52-1.43 (m, 2H), 1.42-1.29 (m, 4H), 0.93 (t, *J* = 7.0 Hz, 3H); $^{13}$C NMR (125 MHz, $CDCl_3$): δ 201.4, 138.9, 128.1, 127.23, 127.20, 97.1, 91.4, 82.0, 76.9, 73.3, 44.0, 31.1, 28.8, 23.9, 22.2, 20.6, 18.8, 14.0; HRMS (ESI): calc. for $C_{30}H_{38}NaO_2$ $[M+Na]^+$: 453.2764; found: 453.2766.

**5. Synthesis of allenynes 1i and 1j.**

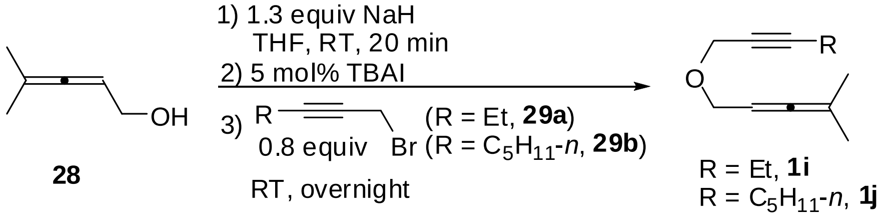


*4-Methyl-1-(pent-2-ynyloxy)penta-2,3-diene (**1i**)*

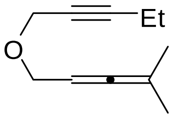


To a suspension of NaH (60% in mineral oil, 110 mg, 2.6 mmol) in anhydrous THF (16 ml) was added a solution of **28** (200 mg, 2.0 mmol) in anhydrous THF (2 ml) at rt. After the addition, the mixture was stirred for another 20 min at rt. Then tetrabutyl-ammonium iodide (TBAI) (37 mg, 0.1 mmol) and **29a** (230 mg, 1.6 mmol) were sequentially added and the resulting mixture was stirred at rt for 15 h. Most of the solvent was removed under vacuum, and then the reaction mixture was diluted with 30 mL of $Et_2O$ and quenched with 10 mL of water. The organic layer was separated, and the aqueous layer was extracted with diethyl ether (2 x 20 mL). The combined organic layers were washed with $H_2O$ (10 mL), dried ($Na_2SO_4$) and the solvent was removed under reduced pressure. The crude product was purified by column chromatography (pentane/ethyl acetate = 100:1) to yield **1i** (221 mg, 86% yield): liquid; $^1$H NMR (400 MHz, $CDCl_3$): 5.08-4.96 (m, 1H), 4.12 (t, $J$ = 2.0 Hz, 2H), 4.00 (d, $J$ = 6.8 Hz, 2H), 2.26-2.16 (m, 2H), 1.69 (d, $J$ = 3.0 Hz, 6H), 1.13 (t, $J$ = 7.2 Hz, 3H); $^{13}$C NMR (100 MHz, $CDCl_3$): δ 203.2, 95.9, 88.1, 85.7, 75.2, 68.3, 57.0, 20.3, 13.7, 12.4; HRMS (ESI): calc. for $C_{11}H_{16}NaO$ $[M+Na]^+$: 187.1093; found: 187.1101.

The allenyne **li** was prepared according to the procedure for the preparation of **1j**.

*1-(4-Methylpenta-2,3-dienyloxy)oct-2-yne (**1j**)*

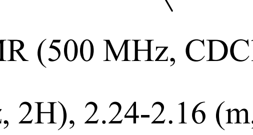


94% isolated yield, liquid. $^1$H NMR (500 MHz, $CDCl_3$): 5.08-5.00 (m, 1H), 4.14 (t, $J$ = 2.0 Hz, 2H), 4.02 (d, $J$ = 7.0 Hz, 2H), 2.24-2.16 (m, 2H), 1.70 (d, $J$ = 2.5 Hz, 6H), 1.54-1.47 (m, 2H), 1.40-1.26 (m, 4H), 0.89 (t, $J$ = 7.0 Hz, 3H); $^{13}$C NMR (125 MHz,

$CDCl_3$): δ 203.3, 96.0, 86.9, 85.7, 75.8, 68.3, 57.1, 31.0, 28.3, 22.2, 20.4, 18.7, 13.9; HRMS (ESI): calc. for $C_{14}H_{22}NaO$ $[M+Na]^+$: 229.1563; found: 229.1559.

***General procedure for oxidative acyloxylation/carbocyclization for the formation of vinylallenes 3***

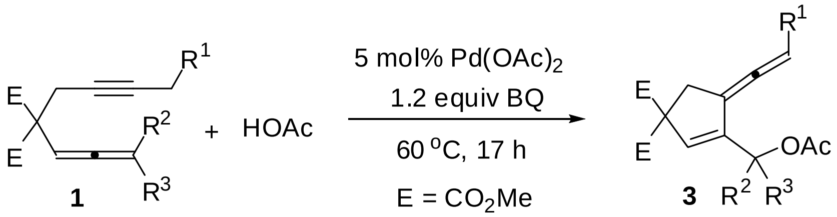


*Representative procedure A for the synthesis of **3**. Dimethyl 3-(2-acetoxypropan-2-yl)-4-(hex-1-enylidene)cyclopent-2-ene-1,1-dicarboxylate (**3aa**)*

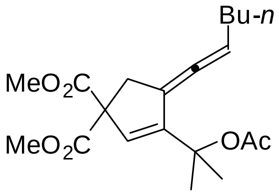


To a mixture of BQ (25.8 mg, 0.24 mmol) and $Pd(OAc)_2$ (2.1 mg, 0.01 mmol) were added **1a** (60.2 mg, 0.20 mmol) and 0.4 mL of HOAc at rt. The reaction was stirred at 60 °C for 17 h. After full consumption of starting material **1a**, as monitored by TLC, the reaction was cooled to room temperature, diluted with $Et_2O$ (20 mL), and quenched with 5 mL of $H_2O$. The organic phase was separated and the aqueous phase was extracted with $Et_2O$ (2 x 20 mL). The combined organic layers were washed with $H_2O$ and dried over *anhydrous* $Na_2SO_4$. Evaporation and column chromatography on silica gel (pentane/ethyl acetate = 10/1) afforded **3aa** (45.3 mg, 63%) as a liquid: $^1H$ NMR (500 MHz, $CDCl_3$): 5.78 (d, $J$ = 1.5 Hz, 1H), 5.40-5.30 (m, 1H), 3.75 (s, 3H), 3.74 (s, 3H), 3.22 (d, $J$ = 3.5 Hz, 2H), 2.10-2.00 (m, 2H), 1.96 (s, 3H), 1.63 (s, 3H), 1.59 (s, 3H), 1.44-1.30 (m, 4H), 0.90 (t, $J$ = 7.0 Hz, 3H); $^{13}C$ NMR (125 MHz, $CDCl_3$): δ 198.3, 170.8, 170.7, 169.5, 149.9, 125.0, 104.3, 95.5, 79.2, 63.3, 52.94, 52.91, 36.7, 31.2, 29.1, 26.5, 26.0, 22.2, 22.0, 13.9; HRMS (ESI): calc. for $C_{20}H_{28}NaO_6$ $[M+Na]^+$: 387.1778; found: 387.1777.

*$[D_6]$-Dimethyl 3-(2-acetoxypropan-2-yl)-4-(hex-1-enylidene)cyclopent-2-ene-1,1-dicarboxylate ($[D_6]$-**3aa**)*

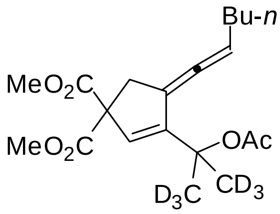


80% isolated yield, liquid. $^{1}$H NMR (500 MHz, $CDCl_3$): 5.76 (d, $J$ = 1.5 Hz, 1H), 5.36-5.28 (m, 1H), 3.73 (s, 3H), 3.72 (s, 3H), 3.21 (d, $J$ = 3.5 Hz, 2H), 2.10-2.00 (m, 2H), 1.95 (s, 3H), 1.42-1.28 (m, 4H), 0.88 (t, $J$ = 7.0 Hz, 3H); $^{13}$C NMR (125 MHz, $CDCl_3$): δ 198.2, 170.8, 170.7, 169.4, 149.8, 124.9, 104.3, 95.5, 78.9, 63.3, 52.88, 52.86, 36.7, 31.1, 29.1, 22.1, 21.9, 13.8; HRMS (ESI): calc. for $C_{20}H_{22}D_6NaO_6$ $[M+Na]^+$: 393.2155; found: 393.2154.

*Dimethyl 3-(1-acetoxycyclohexyl)-4-(hex-1-enylidene)cyclopent-2-ene-1,1-dicarboxylate (**3ba**)*

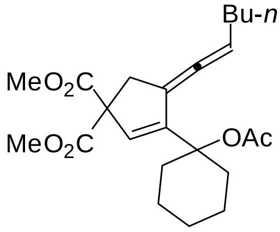


66% isolated yield, liquid. $^{1}$H NMR (400 MHz, $CDCl_3$): 5.74 (d, $J$ = 1.2 Hz, 1H), 5.34-5.24 (m, 1H), 3.73 (s, 3H), 3.72 (s, 3H), 3.18 (d, $J$ = 3.6 Hz, 2H), 2.46-2.38 (m, 1H), 2.36-2.24 (m, 1H), 2.08-1.98 (m, 2H), 1.97 (s, 3H), 1.66-1.46 (m, 7H), 1.45-1.18 (m, 5H), 0.89 (t, $J$ = 7.2 Hz, 3H); $^{13}$C NMR (100 MHz, $CDCl_3$): δ 198.1, 170.8, 170.7, 169.1, 149.4, 125.3, 104.2, 95.0, 79.7, 63.5, 52.9, 52.8, 36.6, 34.8, 33.6, 31.2, 29.2, 25.4, 22.1, 21.6, 21.52, 21.50, 13.9; HRMS (ESI): calc. for $C_{23}H_{32}NaO_6$ $[M+Na]^+$: 427.2091; found: 427.2091.

*Dimethyl 4-(hex-1-enylidene)-3-(1-(propionyloxy)cyclohexyl)cyclopent-2-ene-1,1-dicarboxylate (**3bb**)*

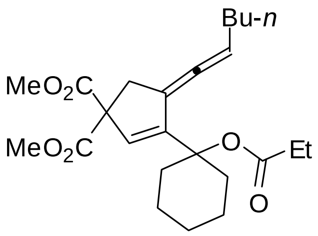


65% isolated yield, liquid. $^1$H NMR (400 MHz, $CDCl_3$): 5.73 (d, *J* = 1.6 Hz, 1H), 5.30-5.24 (m, 1H), 3.722 (s, 3H), 3.717 (s, 3H), 3.17 (d, *J* = 3.6 Hz, 2H), 2.48-2.40 (m, 1H), 2.35-2.25 (m, 1H), 2.25 (q, *J* = 7.2 Hz, 2H), 2.08-1.98 (m, 2H), 1.66-1.45 (m, 7H), 1.44-1.15 (m, 5H), 1.09 (t, *J* = 7.6 Hz, 3H), 0.88 (t, *J* = 7.2 Hz, 3H); $^{13}$C NMR (100 MHz, $CDCl_3$): δ 198.1, 172.3, 170.8, 170.7, 149.5, 125.2, 104.2, 95.0, 79.4, 63.4, 52.84, 52.81, 36.7, 34.9, 33.6, 31.2, 29.2, 28.1, 25.4, 22.1, 21.51, 21.48, 13.8, 9.2; HRMS (ESI): calc. for $C_{24}H_{34}NaO_6$ $[M+Na]^+$: 441.2248; found: 441.2251.

*Dimethyl 3-(1-(butyryloxy)cyclohexyl)-4-(hex-1-enylidene)cyclopent-2-ene-1,1-dicarboxylate (**3bc**)*

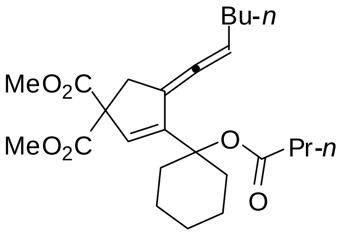


74% isolated yield, liquid. $^1$H NMR (500 MHz, $CDCl_3$): 5.73 (s, 1H), 5.30-5.24 (m, 1H), 3.72 (s, 3H), 3.71 (s, 3H), 3.17 (d, *J* = 4.0 Hz, 2H), 2.47-2.40 (m, 1H), 2.32-2.24 (m, 1H), 2.20 (t, *J* = 7.5 Hz, 2H), 2.06-1.96 (m, 2H), 1.66-1.58 (m, 4H), 1.57-1.41 (m, 5H), 1.40-1.28 (m, 4H), 1.27-1.17 (m, 1H), 0.92 (t, *J* = 7.5 Hz, 3H), 0.88 (t, *J* = 7.5 Hz, 3H); $^{13}$C NMR (125 MHz, $CDCl_3$): δ 198.1, 171.6, 170.8, 170.6, 149.4, 125.3, 104.2, 95.0, 79.4, 63.4, 52.83, 52.80, 36.8, 36.7, 34.9, 33.5, 31.2, 29.2, 25.4, 22.1, 21.50, 21.46, 18.4, 13.8, 13.7; HRMS (ESI): calc. for $C_{25}H_{36}NaO_6$ $[M+Na]^+$: 455.2404; found: 455.2405.

*Representative procedure B for the synthesis of **3**. Dimethyl 3-(1-(benzoyloxy)-cyclohexyl)-4-(hex-1-enylidene)cyclopent-2-ene-1,1-dicarboxylate (**3bd**)*

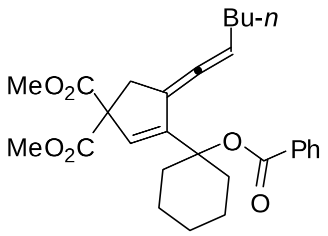


To a mixture of $PhCO_2H$ (120.0 mg, 0.98 mmol), BQ (26.1 mg, 0.24 mmol) and $Pd(OAc)_2$ (2.4 mg, 0.01 mmol) were added **1b** (70.1 mg, 0.20 mmol) and 0.4 mL of acetone at rt. The reaction was sealed and stirred at 60 $^oC$ for 17 h. After full consumption of starting material **1b**, as monitored by TLC, the reaction was cooled to room temperature, diluted with $Et_2O$ (20 mL), and washed with saturated aqueous solution of $Na_2CO_3$ (5 mL). The organic phase was separated and the aqueous phase was extracted with $Et_2O$ (2 x 20 mL). The combined organic layer was dried over *anhydrous* $Na_2SO_4$. Evaporation and column chromatography on silica gel (pentane/ethyl acetate = 10/1) afforded **3bd** (70.9 mg, 75%): liquid. $^1$H NMR (400 MHz, $CDCl_3$): 8.02-7.96 (m, 2H), 7.54-7.48 (m, 1H), 7.42-7.36 (m, 2H), 5.83 (d, $J$ = 1.2 Hz, 1H), 5.12-5.02 (m, 1H), 3.75 (s, 3H), 3.74 (s, 3H), 3.24-3.12 (m, 2H), 2.64-2.50 (m, 2H), 1.74-1.52 (m, 9H), 1.34-1.22 (m, 1H), 1.21-1.06 (m, 4H), 0.78 (t, $J$ = 7.2 Hz, 3H); $^{13}$C NMR (100 MHz, $CDCl_3$): δ 198.1, 170.78, 170.76, 164.2, 149.6, 132.4, 131.3, 129.6, 128.0, 125.1, 104.1, 95.3, 80.1, 63.5, 52.9, 52.8, 36.6, 34.6, 34.3, 31.0, 28.9, 25.4, 22.1, 21.7, 21.6, 13.7; HRMS (ESI): calc. for $C_{28}H_{34}NaO_6$ $[M+Na]^+$: 489.2248; found: 489.2249.

*Dimethyl 4-(hex-1-enylidene)-3-(1-(2-methoxybenzoyloxy)cyclohexyl)cyclopent-2-ene-1,1-dicarboxylate (**3be**)*

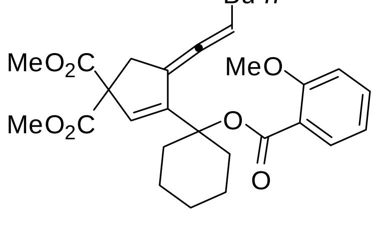


64% isolated yield, liquid. $^1$H NMR (400 MHz, $CDCl_3$): 7.83 (dd, $J_1$ = 8.0 Hz, $J_2$ = 2.0 Hz, 1H), 7.45-7.38 (m, 1H), 6.96-6.90 (m, 2H), 5.82 (d, $J$ = 1.2 Hz, 1H), 5.14-5.06 (m, 1H), 3.86 (s, 3H), 3.74 (s, 3H), 3.73 (s, 3H), 3.24-3.14 (m, 2H),

2.64-2.55 (m, 1H), 2.54-2.44 (m, 1H), 1.84-1.74 (m, 2H), 1.73-1.52 (m, 7H), 1.32-1.08 (m, 5H), 0.79 (t, *J* = 7.2 Hz, 3H); $^{13}$C NMR (100 MHz, $CDCl_3$): δ 198.1, 170.9, 170.8, 163.8, 159.4, 149.6, 133.0, 132.0, 125.0, 120.9, 119.7, 111.8, 104.2, 95.3, 80.3, 63.5, 55.7, 52.83, 52.81, 36.7, 34.8, 34.1, 31.1, 29.0, 25.5, 22.1, 21.46, 21.45, 13.8; HRMS (ESI): calc. for $C_{29}H_{36}NaO_7$ $[M+Na]^+$: 519.2353; found: 519.2360.

*Dimethyl 3-(1-(2,4-dimethoxybenzoyloxy)cyclohexyl)-4-(hex-1-enylidene)cyclopent-2-ene-1,1-dicarboxylate (**3bf**)*

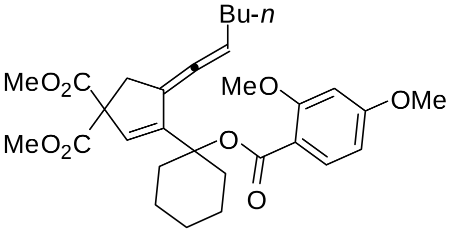


68% isolated yield, liquid. $^1$H NMR (400 MHz, $CDCl_3$): 7.87 (d, *J* = 9.2 Hz, 1H), 6.48-6.42 (m, 2H), 5.78 (d, *J* = 1.2 Hz, 1H), 5.14-5.06 (m, 1H), 3.833 (s, 3H), 3.826 (s, 3H), 3.73 (s, 3H), 3.72 (s, 3H), 3.25-3.12 (m, 2H), 2.62-2.54 (m, 1H), 2.53-2.44 (m, 1H), 1.84-1.73 (m, 2H), 1.72-1.50 (m, 7H), 1.32-1.08 (m, 5H), 0.80 (t, *J* = 7.2 Hz, 3H); $^{13}$C NMR (100 MHz, $CDCl_3$): δ 198.1, 170.9, 170.8, 163.9, 163.1, 161.6, 150.0, 134.1, 124.6, 113.1, 104.2, 104.1, 98.7, 95.1, 79.6, 63.5, 55.7, 55.4, 52.81, 52.78, 36.7, 34.8, 34.2, 31.1, 29.0, 25.5, 22.1, 21.52, 21.51, 13.8; HRMS (ESI): calc. for $C_{30}H_{38}NaO_8$ $[M+Na]^+$: 549.2459; found: 549.2458.

*Dimethyl 3-(1-(2-fluorobenzoyloxy)cyclohexyl)-4-(hex-1-enylidene)cyclopent-2-ene-1,1-dicarboxylate (**3bg**)*

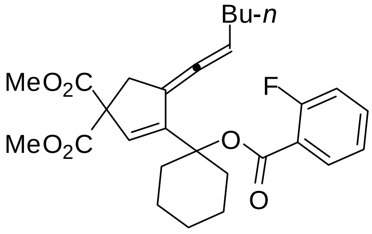


75% isolated yield, liquid. $^1$H NMR (400 MHz, $CDCl_3$): 7.94-7.88 (m, 1H), 7.50-7.40 (m, 1H), 7.19-7.12 (m, 1H), 7.10-7.06 (m, 1H), 5.83 (d, *J* = 1.2 Hz, 1H), 5.12-5.02 (m,

1H), 3.75 (s, 3H), 3.74 (s, 3H), 3.24-3.14 (m, 2H), 2.64-2.54 (m, 1H), 2.53-2.44 (m, 1H), 1.80-1.50 (m, 9H), 1.42-1.02 (m, 5H), 0.78 (t, $J$ = 7.2 Hz, 3H); $^{13}$C NMR (100 MHz, $CDCl_3$): δ 198.0, 170.8, 170.7, 162.13, 162.09, 162.0 (d, $J$ = 259.9 Hz), 149.3, 133.9 (d, $J$ = 8.9 Hz), 132.3, 125.2, 123.6 (d, $J$ = 4.0 Hz), 116.8 (d, $J$ = 22.7 Hz), 104.1, 95.3, 81.1, 63.5, 52.9, 52.8, 36.7, 34.6, 34.2, 31.0, 28.9, 25.4, 22.1, 21.45, 21.43, 13.7; $^{19}$F NMR (376 MHz, $CDCl_3$): -108.6; HRMS (ESI): calc. for $C_{28}H_{33}FNaO_6$ $[M+Na]^+$: 507.2153; found: 507.2153.

*Dimethyl 3-(1-(2-chlorobenzoyloxy)cyclohexyl)-4-(hex-1-enylidene)cyclopent-2-ene-1,1-dicarboxylate (**3bh**)*

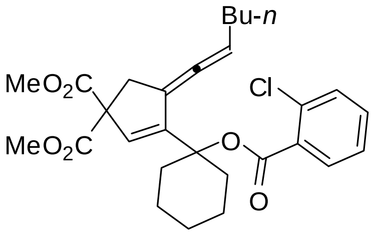


73% isolated yield, liquid. $^1$H NMR (500 MHz, $CDCl_3$): 7.86 (dd, $J_1$ = 8.0 Hz, $J_2$ = 1.5 Hz, 1H), 7.44-7.35 (m, 2H), 7.31-7.26 (m, 1H), 5.88 (s, 1H), 5.14-5.04 (m, 1H), 3.760 (s, 3H), 3.755 (s, 3H), 3.24-3.16 (m, 2H), 2.68-2.60 (m, 1H), 2.54-2.44 (m, 1H), 1.78-1.52 (m, 9H), 1.45-1.12 (m, 5H), 0.80 (t, $J$ = 7.2 Hz, 3H); $^{13}$C NMR (125 MHz, $CDCl_3$): δ 198.0, 170.8, 170.6, 163.3, 148.8, 133.6, 132.1, 131.7, 130.99, 130.96, 126.3, 125.8, 104.1, 95.5, 81.7, 63.5, 52.89, 52.86, 36.8, 34.7, 33.8, 31.1, 28.9, 25.4, 22.0, 21.6, 21.5, 13.8; HRMS (ESI): calc. for $C_{28}H_{33}ClNaO_6$ $[M+Na]^+$: 523.1858; found: 523.1858.

*Dimethyl 3-(1-(2,4-dichlorobenzoyloxy)cyclohexyl)-4-(hex-1-enylidene)cyclopent-2-ene-1,1-dicarboxylate (**3bi**)*

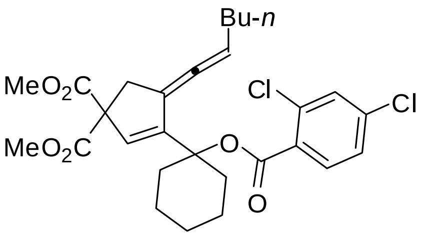


84% isolated yield, liquid. $^1$H NMR (500 MHz, $CDCl_3$): 7.81 (d, $J$ = 8.5 Hz, 1H), 7.44

(d, $J$ = 2.0 Hz, 1H), 7.26 (dd, $J_1$ = 8.5 Hz, $J_2$ = 2.0 Hz, 1H), 5.86 (d, $J$ = 1.0 Hz, 1H), 5.14-5.04 (m, 1H), 3.75 (s, 3H), 3.74 (s, 3H), 3.21-3.17 (m, 2H), 2.62-2.56 (m, 1H), 2.50-2.45 (m, 1H), 1.82-1.52 (m, 9H), 1.42-1.12 (m, 5H), 0.81 (t, $J$ = 7.0 Hz, 3H); $^{13}$C NMR (125 MHz, $CDCl_3$): δ 197.9, 170.7, 170.6, 162.4, 148.6, 137.8, 134.7, 132.8, 130.9, 129.2, 126.7, 126.0, 104.1, 95.5, 82.0, 63.5, 52.90, 52.88, 36.7, 34.7, 33.8, 31.1, 29.0, 25.3, 22.1, 21.58, 21.56, 13.8; HRMS (ESI): calc. for $C_{28}H_{32}Cl_2NaO_6$ $[M+Na]^+$: 557.1468; found: 557.1472.

*Dimethyl 3-(2-acetoxybutan-2-yl)-4-(hex-1-enylidene)cyclopent-2-ene-1,1-dicarboxylate* ***(3ca)***

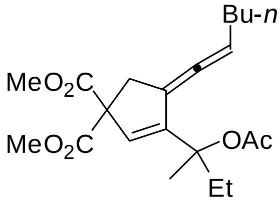


66% isolated yield, liquid. $^1$H NMR (400 MHz, $CDCl_3$): 5.81-5.77 (m, 1H), 5.39-5.28 (m, 1H), 3.74-3.73 (m, 6H), 3.24-3.14 (m, 2H), 2.14-1.96 (m, 6H), 1.94-1.80 (m, 1H), 1.64-1.58 (m, 3H), 1.48-1.22 (m, 4H), 0.92-0.81 (m, 3H), 0.80-0.72 (m, 3H); $^{13}$C NMR (100 MHz, $CDCl_3$): δ 198.1, 198.0, 170.9, 170.82, 170.76, 170.68, 169.34, 169.31, 148.3, 126.4, 104.39, 104.36, 95.8, 95.5, 82.9, 82.6, 63.33, 63.26, 52.9, 52.86, 52.84, 52.83, 36.9, 36.8, 31.04, 31.01, 30.8, 30.1, 29.1, 29.0, 23.4, 23.0, 22.2, 22.0, 21.9, 13.8, 7.82, 7.78; HRMS (ESI): calc. for $C_{21}H_{30}NaO_6$ $[M+Na]^+$: 401.1935; found: 401.1934.

*Dimethyl 3-(2-acetoxypropan-2-yl)-4-(prop-1-enylidene)cyclopent-2-ene-1,1-dicarboxylate* ***(3da)***

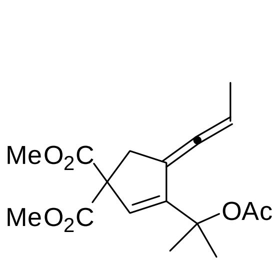


52% isolated yield, liquid. $^1$H NMR (500 MHz, $CDCl_3$): 5.78 (d, $J$ = 1.5 Hz, 1H), 5.38-5.31 (m, 1H), 3.74 (s, 3H), 3.73 (s, 3H), 3.22 (qd, $J_1$ = 16.0 Hz, $J_2$ = 4.0 Hz, 2H),

1.96 (s, 3H), 1.69 (d, $J$ = 7.0 Hz, 3H), 1.60 (s, 3H), 1.59 (s, 3H); $^{13}$C NMR (125 MHz, $CDCl_3$): δ 199.2, 170.8, 170.7, 169.4, 149.7, 125.1, 103.9, 90.1, 79.1, 63.3, 52.93, 52.91, 36.4, 26.3, 26.1, 22.0, 14.7; HRMS (ESI): calc. for $C_{17}H_{22}NaO_6$ $[M+Na]^+$: 345.1309; found: 345.1301.

*Dimethyl 3-(2-acetoxypropan-2-yl)-4-vinylidenecyclopent-2-ene-1,1-dicarboxylate* ***(3ea)***

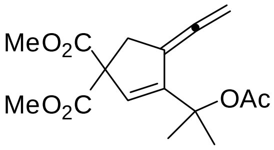


39% isolated yield, liquid. $^1$H NMR (400 MHz, $CDCl_3$): 5.77 (s, 1H), 4.98 (td, $J_1$ = 4.0 Hz, $J_2$ = 1.6 Hz, 2H), 3.74 (s, 6H), 3.26 (t, $J$ = 4.0 Hz, 2H), 1.96 (s, 3H), 1.61 (s, 6H); $^{13}$C NMR (100 MHz, $CDCl_3$): δ 203.2, 170.5, 169.4, 149.4, 125.2, 104.1, 79.3, 78.9, 63.6, 53.0, 36.3, 26.3, 21.8; HRMS (ESI): calc. for $C_{16}H_{20}NaO_6$ $[M+Na]^+$: 331.1152; found: 331.1151.

*2-(5-(Hex-1-enylidene)-3,3-bis(hydroxymethyl)cyclopent-1-enyl)propan-2-yl acetate* ***(3ga)***

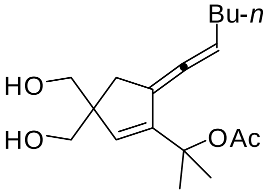


60% isolated yield, liquid. $^1$H NMR (500 MHz, $CDCl_3$): 5.59 (d, $J$ = 1.5 Hz, 1H), 5.28-5.21 (m, 1H), 3.64-3.51 (m, 4H), 2.77 (bs, 1H), 2.70 (bs, 1H), 2.58-2.46 (m, 2H), 2.04-1.96 (m, 2H), 1.94 (s, 3H), 1.59 (s, 3H), 1.54 (s, 3H), 1.41-1.30 (m, 4H), 0.89 (t, $J$ = 7.0 Hz, 3H); $^{13}$C NMR (125 MHz, $CDCl_3$): δ 198.5, 170.4, 148.5, 130.5, 105.7, 94.2, 79.1, 67.71, 67.66, 54.2, 35.8, 31.3, 29.3, 27.0, 26.3, 22.2, 22.0, 13.9; HRMS (ESI): calc. for $C_{18}H_{28}NaO_4$ $[M+Na]^+$: 331.1880; found: 331.1874.

*2-(3,3-Bis(benzyloxymethyl)-5-(hex-1-enylidene)cyclopent-1-enyl)propan-2-yl acetate* ***(3ha)***

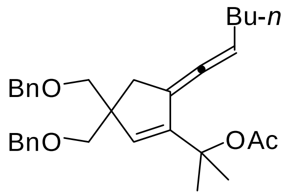

70% isolated yield, liquid. $^{1}H$ NMR (500 MHz, $CDCl_3$): 7.40-7.20 (m, 10H), 5.72 (s, 1H), 5.30-5.22 (m, 1H), 4.58-4.51 (m, 4H), 3.54-3.44 (m, 4H), 2.58-2.50 (m, 2H), 2.10-2.01 (m, 2H), 1.99 (s, 3H), 1.63 (s, 3H), 1.59 (s, 3H), 1.48-1.32 (m, 4H), 0.93 (t, $J$ = 7.0 Hz, 3H); $^{13}C$ NMR (125 MHz, $CDCl_3$): δ 198.5, 169.6, 147.1, 138.8, 131.6, 128.2, 127.33, 127.30, 105.7, 93.9, 79.5, 73.31, 73.27, 73.2, 52.1, 36.9, 31.3, 29.4, 26.9, 26.2, 22.2, 22.1, 13.9; HRMS (ESI): calc. for $C_{32}H_{40}NaO_4$ $[M+Na]^+$: 511.2819; found: 511.2825.

*2-(3-(Prop-1-enylidene)-3,6-dihydro-2H-pyran-4-yl)propan-2-yl acetate (**3ia**)*

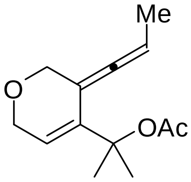

51% isolated yield, liquid. $^{1}H$ NMR (500 MHz, $CDCl_3$): 5.72 (td, $J_1$ = 3.0 Hz, $J_1$ = 1.5 Hz, 1H), 5.28 (qd, $J_1$ = 3.0 Hz, $J_1$ = 1.5 Hz, 1H), 4.30 (d, $J$ = 3.0 Hz, 1H), 4.29 (d, $J$ = 3.0 Hz, 1H), 4.22 (dd, $J_1$ = 12.0 Hz, $J_1$ = 2.0 Hz, 1H), 4.18 (dd, $J_1$ = 12.0 Hz, $J_1$ = 2.0 Hz, 1H), 1.96 (s, 3H), 1.71 (d, $J$ = 7.0 Hz, 3H), 1.61 (s, 3H), 1.57 (s, 3H); $^{13}C$ NMR (125 MHz, $CDCl_3$): δ 201.8, 169.7, 136.6, 121.4, 94.4, 88.0, 81.1, 68.5, 66.1, 27.0, 26.5, 22.1, 14.4; HRMS (ESI): calc. for $C_{13}H_{18}NaO_3$ $[M+Na]^+$: 245.1148; found: 245.1151.

*2-(3-(Hex-1-enylidene)-3,6-dihydro-2H-pyran-4-yl)propan-2-yl acetate (**3ja**)*

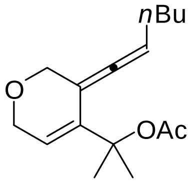

52% isolated yield, liquid. $^{1}H$ NMR (500 MHz, $CDCl_3$): 5.71 (td, $J_1$ = 3.0 Hz, $J_1$ = 1.5 Hz, 1H), 5.29-5.22 (m, 1H), 4.30 (d, $J$ = 3.0 Hz, 1H), 4.29 (d, $J$ = 3.0 Hz, 1H), 4.22

(dd, $J_1$ = 12.0 Hz, $J_1$ = 2.0 Hz, 1H), 4.18 (dd, $J_1$ = 12.0 Hz, $J_1$ = 2.0 Hz, 1H), 2.08 (q, $J$ = 7.0 Hz, 2H), 1.95 (s, 3H), 1.62 (s, 3H), 1.57 (s, 3H), 1.48-1.39 (m, 2H), 1.38-1.31 (m, 2H), 0.90 (t, $J$ = 7.0 Hz, 3H); $^{13}$C NMR (125 MHz, $CDCl_3$): δ 200.8, 169.6, 136.6, 121.2, 94.6, 93.4, 81.1, 68.7, 66.1, 31.4, 28.9, 27.2, 26.3, 22.2, 22.1, 13.9; HRMS (ESI): calc. for $C_{16}H_{24}NaO_3$ $[M+Na]^+$: 287.1618; found: 287.1615.

**The reaction of phenyl-substituted allenyne 1f.**

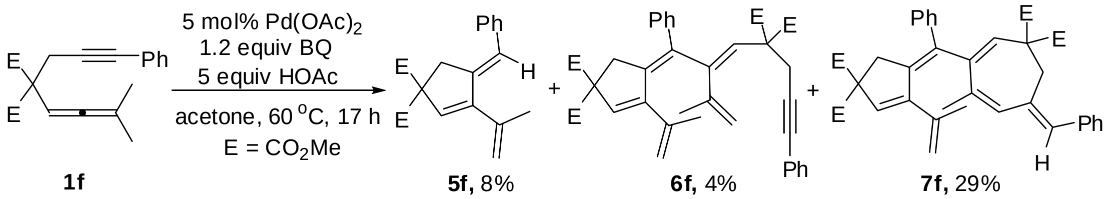


To a mixture of BQ (52.1 mg, 0.48 mmol), $Pd(OAc)_2$ (4.6 mg, 0.02 mmol), and HOAc (120.2 mg, 2.0 mmol) were added **1f** (125.2 mg, 0.40 mmol) and 0.8 mL of acetone at rt. The reaction was sealed and stirred at 60 °C for 17 h. After full consumption of starting material **1f**, as monitored by TLC, the reaction was cooled to room temperature, diluted with $Et_2O$ (20 mL), and quenched with 5 mL of $H_2O$. The organic phase was separated and the aqueous phase was extracted with $Et_2O$ (2 x 20 mL). The combined organic layers were washed with $H_2O$ and dried over *anhydrous* $Na_2SO_4$. Evaporation and column chromatography on silica gel (pentane/ethyl acetate = 30/1, then 10/1) afforded **5f** (9.4 mg, 8%) and (**6f**+**7f**) (41.3 mg, 4% yield for **6f** and 29% yield for **7f**, respectively).

*(E)-dimethyl 4-benzylidene-3-(prop-1-en-2-yl)cyclopent-2-ene-1,1-dicarboxylate (**5f**)*

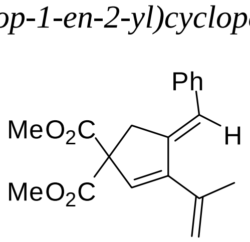


Liquid. $^1$H NMR (500 MHz, $CDCl_3$): δ 7.45-7.27 (m, 4H), 7.27-7.15 (m, 1H), 6.55 (s, 1H), 6.04 (s, 1H), 5.25 (s, 1H), 5.17 (s, 1H), 3.76 (s, 6H), 3.53 (s, 2H), 2.01 (s, 3H); $^{13}$C NMR (125 MHz, $CDCl_3$): δ 171.0, 151.5, 142.0, 138.0, 137.6, 128.8, 128.6, 128.5, 126.8, 123.0, 116.8, 64.4, 53.1, 38.2, 23.2; HRMS (ESI): calc. for $C_{19}H_{20}NaO_4$

$[M+Na]^+$: 335.1254; found: 335.1246.

*(2E,4Z,6E)-dimethyl 6-benzylidene-3-((Z)-(4,4-bis(methoxycarbonyl)-2-(prop-1-en-2-yl)cyclopent-2-enylidene)(phenyl)methyl)-4-methylcyclohepta-2,4-diene-1,1-dicarboxylate* ***(7f)***

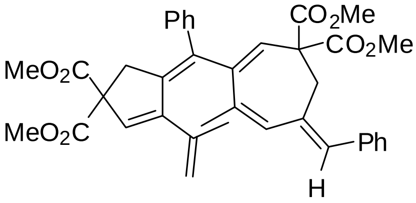


Liquid. $^{1}$H NMR (500 MHz, $CDCl_3$): δ 7.45-7.25 (m, 10H), 6.33 (s, 1H), 6.22 (d, *J* = 1.5 Hz, 1H), 6.12 (s, 1H), 6.05 (s, 1H), 4.99 (d, *J* = 1.0 Hz, 1H), 4.86 (s, 1H), 3.81 (s, 6H), 3.67 (s, 6H), 3.45 (d, *J* = 2.0 Hz, 2H), 3.22 (s, 2H), 2.07 (d, *J* = 1.5 Hz, 3H), 1.82 (s, 3H); $^{13}$C NMR (125 MHz, $CDCl_3$): δ 170.6, 170.1, 149.8, 143.3, 142.0, 141.0, 137.6, 135.8, 131.8, 131.7, 130.7, 128.51, 128.49, 128.2, 127.9, 126.7, 125.5, 123.7, 123.4, 123.1, 115.8, 84.7, 83.5, 64.8, 58.4, 53.1, 52.9, 37.5, 27.3, 24.0, 21.0; HRMS (ESI): calc. for $C_{38}H_{38}NaO_8$ $[M+Na]^+$: 645.2459; found: 645.2481.

### *General procedure for aerobic acetoxylation/carbocyclization*

Oxidation processes where the terminal oxidant is molecular oxygen have attracted considerable attention in recent years. To further enhance the synthetic utility of the oxidative carbocyclization in Table 2 and Scheme 3, the reaction was studied under various aerobic conditions. Gratifyingly, it was found that the combination of cocatalyst [Co]-salophen with molecular oxygen (balloon) in the presence of catalytic amounts of BQ (20 mol%) permits the efficient reoxidation of Pd(0) to Pd(II) and makes it possible to use $O_2$ as the oxidant in the acetoxylation/carbocyclization of allenynes (Scheme S1).

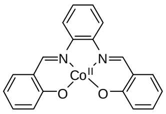

[Co]-salophen

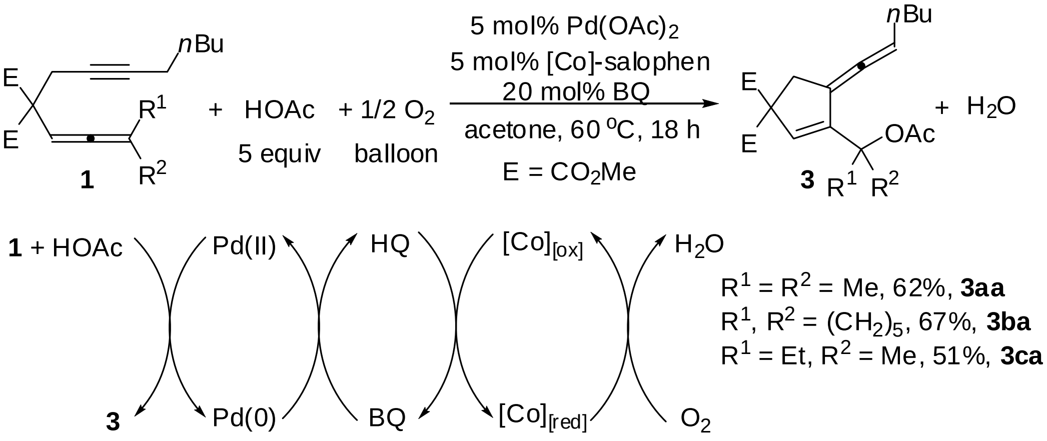


***Scheme S1.*** Aerobic acetoxylation/carbocyclization of allenynes **1**.

*Representative procedure C for the synthesis of **3**. Dimethyl 3-(2-acetoxypropan-2-yl)-4-(hex-1-enylidene)cyclopent-2-ene-1,1-dicarboxylate (**3aa**)*

To a mixture of BQ (4.2 mg, 0.04 mmol), [Co]-salophen (3.9 mg, 0.01 mmol), $Pd(OAc)_2$ (2.1 mg, 0.01 mmol), and HOAc (60.2 mg, 1.0 mmol) were added **1a** (61.9 mg, 0.20 mmol) and 0.4 mL of acetone at rt. The reaction was stirred at 60 °C under an oxygen atmosphere (balloon) for 18 h. Then the reaction was cooled to room temperature, diluted with $Et_2O$ (20 mL), and quenched with 5 mL of $H_2O$. The organic phase was separated and the aqueous phase was extracted with $Et_2O$ (2 x 20 mL). The combined organic layers were washed with $H_2O$ and dried over *anhydrous* $Na_2SO_4$. Evaporation and column chromatography on silica gel (pentane/ethyl acetate = 8/1) afforded **3aa** (45.1 mg, 62%).

**Application of product 3aa.**

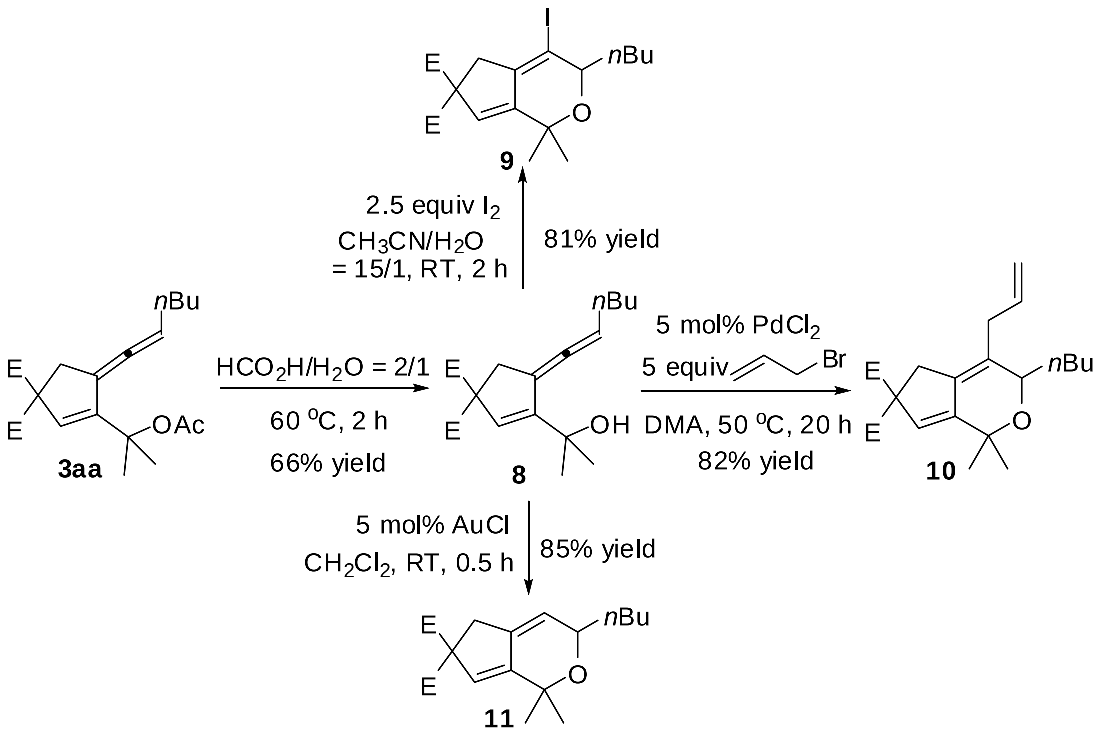


*Dimethyl 4-(hex-1-enylidene)-3-(2-hydroxypropan-2-yl)cyclopent-2-ene-1,1-dicarboxylate (**8**)*[8]

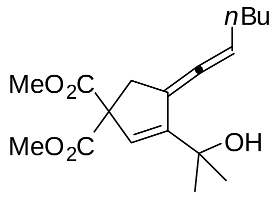


A solution of **3aa** (38.6 mg, 0.11 mmol) and $H_2O$ (0.1 mL) in formic acid (0.2 mL, 88%) was stirred at 60 °C for 2 h. When the reaction was complete as monitored by TLC, the mixture was diluted with $Et_2O$ (20 mL) and washed with saturated aqueous solution of $Na_2CO_3$ (5 mL). The organic layer was dried over $Na_2SO_4$. Concentration and column chromatography on silica gel (pentane/ethyl acetate = 3:1) afforded **8** (22.5 mg, 66%): liquid; $^1$H NMR (500 MHz, $CDCl_3$): 5.84 (s, 1H), 5.50-5.41 (m, 1H), 3.75 (s, 3H), 3.74 (s, 3H), 3.30-3.20 (m, 2H), 2.14 (s, 1H), 2.13-2.02 (m, 2H), 1.43 (s, 6H), 1.42-1.30 (m, 4H), 0.90 (t, $J$ = 7.0 Hz, 3H); $^{13}$C NMR (125 MHz, $CDCl_3$): δ 198.0, 170.9, 153.1, 124.4, 105.2, 96.7, 71.0, 63.3, 52.94, 52.93, 36.8, 31.0, 29.0, 28.93, 28.86, 22.2, 13.9; HRMS (ESI): calc. for $C_{18}H_{26}NaO_5$ $[M+Na]^+$: 345.1672;

found: 345.1676.

*Dimethyl 3-butyl-4-iodo-1,1-dimethyl-3,5-dihydrocyclopenta[c]pyran-6,6(1H)-dicarboxylate (**9**)*[9]

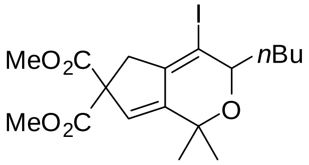


A solution of **8** (33.0 mg, 0.10 mmol) and $I_2$ (64.0 mg, 0.25 mmol, 2.5 equiv) in $CH_3CN$ (1 mL) and $H_2O$ (67 μL) was stirred at rt for 2 h. When the reaction was complete as monitored by TLC, $H_2O$ (5 mL) was added together with a saturated aqueous solution of $Na_2S_2O_3$ (2 mL) to remove the excess $I_2$. This mixture was extracted with ether (3 x 20 mL). The combined organic layers were washed with $H_2O$ (10 mL) and dried over $Na_2SO_4$. Concentration and column chromatography on silica gel (pentane/ethyl acetate = 20:1) afforded **9** (37.0 mg, 81%): liquid; $^1H$ NMR (500 MHz, $CDCl_3$): 5.87 (s, 1H), 4.18-4.14 (m, 1H), 3.75 (s, 6H), 3.17 (dd, $J_1$ = 17.5 Hz, $J_2$ = 2.5 Hz, 1H), 3.07 (dd, $J_1$ = 17.5 Hz, $J_2$ = 2.5 Hz, 1H), 1.98-1.88 (m, 1H), 1.68-1.60 (m, 1H), 1.41 (s, 3H), 1.40-1.29 (m, 4H), 1.28 (s, 3H), 0.90 (t, $J$ = 7.0 Hz, 3H); $^{13}C$ NMR (125 MHz, $CDCl_3$): δ 170.8, 170.4, 149.4, 145.4, 123.8, 95.3, 72.6, 71.9, 62.2, 53.1, 53.0, 42.6, 36.1, 27.0, 26.6, 25.5, 22.5, 14.1; HRMS (ESI): calc. for $C_{18}H_{25}INaO_5$ $[M+Na]^+$: 471.0639; found: 471.0640.

*Dimethyl 4-allyl-3-butyl-1,1-dimethyl-3,5-dihydrocyclopenta[c]pyran-6,6(1H)-dicarboxylate (**10**)*[10]

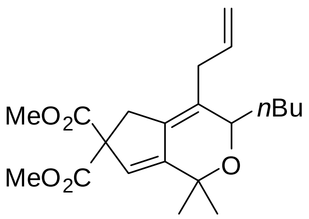


A solution of **8** (33.2 mg, 0.10 mmol), allyl bromide (60.9 mg, 0.50 mmol, 5 equiv), and $PdCl_2$ (1.0 mg, 0.005 mmol) in DMA (0.5 mL) was stirred at 50 °C for 20 h.

When the reaction was complete as monitored by TLC, the mixture was purified by column chromatography on silica gel (pentane/ethyl acetate = 20:1) directly to afford **10** (30.7 mg, 82%): liquid; $^1$H NMR (500 MHz, $CDCl_3$): 5.74-5.64 (m, 1H), 5.61 (s, 1H), 5.06 (dd, $J_1$ = 17.0 Hz, $J_2$ = 1.5 Hz, 1H), 5.01 (dd, $J_1$ = 10.0 Hz, $J_2$ = 1.5 Hz, 1H), 4.16-4.11 (m, 1H), 3.73 (s, 3H), 3.72 (s, 3H), 3.19 (dd, $J_1$ = 17.0 Hz, $J_2$ = 2.0 Hz, 1H), 3.07 (dd, $J_1$ = 17.0 Hz, $J_2$ = 2.5 Hz, 1H), 2.90-2.76 (m, 2H), 1.74-1.66 (m, 1H), 1.50-1.42 (m, 1H), 1.41 (s, 3H), 1.38-1.25 (m, 4H), 1.24 (s, 3H), 0.88 (t, $J$ = 7.0 Hz, 3H); $^{13}$C NMR (125 MHz, $CDCl_3$): δ 171.5, 171.1, 150.1, 134.6, 134.5, 129.5, 120.4, 115.9, 71.2, 70.5, 64.2, 52.9, 52.8, 35.2, 34.0, 32.2, 27.4, 27.3, 25.7, 22.7, 14.1; HRMS (ESI): calc. for $C_{21}H_{30}NaO_5$ $[M+Na]^+$: 385.1985; found: 385.1982.

*Dimethyl 3-butyl-1,1-dimethyl-3,5-dihydrocyclopenta[c]pyran-6,6(1H)-dicarboxylate* ***(11)***[11]

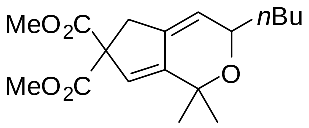


A solution of **8** (30.0 mg, 0.09 mmol) and AuCl (1.3 mg, 0.006 mmol) in $CH_2Cl_2$ (0.5 mL) was stirred at rt for 0.5 h. When the reaction was complete as monitored by TLC, the mixture was purified by column chromatography on silica gel (pentane/ethyl acetate = 15:1) directly to afford **11** (25.5 mg, 85%): liquid; $^1$H NMR (500 MHz, $CDCl_3$): 5.69 (s, 1H), 5.56 (s, 1H), 4.16-4.08 (m, 1H), 3.73 (s, 6H), 3.22-3.16 (m, 1H), 3.13-3.07 (m, 1H), 1.56-1.45 (m, 2H), 1.43 (s, 3H), 1.38-1.28 (m, 4H), 1.27 (s, 3H), 0.90 (t, $J$ = 7.0 Hz, 3H); $^{13}$C NMR (125 MHz, $CDCl_3$): δ 171.3, 171.0, 149.6, 138.2, 121.9, 120.8, 71.8, 68.7, 64.0, 52.9, 36.0, 35.4, 27.4, 27.3, 25.4, 22.7, 14.0; HRMS (ESI): calc. for $C_{18}H_{26}NaO_5$ $[M+Na]^+$: 345.1672; found: 345.1671.

## Kinetic Isotope Effect (KIE) Experiments

### 1. Determination of Intermolecular Competition KIE

BQ (26.0 mg, 0.24 mmol), $Pd(OAc)_2$ (2.3 mg, 0.01 mmol), **1a** (30.6 mg, 0.10 mmol), 0.2 mL of HOAc, [$D_2$]-**1a** (30.8 mg, 0.10 mmol), and 0.2 mL of HOAc were introduced sequentially in a vial at rt. The mixture was stirred at 60 °C for 78 min. The reaction was stopped, diluted with $Et_2O$ (25 mL), and quenched with saturated aqueous solution of $Na_2CO_3$ (5 mL). The organic phase was separated, washed with $H_2O$, and concentrated under reduced pressure. The yields and the ratio of **3aa** and [$D_1$]-**3aa** were analyzed by $^1$H NMR measurement using anisole as the internal standard (22 μL, 0.2 mmol).

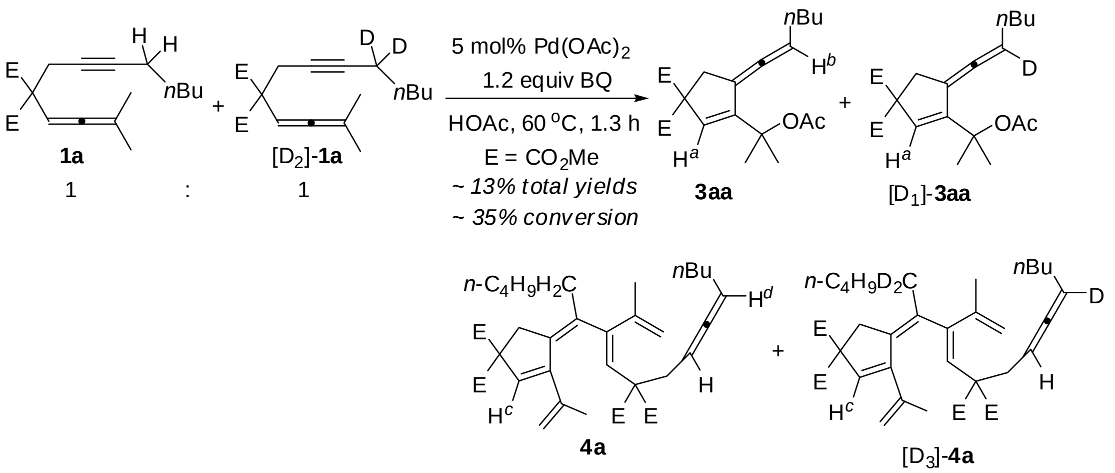


**Table S1.**

| | 1st | 2nd | 3rd | 4th | average |
|---|---|---|---|---|---|
| Integration of $H^a$ ($IntH^a$) | 1 | 1 | 1 | 1 | - |
| Integration of ($H^b$+$H^d$) ($H^b$ and $H^d$ overlap) | 0.86 | 0.89 | 0.90 | 0.87 | - |
| Integration of $H^c$ ($IntH^c$) | 0.04 | 0.06 | 0.06 | 0.05 | - |
| **3aa**/([$D_1$]-**3aa**) = $IntH^b/(IntH^a–IntH^b)$ | 4.56 | 4.88 | 5.25 | 4.56 | 4.81 |

*See the attached spectra.

**3aa**/([$D_1$]-**3aa**) = $IntH^b/(IntH^a–IntH^b) = (IntH^{b+d} - IntH^c)/[1-(IntH^{b+d} - IntH^c)]$

*Assuming Integration of $H^c$ = Integration of $H^d$.

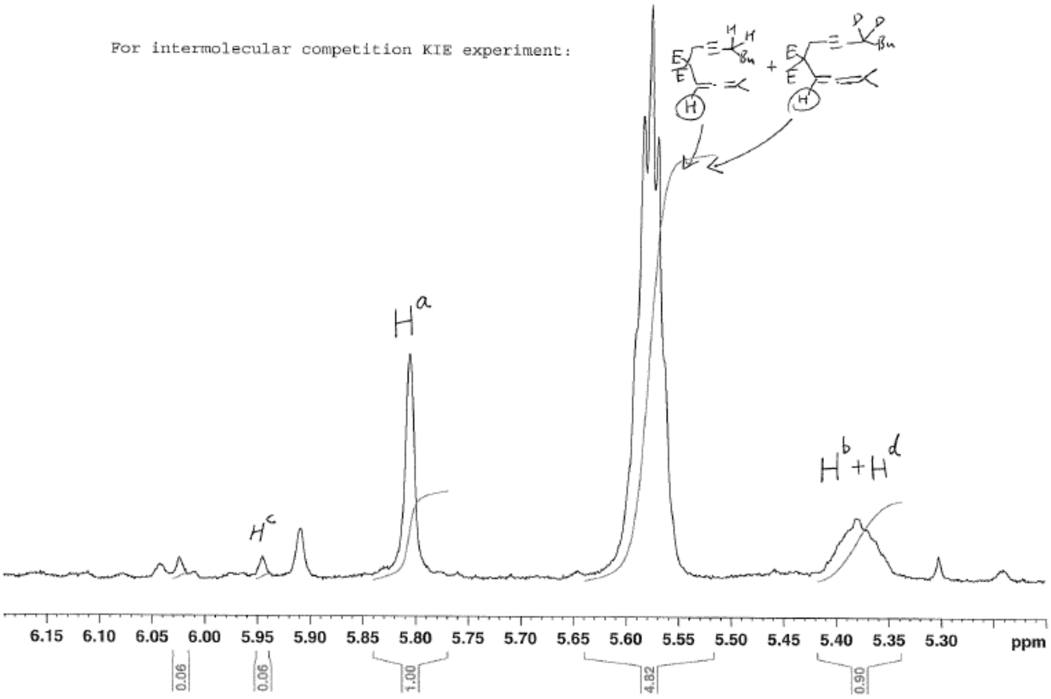


The ratio of **3aa**/([D$_1$]-**3aa**) measured was 4.8/1. However, since the ratio of the starting material continuously changes during the reaction, the isotope effect is obtained after a slight correction of this factor. The ratio (**1a**/[D$_2$]-**1a**) in the end of the reaction was estimated to be 1:1.30. The yield of **3aa**+[D$_1$]-**3aa** was 13% at a 35% conversion. Here, we assume the 22% (35%-13%) of the starting materials were converted to by-products without any isotope effect according to the proposed mechanism. Then the remaining **1a** should be 50%-(22%/2)-10.76% = 28.24% and the remaining [D$_2$]-**1a** should be 50%-(22%/2)-2.24% = 36.76%, respectively. Therefore, the isotope effect calculated from the product ratio and the change of the starting material ratio is approximately 5.5 (namely, 4.8*[(1+1.30)/2]).

## 2. Determination of Intramolecular KIE

BQ (26.0 mg, 0.24 mmol), $Pd(OAc)_2$ (2.3 mg, 0.01 mmol), [$D_1$]-**1a** (61.4 mg, 0.20 mmol), and 0.4 mL of HOAc were introduced sequentially in a vial at rt. The mixture was stirred at 60 °C for 17 h. The reactions was stopped, diluted with $Et_2O$ (50 mL), and quenched with saturated aqueous solution of $Na_2CO_3$ (15 mL). The organic phase was separated, washed with $H_2O$, dried ($Na_2SO_4$), and concentrated under reduced pressure. The yields and ratios were analyzed by $^1H$ NMR measurement using anisole as the internal standard (22 μL, 0.2 mmol).

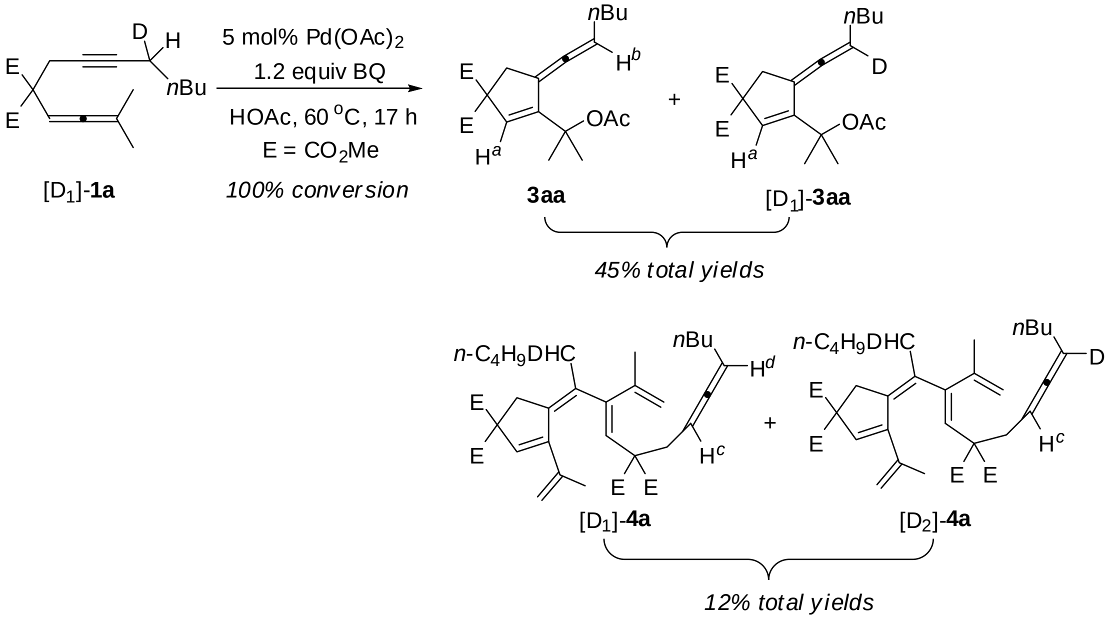


**Table S2.**

| | 1st | 2nd | 3rd | average |
|---|---|---|---|---|
| Integration of ($H^b$+$H^d$) ($H^b$ and $H^d$ overlap) | 1 | 1 | 1 | - |
| Integration of $H^a$ ($IntH^a$) | 6.16 | 6.19 | 6.06 | - |
| Integration of $H^c$ ($IntH^c$) | 0.84 | 0.85 | 0.87 | - |
| KIE = ([$D_1$]-**3aa**)/**3aa** = ($IntH^a$–$IntH^b$)/$IntH^b$ | 6.14 | 6.18 | 6.08 | 6.13 |

*See the attached spectra.

Calculation of $IntH^b$ = 1- Integration of $H^d$ = 1- $IntH^c$/KIE

KIE = ($IntH^a$–$IntH^b$)/$IntH^b$ = [$IntH^a$-(1- $IntH^c$/KIE)]/(1- $IntH^c$/KIE),

→ $IntH^a$ + $IntH^c$ - 1 = KIE - $IntH^c$/ KIE,

→ $\mathrm{KIE} = (\mathrm{IntH}^{a} + \mathrm{IntH}^{c} - 1)/2 + [\mathrm{IntH}^{c} + (\mathrm{IntH}^{a} + \mathrm{IntH}^{c} - 1)^{2}/4]^{1/2}$

*Assuming **3aa** and **4a** have a same KIE.

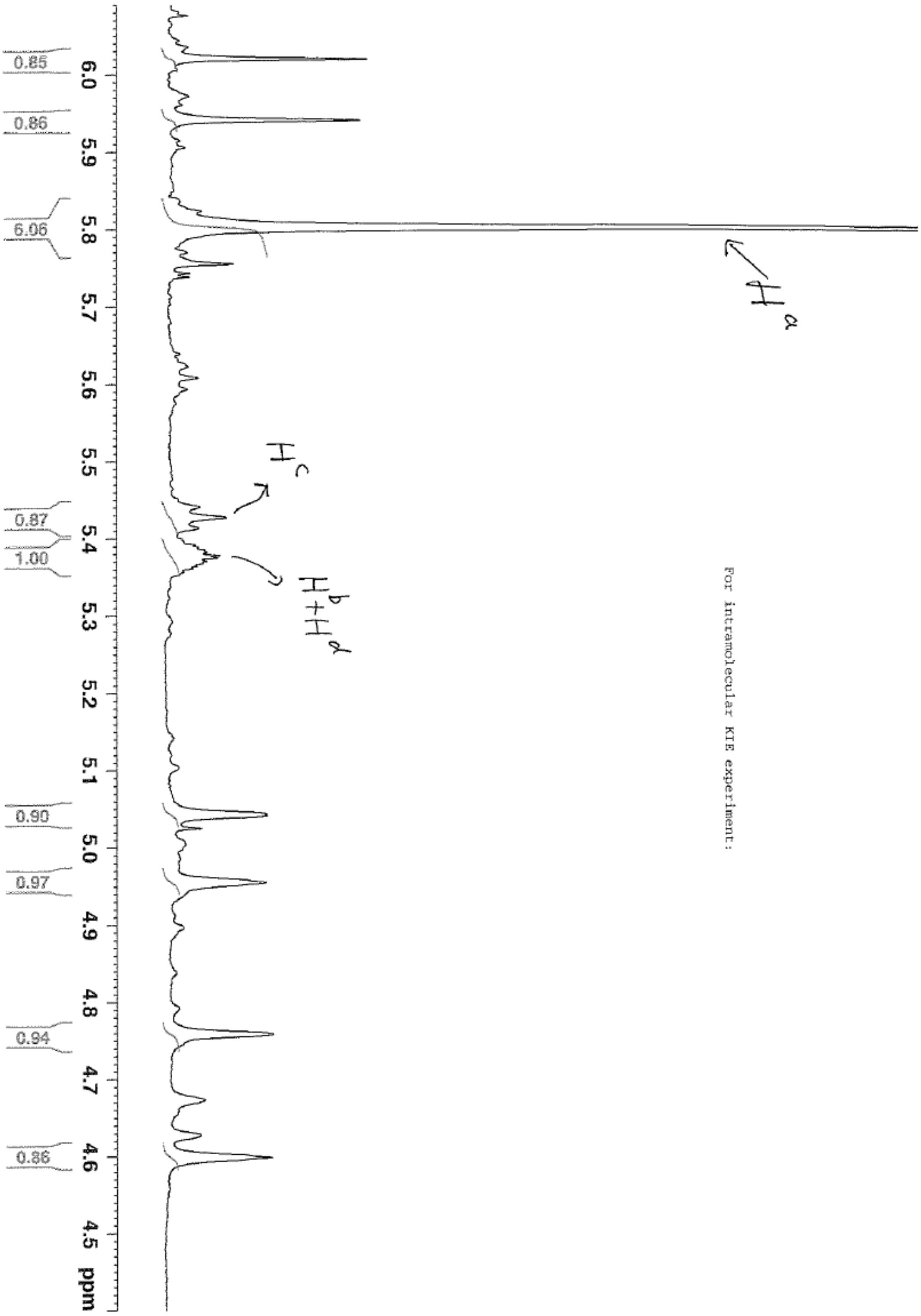

### 3. Intermolecular KIE Experiments (Separate experiments)

Side by side reactions were set-up following general procedure A (See page S10). BQ (13.0 mg, 0.12 mmol), $Pd(OAc)_2$ (1.2 mg, 0.005 mmol), **1a** (30.6 mg, 0.10 mmol) or [$D_2$]-**1a** (30.8 mg, 0.10 mmol), and 0.2 mL of HOAc were introduced in each vial at rt. The vials were stirred at 60 $^{o}$C. The reactions were stopped at the points of 30 min, 40 min, 50 min, and 60 min for substrate **1a** and 40 min, 50 min, 60 min, 120 min, 150 min, and 180 min for substrate [$D_2$]-**1a**, respectively (yield < 10%), diluted with $Et_2O$ (25 mL), and quenched with saturated aqueous solution of $Na_2CO_3$ (5 mL). The organic phase was separated, washed with $H_2O$, and concentrated under reduced pressure. The yields were analyzed by $^1$H NMR measurement using anisole as the internal standard (5.5 µL, 0.05 mmol, for each sample).

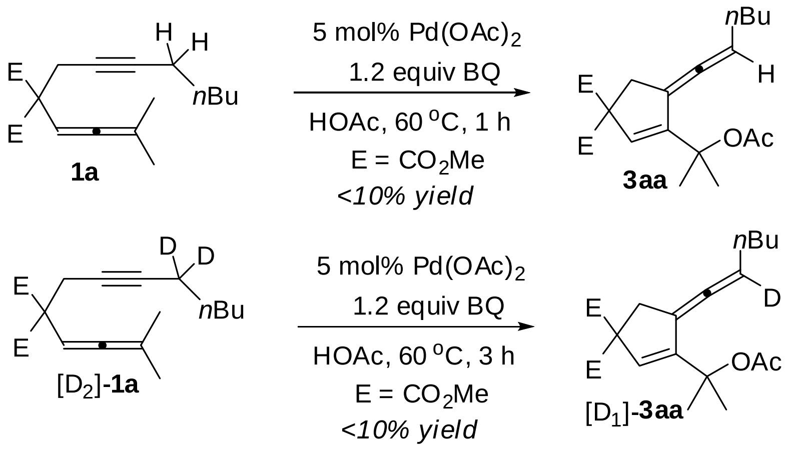


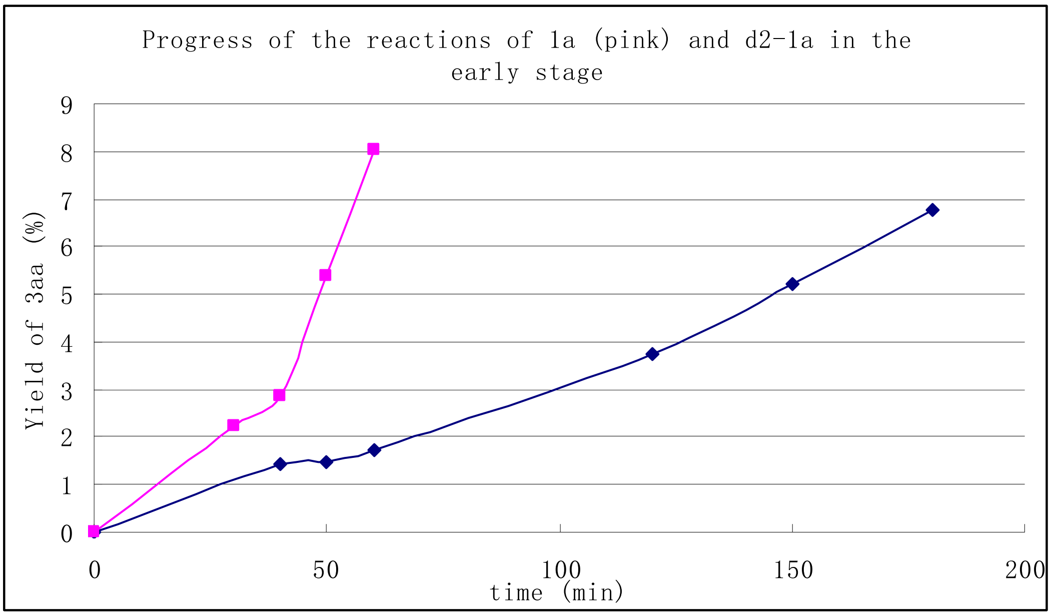


**Figure S1.** Progress of **1a** (pink) and [$D_2$]-**1a** (blue) in the early stage.

Due to the nature of the experiment, plots to determine the KIE were taken between 40-60 min for **1a** and 120-180 min for [$D_2$]-**1a**, respectively (Figure S2).

**Table S3.** For **1a**:

| Time (min) | Yield of **3aa** (%) 1st | 2nd | 3rd | average |
|---|---|---|---|---|
| 40 | 2.4038 | 2.208 | 4.0012 | 2.871 |
| 50 | 5.7793 | 5.372 | 4.9621 | 5.3711 |
| 60 | 9.147 | 7.7231 | 7.2291 | 8.0331 |

**Table S4.** For [$D_2$]-**1a**:

| Time (min) | Yield of [$D_1$]-**3aa** (%) 1st | 2nd | average |
|---|---|---|---|
| 120 | 3.3227 | 4.1438 | 3.7333 |
| 150 | 5.1877 | 5.2617 | 5.2247 |
| 180 | 6.723 | 6.8242 | 6.7736 |

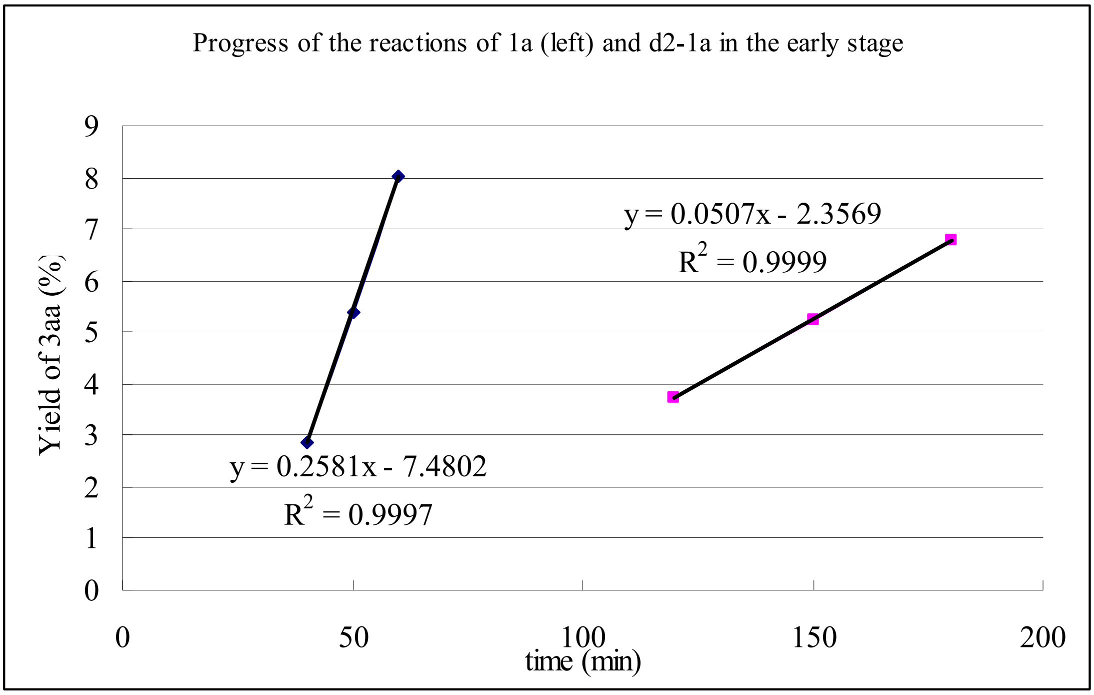


**Figure S2.** Rate comparision for the acetoxylation of **1a** (left) and [$D_2$]-**1a** (right). Data points represent the average of three runs (for **1a**) or two runs (for [$D_2$]-**1a**).

$k_H/k_D$ = (1.2905 x $10^{-3}$ M/min)/(2.5350 x $10^{-4}$ M/min) = 0.2581/0.0507 = 5.1

**Mechanism via initial acyloxylation of allene and detailed mechanism for the formation of products 4, 6, and 7.**

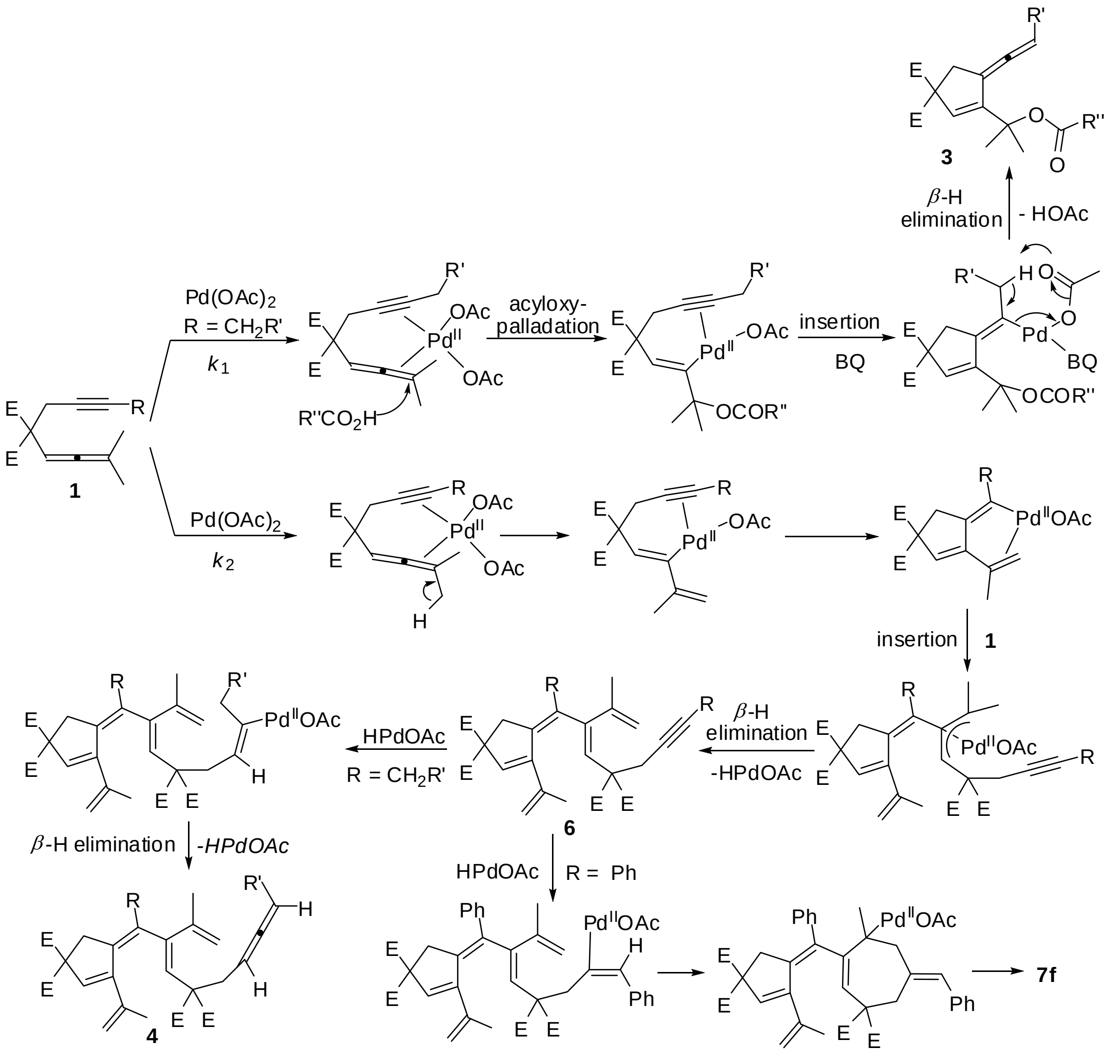


In the mechanism via initial acyloxylation of the allene the ratio between **3** and dimers (**4**, **6** and **7**) would be determined by the ratio $k_1/k_2$, which is independent on deuteration in the propargylic position. The dramatic change of the ratio between **3** and dimers on dideuteration of the propargylic position (Scheme 5b) therefore rules out this mechanism for the formation of **3** (initial acyloxylation of the allene).

**Control experiment using enyne 12.**

A control experiment using enyne **12** was also carried out under the standard conditions of Table 2. No formation of the cyclized allene products **13** or **14** was observed, which shows that the allene moiety in the substrate is crucial for the oxidative transformation (Scheme S2).

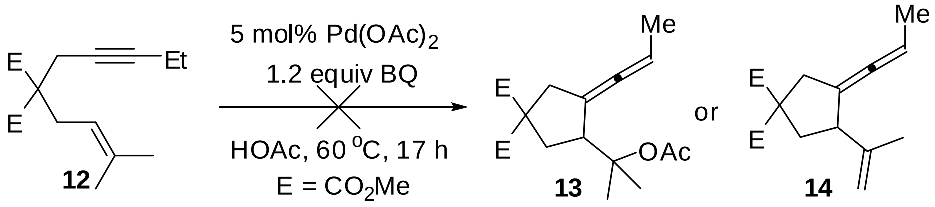


***Scheme S2.*** Reaction of enyne **12** in the presence of acetic acid.

**References:**


1. a) D. K. Black, S. R. Landor, A. N. Patel, P. F. Whiter, *Tetrahedron Lett*, **1963**, *4*, 483; b) A. K. Å. Persson, E. V. Johnston, J.-E. Bäckvall, *Org. Lett.* **2009**, *11*, 3814.
2. C. Sperger, L. H. S. Strand, A. Fiksdahl, *Tetrahedron* **2010**, *66*, 7749.
3. a) V. Pardo-Rodríguez, J. Marco-Martínez, E. Buñuel, D. J. Cárdenas, *Org. Lett.* **2009**, *11*, 4548; b) Y. Deng, T. Bartholomeyzik, A. K. Å. Persson, J. Sun, J.-E. Bäckvall, *Angew. Chem.* **2012**, *124*, 2757; *Angew. Chem. Int. Ed.* **2012**, *51*, 2703.
4. a) J.-L. Abad, S. Rodríguez, F. Camps, G. Fabriàs, *J. Org. Chem.* **2006**, *71*, 7558; b) G. W. Kabalka, M. Varma, R. S. Varma, *J. Org. Chem.* **1986**, *51*, 2386.
5. Y. Akakabe, K. Washizu, K. Matsui, T. Kajiwara, *Biosci. Biotechnol. Biochem.*, **2005**, *69*, 1348.
6. J. G. Millar, M. Giblin, D. Barton, E. W. Underhill, *J. Chem. Ecol.* **1991**, *17*, 911.
7. N. Saito, Y. Kohyama, Y. Tanaka, Y. Sato, *Chem. Commun.*, **2012**, *48*, 3754.
8. T. D. Avery, J. A. Culbert, D. K. Taylor, *Org. Biomol. Chem.*, **2006**, *4*, 323.
9. C. Fu, S. Ma, *Eur. J. Org. Chem.* **2005**, 3942.
10. S. Ma, W. Gao, *Tetrahedron Lett.* **2000**, *41*, 8933.
11. B. Gockel, N. Krause, *Org. Lett.* **2006**, *8*, 4485.

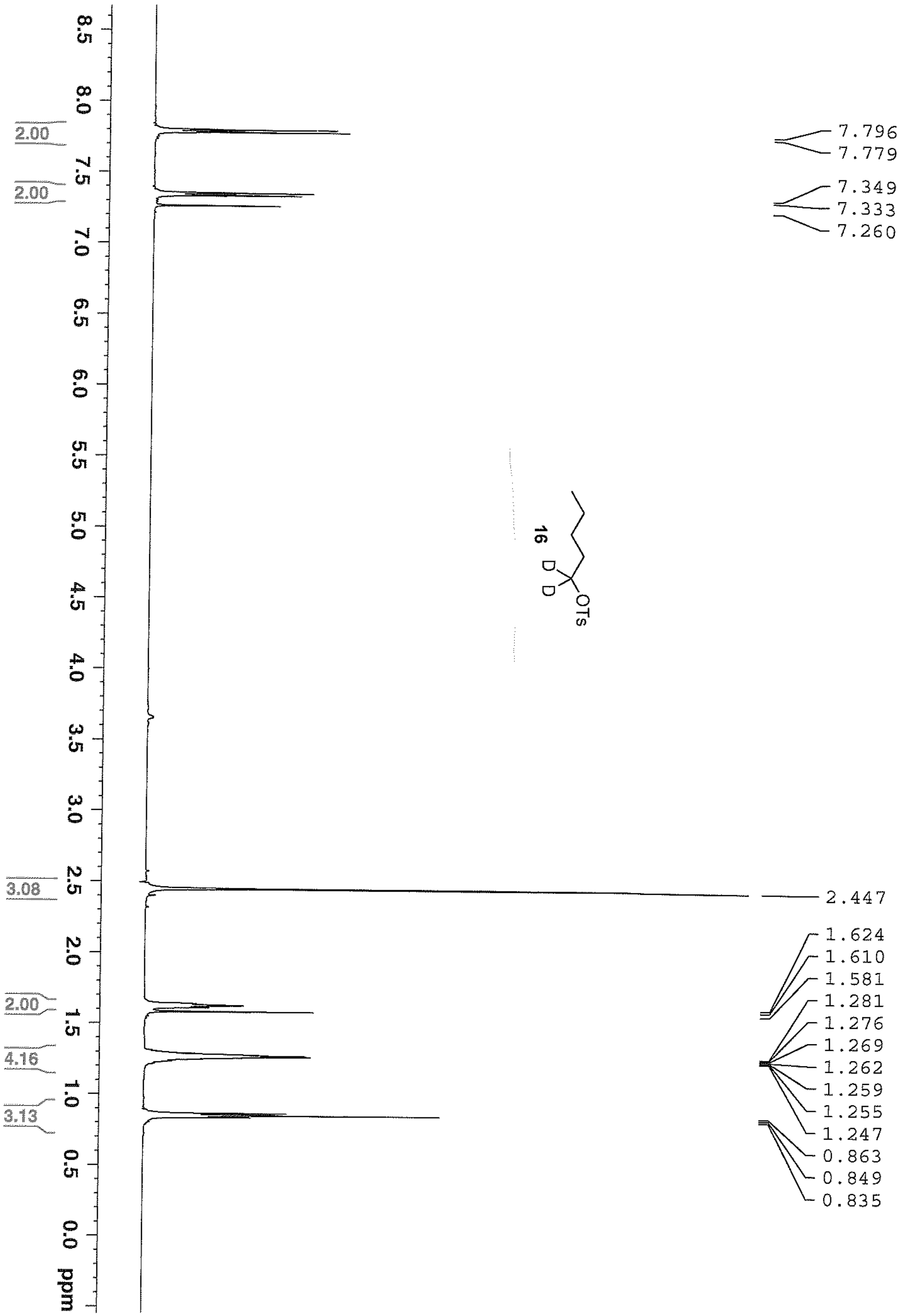
16
OTs
D D
7.796
7.779
7.349
7.333
7.260
2.447
1.624
1.610
1.581
1.281
1.276
1.269
1.262
1.259
1.255
1.247
0.863
0.849
0.835
2.00
2.00
3.08
2.00
4.16
3.13
8.5
8.0
7.5
7.0
6.5
6.0
5.5
5.0
4.5
4.0
3.5
3.0
2.5
2.0
1.5
1.0
0.5
0.0
ppm

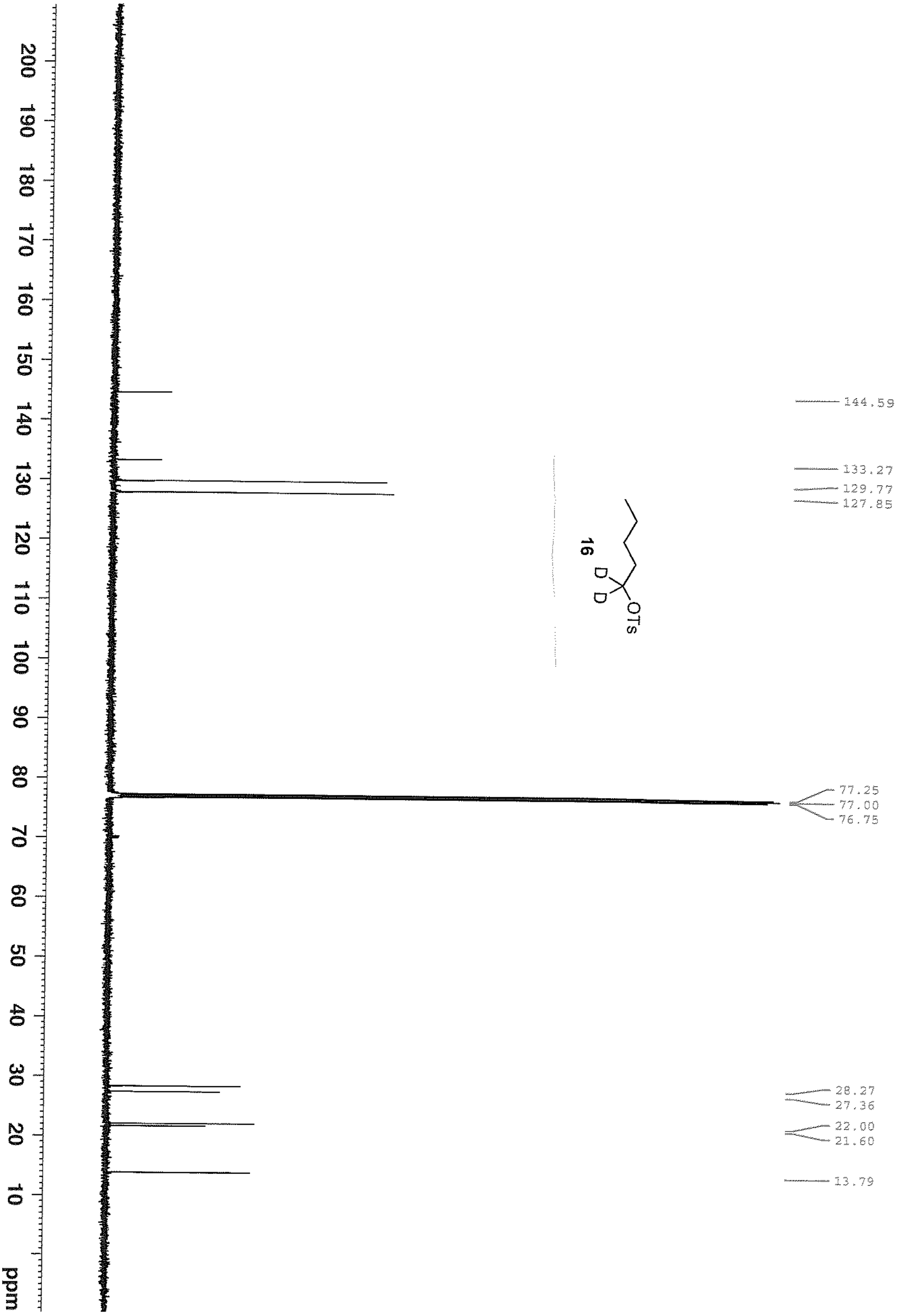
144.59
133.27
129.77
127.85
77.25
77.00
76.75
28.27
27.36
22.00
21.60
13.79
OTs
D
D
16
200
190
180
170
160
150
140
130
120
110
100
90
80
70
60
50
40
30
20
10
ppm

# Mass Spectrum SmartFormula Report

**Analysis Info**

| | | | |
|---|---|---|---|
| Analysis Name | H:\Data2\Youqian\dyq-4-72000002.d | Acquisition Date | 2012-09-24 15:06:39 |
| Method | tune_low_dirk.m | Operator | pia |
| Sample Name | dyq-4-72 | Instrument / Ser# | micrOTOF 125 |
| Comment | | | |

**Acquisition Parameter**

| | | | | | |
|---|---|---|---|---|---|
| Source Type | ESI | Ion Polarity | Positive | Set Nebulizer | 0.4 Bar |
| Focus | Not active | | | Set Dry Heater | 170 °C |
| Scan Begin | 50 m/z | Set Capillary | 4500 V | Set Dry Gas | 4.0 l/min |
| Scan End | 1000 m/z | Set End Plate Offset | -500 V | Set Divert Valve | Source |

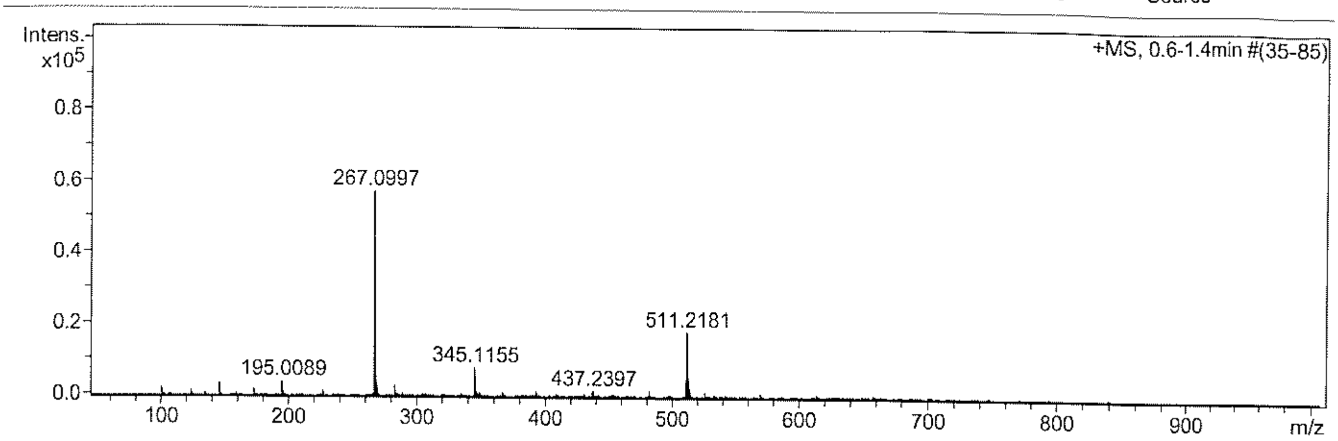


| Formula | Meas. m/z | m/z | err [ppm] | Mean err [ppm] |
|---|---|---|---|---|
| | 267.0997 | | | |
| C 12 H 16 D 2 Na O 3 S | | 267.0994 | -1.1 | -1.4 |

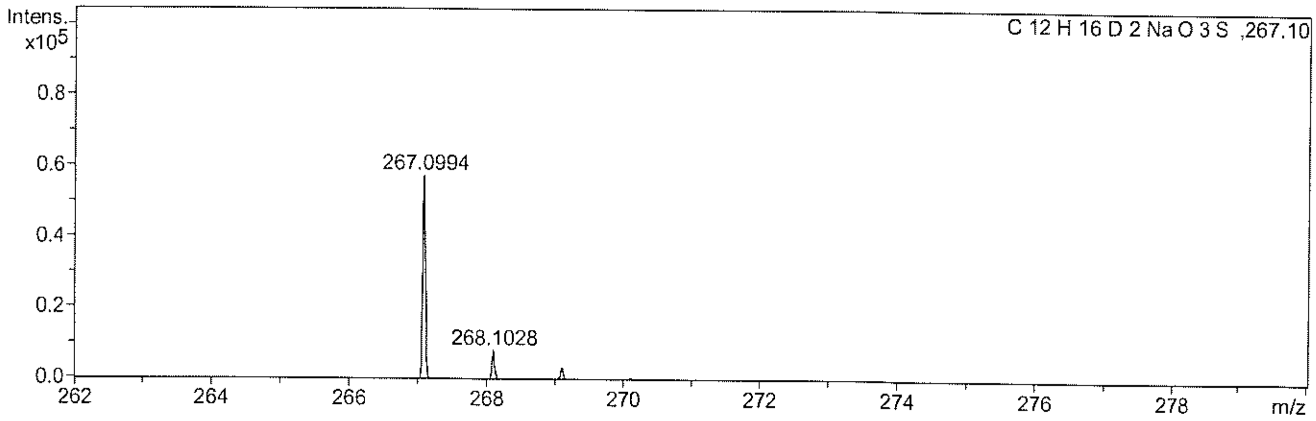


| Formula | Meas. m/z | m/z | err [ppm] | Mean err [ppm] |
|---|---|---|---|---|

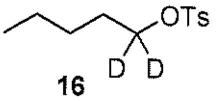


**16**

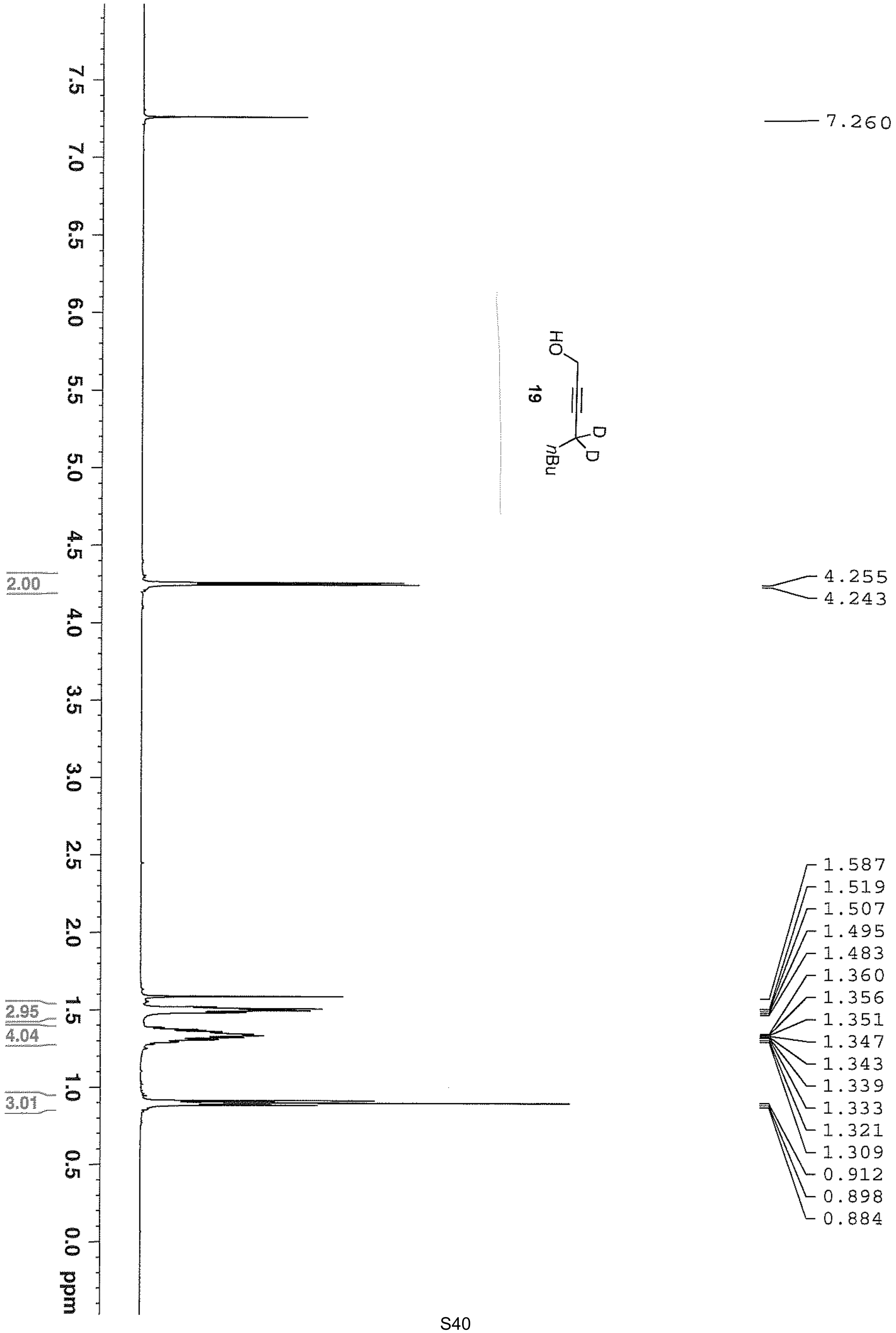
7.260
4.255
4.243
1.587
1.519
1.507
1.495
1.483
1.360
1.356
1.351
1.347
1.343
1.339
1.333
1.321
1.309
0.912
0.898
0.884
HO
D
D
nBu
19
2.00
2.95
4.04
3.01
7.5
7.0
6.5
6.0
5.5
5.0
4.5
4.0
3.5
3.0
2.5
2.0
1.5
1.0
0.5
0.0
ppm

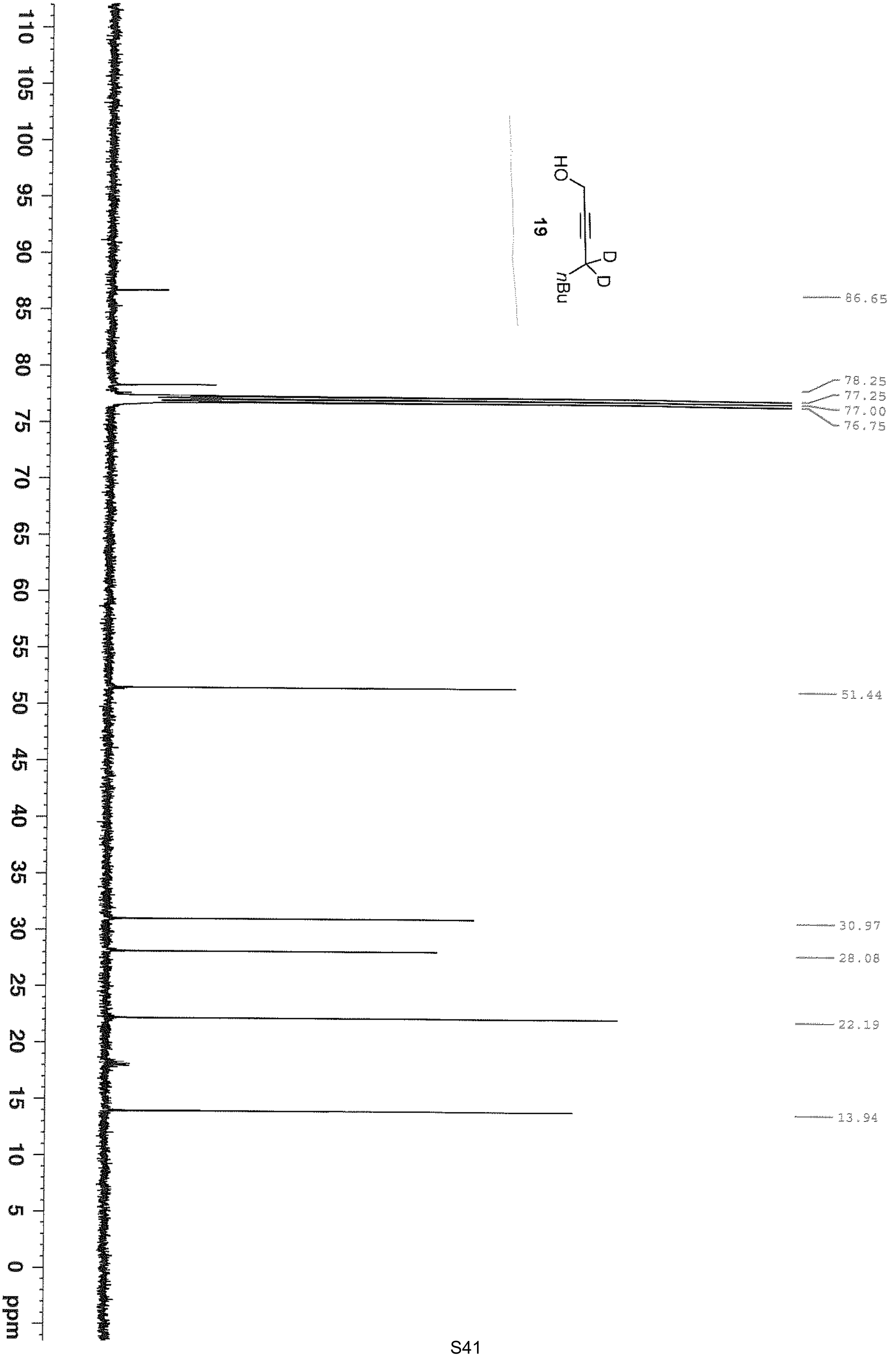
86.65
78.25
77.25
77.00
76.75
51.44
30.97
28.08
22.19
13.94
HO
D
D
nBu
19
110
105
100
95
90
85
80
75
70
65
60
55
50
45
40
35
30
25
20
15
10
5
0
ppm

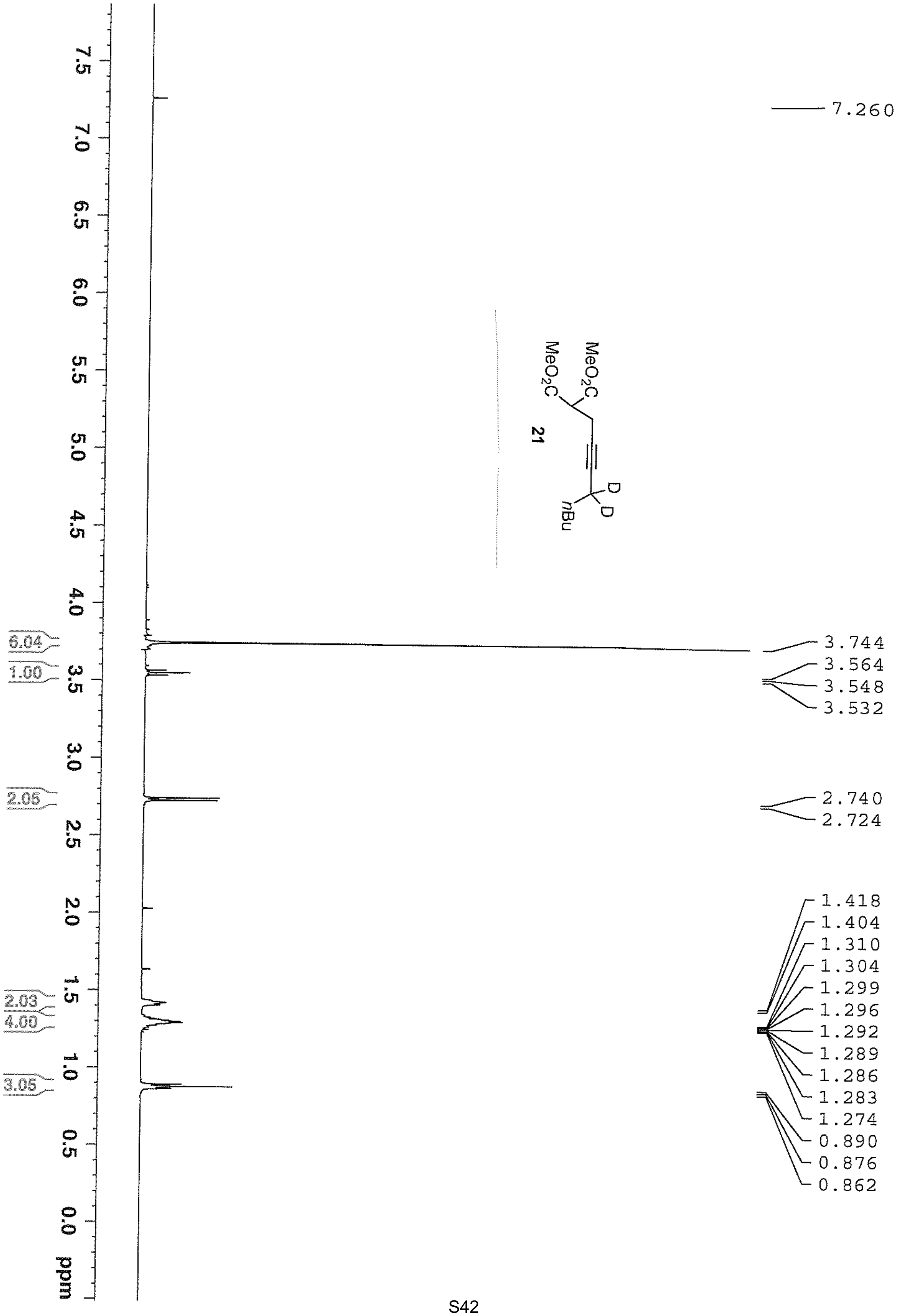

7.260
3.744
3.564
3.548
3.532
2.740
2.724
1.418
1.404
1.310
1.304
1.299
1.296
1.292
1.289
1.286
1.283
1.274
0.890
0.876
0.862
6.04
1.00
2.05
2.03
4.00
3.05
MeO2C
MeO2C
D
D
nBu
21
ppm

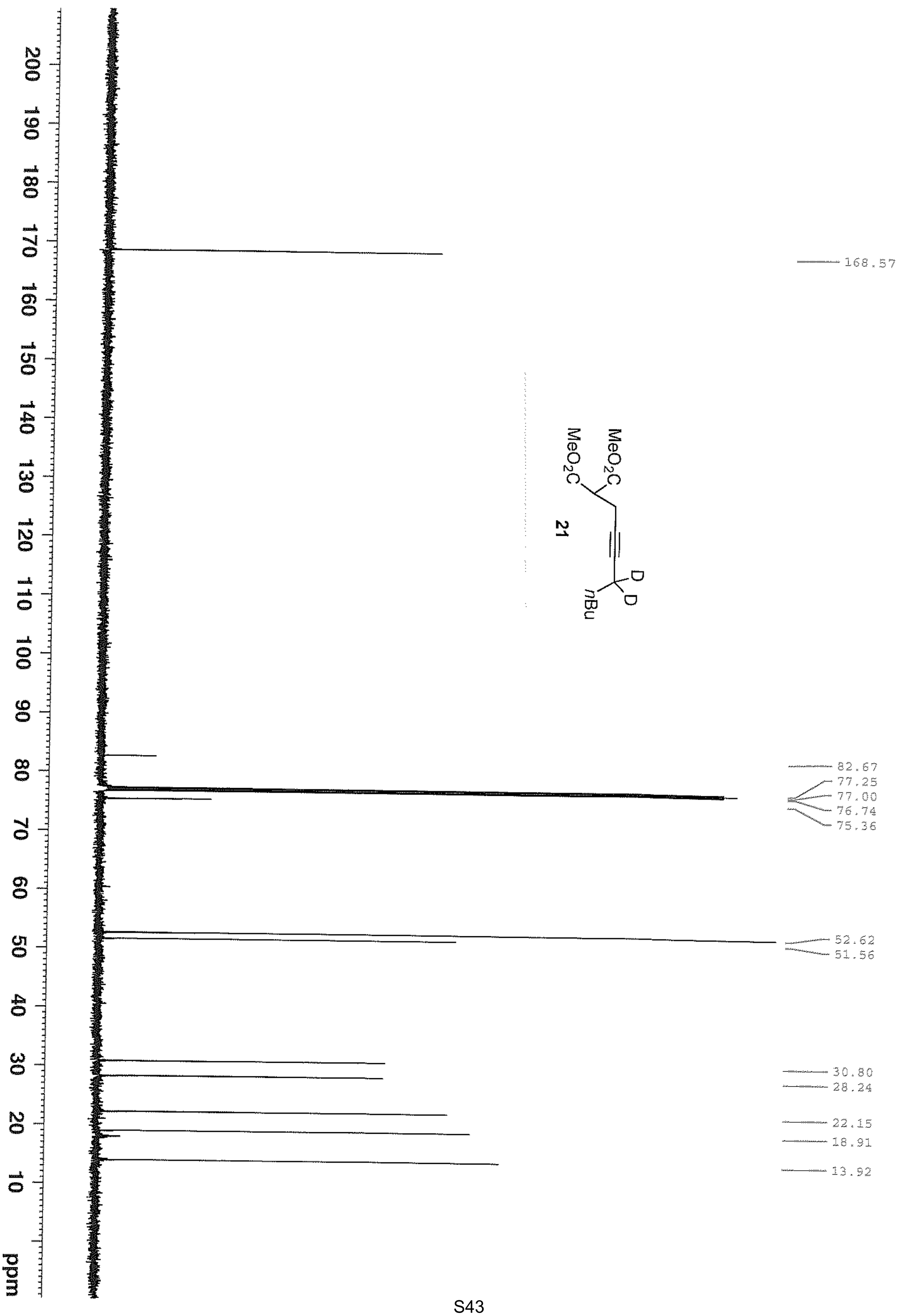
168.57
82.67
77.25
77.00
76.74
75.36
52.62
51.56
30.80
28.24
22.15
18.91
13.92
MeO2C
MeO2C
D
D
nBu
21
200
190
180
170
160
150
140
130
120
110
100
90
80
70
60
50
40
30
20
10
ppm

# Mass Spectrum SmartFormula Report

**Analysis Info**

| | | | |
|---|---|---|---|
| Analysis Name | H:\Data2\Youqian\dyq-4-84000005.d | Acquisition Date | 2012-09-24 16:06:12 |
| Method | tune_low_dirk.m | Operator | pia |
| Sample Name | dyq-4-84 | Instrument / Ser# | micrOTOF 125 |
| Comment | | | |

**Acquisition Parameter**

| | | | | | |
|---|---|---|---|---|---|
| Source Type | ESI | Ion Polarity | Positive | Set Nebulizer | 0.4 Bar |
| Focus | Not active | | | Set Dry Heater | 170 °C |
| Scan Begin | 50 m/z | Set Capillary | 4500 V | Set Dry Gas | 4.0 l/min |
| Scan End | 1000 m/z | Set End Plate Offset | -500 V | Set Divert Valve | Source |

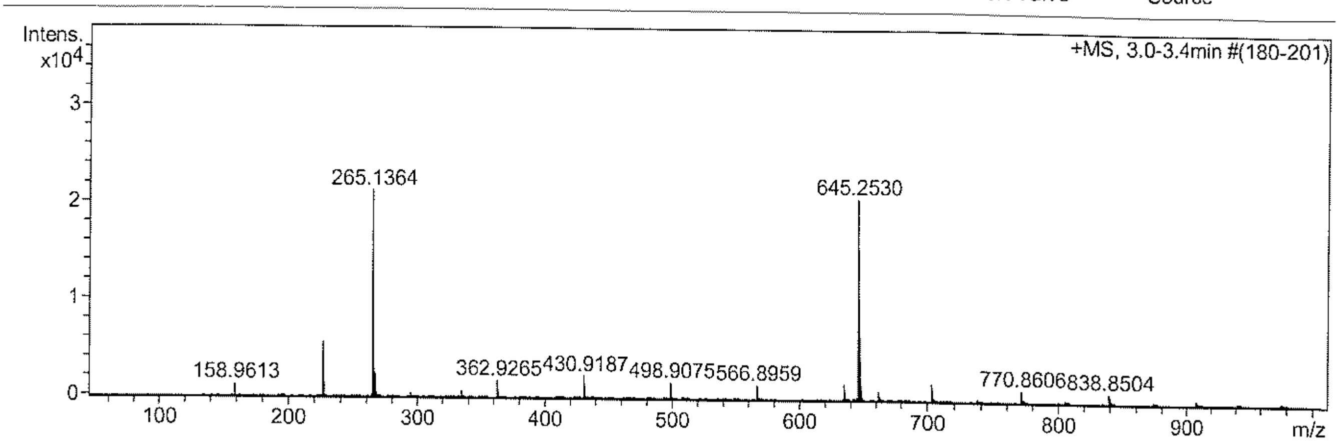


| Formula | Meas. m/z | m/z | err [ppm] | Mean err [ppm] |
|---|---|---|---|---|
| | 265.1364 | | | |
| C 13 H 18 D 2 Na O 4 | | 265.1379 | 5.7 | 5.8 |

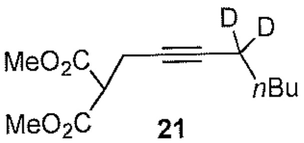


**21**

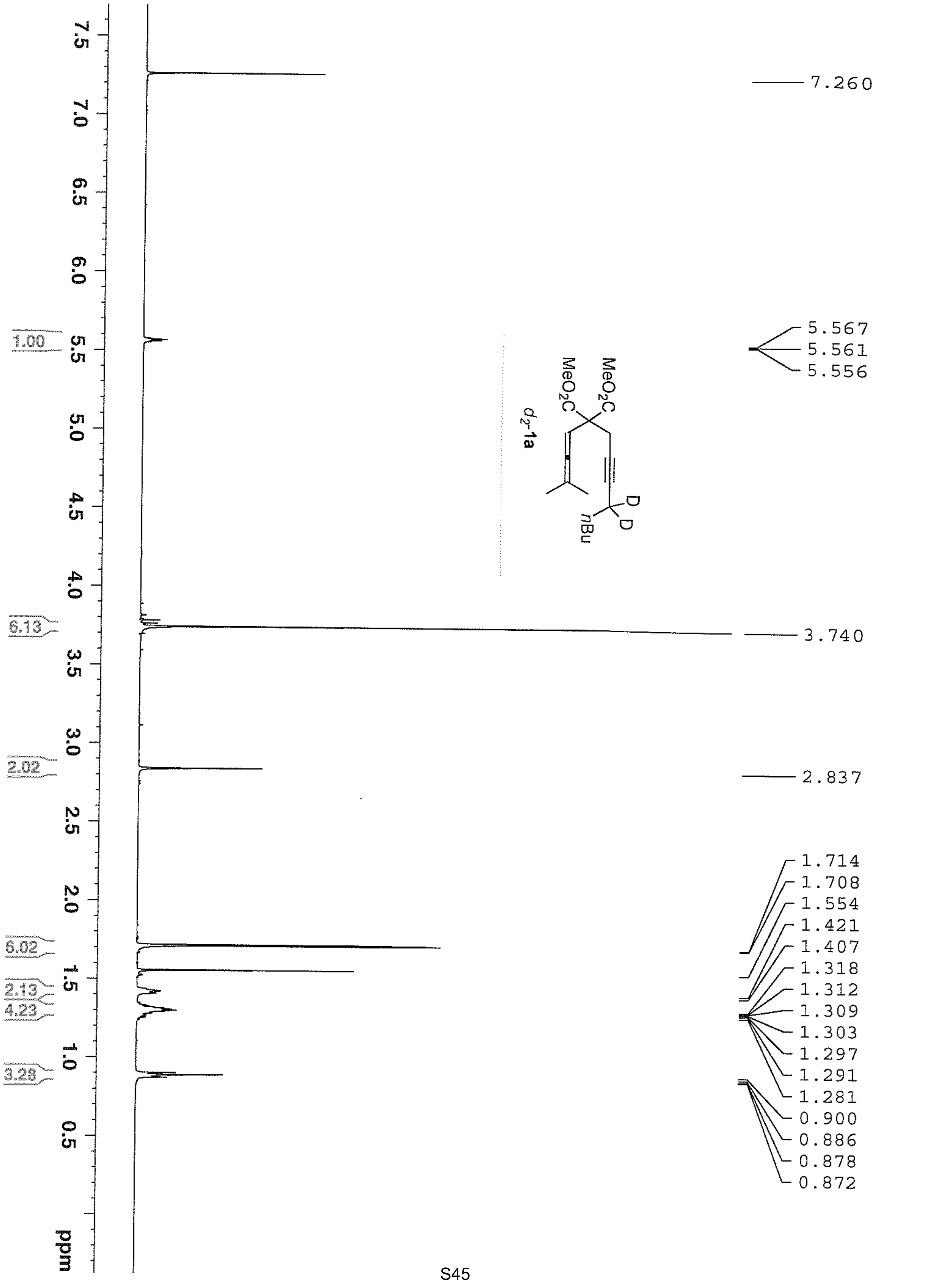
7.260
5.567
5.561
5.556
3.740
2.837
1.714
1.708
1.554
1.421
1.407
1.318
1.312
1.309
1.303
1.297
1.291
1.281
0.900
0.886
0.878
0.872
1.00
6.13
2.02
6.02
2.13
4.23
3.28
MeO2C
MeO2C
D
D
nBu
d2-1a
7.5
7.0
6.5
6.0
5.5
5.0
4.5
4.0
3.5
3.0
2.5
2.0
1.5
1.0
0.5
ppm

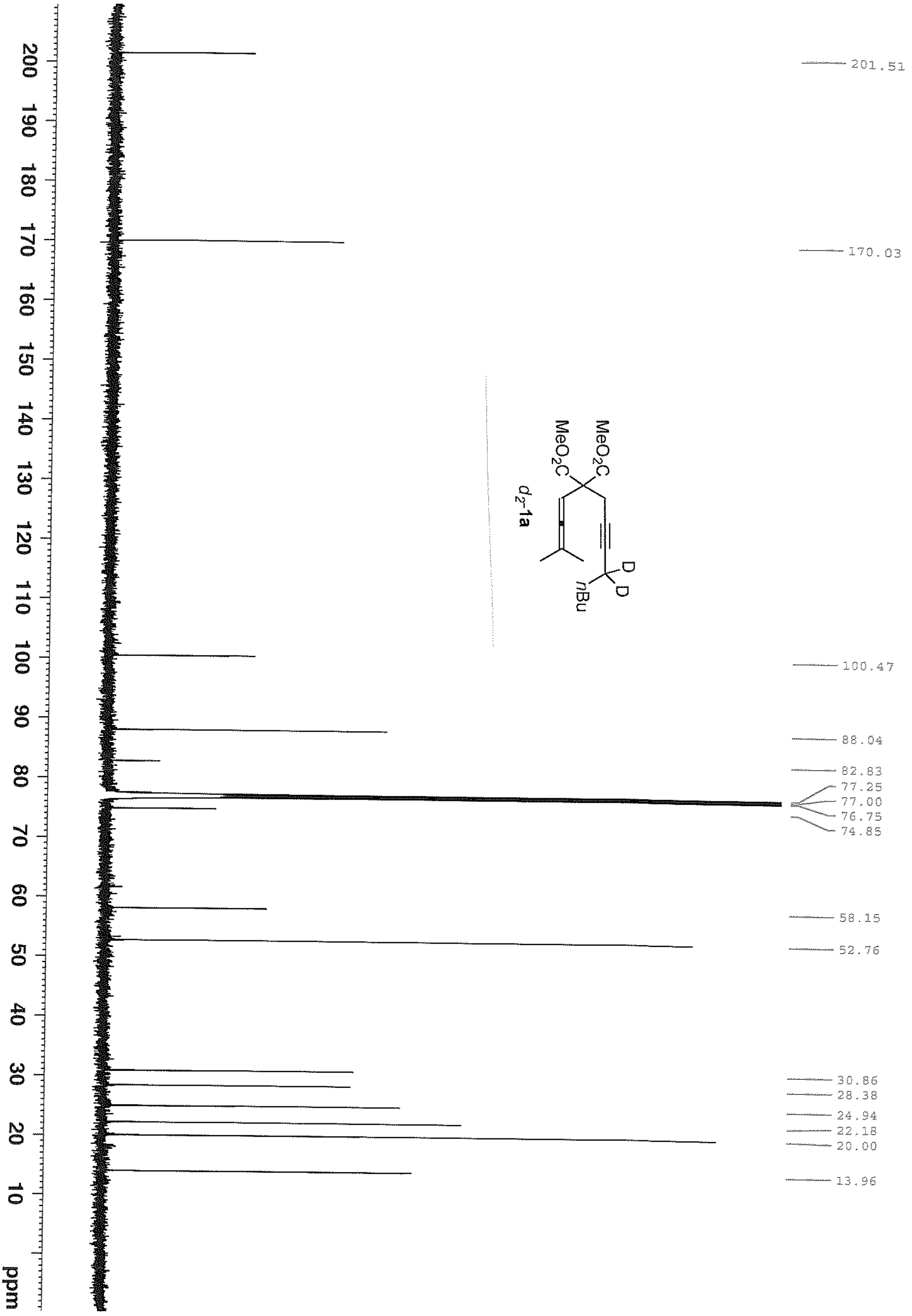
201.51
170.03
100.47
88.04
82.83
77.25
77.00
76.75
74.85
58.15
52.76
30.86
28.38
24.94
22.18
20.00
13.96
MeO2C
MeO2C
D
D
nBu
d2-1a
ppm

# Mass Spectrum SmartFormula Report

**Analysis Info**

| | | | |
|---|---|---|---|
| Analysis Name | H:\Data2\Youqian\dyq-4-85000001.d | Acquisition Date | 2012-09-24 16:16:46 |
| Method | tune_low_dirk.m | Operator | pia |
| Sample Name | dyq-4-85 | Instrument / Ser# | micrOTOF 125 |
| Comment | | | |

**Acquisition Parameter**

| | | | | | |
|---|---|---|---|---|---|
| Source Type | ESI | Ion Polarity | Positive | Set Nebulizer | 0.4 Bar |
| Focus | Not active | | | Set Dry Heater | 170 °C |
| Scan Begin | 50 m/z | Set Capillary | 4500 V | Set Dry Gas | 4.0 l/min |
| Scan End | 1000 m/z | Set End Plate Offset | -500 V | Set Divert Valve | Source |

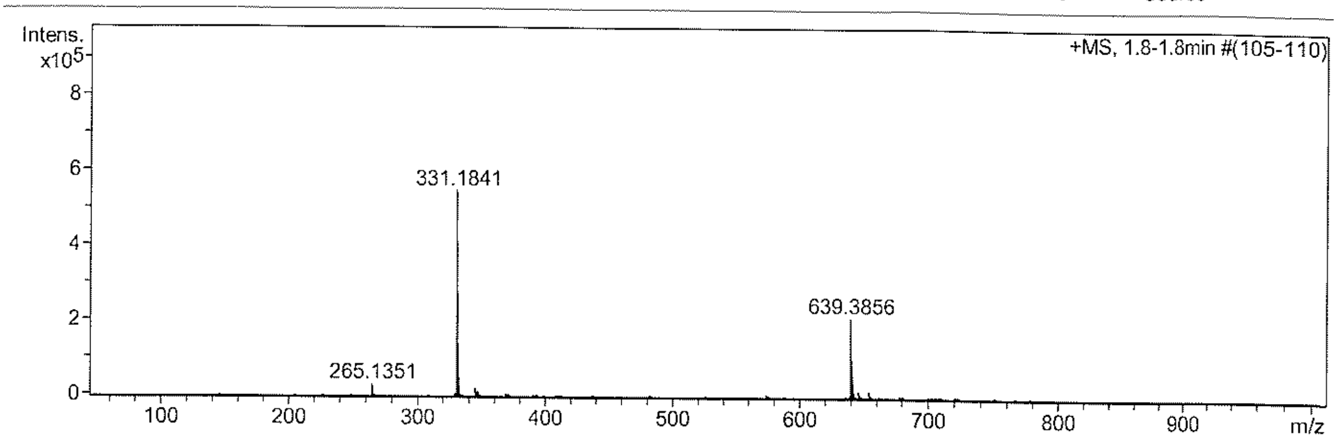


| Formula | Meas. m/z | m/z | err [ppm] | Mean err [ppm] |
|---|---|---|---|---|
| | 331.1841 | | | |
| C 18 H 24 D 2 Na O 4 | | 331.1849 | 2.4 | 2.8 |

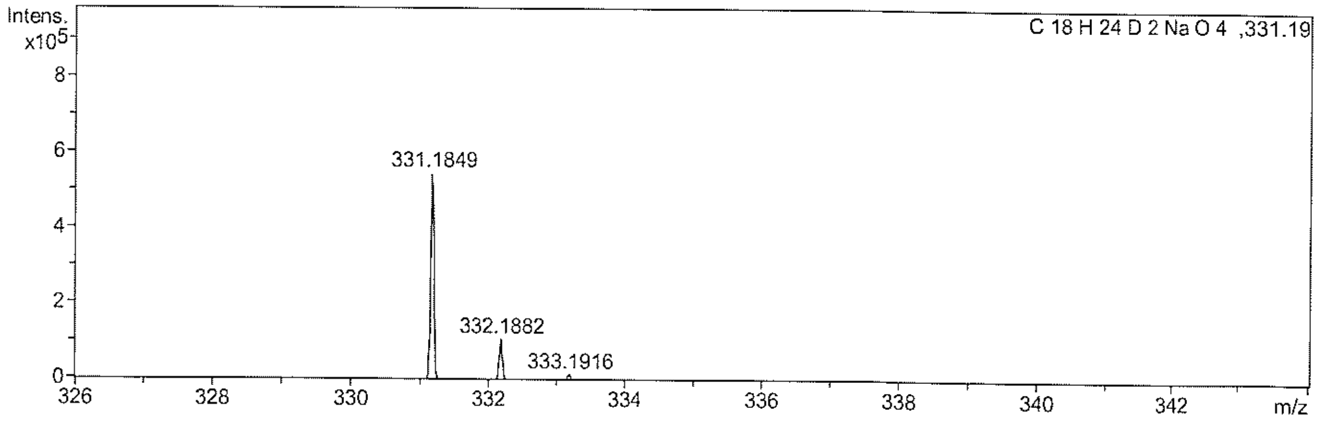


| Formula | Meas. m/z | m/z | err [ppm] | Mean err [ppm] |
|---|---|---|---|---|

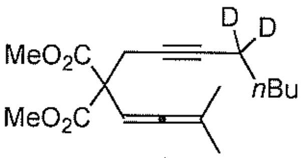


$d_2$-**1a**

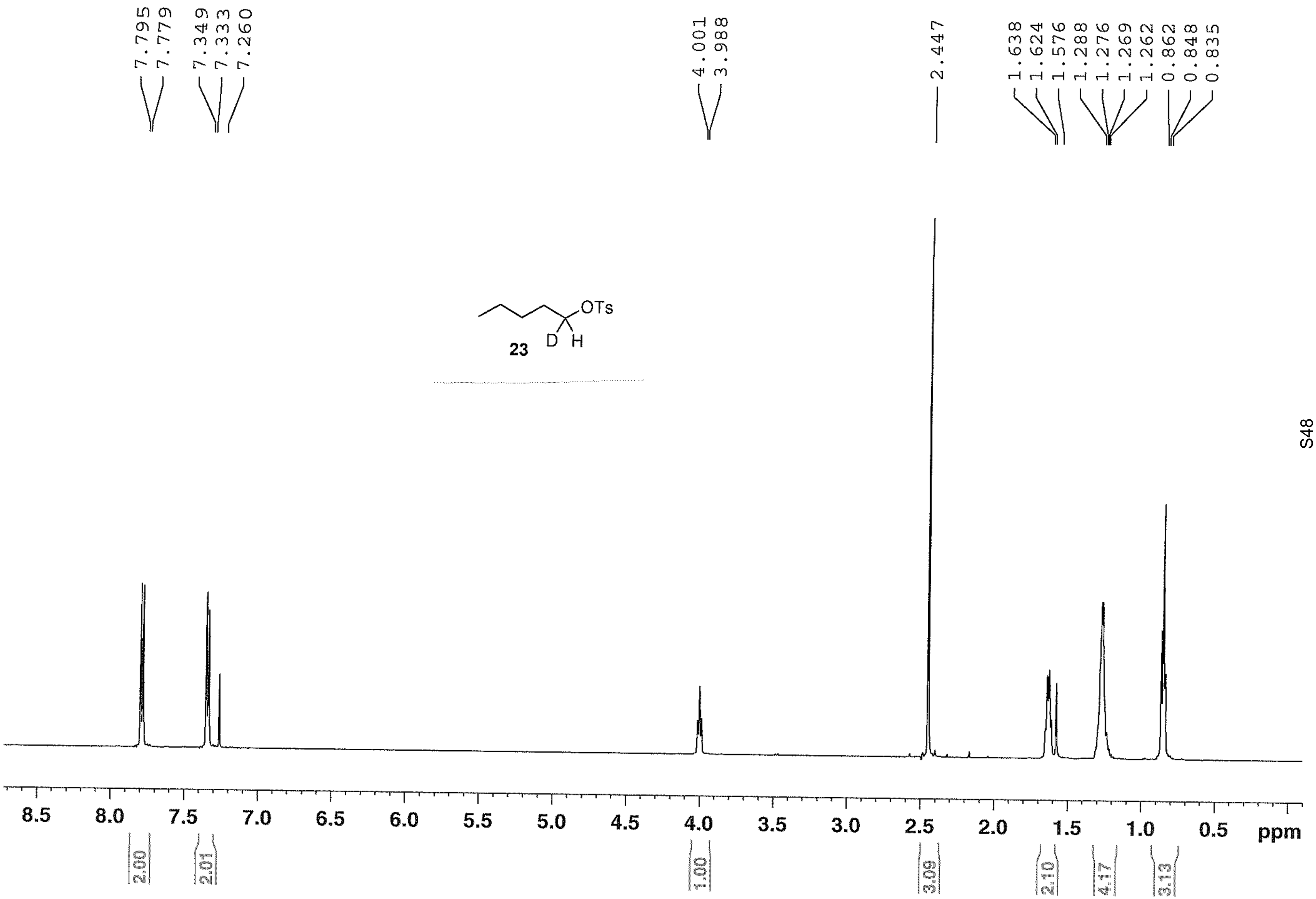
7.795
7.779
7.349
7.333
7.260
4.001
3.988
2.447
1.638
1.624
1.576
1.288
1.276
1.269
1.262
0.862
0.848
0.835
OTs
D
H
23
8.5
8.0
7.5
7.0
6.5
6.0
5.5
5.0
4.5
4.0
3.5
3.0
2.5
2.0
1.5
1.0
0.5
ppm
2.00
2.01
1.00
3.09
2.10
4.17
3.13

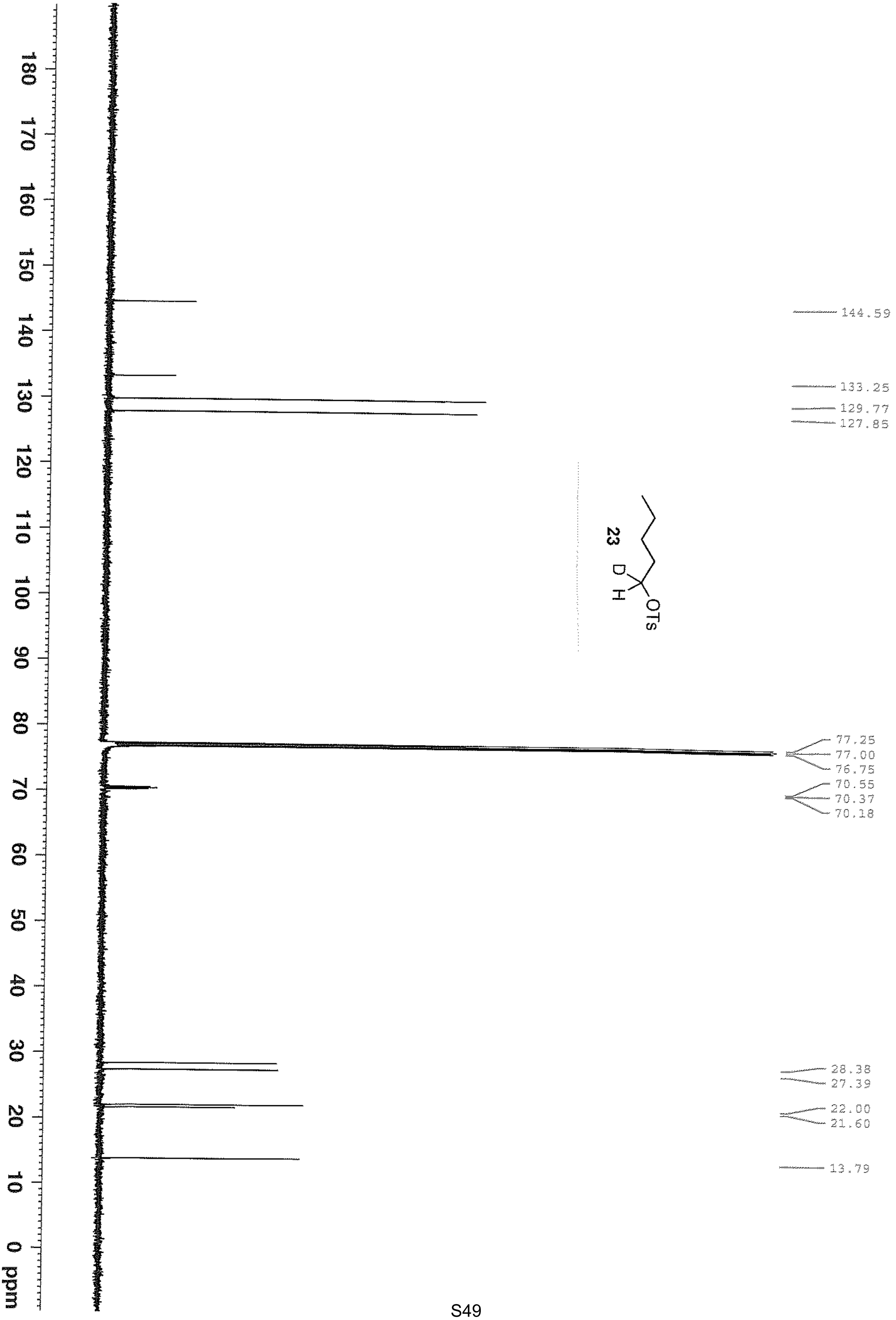
144.59
133.25
129.77
127.85
77.25
77.00
76.75
70.55
70.37
70.18
28.38
27.39
22.00
21.60
13.79
OTs
D H
23
180
170
160
150
140
130
120
110
100
90
80
70
60
50
40
30
20
10
0 ppm

# Mass Spectrum SmartFormula Report

**Analysis Info**

| | | | |
|---|---|---|---|
| Analysis Name | H:\Data2\Youqian\dyq-4-89000001.d | Acquisition Date | 2012-09-24 16:21:46 |
| Method | tune_low_dirk.m | Operator | pia |
| Sample Name | dyq-4-89 | Instrument / Ser# | micrOTOF 125 |
| Comment | | | |

**Acquisition Parameter**

| | | | | | |
|---|---|---|---|---|---|
| Source Type | ESI | Ion Polarity | Positive | Set Nebulizer | 0.4 Bar |
| Focus | Not active | | | Set Dry Heater | 170 °C |
| Scan Begin | 50 m/z | Set Capillary | 4500 V | Set Dry Gas | 4.0 l/min |
| Scan End | 1000 m/z | Set End Plate Offset | -500 V | Set Divert Valve | Source |

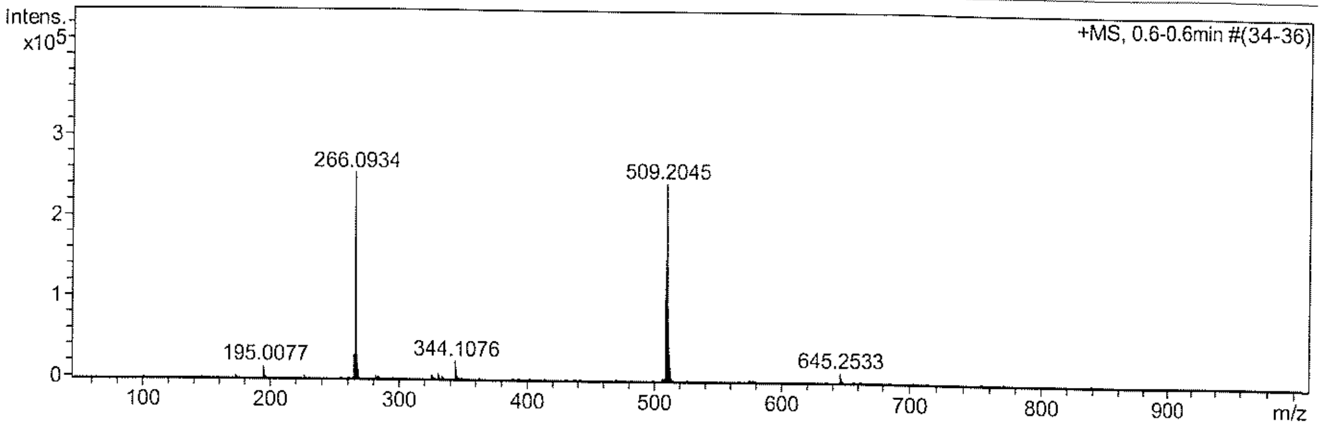


| Formula | Meas. m/z | m/z | err [ppm] | Mean err [ppm] |
|---|---|---|---|---|
| | 266.0934 | | | |
| C 12 H 17 D Na O 3 S | | 266.0932 | -1.1 | -1.2 |

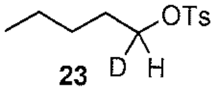


**23**

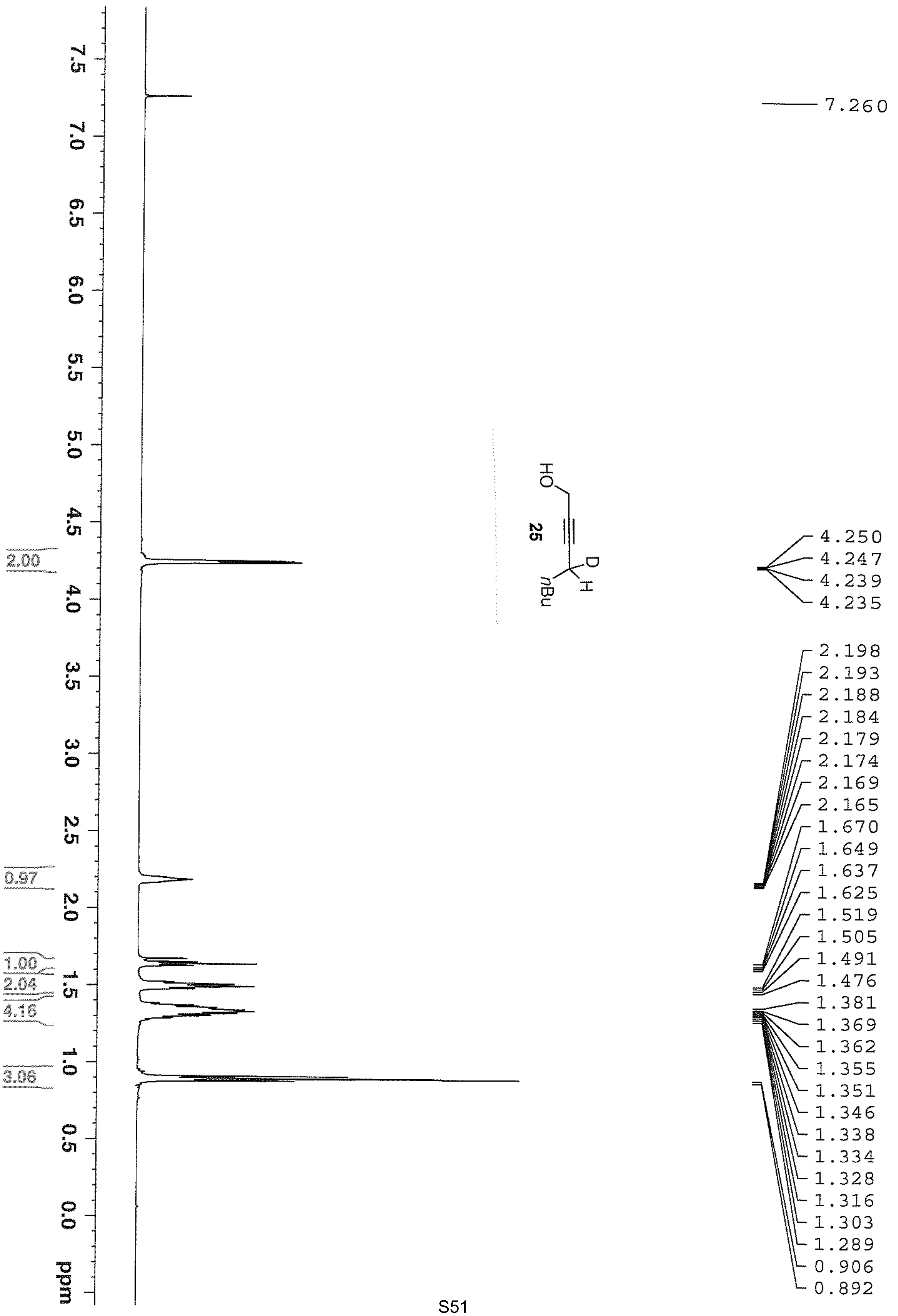
7.260
4.250
4.247
4.239
4.235
2.198
2.193
2.188
2.184
2.179
2.174
2.169
2.165
1.670
1.649
1.637
1.625
1.519
1.505
1.491
1.476
1.381
1.369
1.362
1.355
1.351
1.346
1.338
1.334
1.328
1.316
1.303
1.289
0.906
0.892
HO
D
H
nBu
25
2.00
0.97
1.00
2.04
4.16
3.06
7.5
7.0
6.5
6.0
5.5
5.0
4.5
4.0
3.5
3.0
2.5
2.0
1.5
1.0
0.5
0.0
ppm

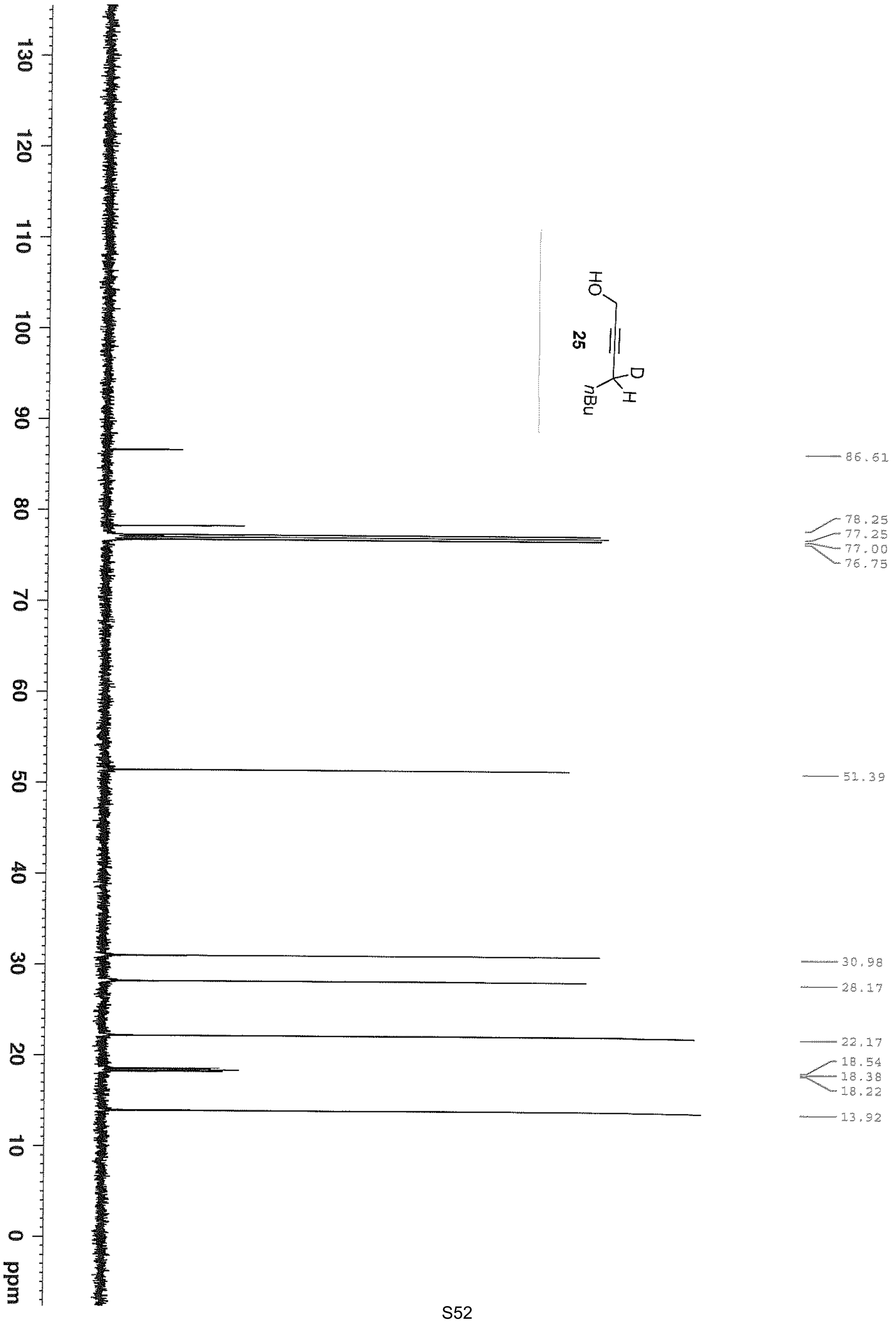

HO
D
H
nBu
25
86.61
78.25
77.25
77.00
76.75
51.39
30.98
28.17
22.17
18.54
18.38
18.22
13.92
130
120
110
100
90
80
70
60
50
40
30
20
10
0
ppm

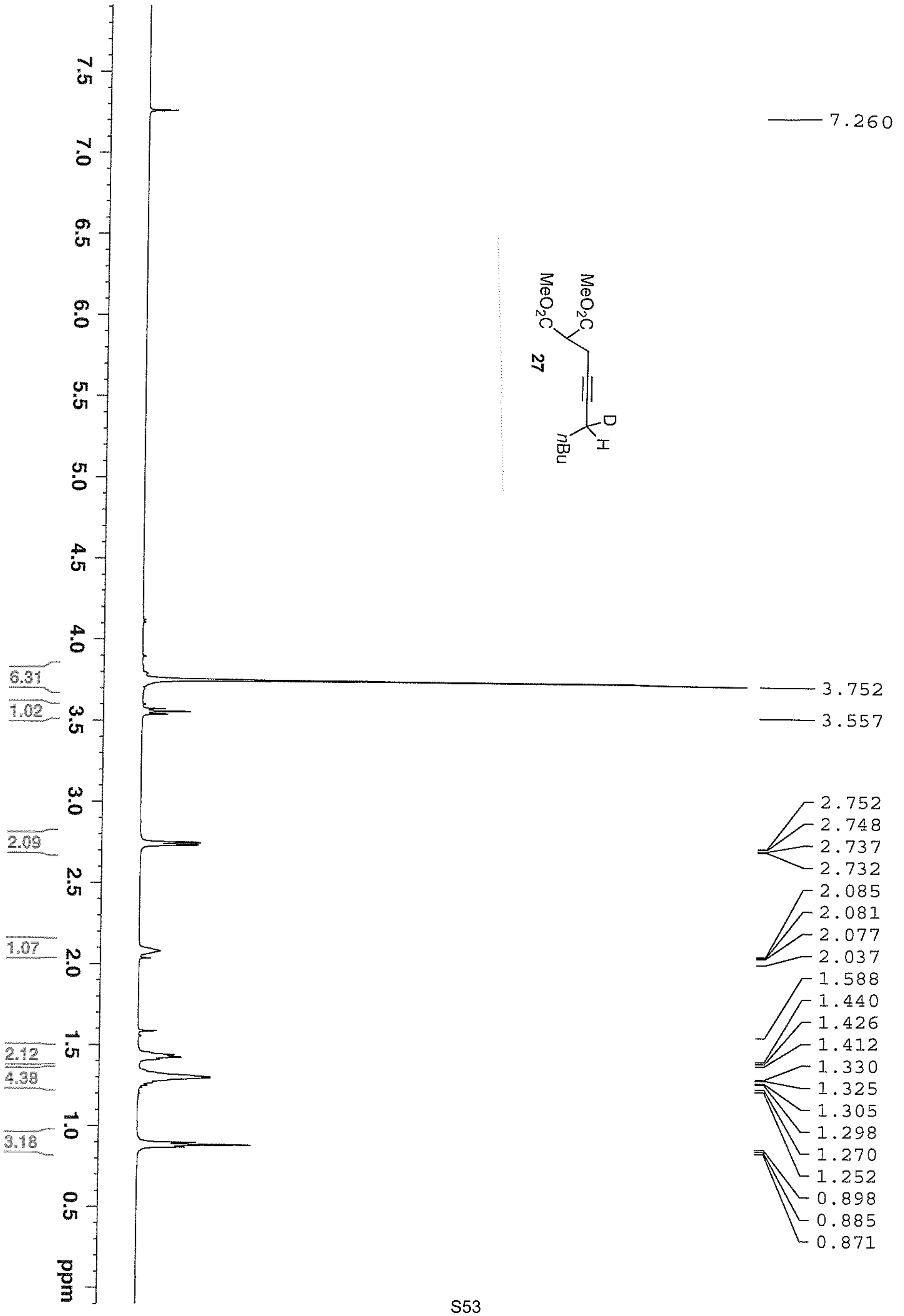
MeO2C
MeO2C
27
D
H
nBu
7.260
3.752
3.557
2.752
2.748
2.737
2.732
2.085
2.081
2.077
2.037
1.588
1.440
1.426
1.412
1.330
1.325
1.305
1.298
1.270
1.252
0.898
0.885
0.871
6.31
1.02
2.09
1.07
2.12
4.38
3.18
7.5
7.0
6.5
6.0
5.5
5.0
4.5
4.0
3.5
3.0
2.5
2.0
1.5
1.0
0.5
ppm

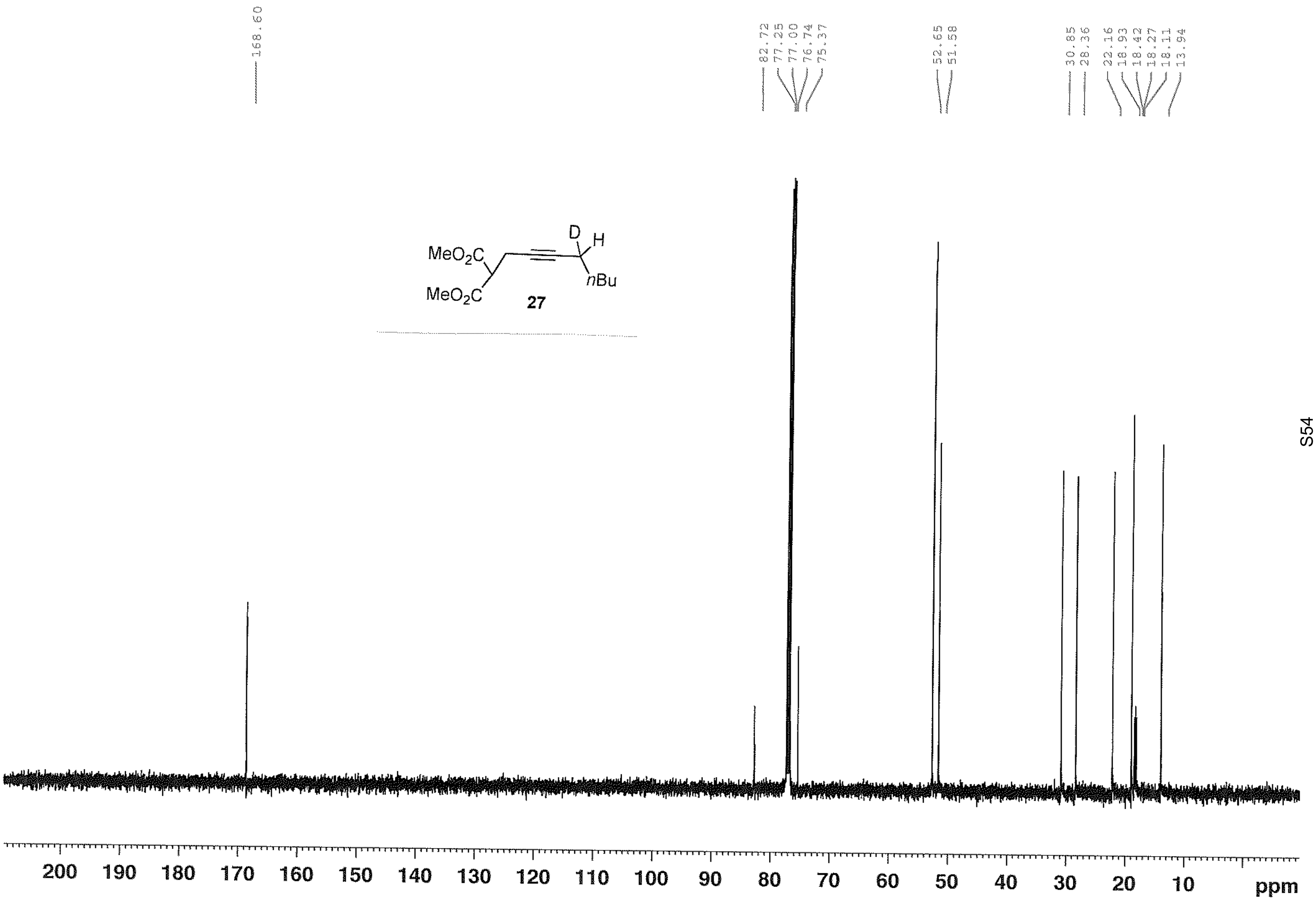
168.60
82.72
77.25
77.00
76.74
75.37
52.65
51.58
30.85
28.36
22.16
18.93
18.42
18.27
18.11
13.94
MeO2C
MeO2C
D
H
nBu
27
200
190
180
170
160
150
140
130
120
110
100
90
80
70
60
50
40
30
20
10
ppm

# Mass Spectrum SmartFormula Report

**Analysis Info**

| | | | |
|---|---|---|---|
| Analysis Name | H:\Data2\Youqian\dyq-4-97000001.d | Acquisition Date | 2012-09-24 16:28:17 |
| Method | tune_low_dirk.m | Operator | pia |
| Sample Name | dyq-4-97 | Instrument / Ser# | micrOTOF 125 |
| Comment | | | |

**Acquisition Parameter**

| | | | | | |
|---|---|---|---|---|---|
| Source Type | ESI | Ion Polarity | Positive | Set Nebulizer | 0.4 Bar |
| Focus | Not active | | | Set Dry Heater | 170 °C |
| Scan Begin | 50 m/z | Set Capillary | 4500 V | Set Dry Gas | 4.0 l/min |
| Scan End | 1000 m/z | Set End Plate Offset | -500 V | Set Divert Valve | Source |

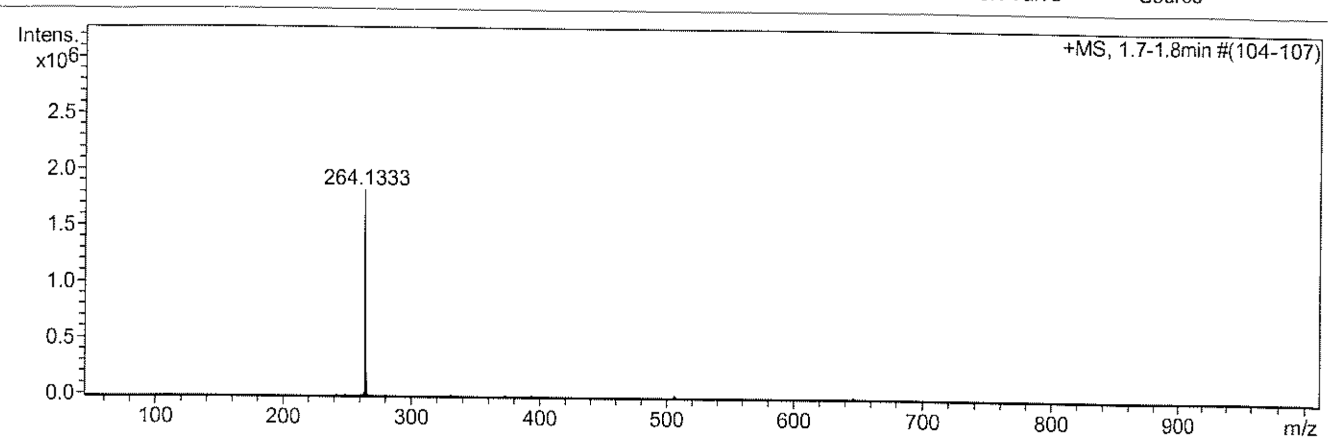


| Formula | Meas. m/z | m/z | err [ppm] | Mean err [ppm] |
|---|---|---|---|---|
| | 264.1333 | | | |
| C 13 H 19 D Na O 4 | | 264.1317 | -6.3 | -5.6 |

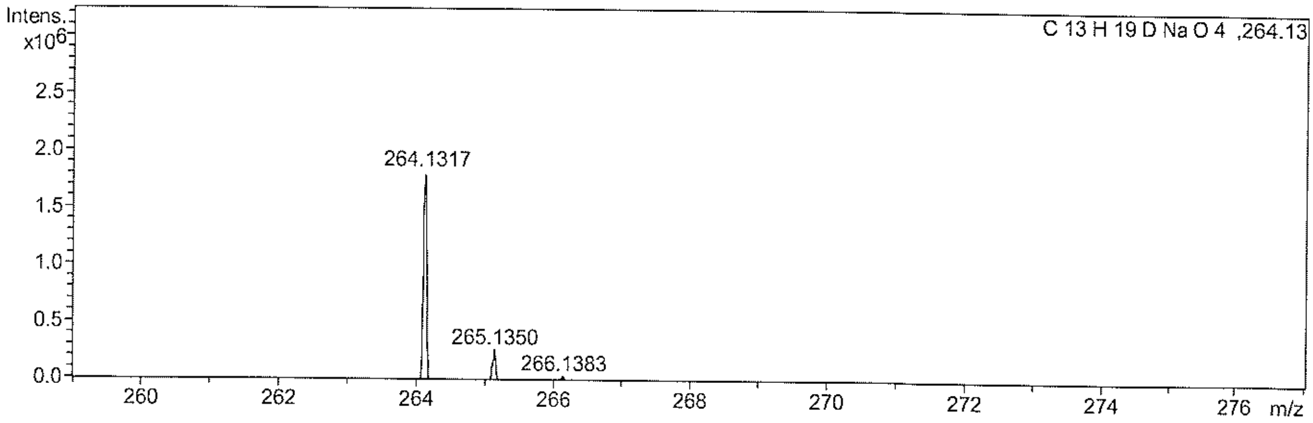


| Formula | Meas. m/z | m/z | err [ppm] | Mean err [ppm] |
|---|---|---|---|---|

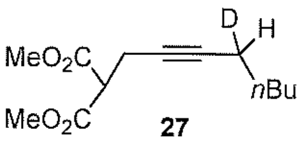


**27**

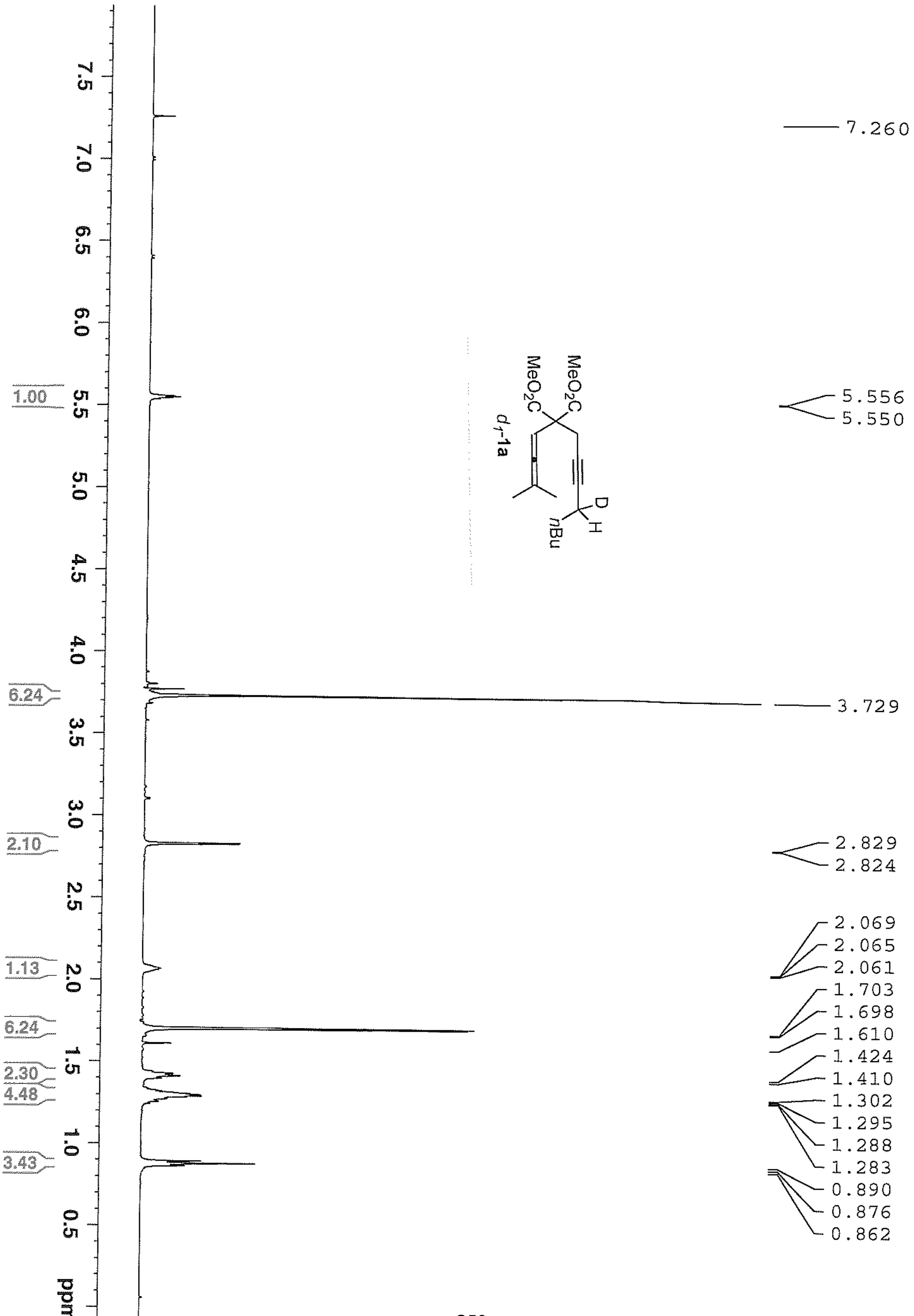
7.260
5.556
5.550
3.729
2.829
2.824
2.069
2.065
2.061
1.703
1.698
1.610
1.424
1.410
1.302
1.295
1.288
1.283
0.890
0.876
0.862
1.00
6.24
2.10
1.13
6.24
2.30
4.48
3.43
7.5
7.0
6.5
6.0
5.5
5.0
4.5
4.0
3.5
3.0
2.5
2.0
1.5
1.0
0.5
ppm
MeO2C
MeO2C
D
H
nBu
d1-1a

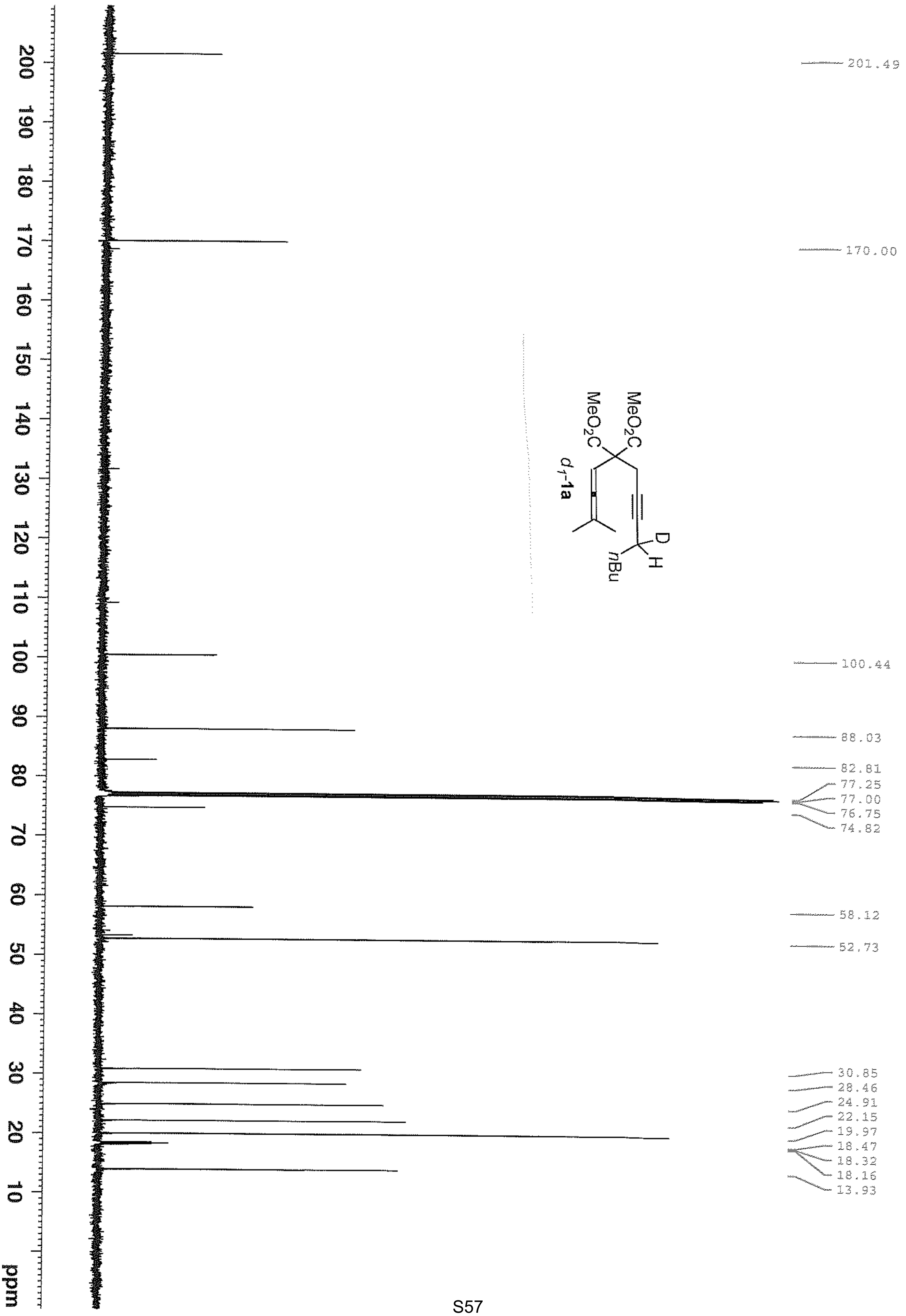
201.49
170.00
100.44
88.03
82.81
77.25
77.00
76.75
74.82
58.12
52.73
30.85
28.46
24.91
22.15
19.97
18.47
18.32
18.16
13.93
MeO2C
MeO2C
D
H
nBu
d1-1a
ppm

# Mass Spectrum SmartFormula Report

**Analysis Info**

| | | | |
|---|---|---|---|
| Analysis Name | H:\Data2\Youqian\dyq-4-98000001.d | Acquisition Date | 2012-09-24 16:36:42 |
| Method | tune_low_dirk.m | Operator | pia |
| Sample Name | dyq-4-98 | Instrument / Ser# | micrOTOF 125 |
| Comment | | | |

**Acquisition Parameter**

| | | | | | |
|---|---|---|---|---|---|
| Source Type | ESI | Ion Polarity | Positive | Set Nebulizer | 0.4 Bar |
| Focus | Not active | | | Set Dry Heater | 170 °C |
| Scan Begin | 50 m/z | Set Capillary | 4500 V | Set Dry Gas | 4.0 l/min |
| Scan End | 1000 m/z | Set End Plate Offset | -500 V | Set Divert Valve | Source |

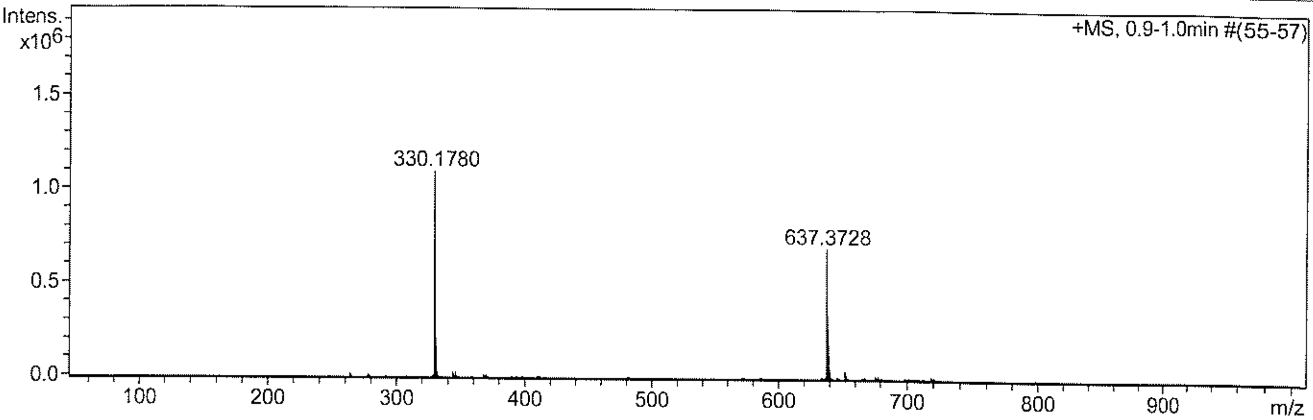


| Formula | Meas. m/z | m/z | err [ppm] | Mean err [ppm] |
|---|---|---|---|---|
| | 330.1780 | | | |
| C 18 H 25 D Na O 4 | | 330.1786 | 1.9 | 2.3 |

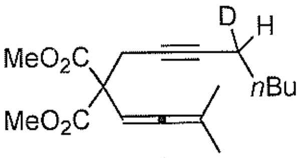


$d_1$-**1a**

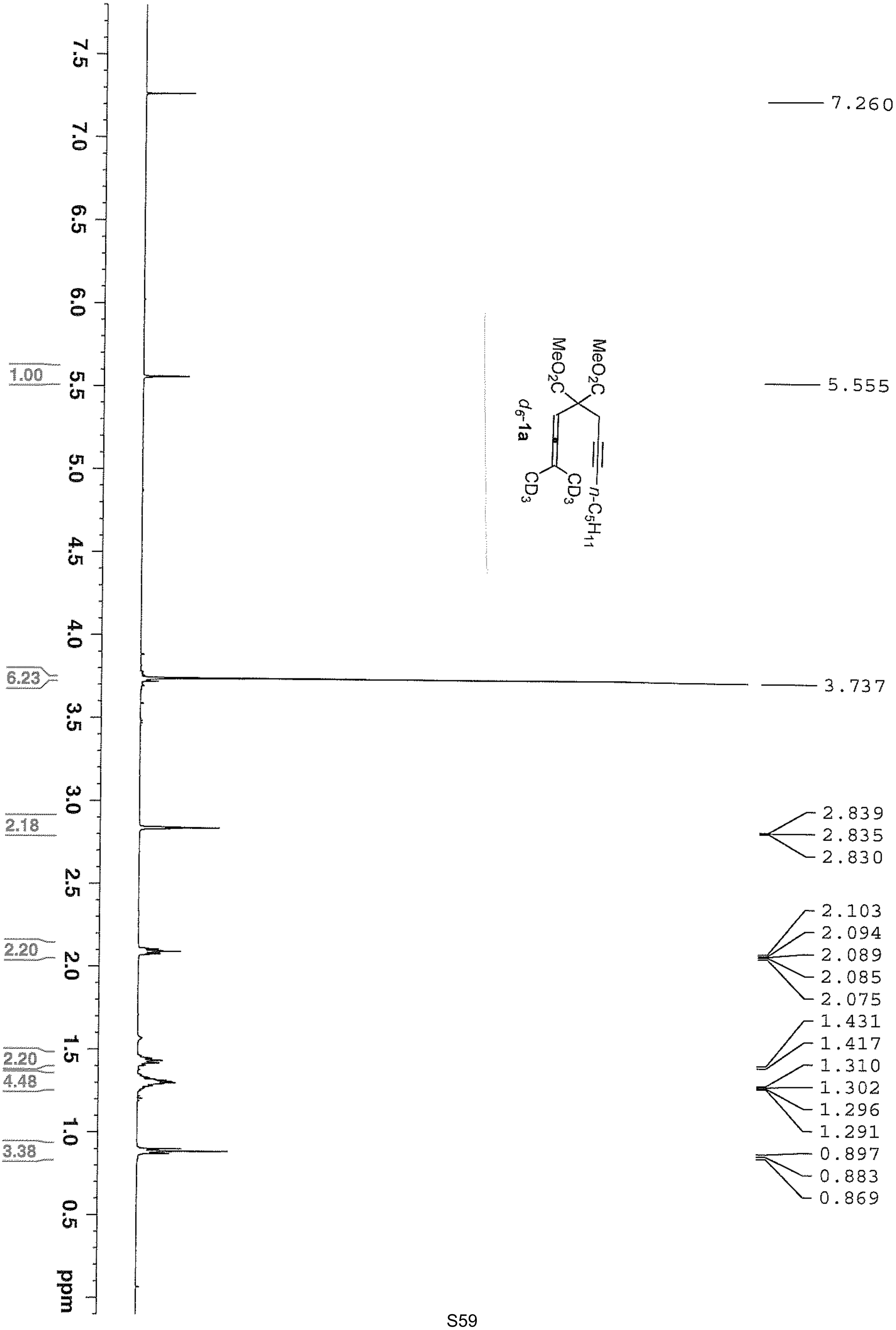

7.260
5.555
3.737
2.839
2.835
2.830
2.103
2.094
2.089
2.085
2.075
1.431
1.417
1.310
1.302
1.296
1.291
0.897
0.883
0.869
1.00
6.23
2.18
2.20
2.20
4.48
3.38
ppm
MeO2C
MeO2C
n-C5H11
CD3
CD3
d6-1a

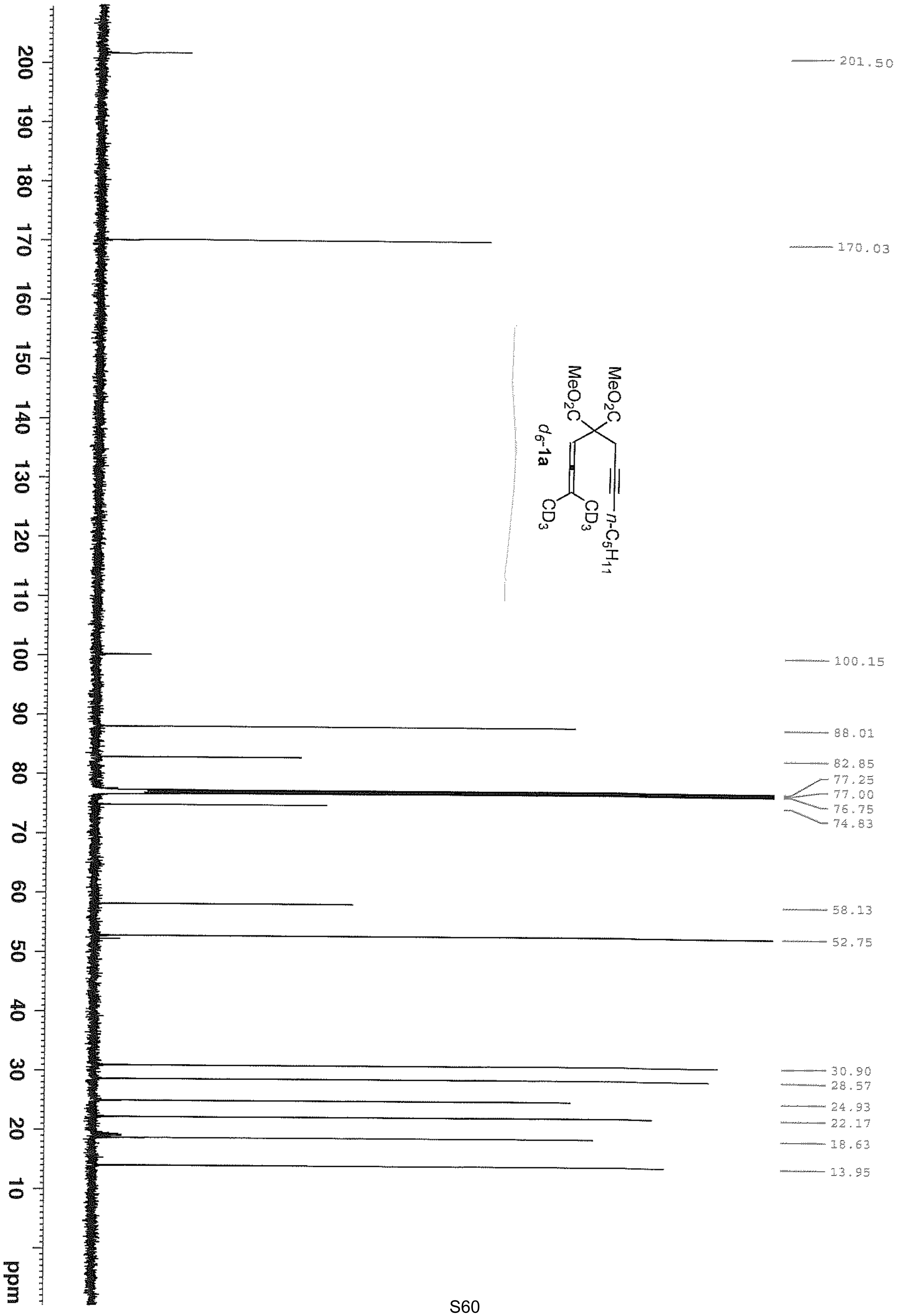
201.50
170.03
100.15
88.01
82.85
77.25
77.00
76.75
74.83
58.13
52.75
30.90
28.57
24.93
22.17
18.63
13.95
MeO2C
MeO2C
n-C5H11
CD3
CD3
d6-1a
ppm

# Mass Spectrum SmartFormula Report

**Analysis Info**

| | | | |
|---|---|---|---|
| Analysis Name | H:\Data2\Youqian\dyq-4-132000002.d | Acquisition Date | 2012-10-15 09:51:45 |
| Method | tune_wide_dirk.m | | |
| Sample Name | dyq-4-132 | Operator | pia |
| Comment | | Instrument / Ser# | micrOTOF 125 |

**Acquisition Parameter**

| | | | | | |
|---|---|---|---|---|---|
| Source Type | ESI | Ion Polarity | Positive | Set Nebulizer | 0.4 Bar |
| Focus | Not active | | | Set Dry Heater | 180 °C |
| Scan Begin | 50 m/z | Set Capillary | 4500 V | Set Dry Gas | 4.0 l/min |
| Scan End | 3000 m/z | Set End Plate Offset | -500 V | Set Divert Valve | Source |

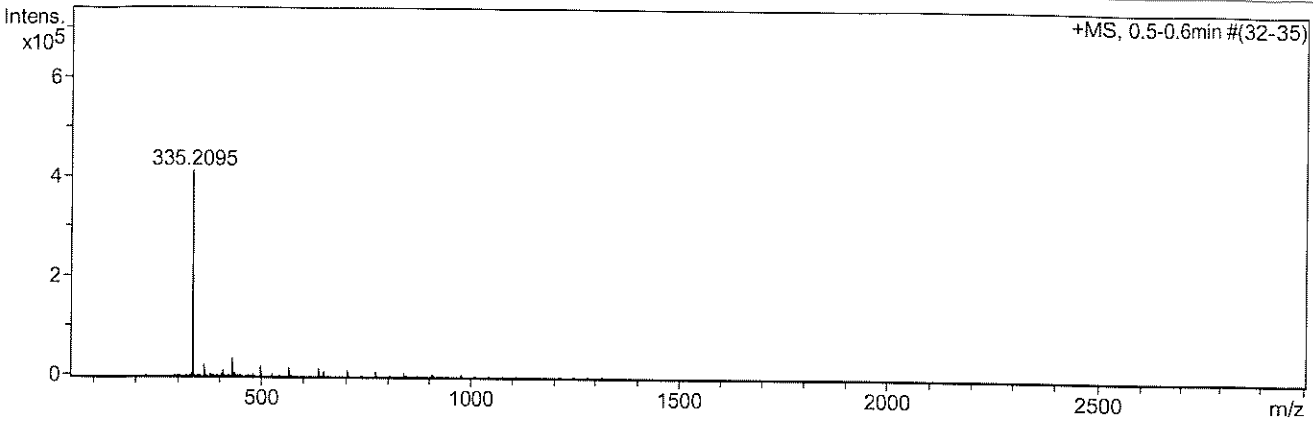


| Formula | Meas. m/z | m/z | err [ppm] | Mean err [ppm] |
|---|---|---|---|---|
| | 335.2095 | | | |
| C 18 H 20 D 6 Na O 4 | | 335.2100 | 1.6 | 0.7 |

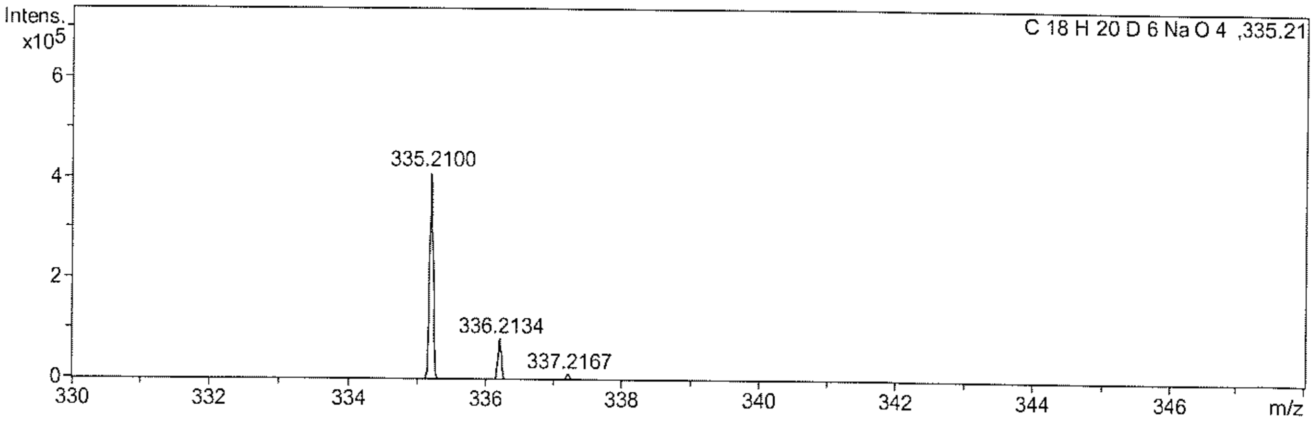


| Formula | Meas. m/z | m/z | err [ppm] | Mean err [ppm] |
|---|---|---|---|---|

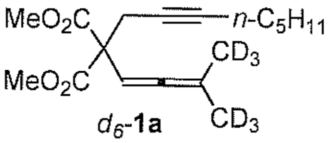


$d_6$-**1a**

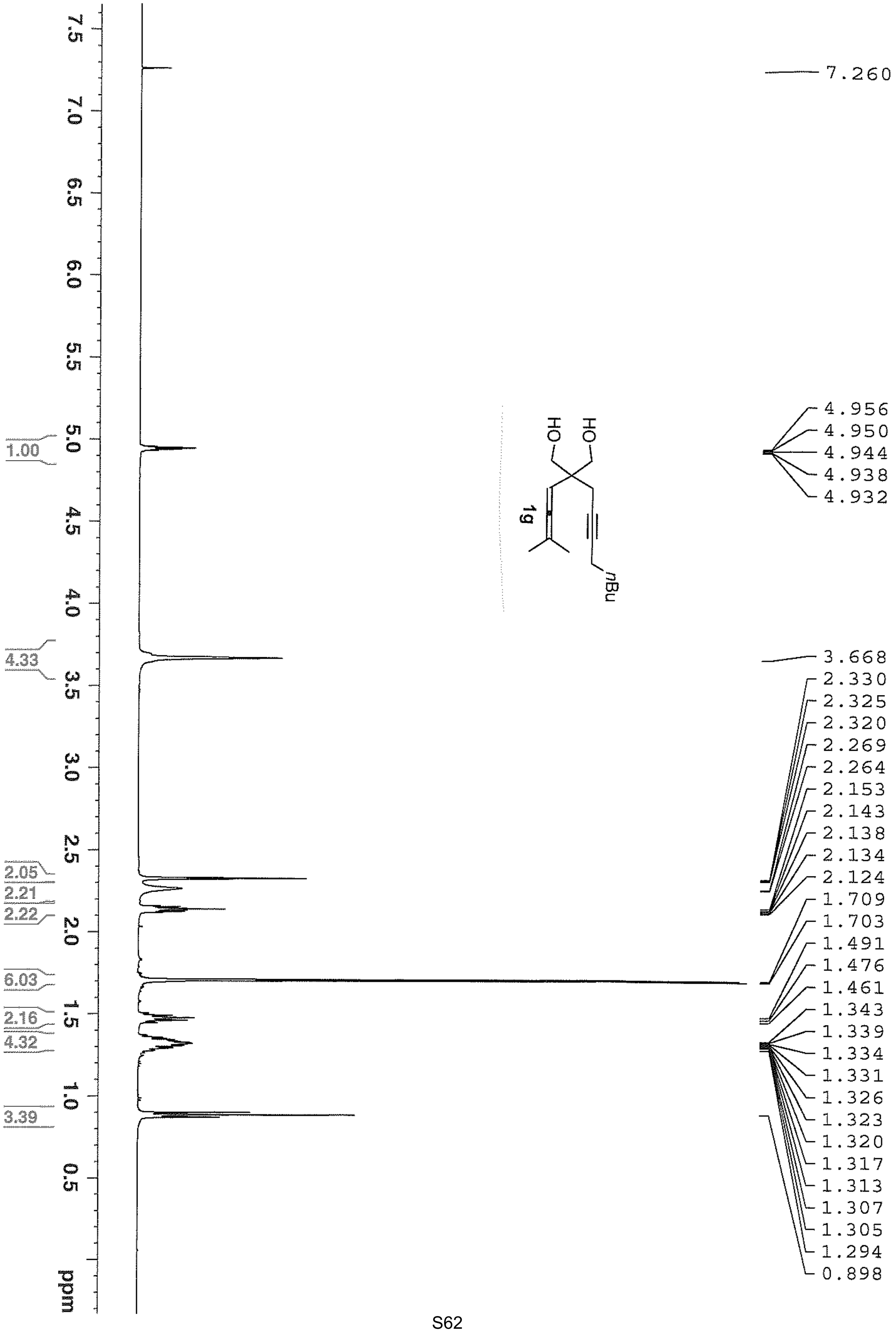
7.260
4.956
4.950
4.944
4.938
4.932
3.668
2.330
2.325
2.320
2.269
2.264
2.153
2.143
2.138
2.134
2.124
1.709
1.703
1.491
1.476
1.461
1.343
1.339
1.334
1.331
1.326
1.323
1.320
1.317
1.313
1.307
1.305
1.294
0.898
HO
HO
nBu
1g
1.00
4.33
2.05
2.21
2.22
6.03
2.16
4.32
3.39
7.5
7.0
6.5
6.0
5.5
5.0
4.5
4.0
3.5
3.0
2.5
2.0
1.5
1.0
0.5
ppm

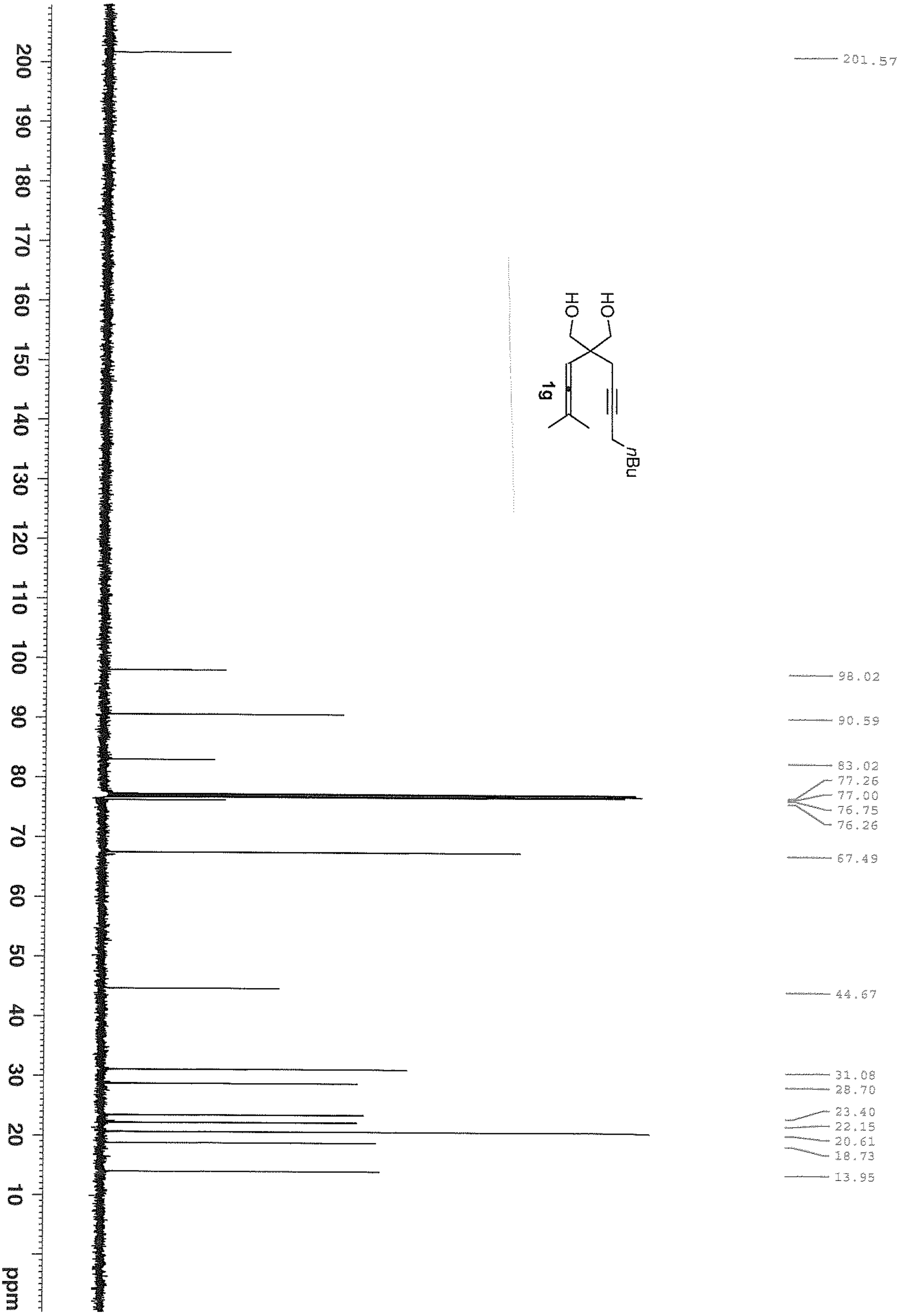
201.57
98.02
90.59
83.02
77.26
77.00
76.75
76.26
67.49
44.67
31.08
28.70
23.40
22.15
20.61
18.73
13.95
HO
HO
nBu
1g
ppm

# Mass Spectrum SmartFormula Report

**Analysis Info**

| | | | |
|---|---|---|---|
| Analysis Name | E:\Data2\Youqian\Teb-c-54000001.d | Acquisition Date | 2012-12-07 18:23:37 |
| Method | Tune_low_pos.m | Operator | Carin Larsson |
| Sample Name | Teb-c-54 | Instrument / Ser# | micrOTOF 125 |
| Comment | | | |

**Acquisition Parameter**

| | | | | | |
|---|---|---|---|---|---|
| Source Type | ESI | Ion Polarity | Positive | Set Nebulizer | 0.4 Bar |
| Focus | Not active | | | Set Dry Heater | 180 °C |
| Scan Begin | 50 m/z | Set Capillary | 4000 V | Set Dry Gas | 4.0 l/min |
| Scan End | 1000 m/z | Set End Plate Offset | -500 V | Set Divert Valve | Source |

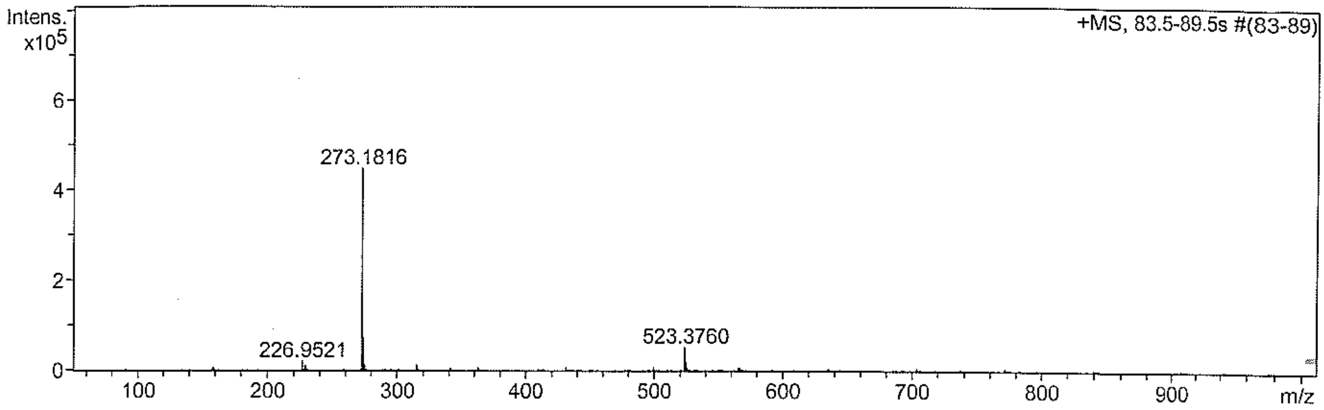


| Formula | Meas. m/z | m/z | err [ppm] | Mean err [ppm] |
|---|---|---|---|---|
| C 16 H 26 Na O 2 | 273.1816 | 273.1825 | 3.4 | 3.2 |

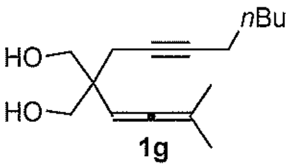


**1g**

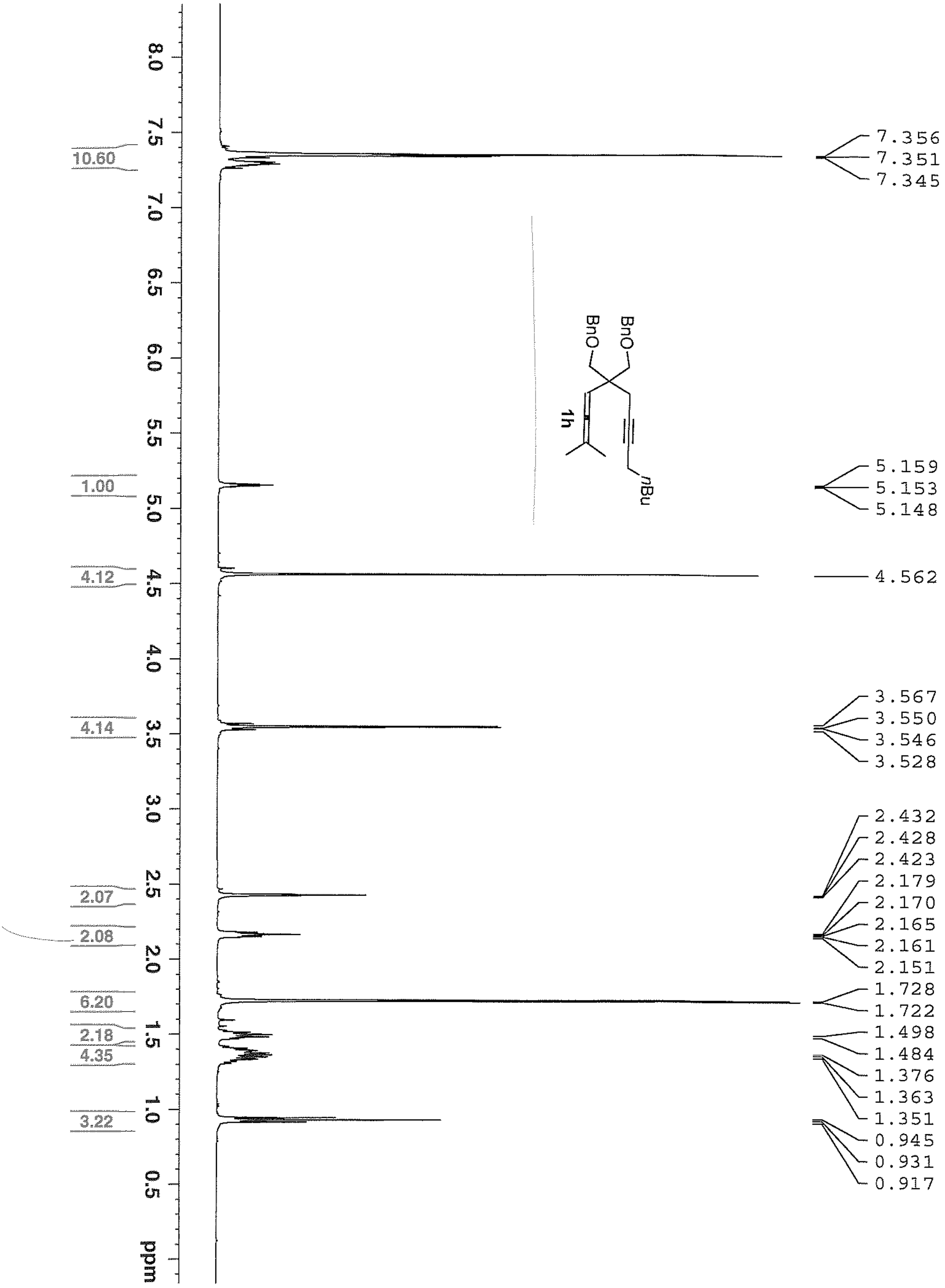
7.356
7.351
7.345
5.159
5.153
5.148
4.562
3.567
3.550
3.546
3.528
2.432
2.428
2.423
2.179
2.170
2.165
2.161
2.151
1.728
1.722
1.498
1.484
1.376
1.363
1.351
0.945
0.931
0.917
10.60
1.00
4.12
4.14
2.07
2.08
6.20
2.18
4.35
3.22
8.0
7.5
7.0
6.5
6.0
5.5
5.0
4.5
4.0
3.5
3.0
2.5
2.0
1.5
1.0
0.5
ppm
BnO
BnO
nBu
1h

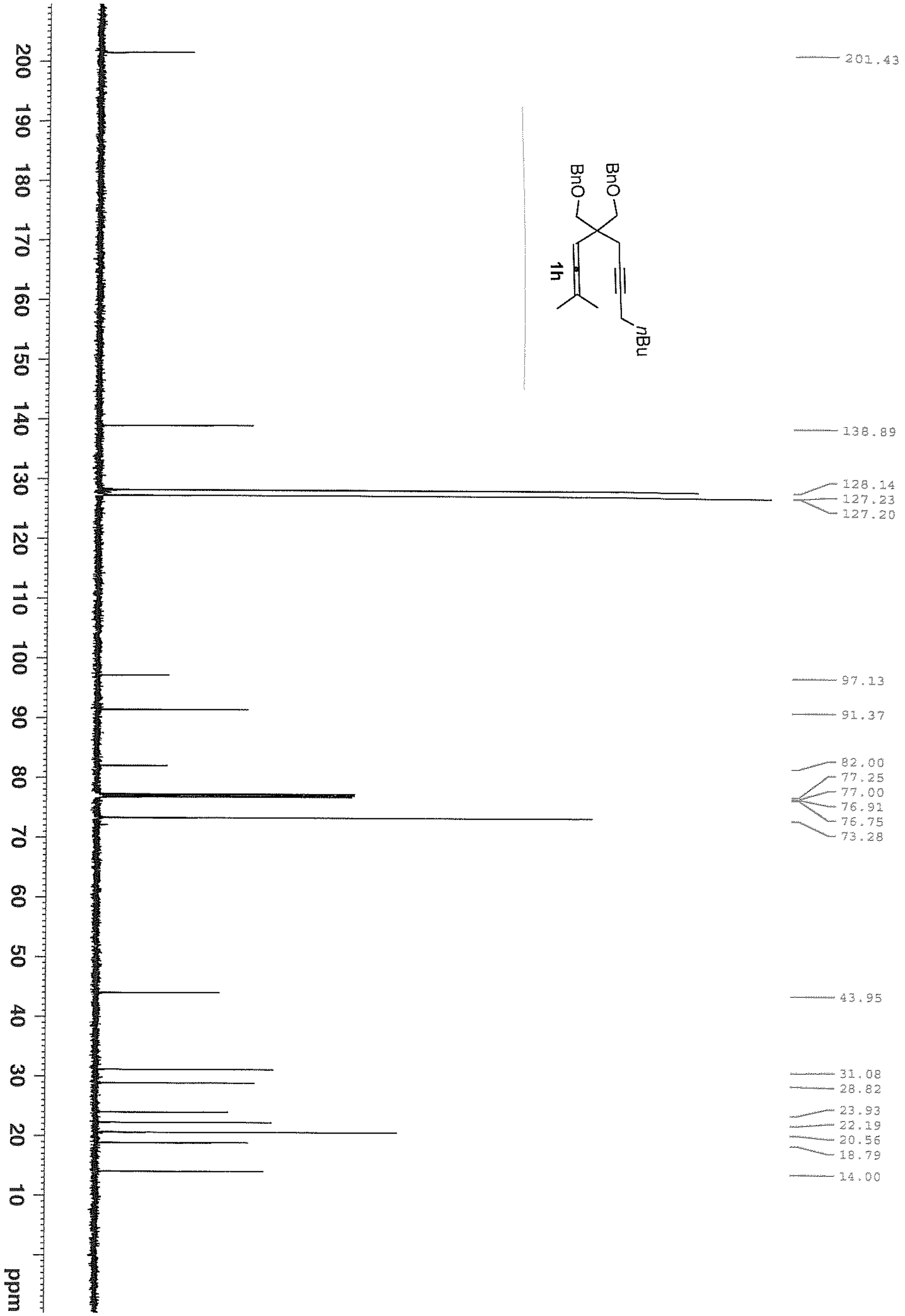

201.43
138.89
128.14
127.23
127.20
97.13
91.37
82.00
77.25
77.00
76.91
76.75
73.28
43.95
31.08
28.82
23.93
22.19
20.56
18.79
14.00
BnO
BnO
nBu
1h
200 190 180 170 160 150 140 130 120 110 100 90 80 70 60 50 40 30 20 10
ppm

# Mass Spectrum SmartFormula Report

**Analysis Info**

| | | | |
|---|---|---|---|
| Analysis Name | E:\Data2\Youqian\dyq-4-198000001.d | Acquisition Date | 2012-12-10 22:32:14 |
| Method | Tune_wide_pos.m | Operator | Carin Larsson |
| Sample Name | dyq-4-198 | Instrument / Ser# | micrOTOF 125 |
| Comment | | | |

**Acquisition Parameter**

| | | | | | |
|---|---|---|---|---|---|
| Source Type | ESI | Ion Polarity | Positive | Set Nebulizer | 0.3 Bar |
| Focus | Not active | | | Set Dry Heater | 180 °C |
| Scan Begin | 50 m/z | Set Capillary | 4500 V | Set Dry Gas | 4.0 l/min |
| Scan End | 3000 m/z | Set End Plate Offset | -500 V | Set Divert Valve | Source |

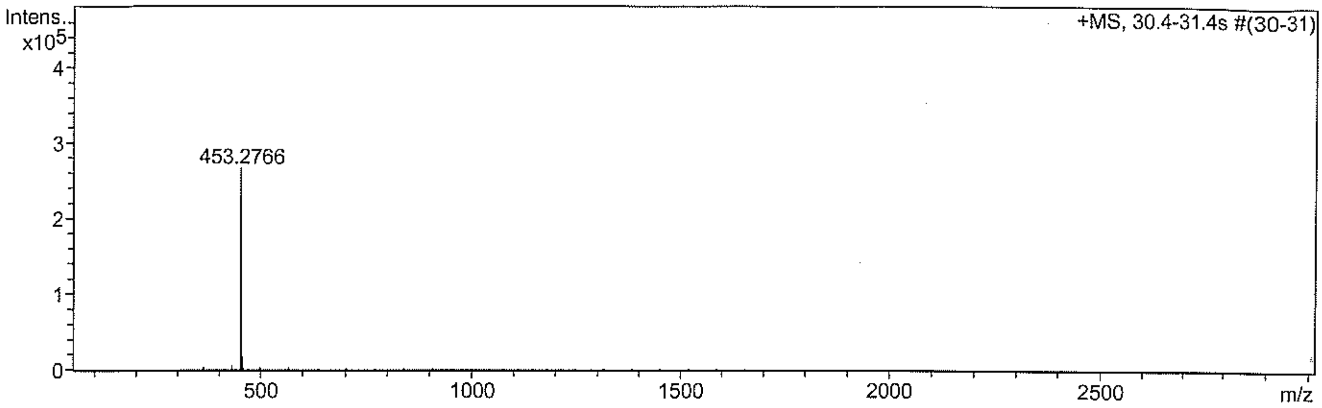


| Formula | Meas. m/z | m/z | err [ppm] | Mean err [ppm] |
|---|---|---|---|---|
| C 30 H 38 Na O 2 | 453.2766 | 453.2764 | -0.5 | -0.4 |

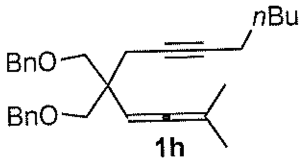


**1h**

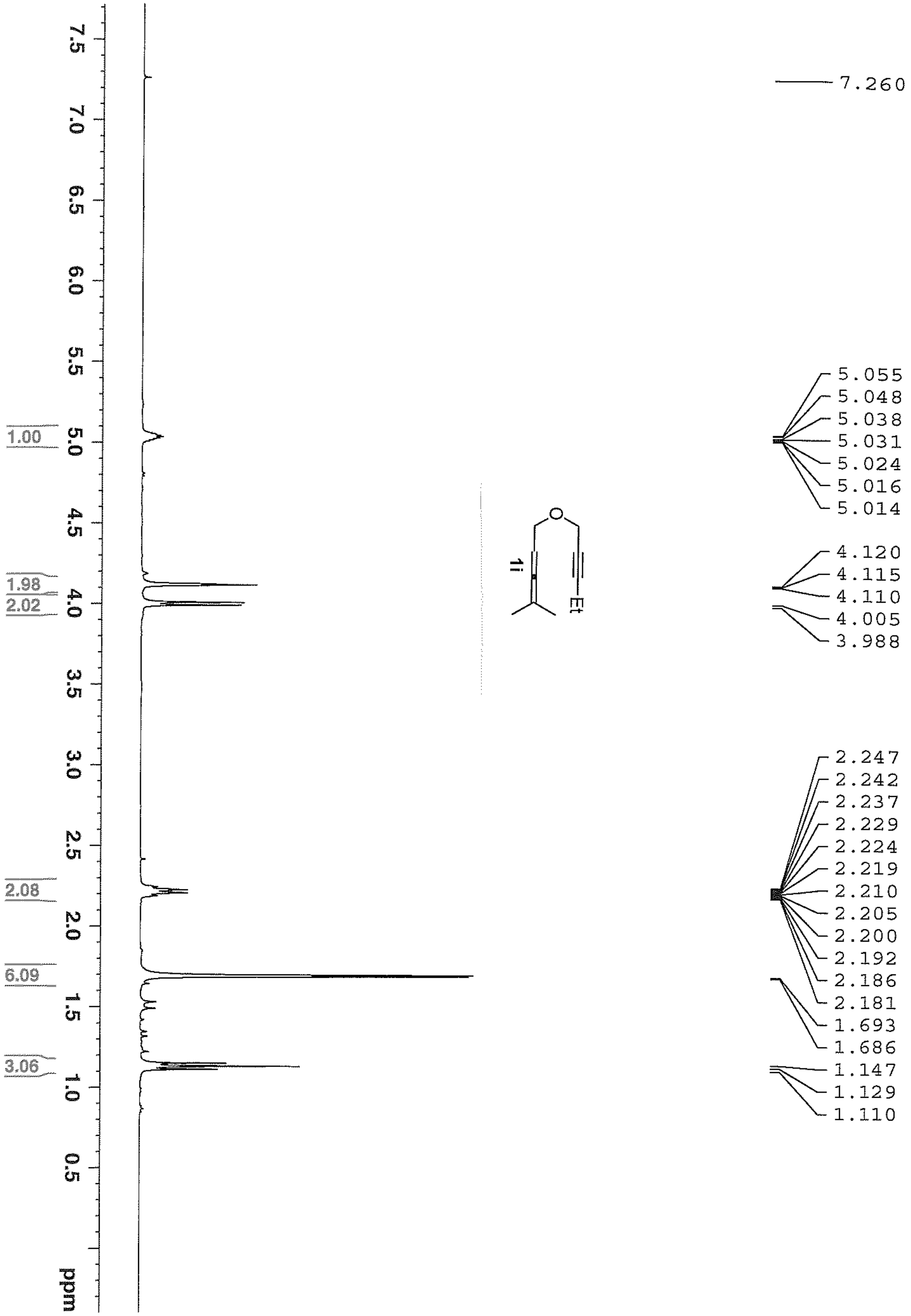
7.260
5.055
5.048
5.038
5.031
5.024
5.016
5.014
4.120
4.115
4.110
4.005
3.988
2.247
2.242
2.237
2.229
2.224
2.219
2.210
2.205
2.200
2.192
2.186
2.181
1.693
1.686
1.147
1.129
1.110
1.00
1.98
2.02
2.08
6.09
3.06
7.5
7.0
6.5
6.0
5.5
5.0
4.5
4.0
3.5
3.0
2.5
2.0
1.5
1.0
0.5
ppm
O
Et
1i

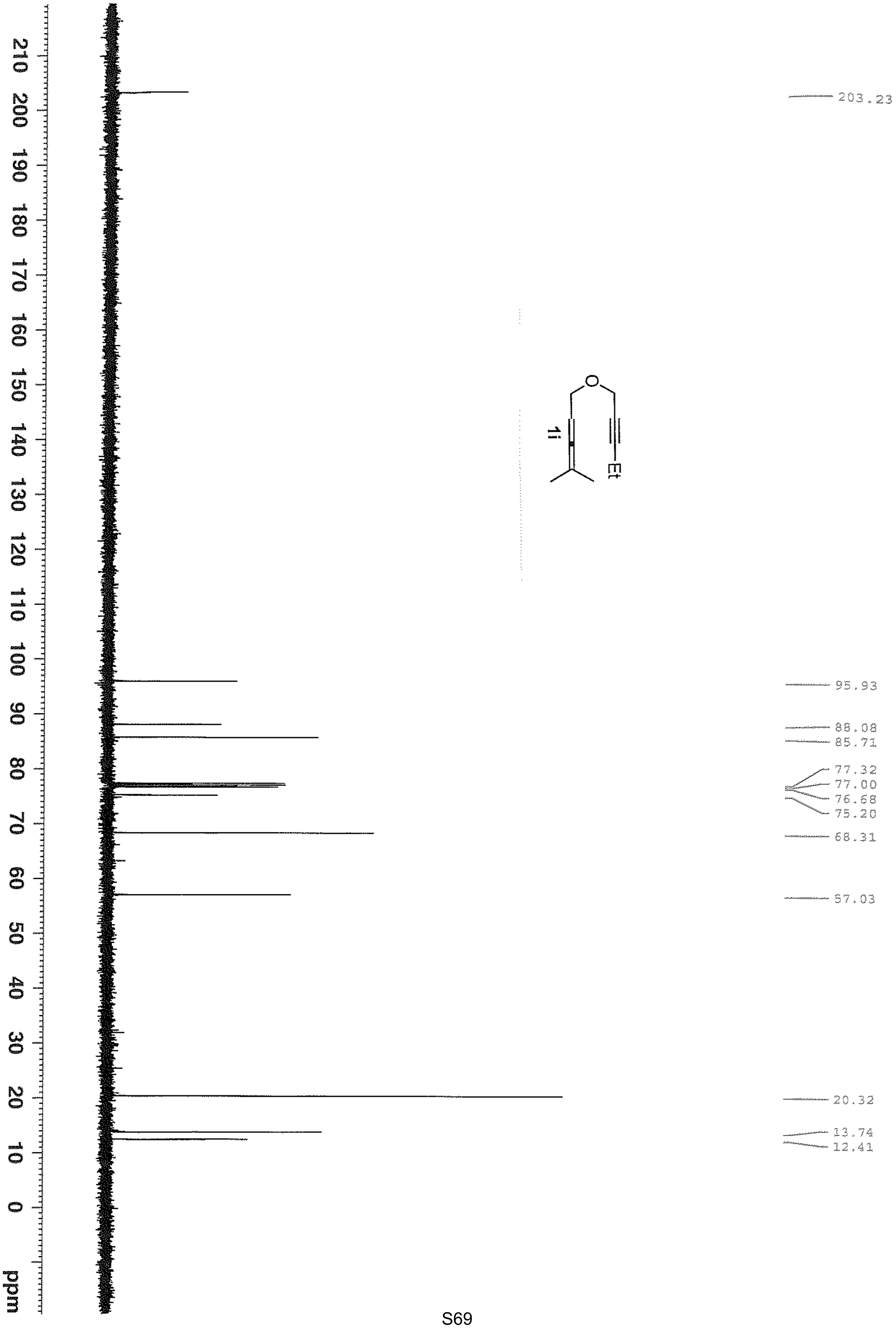

203.23
95.93
88.08
85.71
77.32
77.00
76.68
75.20
68.31
57.03
20.32
13.74
12.41
O
Et
1i
ppm

# Mass Spectrum SmartFormula Report

**Analysis Info**

| | | | |
|---|---|---|---|
| Analysis Name | H:\Data2\Youqian\dyq-3-81000003.d | Acquisition Date | 2012-01-11 10:42:14 |
| Method | tune_low_dirk.m | Operator | pia |
| Sample Name | dyq-3-81 | Instrument / Ser# | micrOTOF 125 |
| Comment | | | |

**Acquisition Parameter**

| | | | | | |
|---|---|---|---|---|---|
| Source Type | ESI | Ion Polarity | Positive | Set Nebulizer | 0.4 Bar |
| Focus | Not active | | | Set Dry Heater | 170 °C |
| Scan Begin | 50 m/z | Set Capillary | 4500 V | Set Dry Gas | 4.0 l/min |
| Scan End | 3000 m/z | Set End Plate Offset | -500 V | Set Divert Valve | Source |

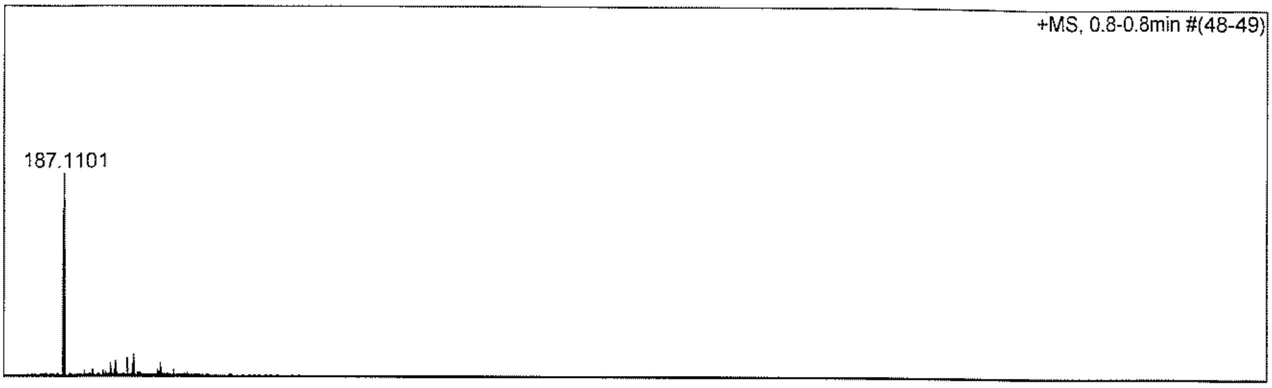


| Formula | Meas. m/z | m/z | err [ppm] | Mean err [ppm] |
|---|---|---|---|---|
| | 187.1101 | | | |
| C 11 H 16 Na O | | 187.1093 | -4.2 | -4.3 |

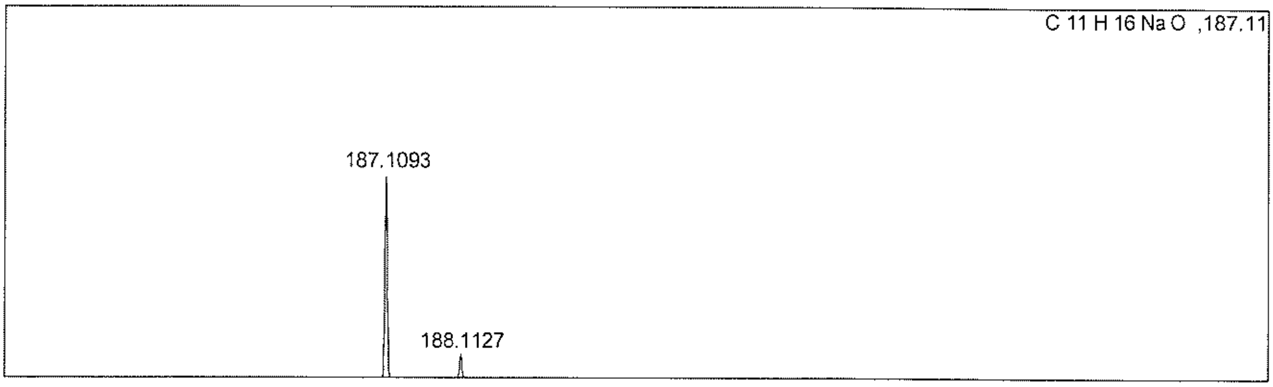


| Formula | Meas. m/z | m/z | err [ppm] | Mean err [ppm] |
|---|---|---|---|---|

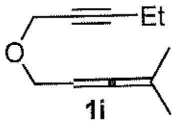


**1i**

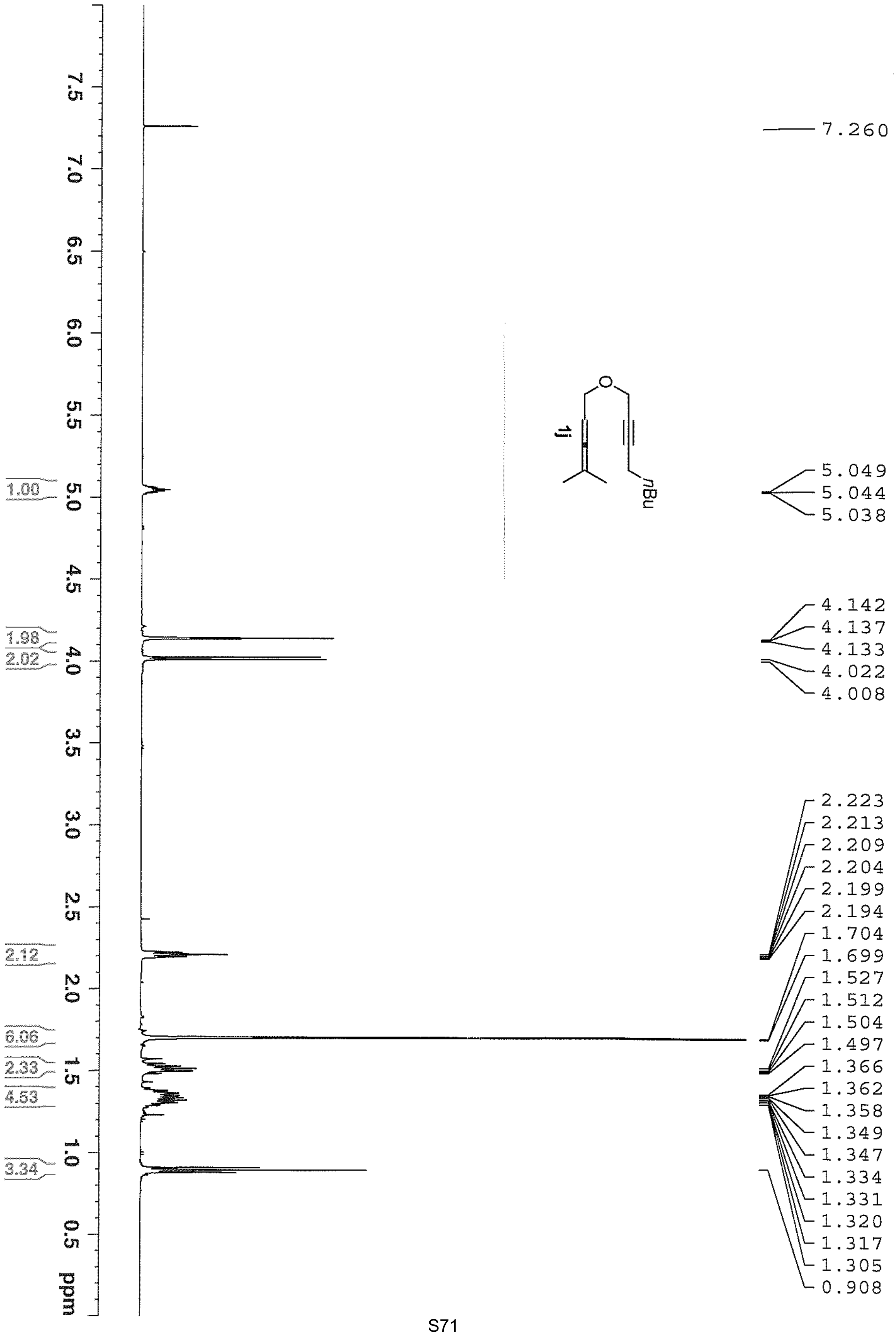
1j
nBu
7.260
5.049
5.044
5.038
4.142
4.137
4.133
4.022
4.008
2.223
2.213
2.209
2.204
2.199
2.194
1.704
1.699
1.527
1.512
1.504
1.497
1.366
1.362
1.358
1.349
1.347
1.334
1.331
1.320
1.317
1.305
0.908
1.00
1.98
2.02
2.12
6.06
2.33
4.53
3.34
7.5
7.0
6.5
6.0
5.5
5.0
4.5
4.0
3.5
3.0
2.5
2.0
1.5
1.0
0.5
ppm

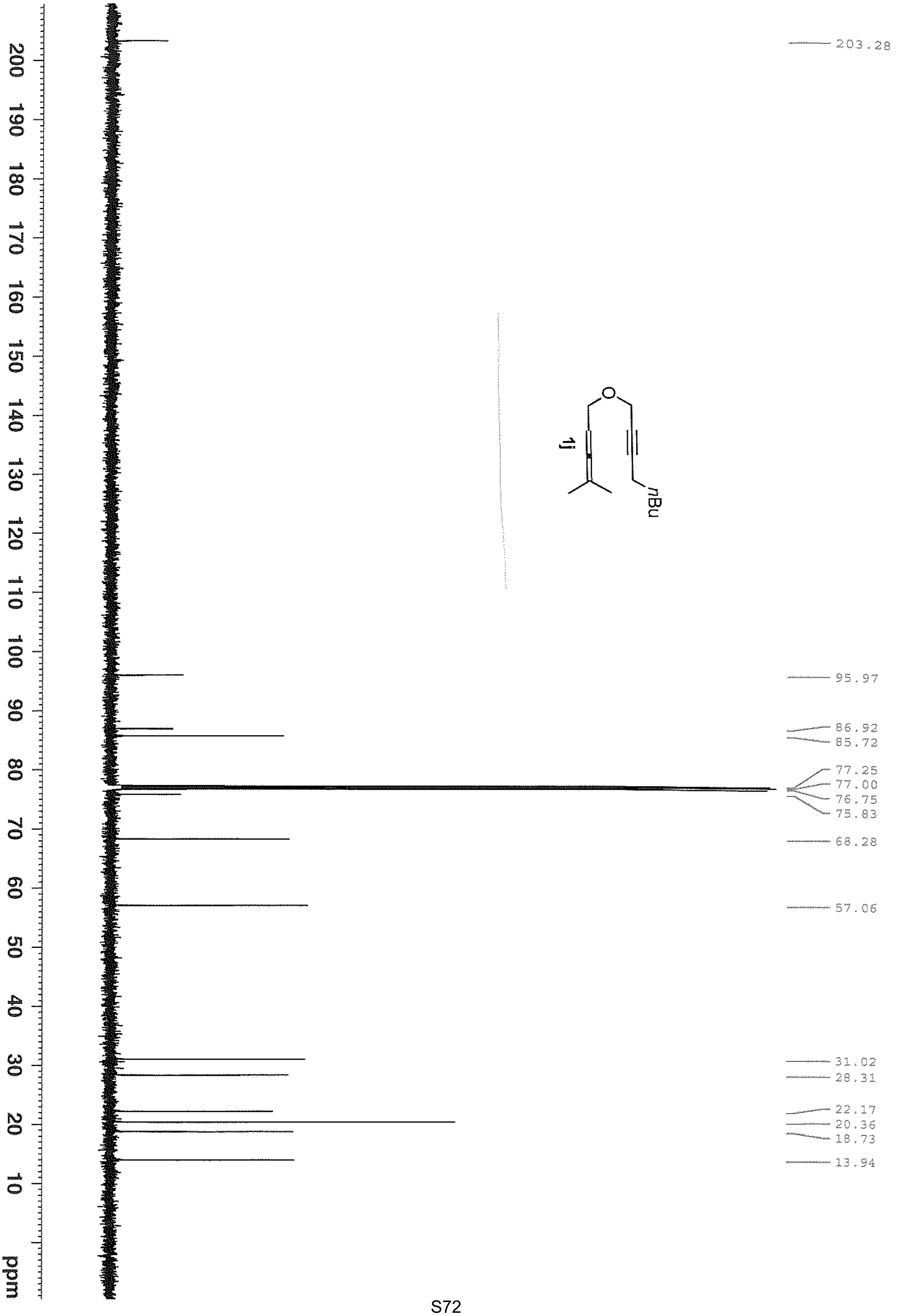

203.28
95.97
86.92
85.72
77.25
77.00
76.75
75.83
68.28
57.06
31.02
28.31
22.17
20.36
18.73
13.94
1j
nBu
ppm

# Mass Spectrum SmartFormula Report

**Analysis Info**

| | | | |
|---|---|---|---|
| Analysis Name | E:\Data2\Youqian\dyq-4-189000001.d | Acquisition Date | 2012-12-07 18:18:32 |
| Method | Tune_low_pos.m | Operator | Carin Larsson |
| Sample Name | dyq-4-189 | Instrument / Ser# | micrOTOF 125 |
| Comment | | | |

**Acquisition Parameter**

| | | | | | |
|---|---|---|---|---|---|
| Source Type | ESI | Ion Polarity | Positive | Set Nebulizer | 0.4 Bar |
| Focus | Not active | | | Set Dry Heater | 180 °C |
| Scan Begin | 50 m/z | Set Capillary | 4000 V | Set Dry Gas | 4.0 l/min |
| Scan End | 1000 m/z | Set End Plate Offset | -500 V | Set Divert Valve | Source |

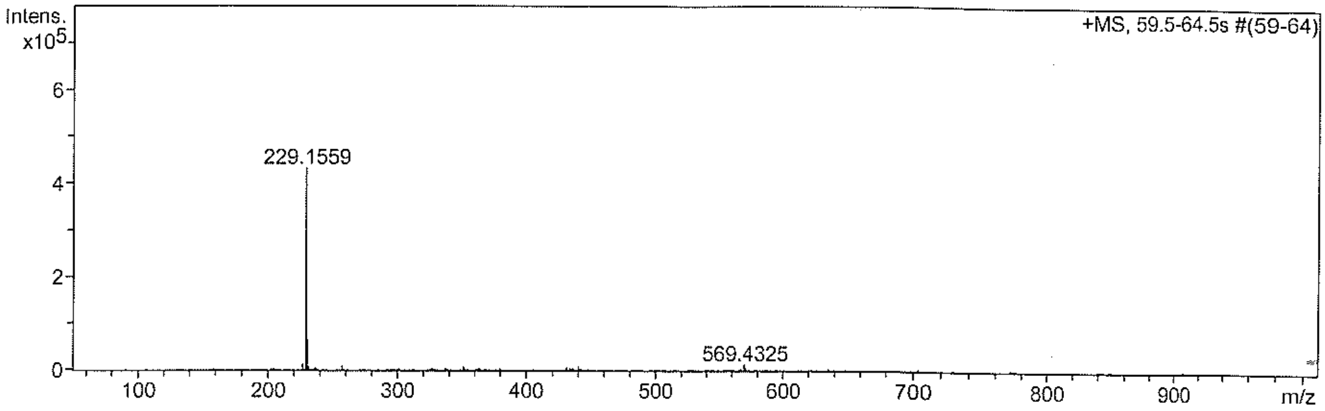


| Formula | Meas. m/z | m/z | err [ppm] | Mean err [ppm] |
|---|---|---|---|---|
| C 14 H 22 Na O | 229.1559 | 229.1563 | 1.8 | 1.7 |

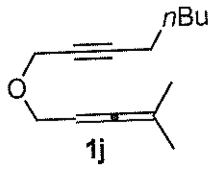


**1j**

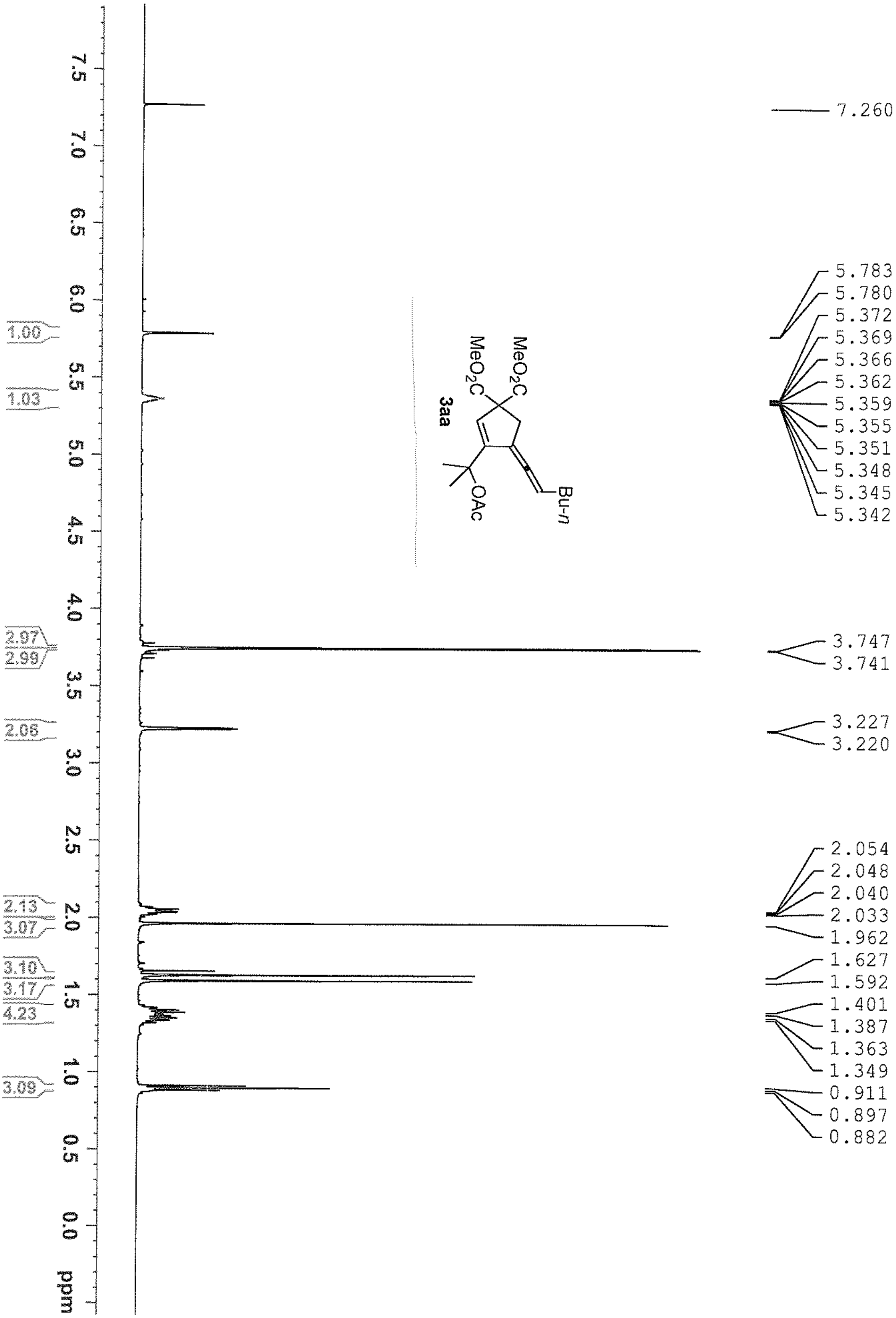
3aa
MeO2C
MeO2C
Bu-n
OAc
7.260
5.783
5.780
5.372
5.369
5.366
5.362
5.359
5.355
5.351
5.348
5.345
5.342
3.747
3.741
3.227
3.220
2.054
2.048
2.040
2.033
1.962
1.627
1.592
1.401
1.387
1.363
1.349
0.911
0.897
0.882
1.00
1.03
2.97
2.99
2.06
2.13
3.07
3.10
3.17
4.23
3.09
7.5
7.0
6.5
6.0
5.5
5.0
4.5
4.0
3.5
3.0
2.5
2.0
1.5
1.0
0.5
0.0
ppm

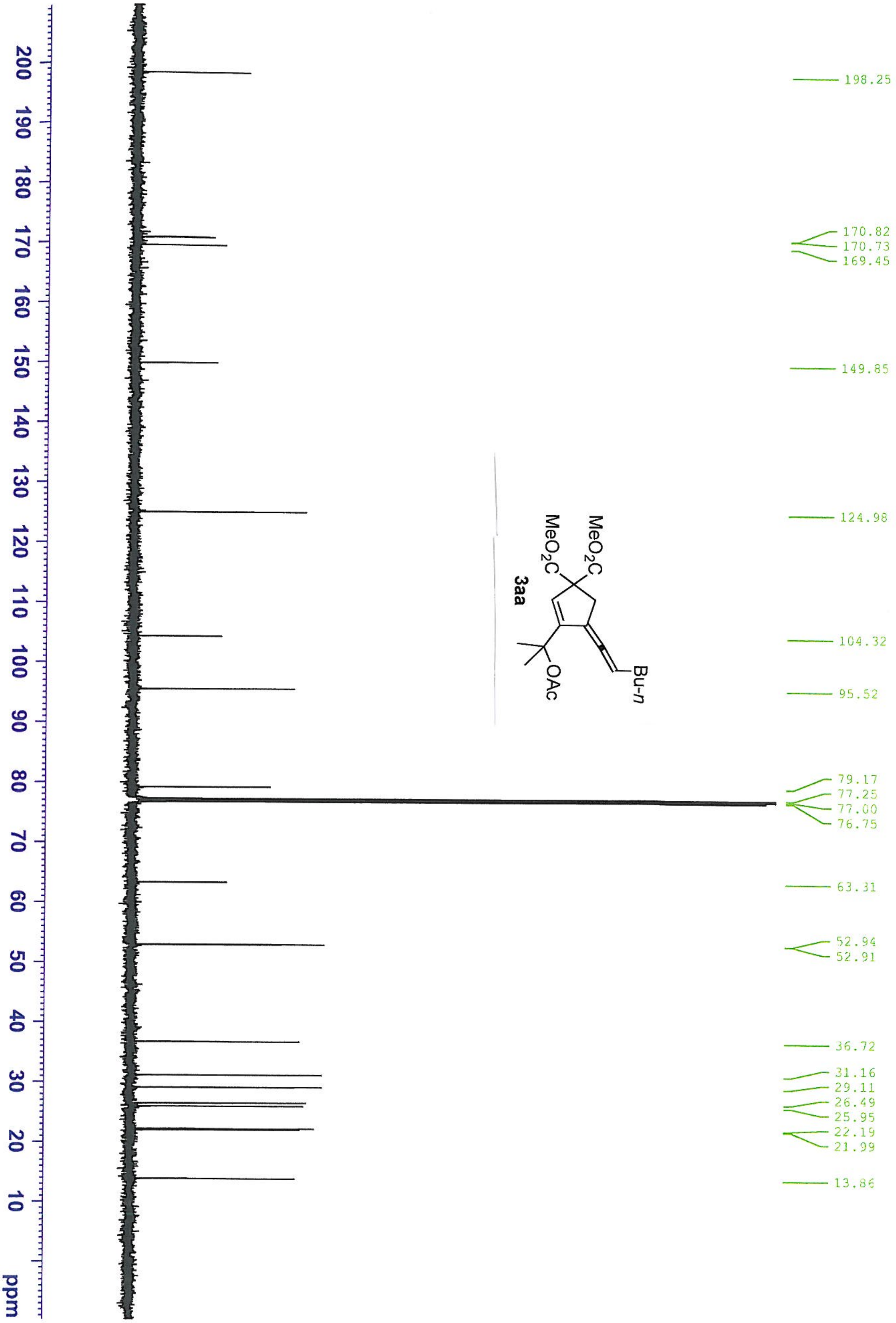

198.25
170.82
170.73
169.45
149.85
124.98
104.32
95.52
79.17
77.25
77.00
76.75
63.31
52.94
52.91
36.72
31.16
29.11
26.49
25.95
22.19
21.99
13.86
MeO2C
MeO2C
Bu-n
OAc
3aa
200
190
180
170
160
150
140
130
120
110
100
90
80
70
60
50
40
30
20
10
ppm

# Mass Spectrum SmartFormula Report

**Analysis Info**

| | | | |
|---|---|---|---|
| Analysis Name | H:\Data2\Youqian\dyq-3-165000001.d | Acquisition Date | 2012-04-23 12:03:38 |
| Method | tune_wide_dirk.m | Operator | pia |
| Sample Name | dyq-3-165 | Instrument / Ser# | micrOTOF 125 |
| Comment | | | |

**Acquisition Parameter**

| | | | | | |
|---|---|---|---|---|---|
| Source Type | ESI | Ion Polarity | Positive | Set Nebulizer | 0.4 Bar |
| Focus | Not active | | | Set Dry Heater | 180 °C |
| Scan Begin | 50 m/z | Set Capillary | 4500 V | Set Dry Gas | 4.0 l/min |
| Scan End | 3000 m/z | Set End Plate Offset | -500 V | Set Divert Valve | Source |

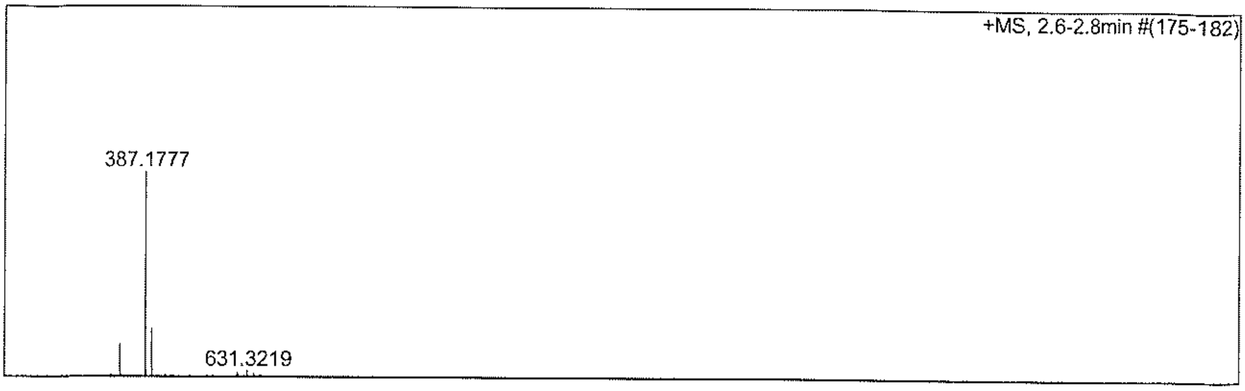


| Formula | Meas. m/z | m/z | err [ppm] | Mean err [ppm] |
|---|---|---|---|---|
| | 387.1777 | | | |
| C 20 H 28 Na O 6 | | 387.1778 | 0.3 | 0.8 |

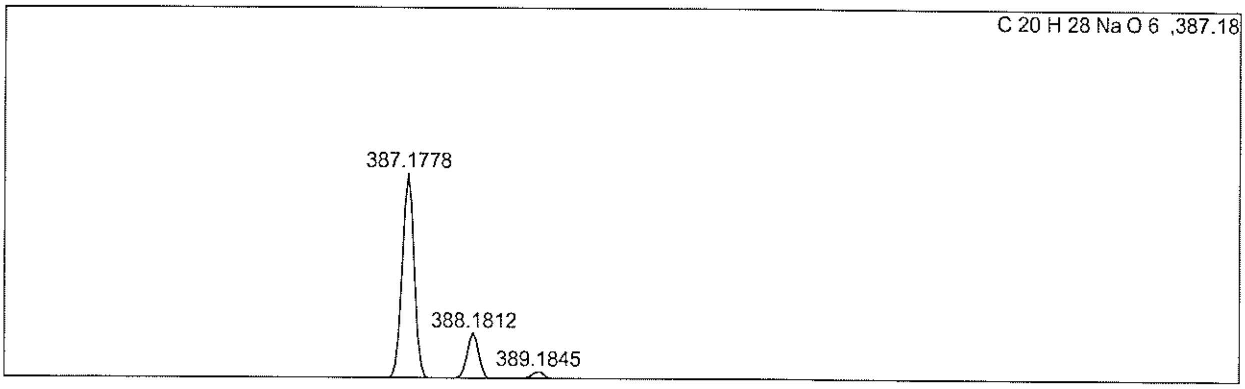


| Formula | Meas. m/z | m/z | err [ppm] | Mean err [ppm] |
|---|---|---|---|---|

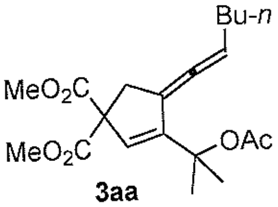


**3aa**

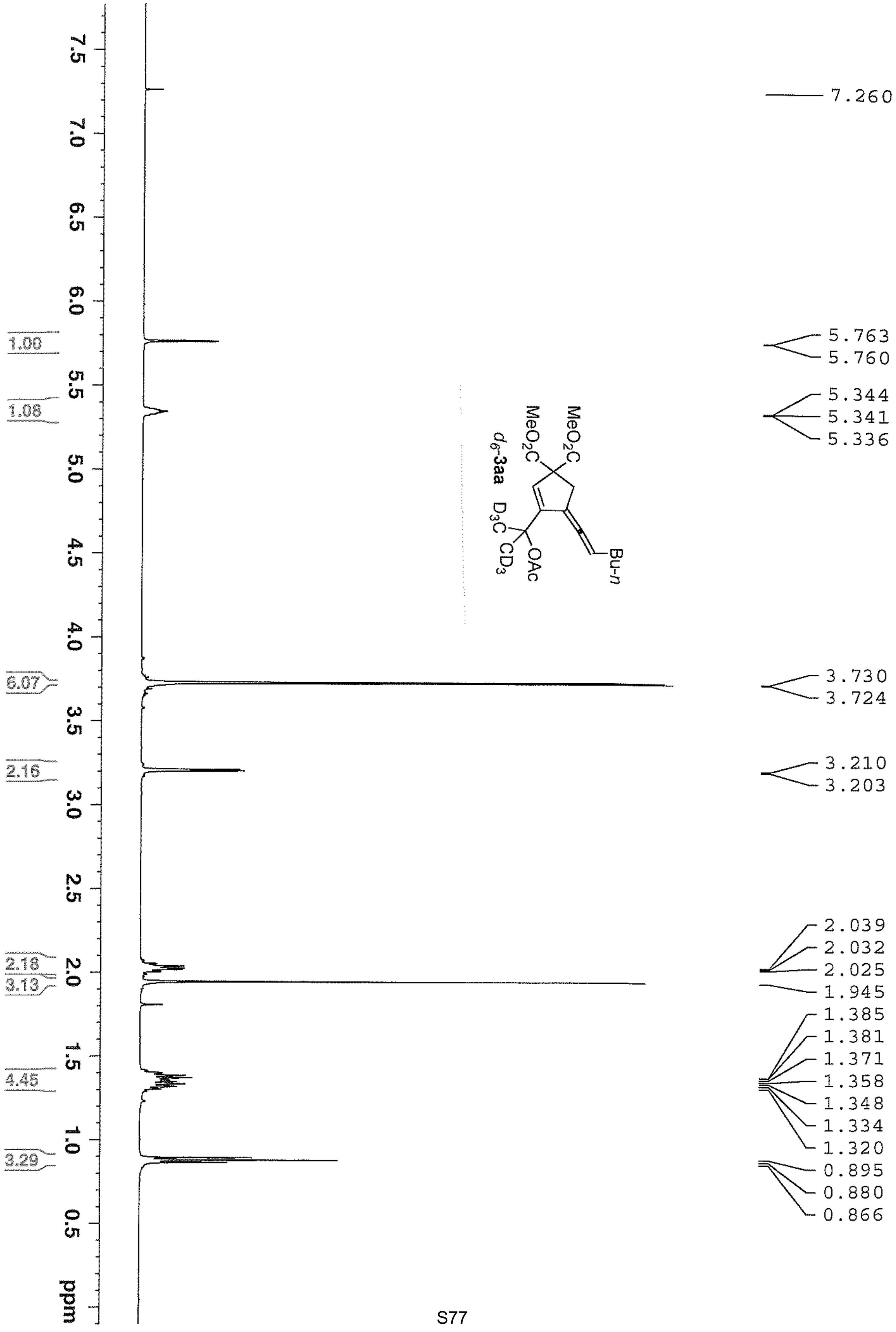

7.260
5.763
5.760
5.344
5.341
5.336
3.730
3.724
3.210
3.203
2.039
2.032
2.025
1.945
1.385
1.381
1.371
1.358
1.348
1.334
1.320
0.895
0.880
0.866
1.00
1.08
6.07
2.16
2.18
3.13
4.45
3.29
7.5
7.0
6.5
6.0
5.5
5.0
4.5
4.0
3.5
3.0
2.5
2.0
1.5
1.0
0.5
ppm
MeO2C
MeO2C
OAc
Bu-n
D3C
CD3
d6-3aa

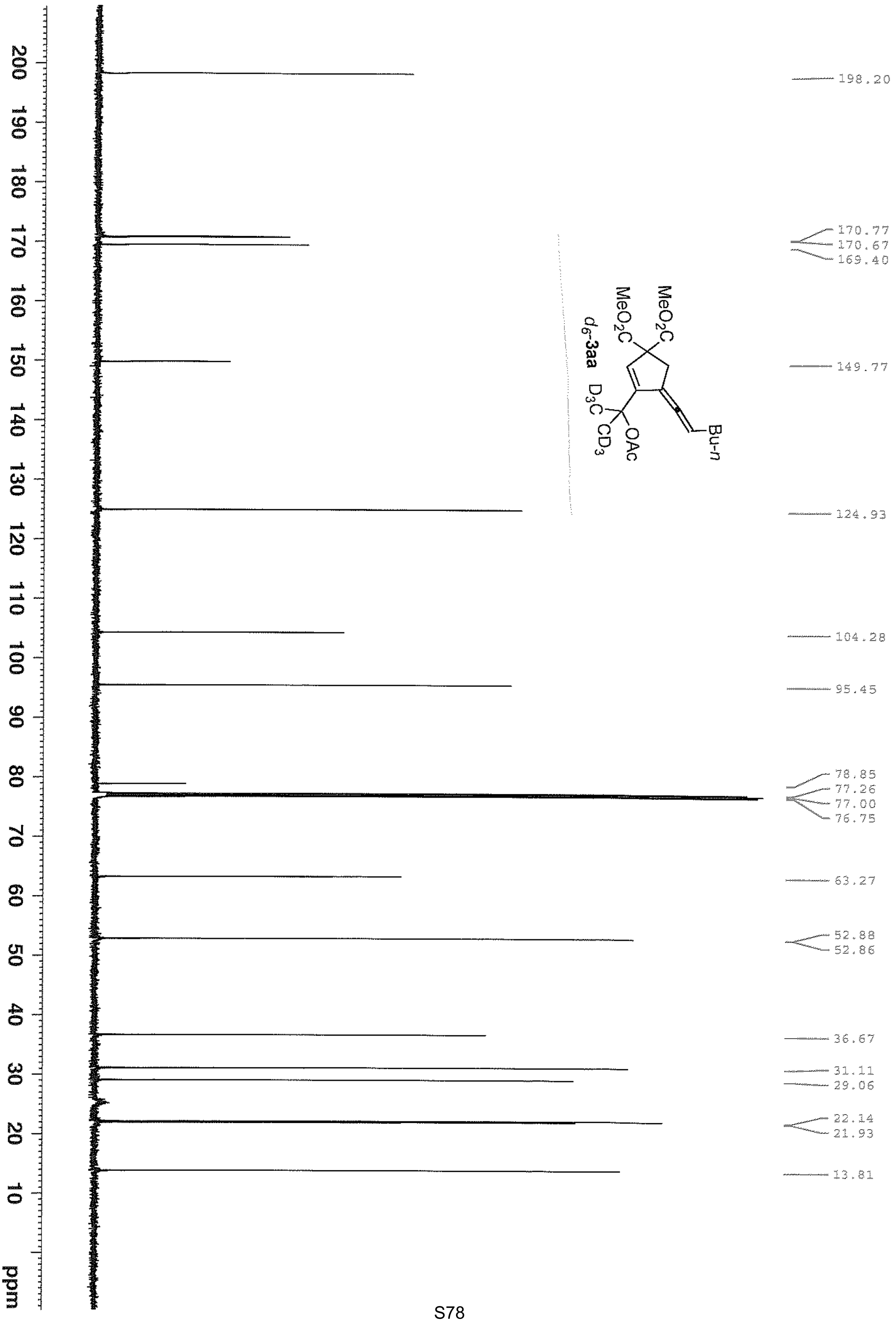
MeO2C
MeO2C
OAc
Bu-n
D3C
CD3
d6-3aa
198.20
170.77
170.67
169.40
149.77
124.93
104.28
95.45
78.85
77.26
77.00
76.75
63.27
52.88
52.86
36.67
31.11
29.06
22.14
21.93
13.81
200
190
180
170
160
150
140
130
120
110
100
90
80
70
60
50
40
30
20
10
ppm

# Mass Spectrum SmartFormula Report

**Analysis Info**

| | | | |
|---|---|---|---|
| Analysis Name | H:\Data2\Youqian\dyq-4-137000001.d | Acquisition Date | 2012-10-15 09:54:19 |
| Method | tune_wide_dirk.m | Operator | pia |
| Sample Name | dyq-4-137 | Instrument / Ser# | micrOTOF 125 |
| Comment | | | |

**Acquisition Parameter**

| | | | | | |
|---|---|---|---|---|---|
| Source Type | ESI | Ion Polarity | Positive | Set Nebulizer | 0.4 Bar |
| Focus | Not active | | | Set Dry Heater | 180 °C |
| Scan Begin | 50 m/z | Set Capillary | 4500 V | Set Dry Gas | 4.0 l/min |
| Scan End | 3000 m/z | Set End Plate Offset | -500 V | Set Divert Valve | Source |

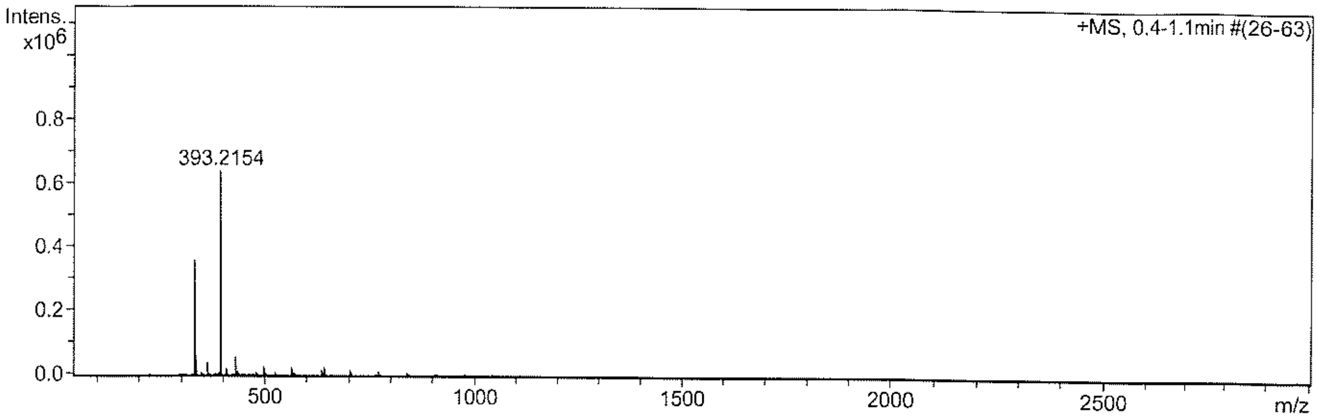


| Formula | Meas. m/z | m/z | err [ppm] | Mean err [ppm] |
|---|---|---|---|---|
| | 393.2154 | | | |
| C 20 H 22 D 6 Na O 6 | | 393.2155 | 0.2 | 0.3 |

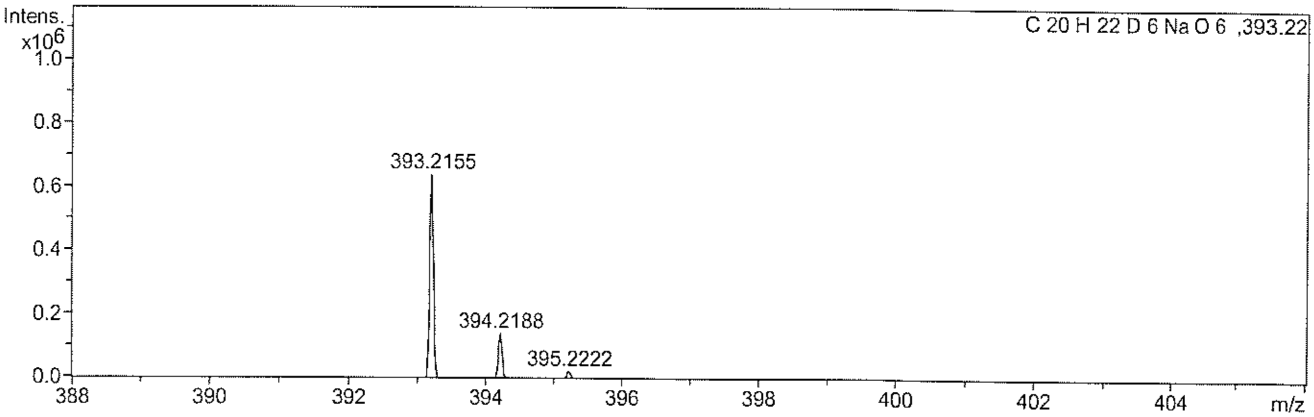


| Formula | Meas. m/z | m/z | err [ppm] | Mean err [ppm] |
|---|---|---|---|---|

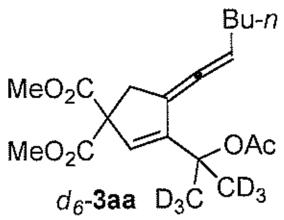


*d*$_6$-**3aa**

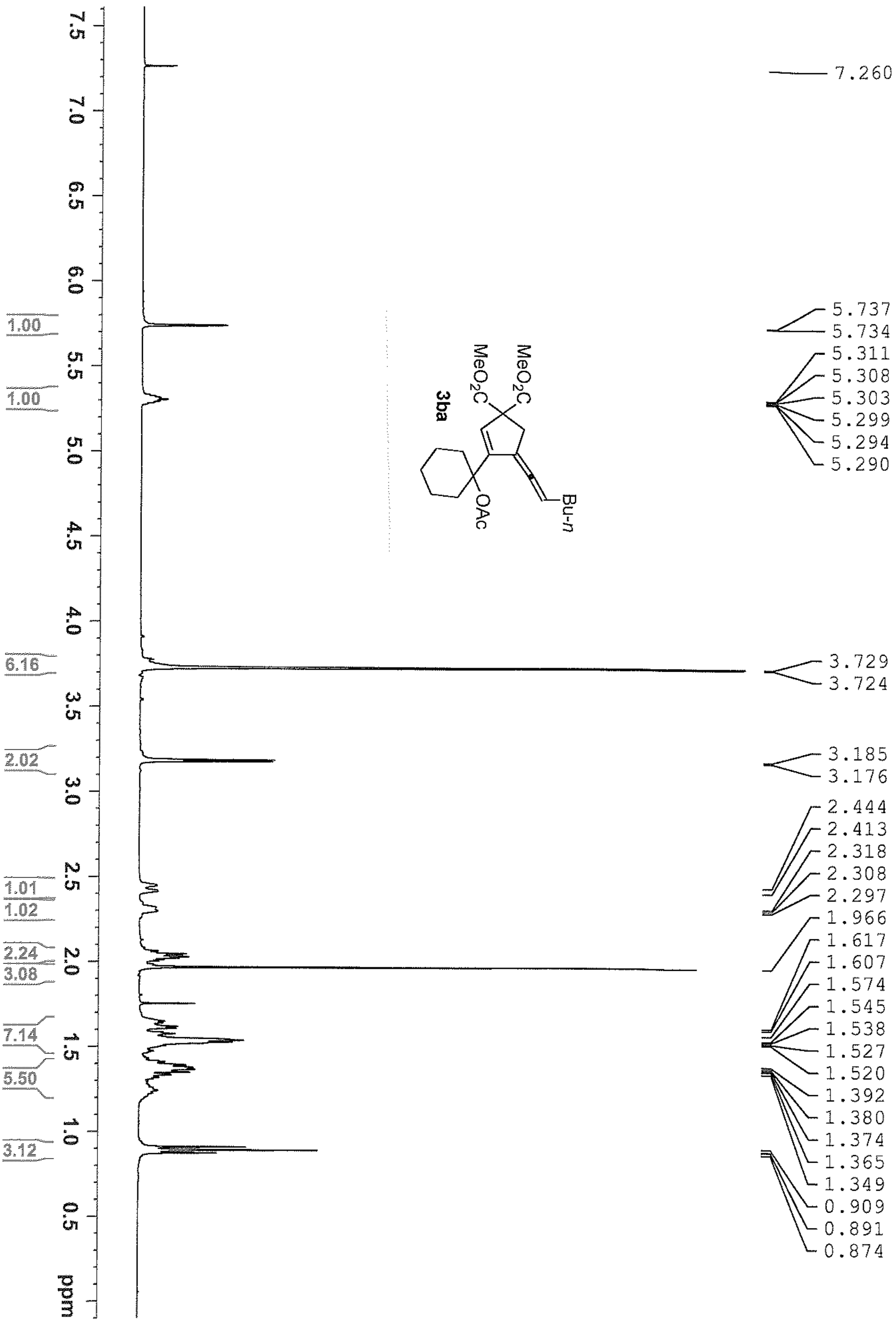

7.260
5.737
5.734
5.311
5.308
5.303
5.299
5.294
5.290
3.729
3.724
3.185
3.176
2.444
2.413
2.318
2.308
2.297
1.966
1.617
1.607
1.574
1.545
1.538
1.527
1.520
1.392
1.380
1.374
1.365
1.349
0.909
0.891
0.874
1.00
1.00
6.16
2.02
1.01
1.02
2.24
3.08
7.14
5.50
3.12
MeO2C
MeO2C
3ba
OAc
Bu-n
ppm

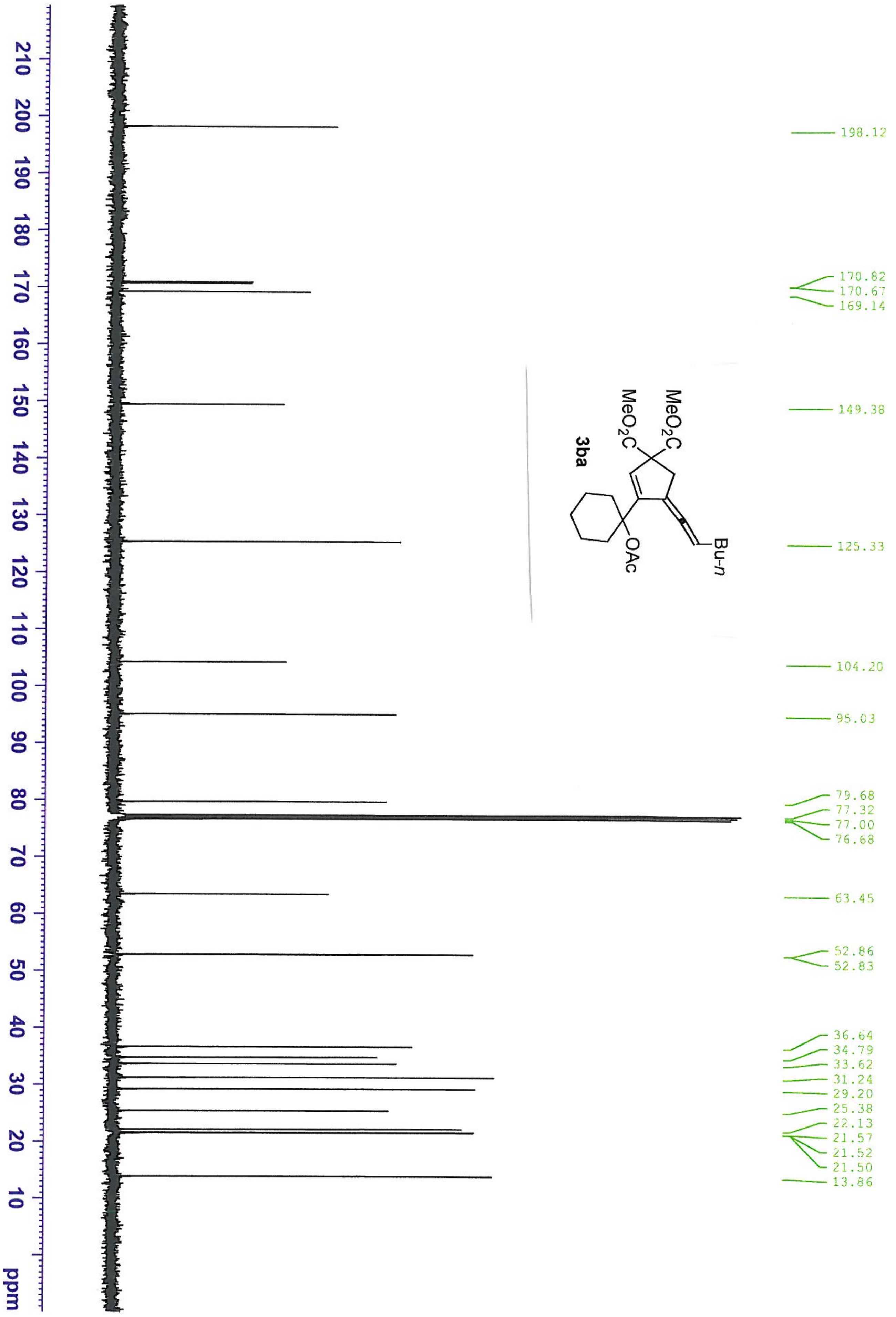
198.12
170.82
170.67
169.14
149.38
125.33
104.20
95.03
79.68
77.32
77.00
76.68
63.45
52.86
52.83
36.64
34.79
33.62
31.24
29.20
25.38
22.13
21.57
21.52
21.50
13.86
MeO2C
MeO2C
Bu-n
OAc
3ba
210
200
190
180
170
160
150
140
130
120
110
100
90
80
70
60
50
40
30
20
10
ppm

# Mass Spectrum SmartFormula Report

**Analysis Info**

| | |
|---|---|
| Analysis Name | H:\Data2\Youqian\dyq-3-169000002.d |
| Method | tune_wide_dirk.m |
| Sample Name | dyq-3-169 |
| Comment | |
| Acquisition Date | 2012-04-26 18:18:58 |
| Operator | pia |
| Instrument / Ser# | micrOTOF 125 |

**Acquisition Parameter**

| | | | | | |
|---|---|---|---|---|---|
| Source Type | ESI | Ion Polarity | Positive | Set Nebulizer | 0.4 Bar |
| Focus | Not active | | | Set Dry Heater | 180 °C |
| Scan Begin | 50 m/z | Set Capillary | 4500 V | Set Dry Gas | 4.0 l/min |
| Scan End | 3000 m/z | Set End Plate Offset | -500 V | Set Divert Valve | Source |

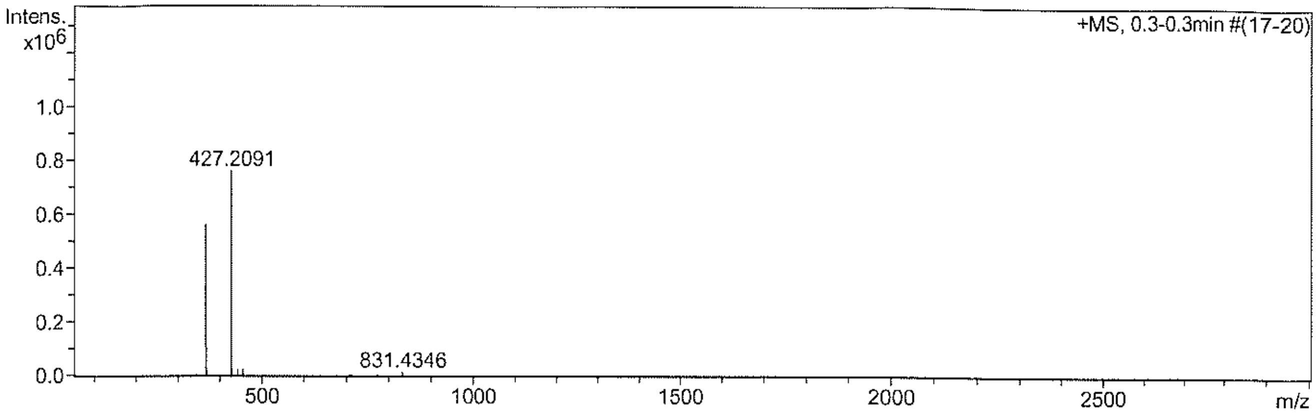


| Formula | Meas. m/z | m/z | err [ppm] | Mean err [ppm] |
|---|---|---|---|---|
| | 427.2091 | | | |
| C 23 H 32 Na O 6 | | 427.2091 | -0.0 | 0.7 |

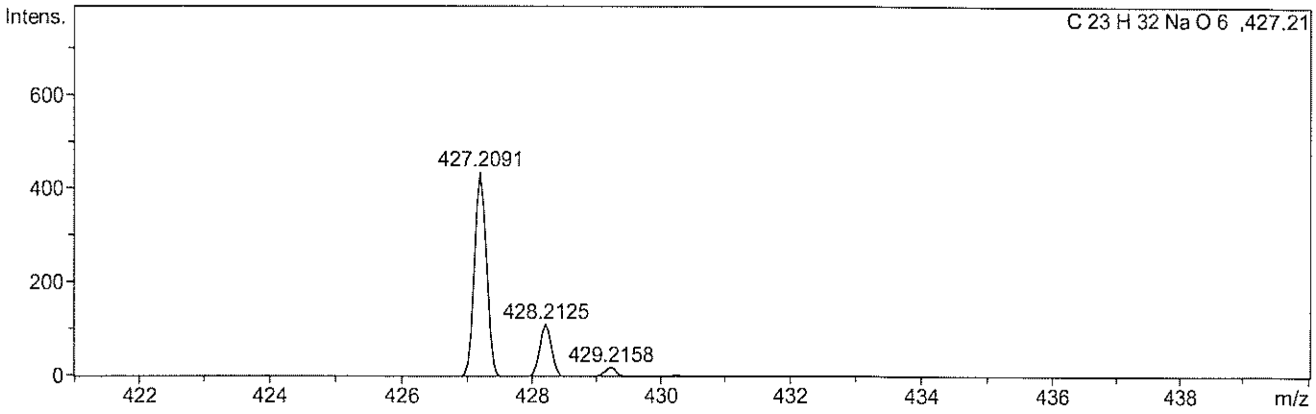


| Formula | Meas. m/z | m/z | err [ppm] | Mean err [ppm] |
|---|---|---|---|---|

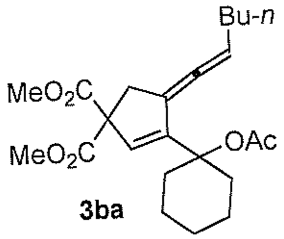


**3ba**

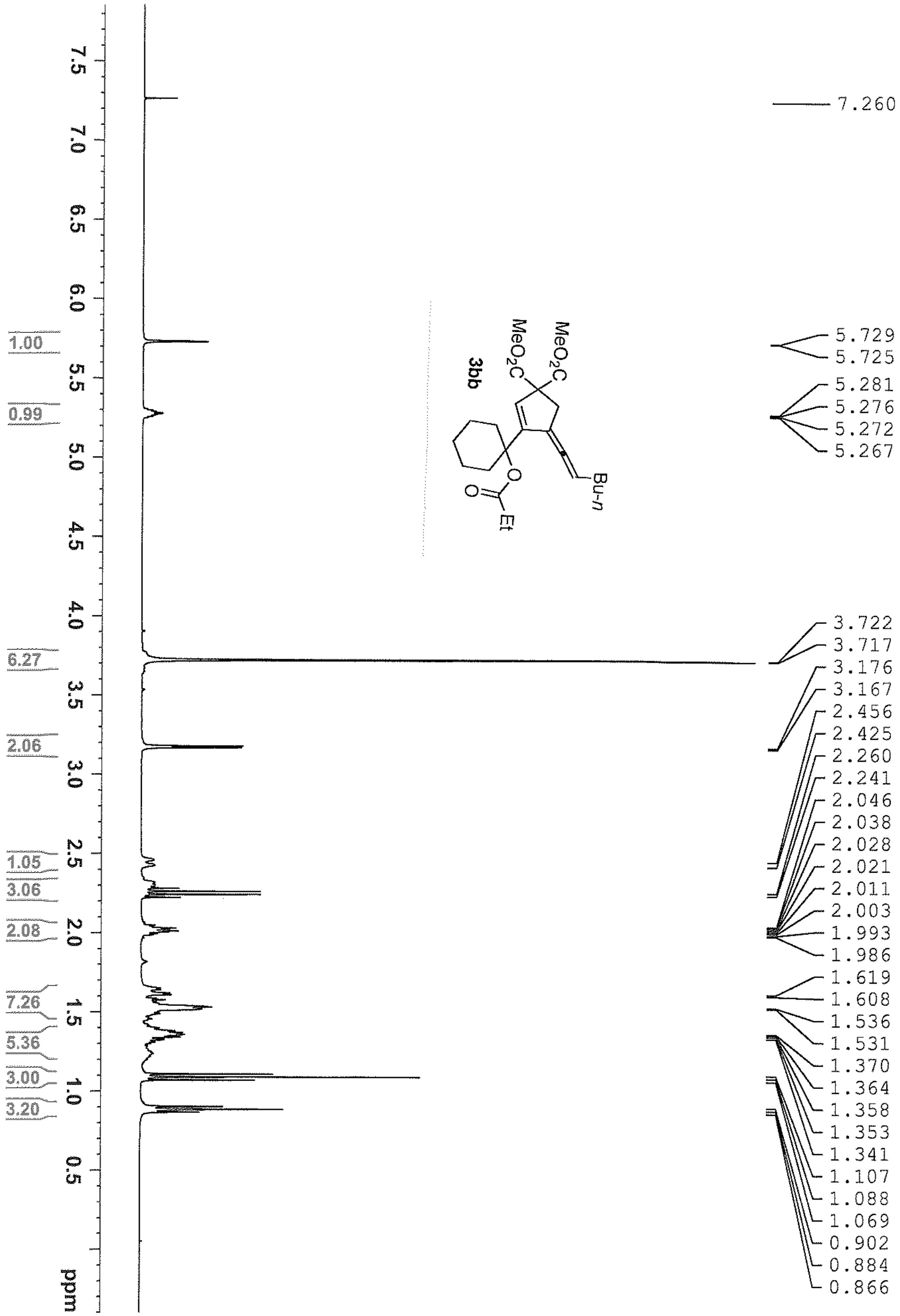

MeO2C
MeO2C
3bb
Bu-n
O
O
Et
7.260
5.729
5.725
5.281
5.276
5.272
5.267
3.722
3.717
3.176
3.167
2.456
2.425
2.260
2.241
2.046
2.038
2.028
2.021
2.011
2.003
1.993
1.986
1.619
1.608
1.536
1.531
1.370
1.364
1.358
1.353
1.341
1.107
1.088
1.069
0.902
0.884
0.866
1.00
0.99
6.27
2.06
1.05
3.06
2.08
7.26
5.36
3.00
3.20
7.5
7.0
6.5
6.0
5.5
5.0
4.5
4.0
3.5
3.0
2.5
2.0
1.5
1.0
0.5
ppm

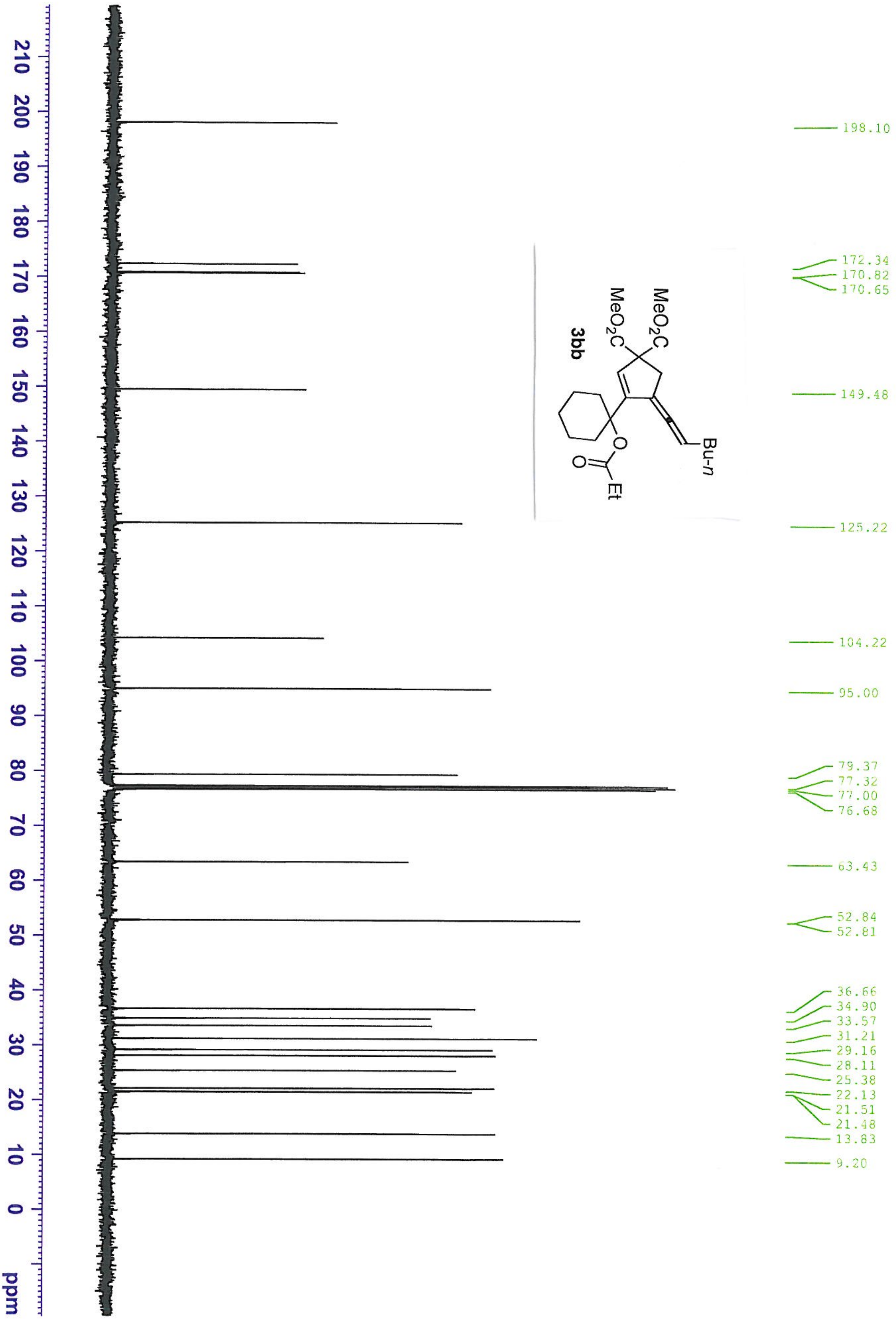
198.10
172.34
170.82
170.65
149.48
125.22
104.22
95.00
79.37
77.32
77.00
76.68
63.43
52.84
52.81
36.66
34.90
33.57
31.21
29.16
28.11
25.38
22.13
21.51
21.48
13.83
9.20
MeO2C
MeO2C
Bu-n
O
O
Et
3bb
210
200
190
180
170
160
150
140
130
120
110
100
90
80
70
60
50
40
30
20
10
0
ppm

# Mass Spectrum SmartFormula Report

**Analysis Info**

| | | | |
|---|---|---|---|
| Analysis Name | H:\Data2\Youqian\dyq-3-174000001.d | Acquisition Date | 2012-04-27 19:02:28 |
| Method | tune_wide_dirk.m | Operator | pia |
| Sample Name | dyq-3-174 | Instrument / Ser# | micrOTOF 125 |
| Comment | | | |

**Acquisition Parameter**

| | | | | | |
|---|---|---|---|---|---|
| Source Type | ESI | Ion Polarity | Positive | Set Nebulizer | 0.4 Bar |
| Focus | Not active | | | Set Dry Heater | 180 °C |
| Scan Begin | 50 m/z | Set Capillary | 4500 V | Set Dry Gas | 4.0 l/min |
| Scan End | 3000 m/z | Set End Plate Offset | -500 V | Set Divert Valve | Source |

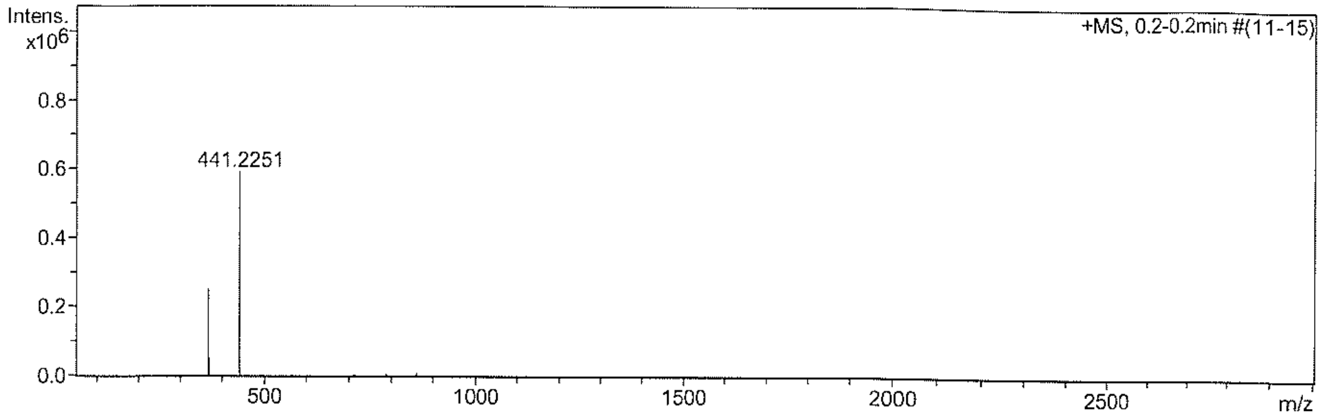


| Formula | Meas. m/z | m/z | err [ppm] | Mean err [ppm] |
|---|---|---|---|---|
| | 441.2251 | | | |
| C 24 H 34 Na O 6 | | 441.2248 | -0.9 | -0.2 |

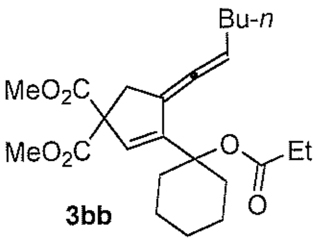


**3bb**

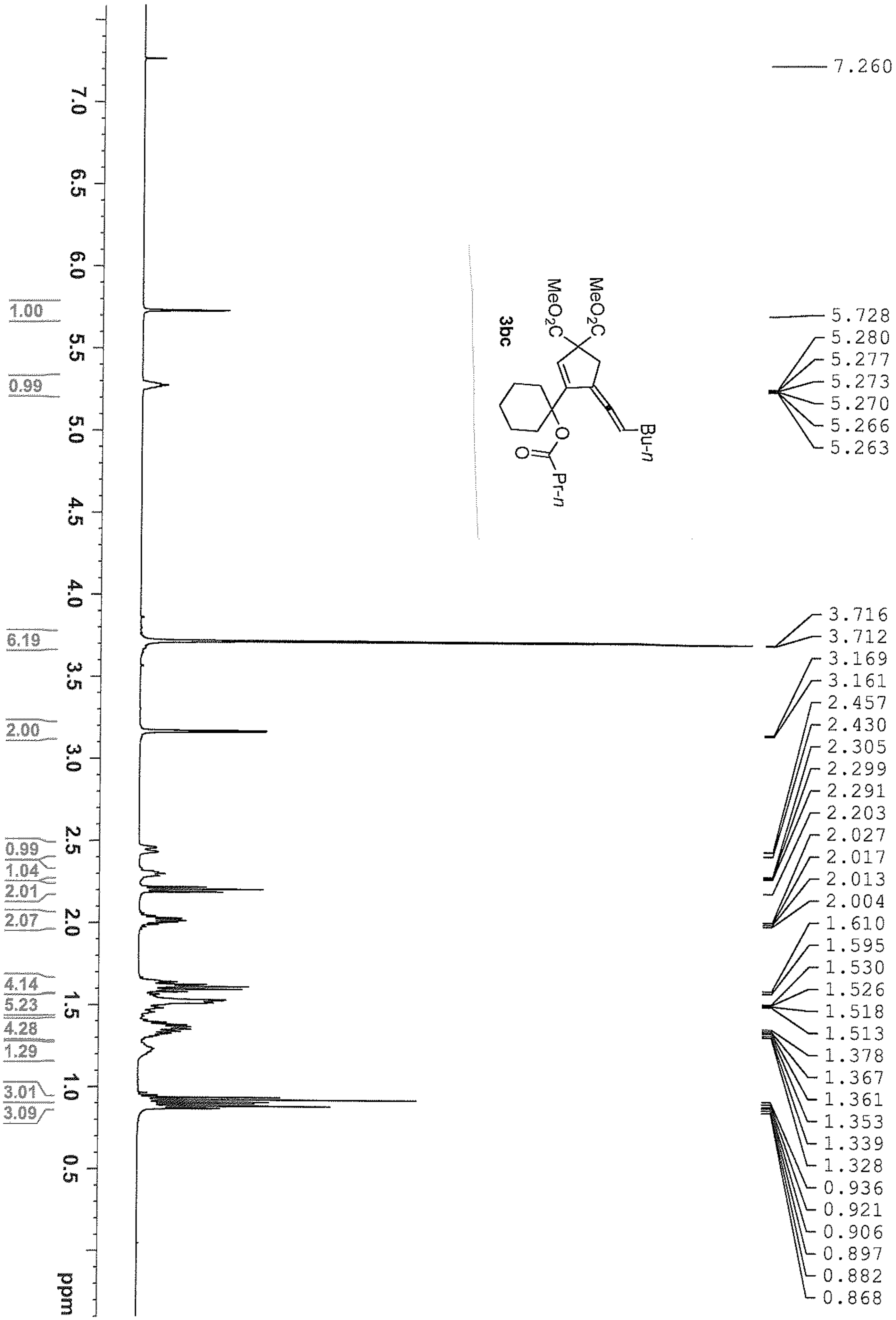

7.260
5.728
5.280
5.277
5.273
5.270
5.266
5.263
3.716
3.712
3.169
3.161
2.457
2.430
2.305
2.299
2.291
2.203
2.027
2.017
2.013
2.004
1.610
1.595
1.530
1.526
1.518
1.513
1.378
1.367
1.361
1.353
1.339
1.328
0.936
0.921
0.906
0.897
0.882
0.868
MeO2C
MeO2C
Bu-n
O
O
Pr-n
3bc
1.00
0.99
6.19
2.00
0.99
1.04
2.01
2.07
4.14
5.23
4.28
1.29
3.01
3.09
7.0
6.5
6.0
5.5
5.0
4.5
4.0
3.5
3.0
2.5
2.0
1.5
1.0
0.5
ppm

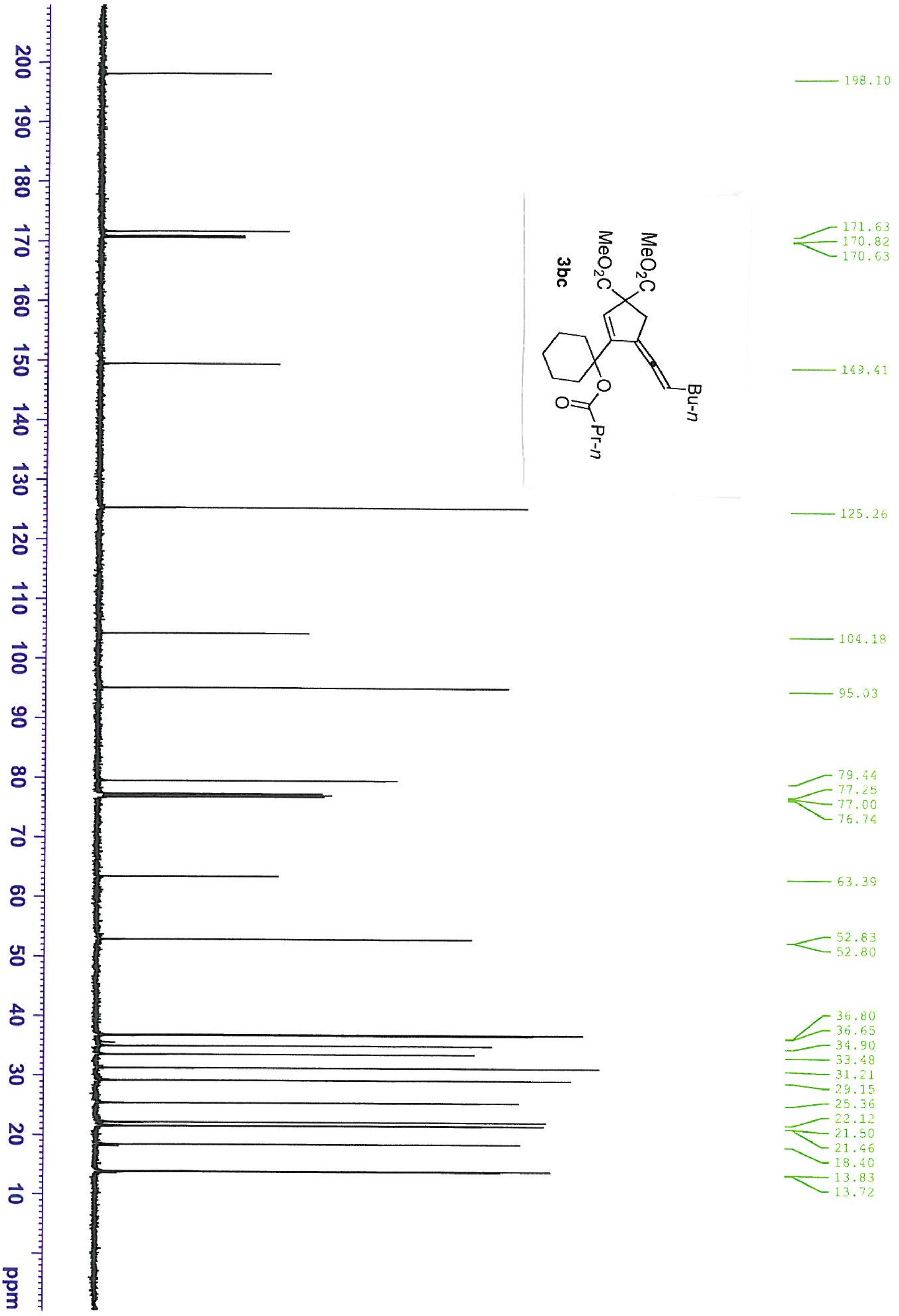
MeO2C
MeO2C
Bu-n
Pr-n
O
O
3bc
198.10
171.63
170.82
170.63
149.41
125.26
104.18
95.03
79.44
77.25
77.00
76.74
63.39
52.83
52.80
36.80
36.65
34.90
33.48
31.21
29.15
25.36
22.12
21.50
21.46
18.40
13.83
13.72
200
190
180
170
160
150
140
130
120
110
100
90
80
70
60
50
40
30
20
10
ppm

# Mass Spectrum SmartFormula Report

**Analysis Info**

| | | | |
|---|---|---|---|
| Analysis Name | H:\Data2\Youqian\dyq-3-176000002.d | Acquisition Date | 2012-04-27 19:11:58 |
| Method | tune_wide_dirk.m | Operator | pia |
| Sample Name | dyq-3-176 | Instrument / Ser# | micrOTOF 125 |
| Comment | | | |

**Acquisition Parameter**

| | | | | | |
|---|---|---|---|---|---|
| Source Type | ESI | Ion Polarity | Positive | Set Nebulizer | 0.4 Bar |
| Focus | Not active | | | Set Dry Heater | 180 °C |
| Scan Begin | 50 m/z | Set Capillary | 4500 V | Set Dry Gas | 4.0 l/min |
| Scan End | 3000 m/z | Set End Plate Offset | -500 V | Set Divert Valve | Source |

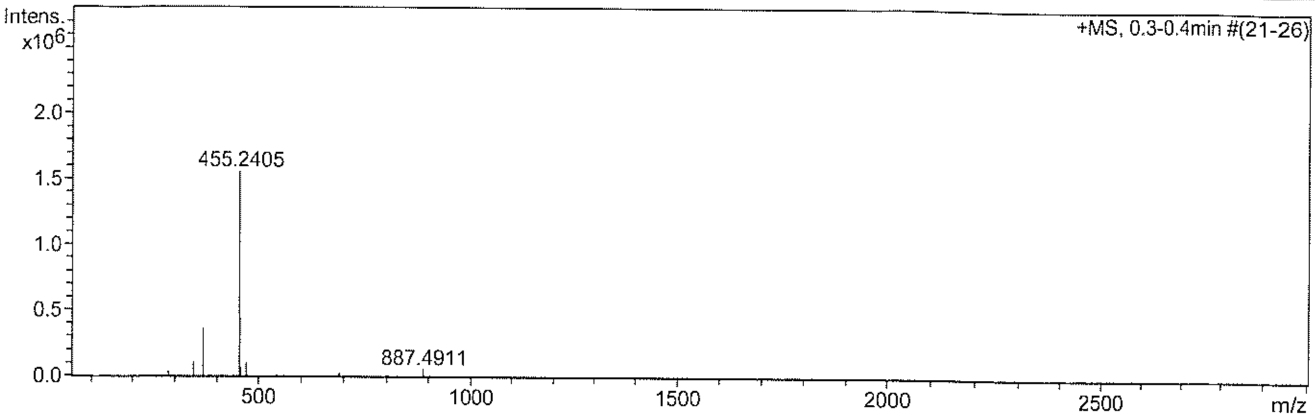


| Formula | Meas. m/z | m/z | err [ppm] | Mean err [ppm] |
|---|---|---|---|---|
| | 455.2405 | | | |
| C 25 H 36 Na O 6 | | 455.2404 | -0.2 | 0.5 |

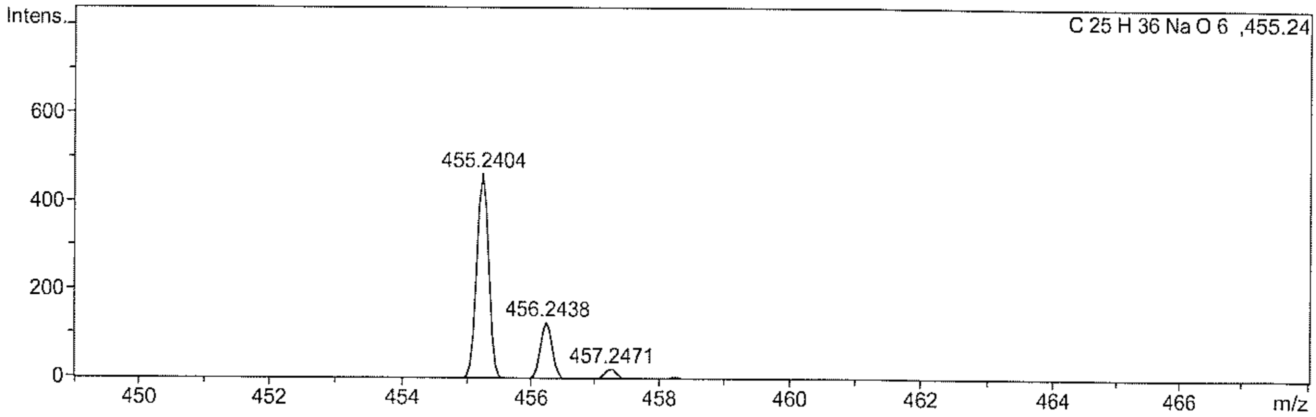


| Formula | Meas. m/z | m/z | err [ppm] | Mean err [ppm] |
|---|---|---|---|---|

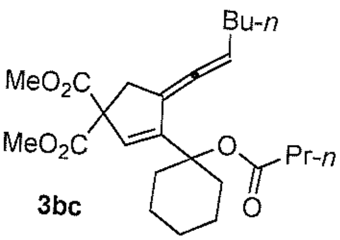


**3bc**

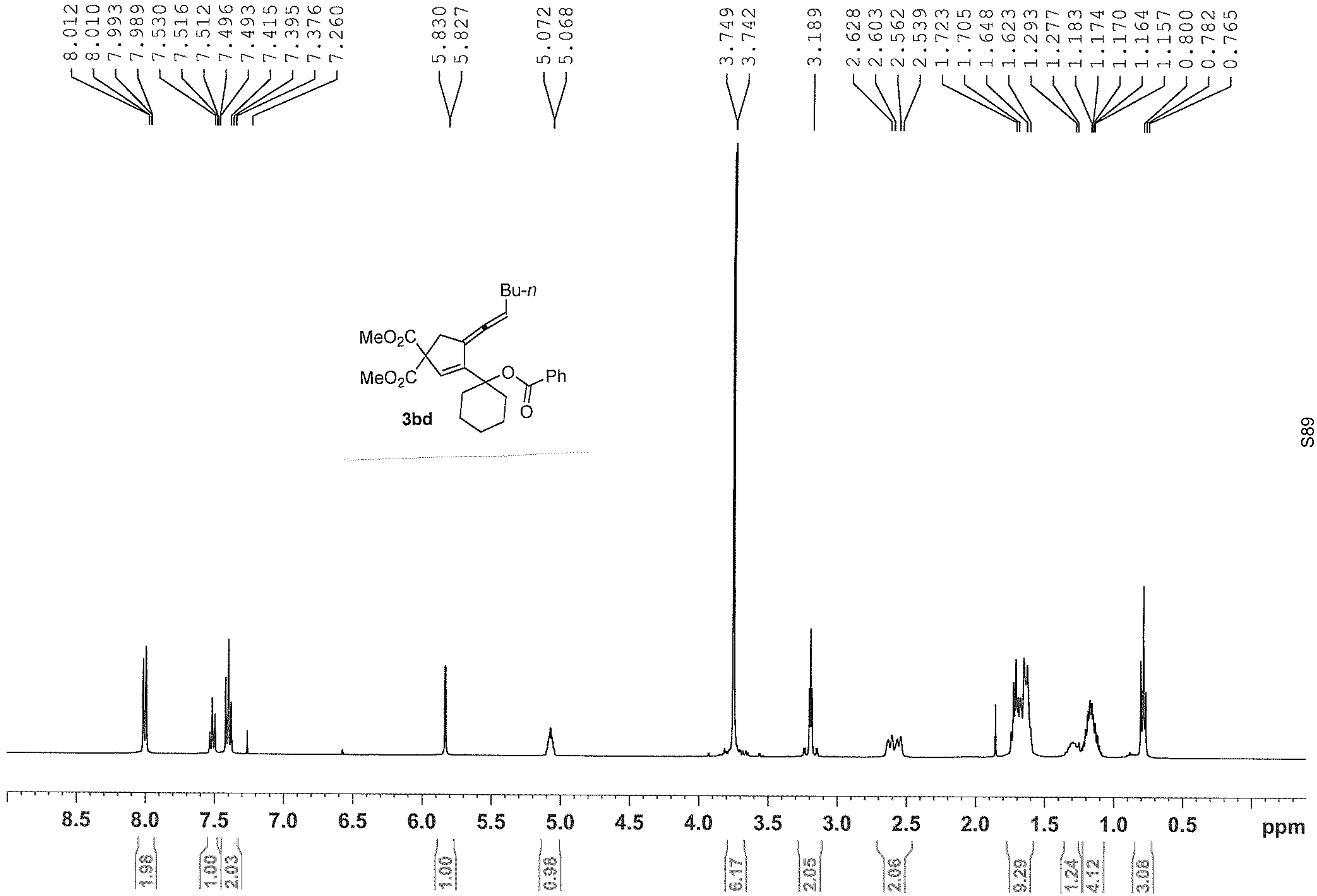
8.012
8.010
7.993
7.989
7.530
7.516
7.512
7.496
7.493
7.415
7.395
7.376
7.260
5.830
5.827
5.072
5.068
3.749
3.742
3.189
2.628
2.603
2.562
2.539
1.723
1.705
1.648
1.623
1.293
1.277
1.183
1.174
1.170
1.164
1.157
0.800
0.782
0.765
Bu-n
MeO2C
MeO2C
O
Ph
O
3bd
8.5
8.0
7.5
7.0
6.5
6.0
5.5
5.0
4.5
4.0
3.5
3.0
2.5
2.0
1.5
1.0
0.5
ppm
1.98
1.00
2.03
1.00
0.98
6.17
2.05
2.06
9.29
1.24
4.12
3.08

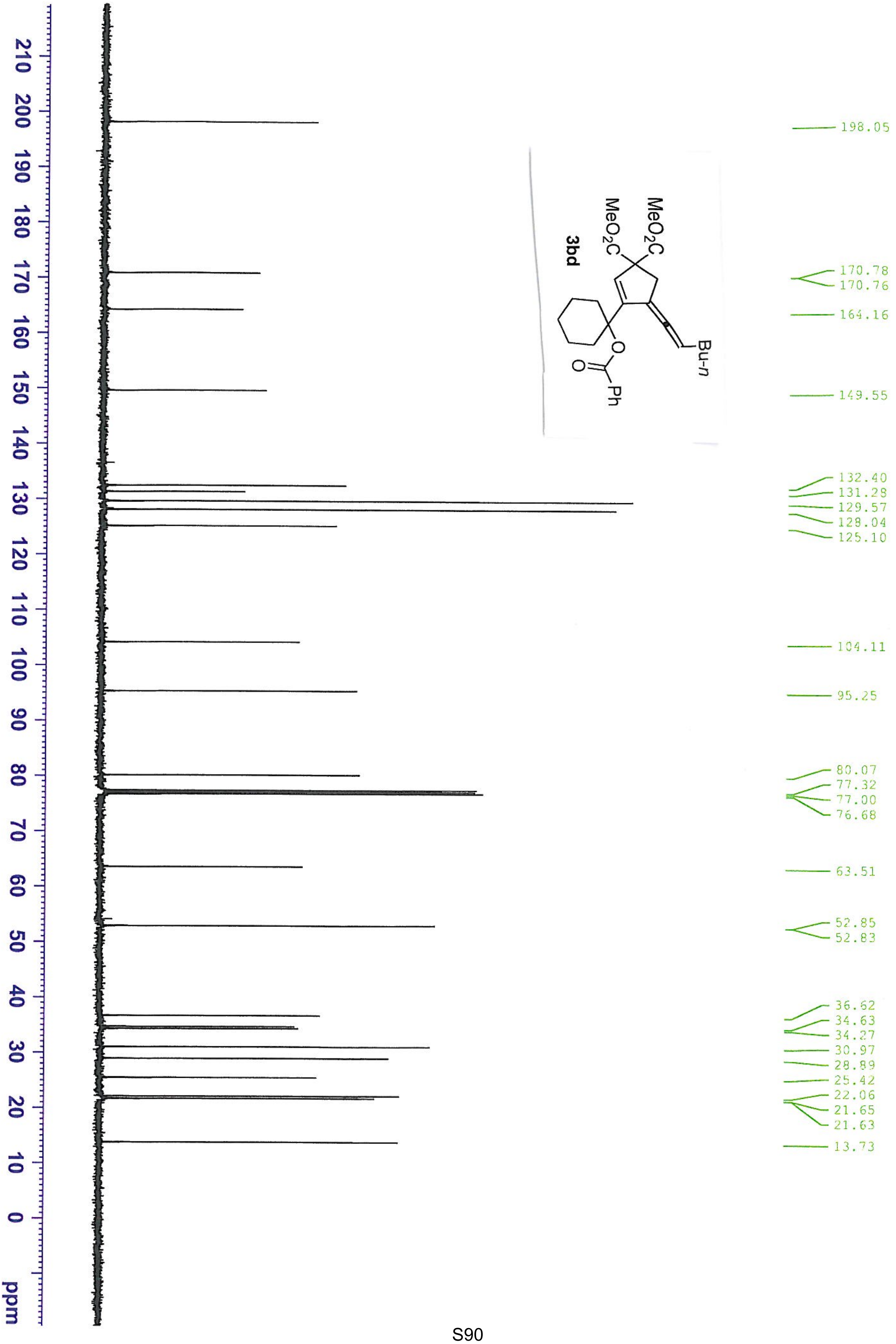
198.05
170.78
170.76
164.16
149.55
132.40
131.28
129.57
128.04
125.10
104.11
95.25
80.07
77.32
77.00
76.68
63.51
52.85
52.83
36.62
34.63
34.27
30.97
28.89
25.42
22.06
21.65
21.63
13.73
MeO2C
MeO2C
Bu-n
Ph
3bd
ppm

# Mass Spectrum SmartFormula Report

**Analysis Info**

| | | | |
|---|---|---|---|
| Analysis Name | H:\Data2\Youqian\dyq-4-19000002.d | Acquisition Date | 2012-06-01 16:23:30 |
| Method | tune_wide_dirk.m | Operator | pia |
| Sample Name | dyq-4-19 | Instrument / Ser# | micrOTOF 125 |
| Comment | | | |

**Acquisition Parameter**

| | | | | | |
|---|---|---|---|---|---|
| Source Type | ESI | Ion Polarity | Positive | Set Nebulizer | 0.4 Bar |
| Focus | Not active | | | Set Dry Heater | 180 °C |
| Scan Begin | 50 m/z | Set Capillary | 4500 V | Set Dry Gas | 4.0 l/min |
| Scan End | 3000 m/z | Set End Plate Offset | -500 V | Set Divert Valve | Source |

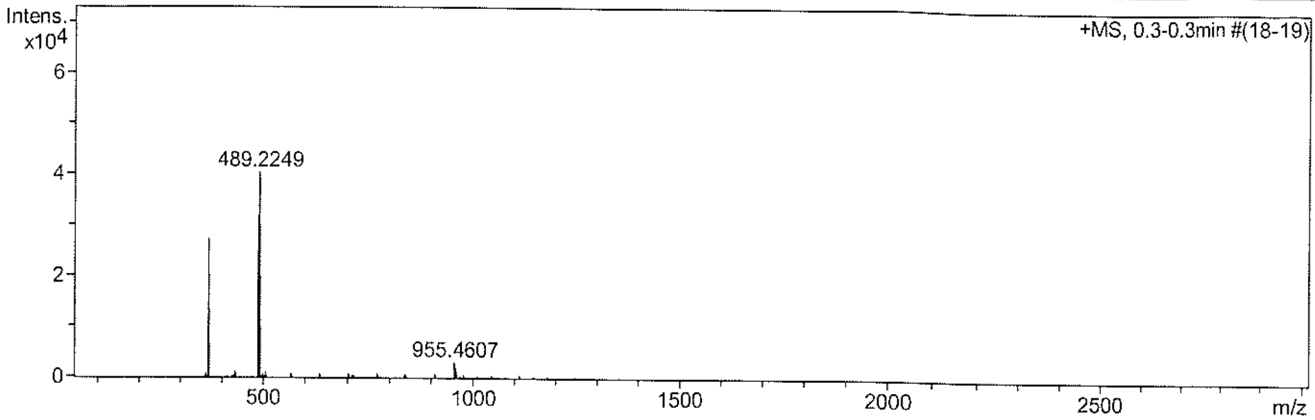


| Formula | Meas. m/z | m/z | err [ppm] | Mean err [ppm] |
|---|---|---|---|---|
| | 489.2249 | | | |
| C 28 H 34 Na O 6 | | 489.2248 | -0.3 | -0.3 |

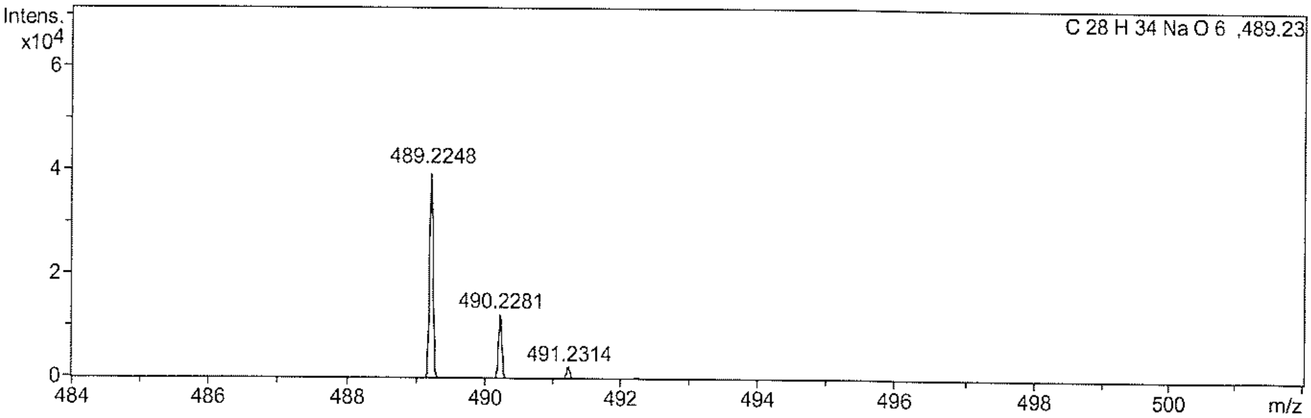


| Formula | Meas. m/z | m/z | err [ppm] | Mean err [ppm] |
|---|---|---|---|---|

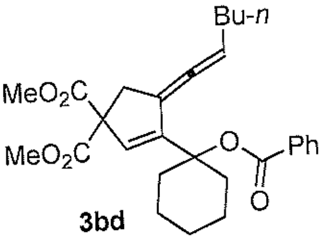

**3bd**

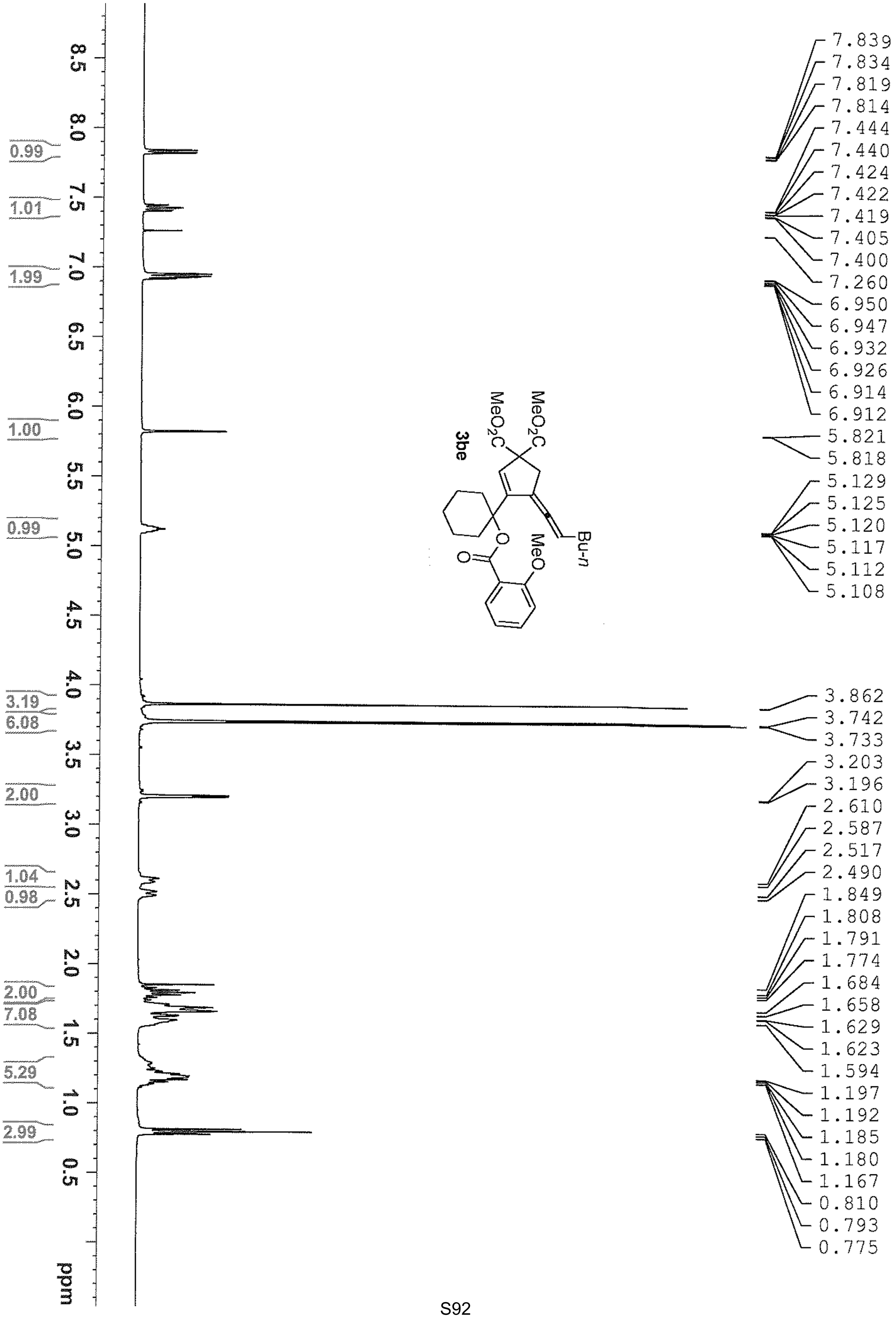
MeO2C
MeO2C
3be
MeO
Bu-n
O
O
8.5
8.0
7.5
7.0
6.5
6.0
5.5
5.0
4.5
4.0
3.5
3.0
2.5
2.0
1.5
1.0
0.5
ppm
0.99
1.01
1.99
1.00
0.99
3.19
6.08
2.00
1.04
0.98
2.00
7.08
5.29
2.99
7.839
7.834
7.819
7.814
7.444
7.440
7.424
7.422
7.419
7.405
7.400
7.260
6.950
6.947
6.932
6.926
6.914
6.912
5.821
5.818
5.129
5.125
5.120
5.117
5.112
5.108
3.862
3.742
3.733
3.203
3.196
2.610
2.587
2.517
2.490
1.849
1.808
1.791
1.774
1.684
1.658
1.629
1.623
1.594
1.197
1.192
1.185
1.180
1.167
0.810
0.793
0.775

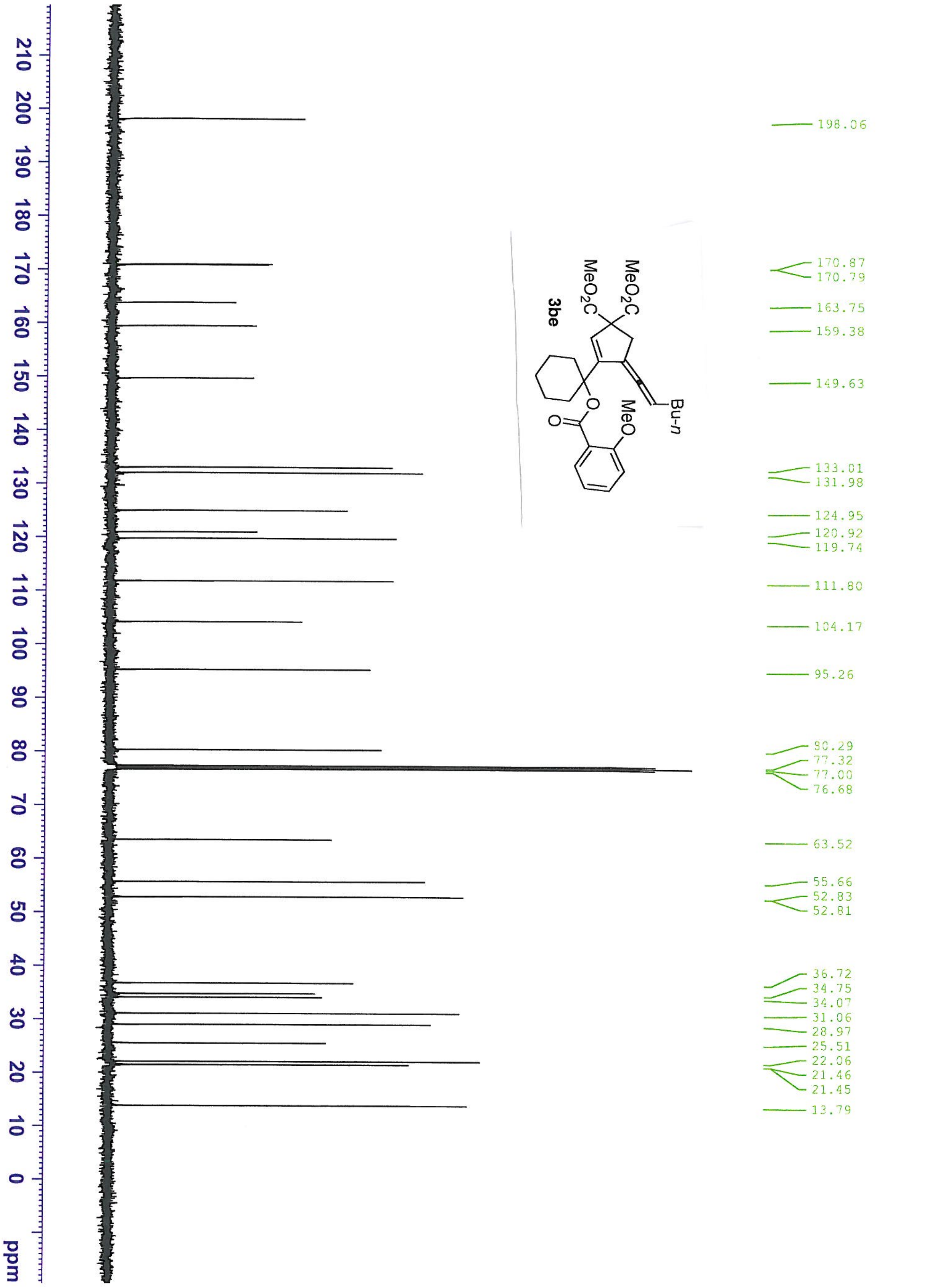
3be
MeO2C
MeO2C
MeO
Bu-n
O
O
198.06
170.87
170.79
163.75
159.38
149.63
133.01
131.98
124.95
120.92
119.74
111.80
104.17
95.26
80.29
77.32
77.00
76.68
63.52
55.66
52.83
52.81
36.72
34.75
34.07
31.06
28.97
25.51
22.06
21.46
21.45
13.79
ppm

# Mass Spectrum SmartFormula Report

**Analysis Info**

| | | | |
|---|---|---|---|
| Analysis Name | H:\Data2\Youqian\dyq-4-63000001.d | Acquisition Date | 2012-08-15 15:10:44 |
| Method | tune_wide_dirk.m | Operator | pia |
| Sample Name | | Instrument / Ser# | micrOTOF 125 |
| Comment | | | |

**Acquisition Parameter**

| | | | | | |
|---|---|---|---|---|---|
| Source Type | ESI | Ion Polarity | Positive | Set Nebulizer | 0.4 Bar |
| Focus | Not active | | | Set Dry Heater | 180 °C |
| Scan Begin | 50 m/z | Set Capillary | 4500 V | Set Dry Gas | 4.0 l/min |
| Scan End | 3000 m/z | Set End Plate Offset | -500 V | Set Divert Valve | Source |

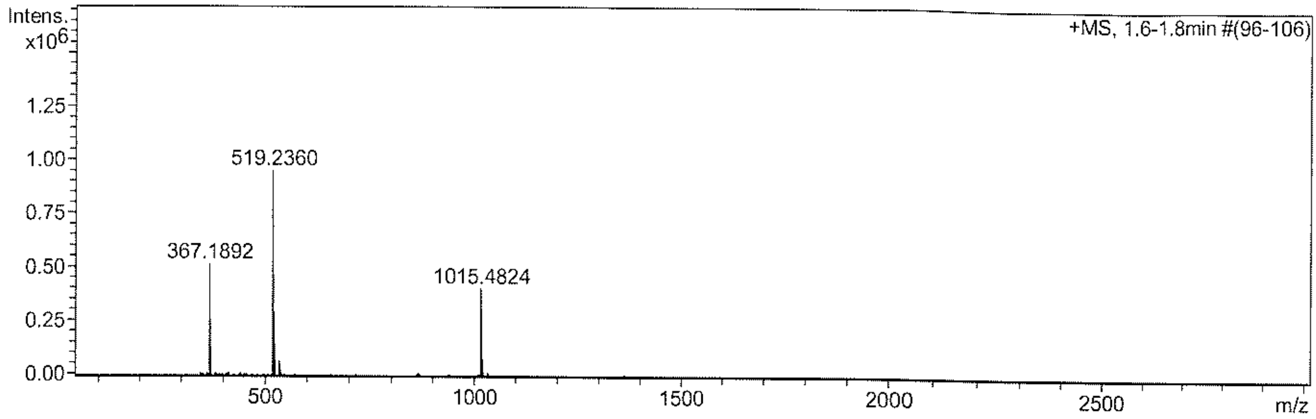


| Formula | Meas. m/z | m/z | err [ppm] | Mean err [ppm] |
|---|---|---|---|---|
| | 519.2360 | | | |
| C 29 H 36 Na O 7 | | 519.2353 | -1.3 | -1.0 |

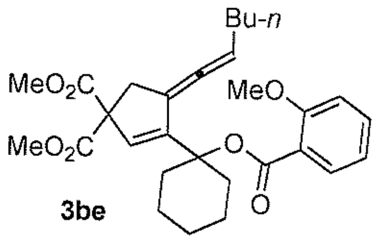


**3be**

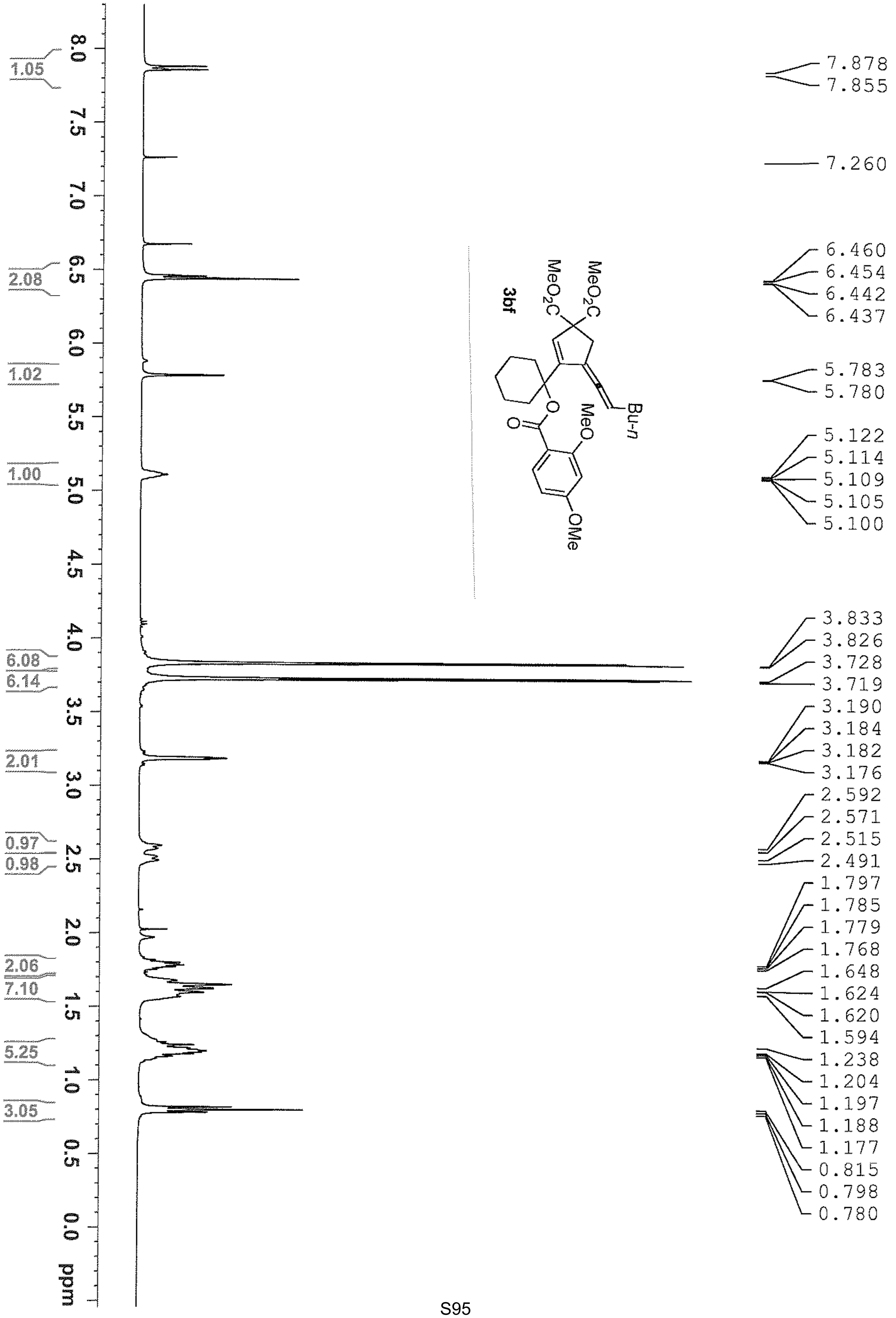
3bf
MeO2C
MeO2C
Bu-n
MeO
OMe
O
O
7.878
7.855
7.260
6.460
6.454
6.442
6.437
5.783
5.780
5.122
5.114
5.109
5.105
5.100
3.833
3.826
3.728
3.719
3.190
3.184
3.182
3.176
2.592
2.571
2.515
2.491
1.797
1.785
1.779
1.768
1.648
1.624
1.620
1.594
1.238
1.204
1.197
1.188
1.177
0.815
0.798
0.780
1.05
2.08
1.02
1.00
6.08
6.14
2.01
0.97
0.98
2.06
7.10
5.25
3.05
ppm

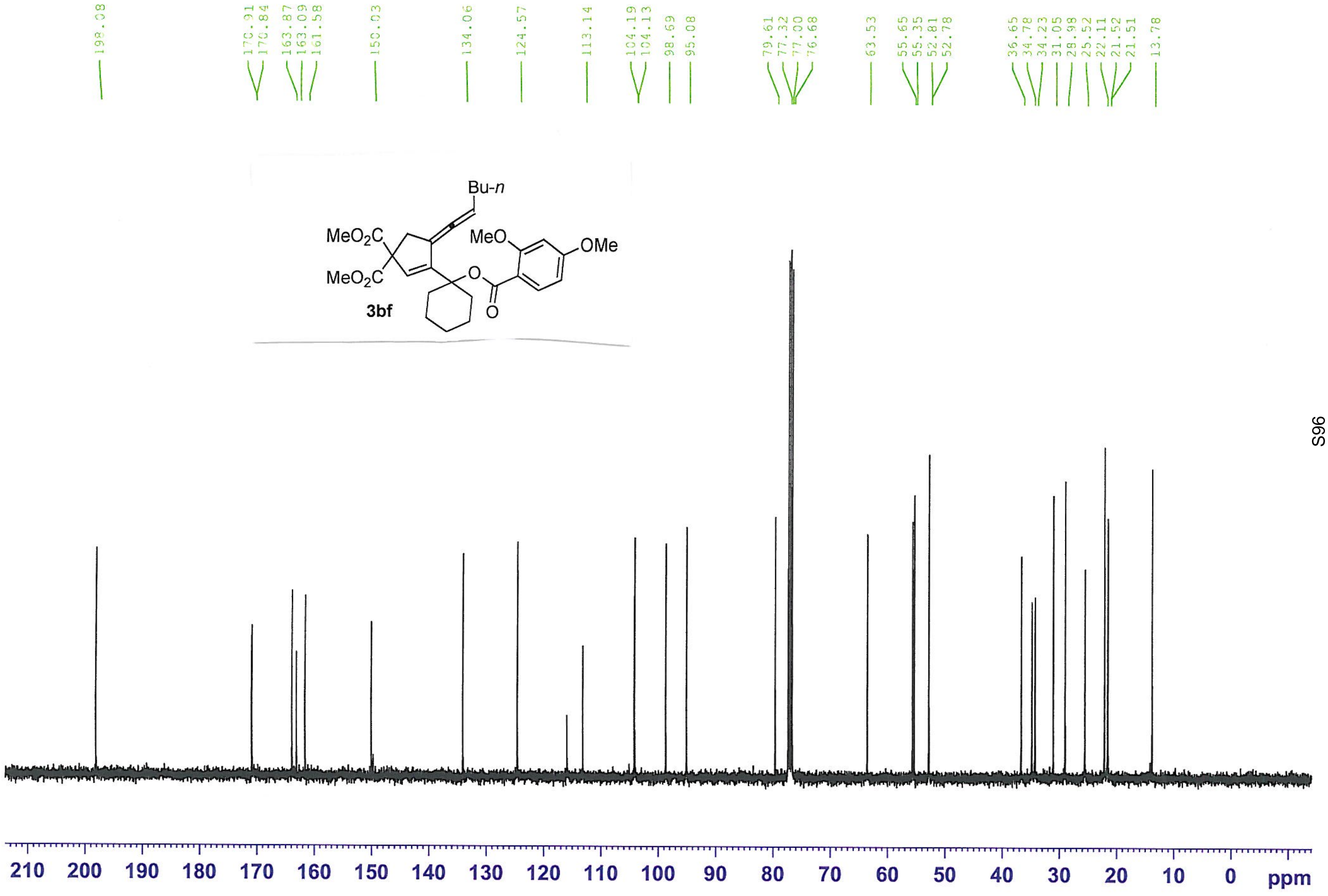
198.08
170.91
170.84
163.87
163.09
161.58
150.03
134.06
124.57
113.14
104.19
104.13
98.69
95.08
79.61
77.32
77.00
76.68
63.53
55.65
55.35
52.81
52.78
36.65
34.78
34.23
31.05
28.98
25.52
22.11
21.52
21.51
13.78
Bu-n
MeO2C
MeO2C
MeO
OMe
O
O
3bf
210 200 190 180 170 160 150 140 130 120 110 100 90 80 70 60 50 40 30 20 10 0 ppm

# Mass Spectrum SmartFormula Report

## Analysis Info

| | | | |
|---|---|---|---|
| Analysis Name | H:\Data2\Youqian\dyq-4-67000002.d | Acquisition Date | 2012-08-15 15:43:44 |
| Method | tune_wide_dirk.m | Operator | pia |
| Sample Name | | Instrument / Ser# | micrOTOF 125 |
| Comment | | | |

## Acquisition Parameter

| | | | | | |
|---|---|---|---|---|---|
| Source Type | ESI | Ion Polarity | Positive | Set Nebulizer | 0.4 Bar |
| Focus | Not active | | | Set Dry Heater | 180 °C |
| Scan Begin | 50 m/z | Set Capillary | 4500 V | Set Dry Gas | 4.0 l/min |
| Scan End | 3000 m/z | Set End Plate Offset | -500 V | Set Divert Valve | Source |

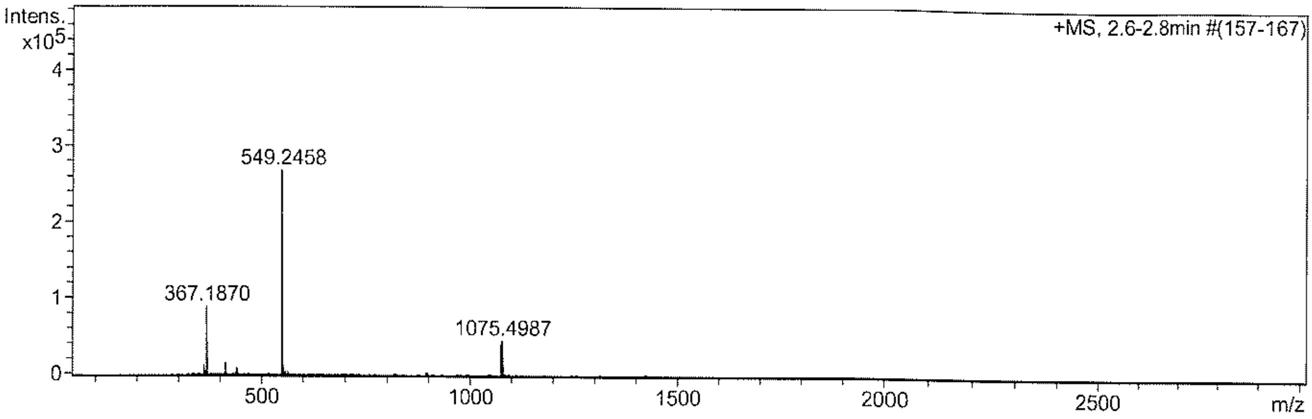


| Formula | Meas. m/z | m/z | err [ppm] | Mean err [ppm] |
|---|---|---|---|---|
| | 549.2458 | | | |
| C 30 H 38 Na O 8 | | 549.2459 | 0.2 | 0.4 |

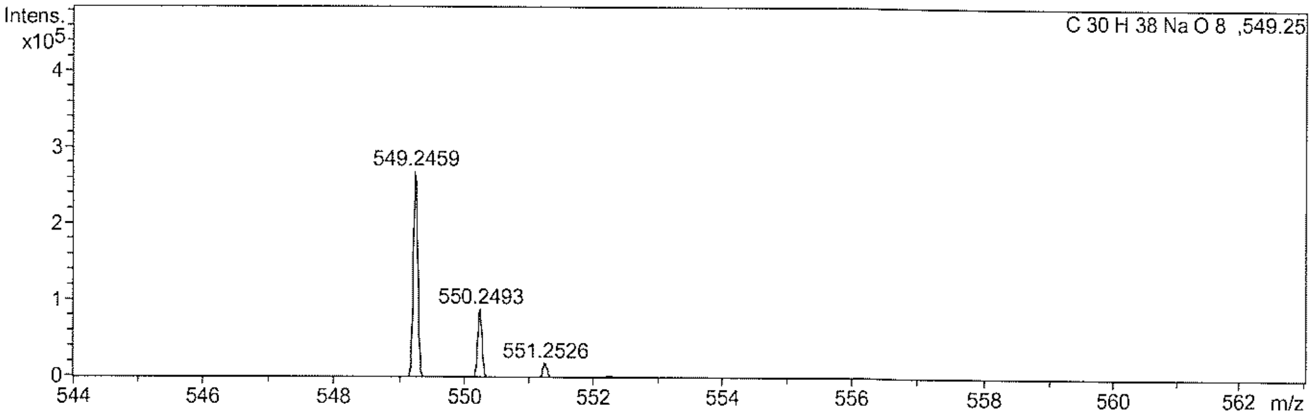


| Formula | Meas. m/z | m/z | err [ppm] | Mean err [ppm] |
|---|---|---|---|---|

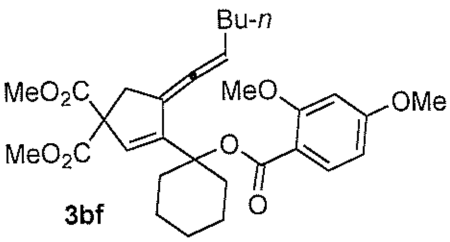


**3bf**

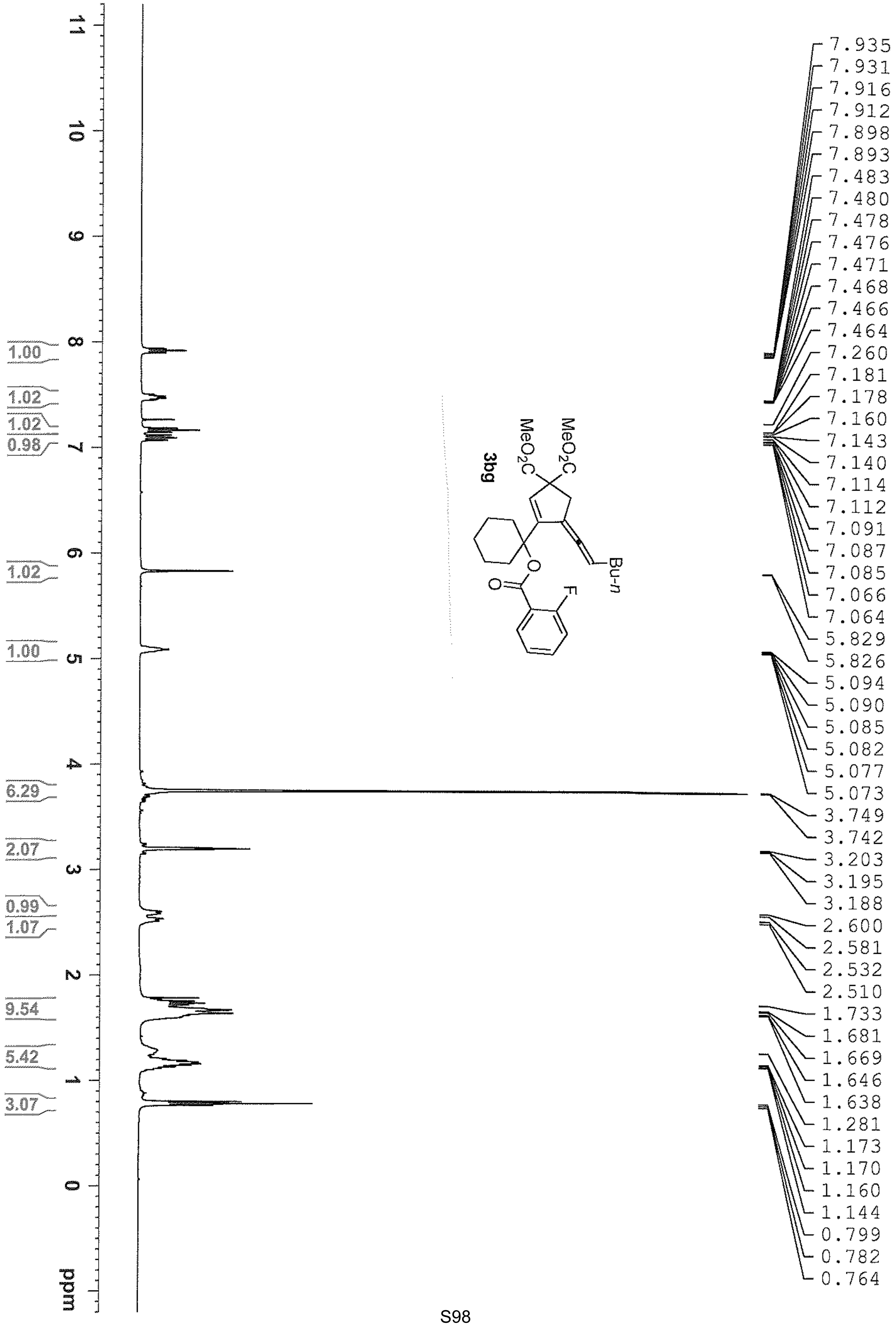
7.935
7.931
7.916
7.912
7.898
7.893
7.483
7.480
7.478
7.476
7.471
7.468
7.466
7.464
7.260
7.181
7.178
7.160
7.143
7.140
7.114
7.112
7.091
7.087
7.085
7.066
7.064
5.829
5.826
5.094
5.090
5.085
5.082
5.077
5.073
3.749
3.742
3.203
3.195
3.188
2.600
2.581
2.532
2.510
1.733
1.681
1.669
1.646
1.638
1.281
1.173
1.170
1.160
1.144
0.799
0.782
0.764
MeO2C
MeO2C
Bu-n
F
O
O
3bg
1.00
1.02
1.02
0.98
1.02
1.00
6.29
2.07
0.99
1.07
9.54
5.42
3.07
11
10
9
8
7
6
5
4
3
2
1
0
ppm

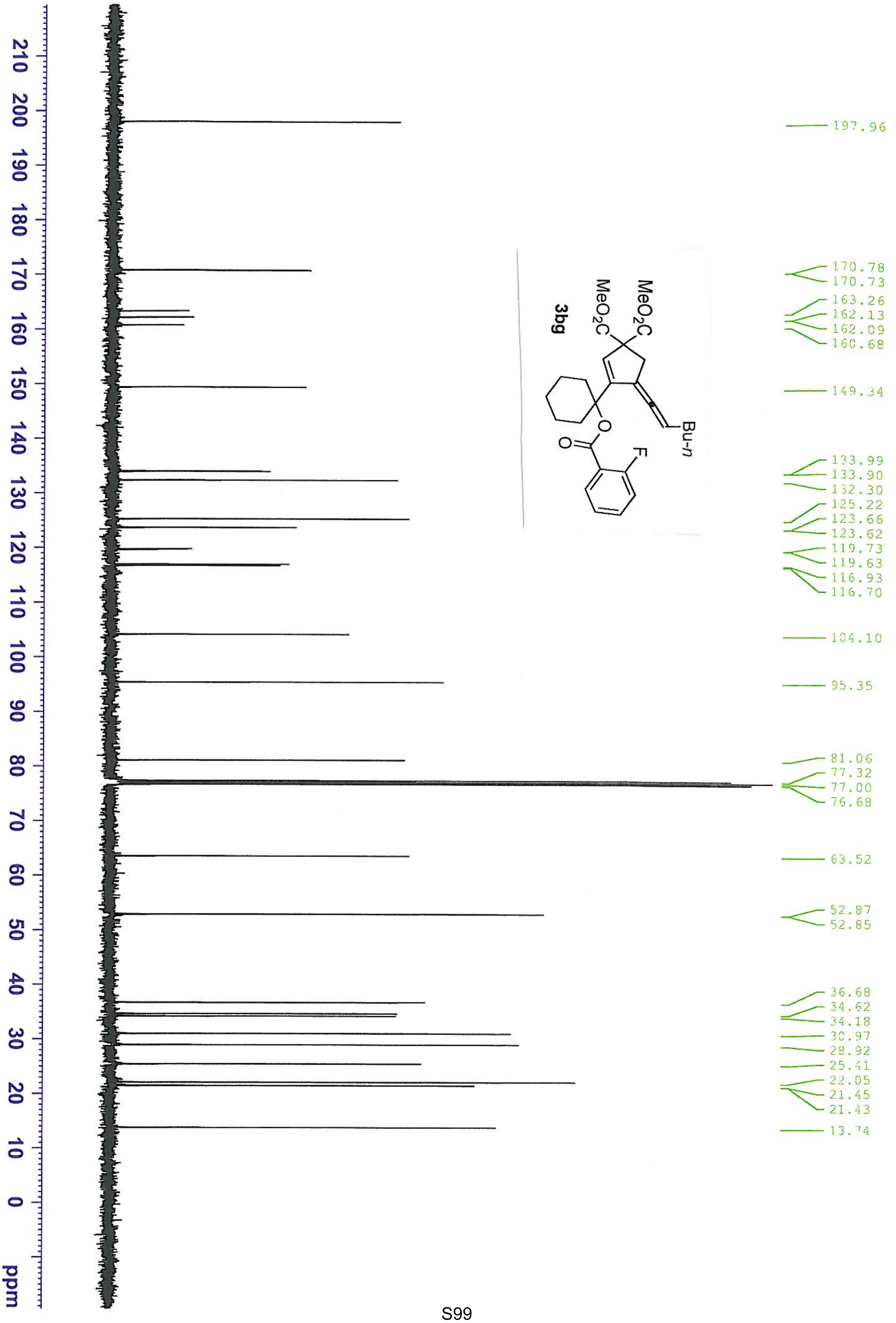
197.96
170.78
170.73
163.26
162.13
162.09
160.68
149.34
133.99
133.90
132.30
125.22
123.66
123.62
119.73
119.63
116.93
116.70
104.10
95.35
81.06
77.32
77.00
76.68
63.52
52.87
52.85
36.68
34.62
34.18
30.97
28.92
25.41
22.05
21.45
21.43
13.74
MeO$_2$C
MeO$_2$C
Bu-*n*
F
O
3bg
ppm

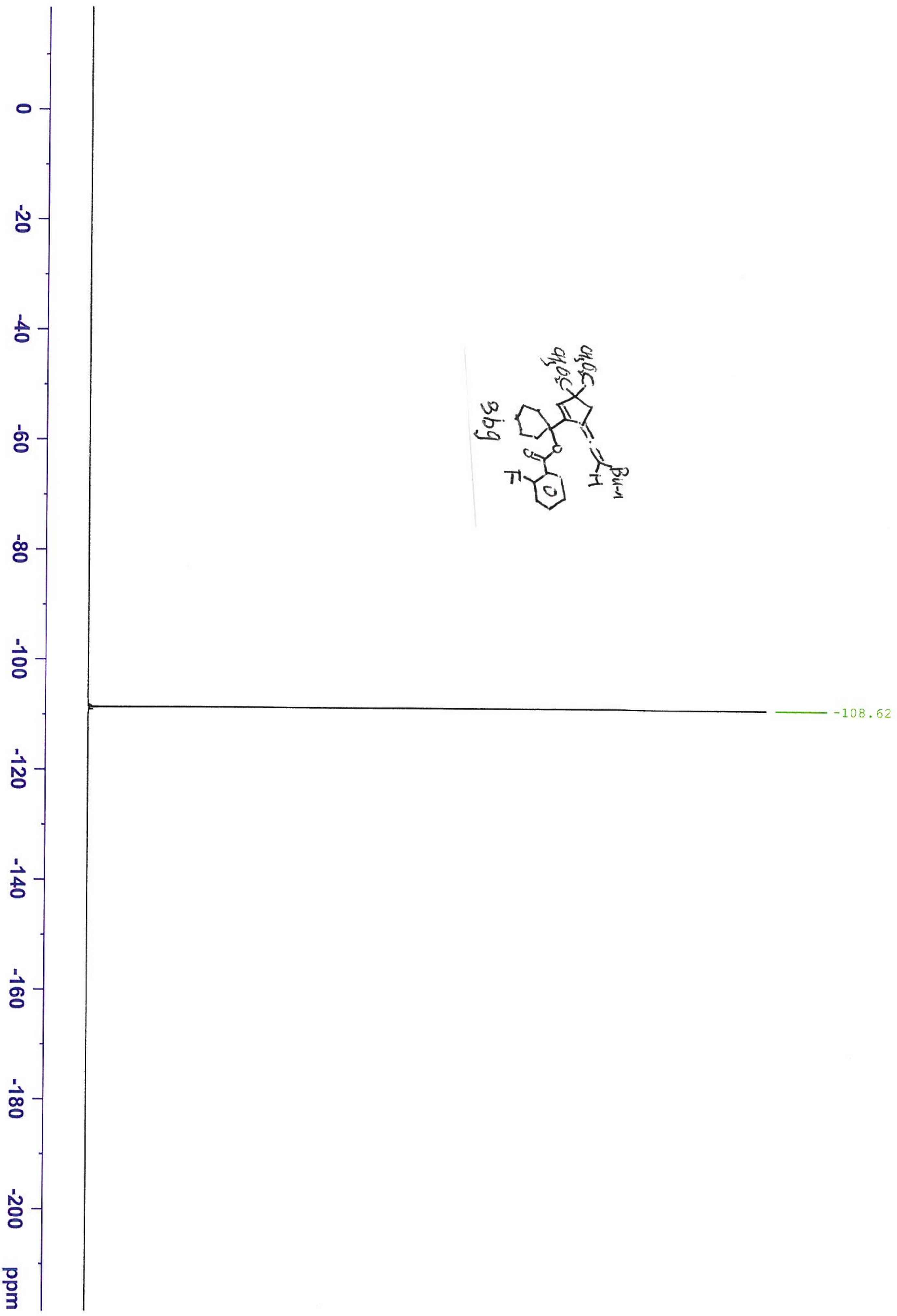
-108.62
3bg
0
-20
-40
-60
-80
-100
-120
-140
-160
-180
-200
ppm

# Mass Spectrum SmartFormula Report

**Analysis Info**

| | | | |
|---|---|---|---|
| Analysis Name | H:\Data2\Youqian\dyq-4-70000002.d | Acquisition Date | 2012-08-15 15:53:05 |
| Method | tune_wide_dirk.m | Operator | pia |
| Sample Name | | Instrument / Ser# | micrOTOF 125 |
| Comment | | | |

**Acquisition Parameter**

| | | | | | |
|---|---|---|---|---|---|
| Source Type | ESI | Ion Polarity | Positive | Set Nebulizer | 0.4 Bar |
| Focus | Not active | | | Set Dry Heater | 180 °C |
| Scan Begin | 50 m/z | Set Capillary | 4500 V | Set Dry Gas | 4.0 l/min |
| Scan End | 3000 m/z | Set End Plate Offset | -500 V | Set Divert Valve | Source |

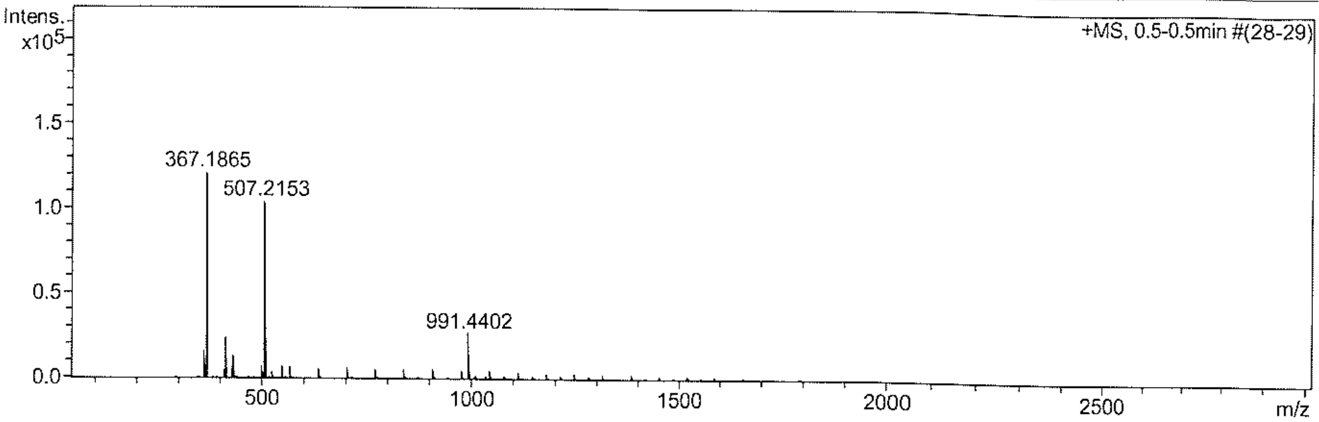


| Formula | Meas. m/z | m/z | err [ppm] | Mean err [ppm] |
|---|---|---|---|---|
| | 507.2153 | | | |
| C 28 H 33 F Na O 6 | | 507.2153 | 0.0 | -0.1 |

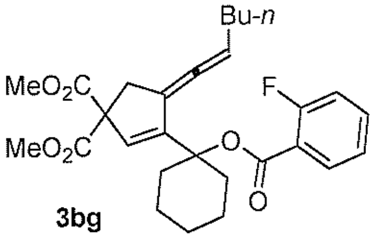


**3bg**

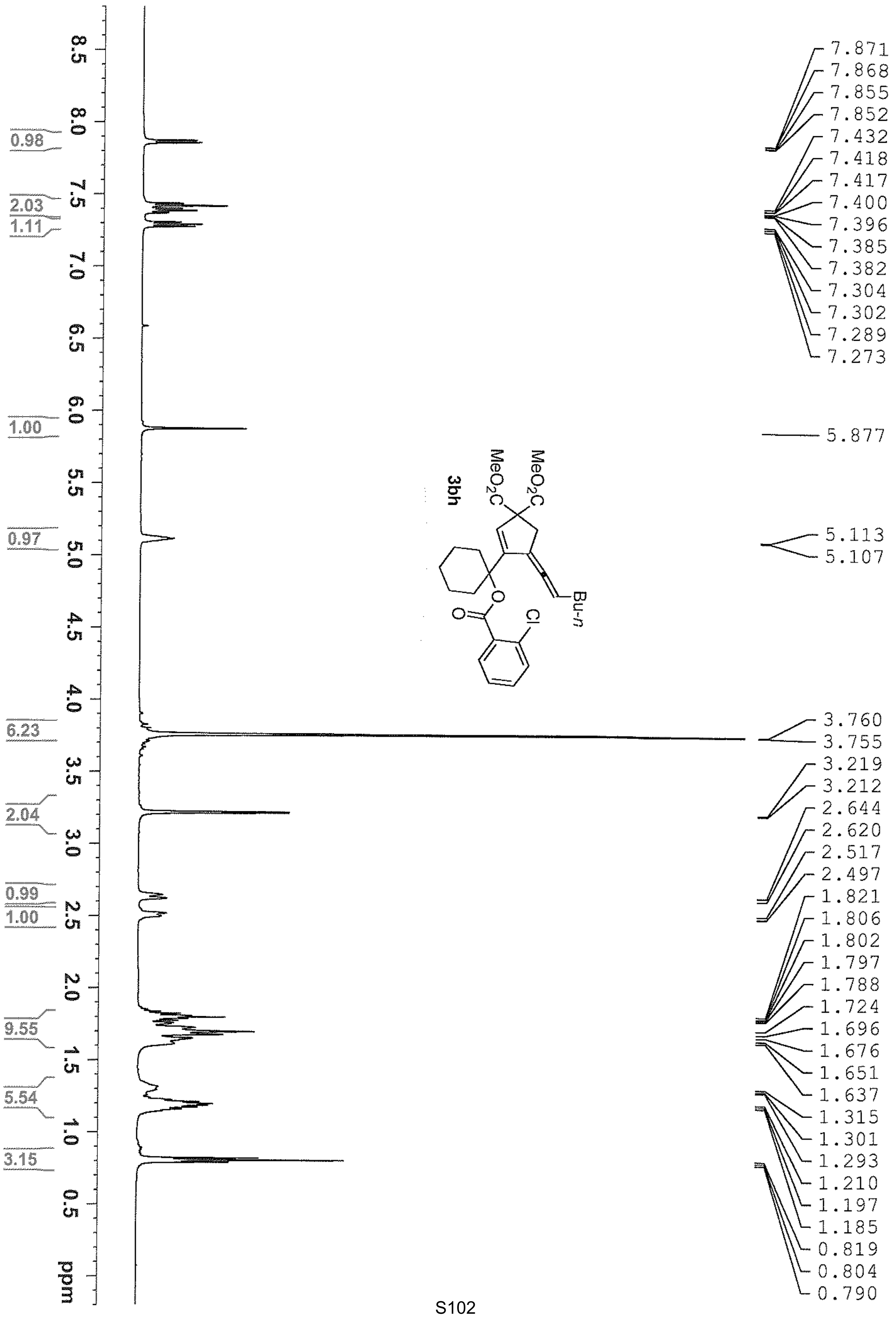
3bh
MeO2C
MeO2C
Bu-n
Cl
O
O
7.871
7.868
7.855
7.852
7.432
7.418
7.417
7.400
7.396
7.385
7.382
7.304
7.302
7.289
7.273
5.877
5.113
5.107
3.760
3.755
3.219
3.212
2.644
2.620
2.517
2.497
1.821
1.806
1.802
1.797
1.788
1.724
1.696
1.676
1.651
1.637
1.315
1.301
1.293
1.210
1.197
1.185
0.819
0.804
0.790
0.98
2.03
1.11
1.00
0.97
6.23
2.04
0.99
1.00
9.55
5.54
3.15
8.5
8.0
7.5
7.0
6.5
6.0
5.5
5.0
4.5
4.0
3.5
3.0
2.5
2.0
1.5
1.0
0.5
ppm

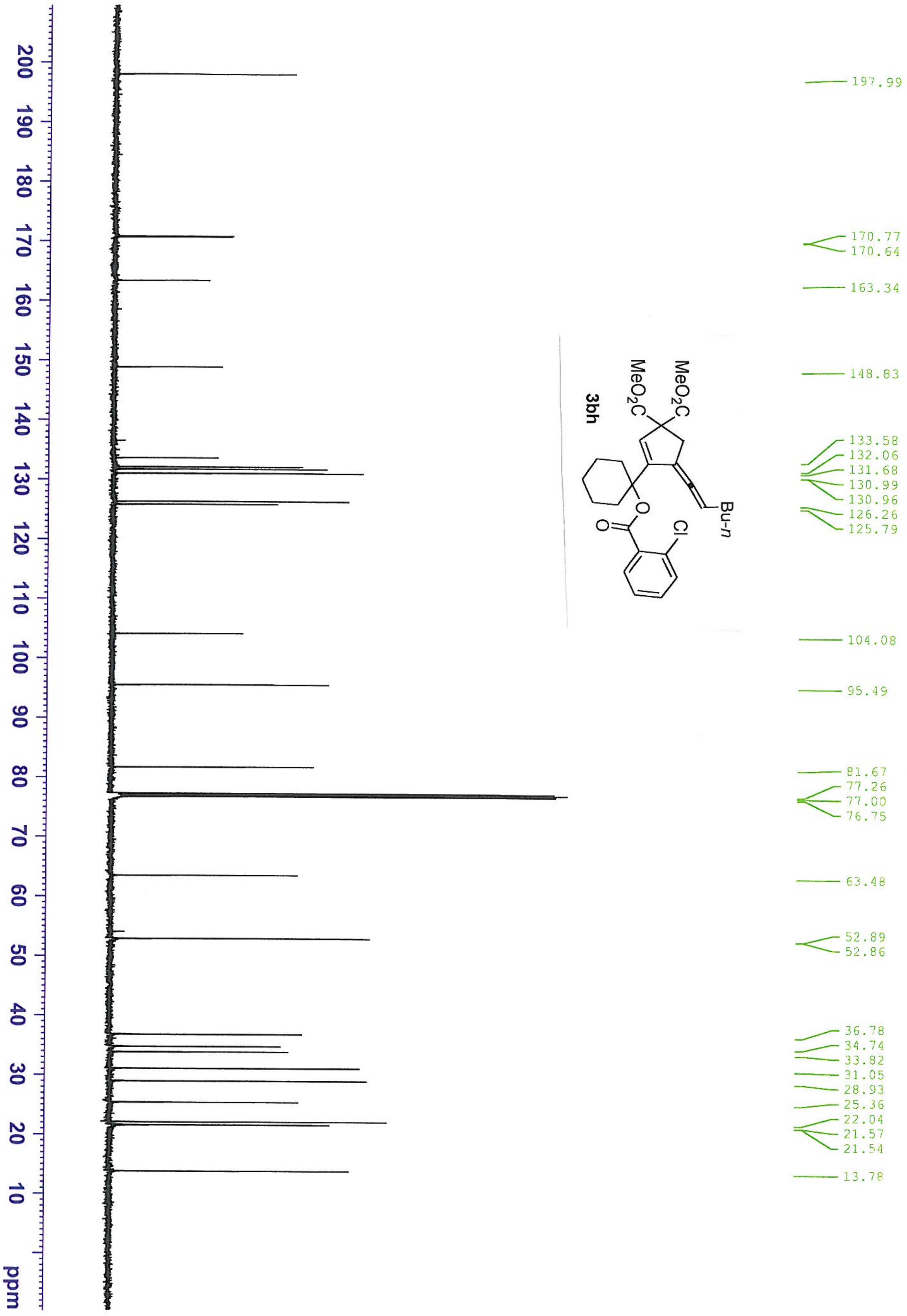

3bh
MeO2C
MeO2C
Bu-n
Cl
O
O
197.99
170.77
170.64
163.34
148.83
133.58
132.06
131.68
130.99
130.96
126.26
125.79
104.08
95.49
81.67
77.26
77.00
76.75
63.48
52.89
52.86
36.78
34.74
33.82
31.05
28.93
25.36
22.04
21.57
21.54
13.78
ppm

# Mass Spectrum SmartFormula Report

**Analysis Info**

| | | | |
|---|---|---|---|
| Analysis Name | H:\Data2\Youqian\dyq-4-62000002.d | Acquisition Date | 2012-08-15 15:03:12 |
| Method | tune_wide_dirk.m | | |
| Sample Name | | Operator | pia |
| Comment | | Instrument / Ser# | micrOTOF 125 |

**Acquisition Parameter**

| | | | | | |
|---|---|---|---|---|---|
| Source Type | ESI | Ion Polarity | Positive | Set Nebulizer | 0.4 Bar |
| Focus | Not active | | | Set Dry Heater | 180 °C |
| Scan Begin | 50 m/z | Set Capillary | 4500 V | Set Dry Gas | 4.0 l/min |
| Scan End | 3000 m/z | Set End Plate Offset | -500 V | Set Divert Valve | Source |

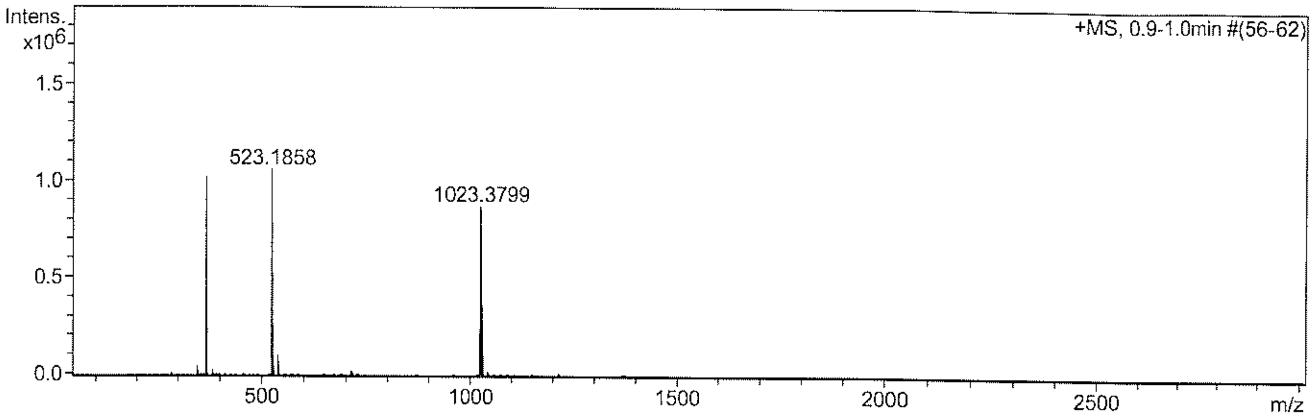


| Formula | Meas. m/z | m/z | err [ppm] | Mean err [ppm] |
|---|---|---|---|---|
| | 523.1858 | | | |
| C 28 H 33 Cl Na O 6 | | 523.1858 | -0.0 | -0.1 |

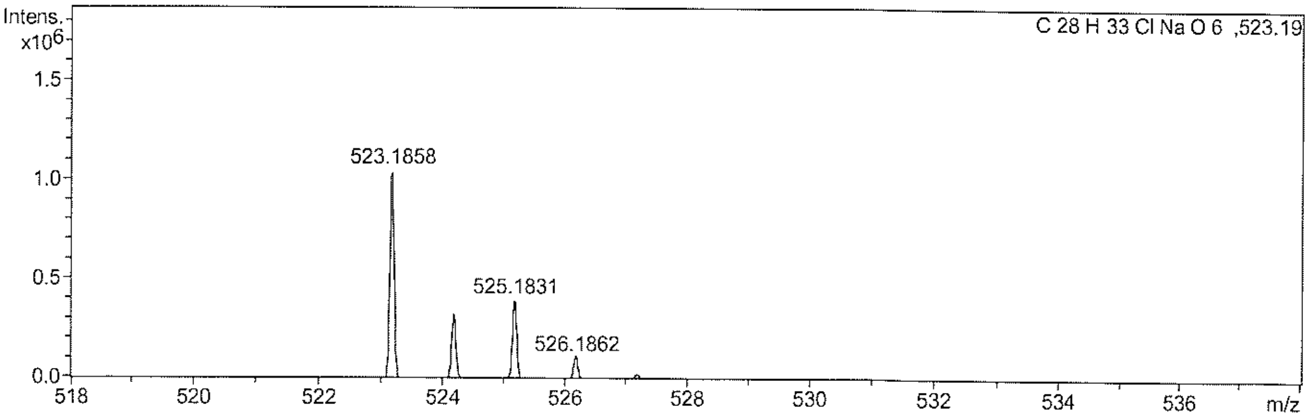


| Formula | Meas. m/z | m/z | err [ppm] | Mean err [ppm] |
|---|---|---|---|---|

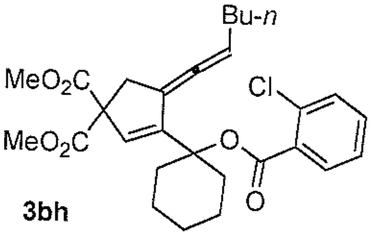


**3bh**

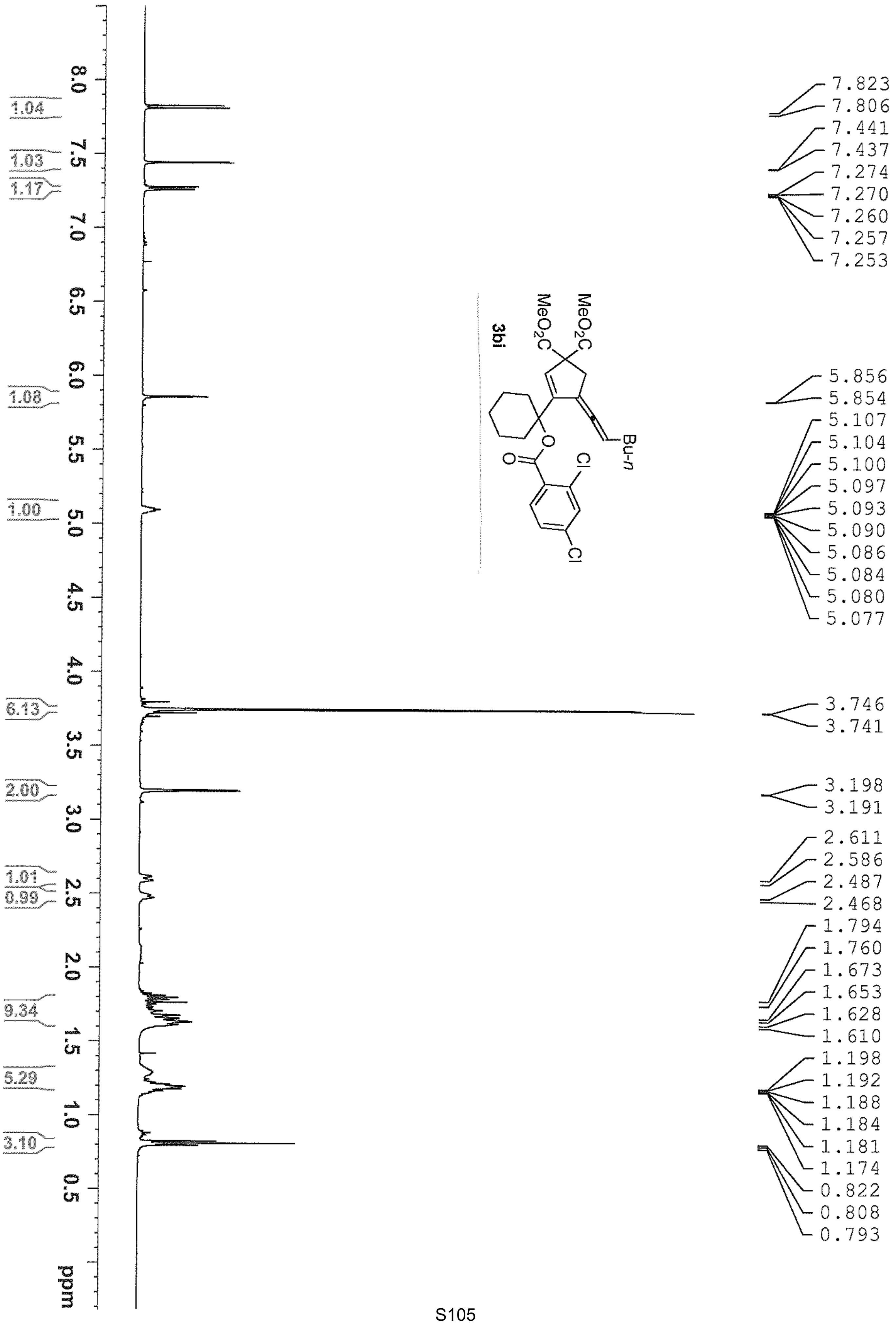

7.823
7.806
7.441
7.437
7.274
7.270
7.260
7.257
7.253
5.856
5.854
5.107
5.104
5.100
5.097
5.093
5.090
5.086
5.084
5.080
5.077
3.746
3.741
3.198
3.191
2.611
2.586
2.487
2.468
1.794
1.760
1.673
1.653
1.628
1.610
1.198
1.192
1.188
1.184
1.181
1.174
0.822
0.808
0.793
1.04
1.03
1.17
1.08
1.00
6.13
2.00
1.01
0.99
9.34
5.29
3.10
8.0
7.5
7.0
6.5
6.0
5.5
5.0
4.5
4.0
3.5
3.0
2.5
2.0
1.5
1.0
0.5
ppm
MeO2C
MeO2C
Bu-n
Cl
Cl
O
O
3bi

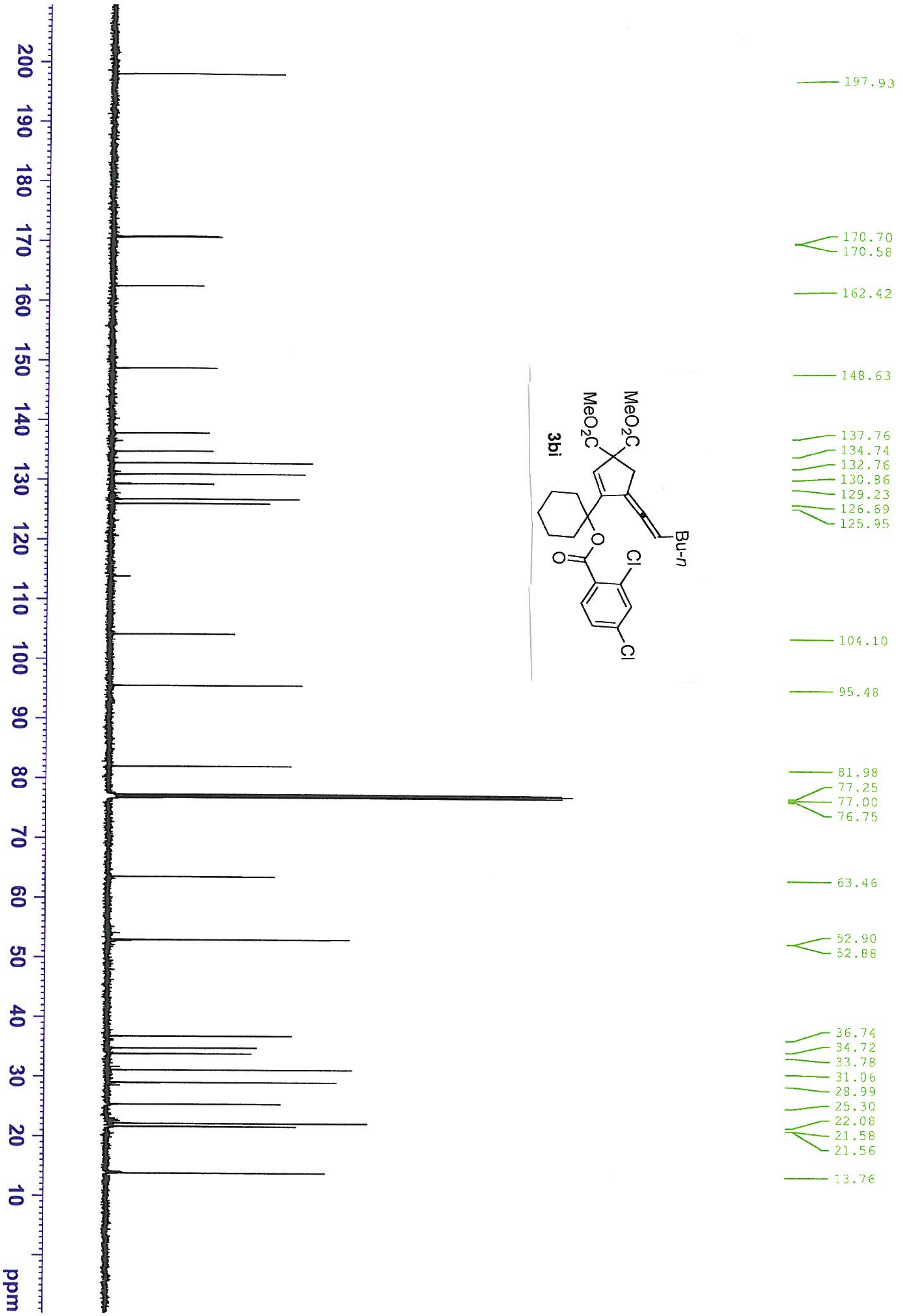
197.93
170.70
170.58
162.42
148.63
137.76
134.74
132.76
130.86
129.23
126.69
125.95
104.10
95.48
81.98
77.25
77.00
76.75
63.46
52.90
52.88
36.74
34.72
33.78
31.06
28.99
25.30
22.08
21.58
21.56
13.76
ppm
MeO2C
MeO2C
Bu-n
Cl
Cl
O
O
3bi

# Mass Spectrum SmartFormula Report

**Analysis Info**

| | | | |
|---|---|---|---|
| Analysis Name | H:\Data2\Youqian\dyq-4-66000001.d | Acquisition Date | 2012-08-15 15:27:56 |
| Method | tune_wide_dirk.m | Operator | pia |
| Sample Name | | Instrument / Ser# | micrOTOF 125 |
| Comment | | | |

**Acquisition Parameter**

| | | | | | |
|---|---|---|---|---|---|
| Source Type | ESI | Ion Polarity | Positive | Set Nebulizer | 0.4 Bar |
| Focus | Not active | | | Set Dry Heater | 180 °C |
| Scan Begin | 50 m/z | Set Capillary | 4500 V | Set Dry Gas | 4.0 l/min |
| Scan End | 3000 m/z | Set End Plate Offset | -500 V | Set Divert Valve | Source |

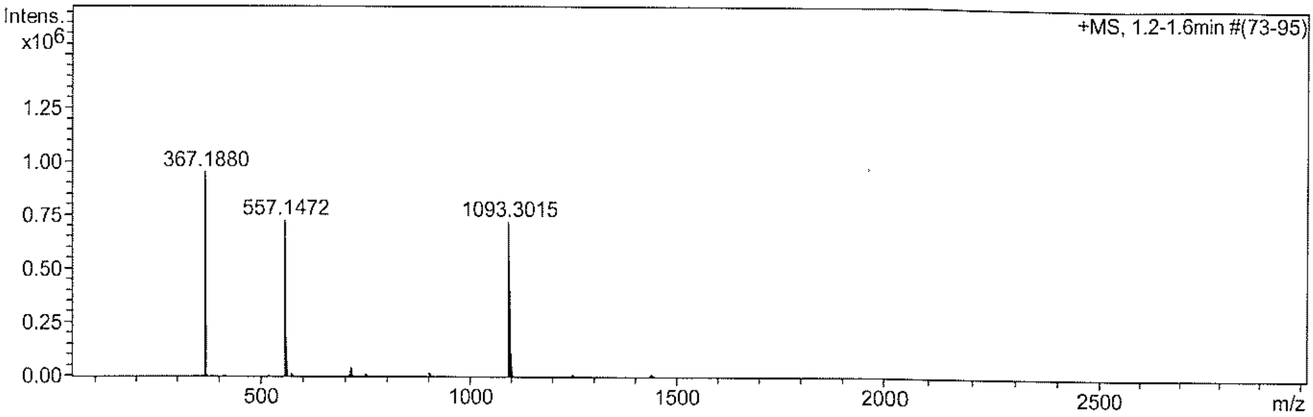


| Formula | Meas. m/z | m/z | err [ppm] | Mean err [ppm] |
|---|---|---|---|---|
| | 557.1472 | | | |
| C 28 H 32 Cl 2 Na O 6 | | 557.1468 | -0.6 | -0.8 |

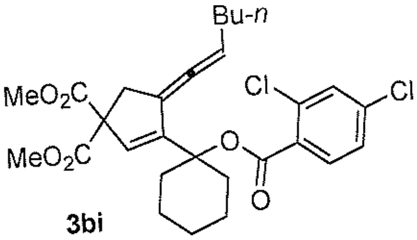


**3bi**

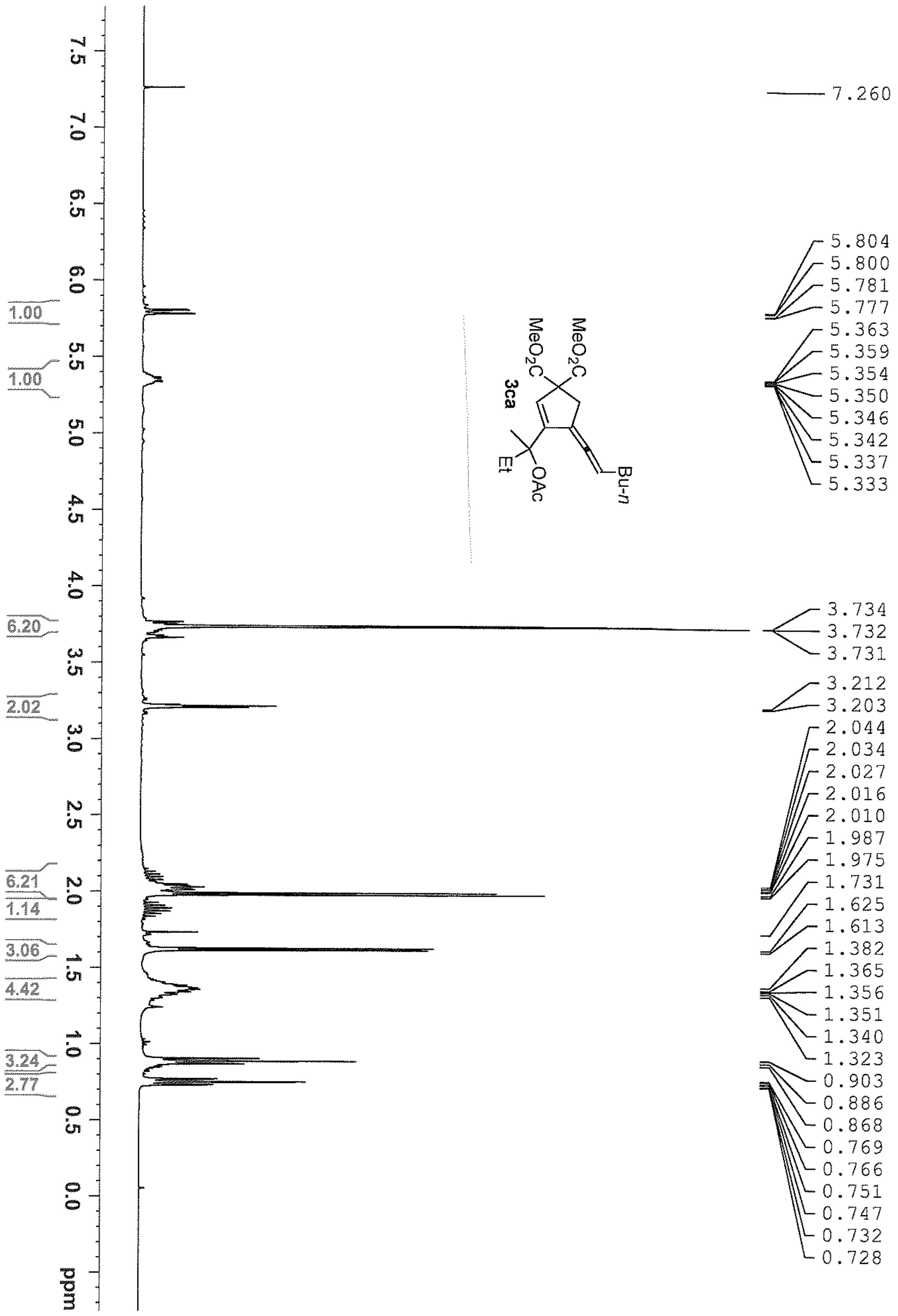
MeO2C
MeO2C
Bu-n
OAc
Et
3ca
7.260
5.804
5.800
5.781
5.777
5.363
5.359
5.354
5.350
5.346
5.342
5.337
5.333
3.734
3.732
3.731
3.212
3.203
2.044
2.034
2.027
2.016
2.010
1.987
1.975
1.731
1.625
1.613
1.382
1.365
1.356
1.351
1.340
1.323
0.903
0.886
0.868
0.769
0.766
0.751
0.747
0.732
0.728
1.00
1.00
6.20
2.02
6.21
1.14
3.06
4.42
3.24
2.77
7.5
7.0
6.5
6.0
5.5
5.0
4.5
4.0
3.5
3.0
2.5
2.0
1.5
1.0
0.5
0.0
ppm

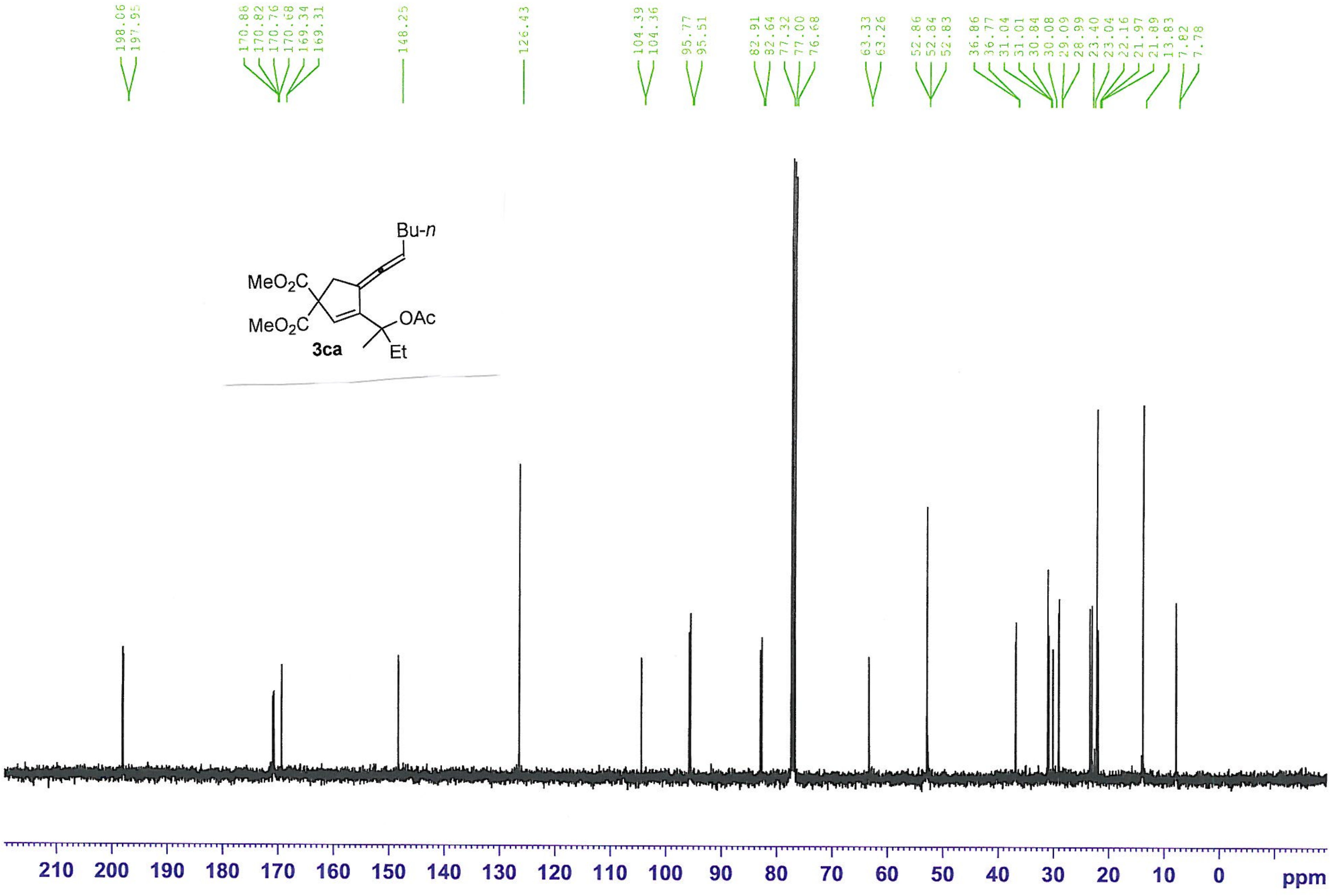
198.06
197.95
170.86
170.82
170.76
170.68
169.34
169.31
148.25
126.43
104.39
104.36
95.77
95.51
82.91
82.64
77.32
77.00
76.68
63.33
63.26
52.86
52.84
52.83
36.86
36.77
31.04
31.01
30.84
30.08
29.09
28.99
23.40
23.04
22.16
21.97
21.89
13.83
7.82
7.78
Bu-n
MeO2C
MeO2C
OAc
Et
3ca
210 200 190 180 170 160 150 140 130 120 110 100 90 80 70 60 50 40 30 20 10 0 ppm

# Mass Spectrum SmartFormula Report

**Analysis Info**

| | | | |
|---|---|---|---|
| Analysis Name | H:\Data2\Youqian\dyq-4-5000001.d | Acquisition Date | 2012-05-26 15:13:59 |
| Method | tune_wide_dirk.m | Operator | pia |
| Sample Name | dyq-4-5 | Instrument / Ser# | micrOTOF 125 |
| Comment | | | |

**Acquisition Parameter**

| | | | | | |
|---|---|---|---|---|---|
| Source Type | ESI | Ion Polarity | Positive | Set Nebulizer | 0.4 Bar |
| Focus | Not active | | | Set Dry Heater | 180 °C |
| Scan Begin | 50 m/z | Set Capillary | 4500 V | Set Dry Gas | 4.0 l/min |
| Scan End | 3000 m/z | Set End Plate Offset | -500 V | Set Divert Valve | Source |

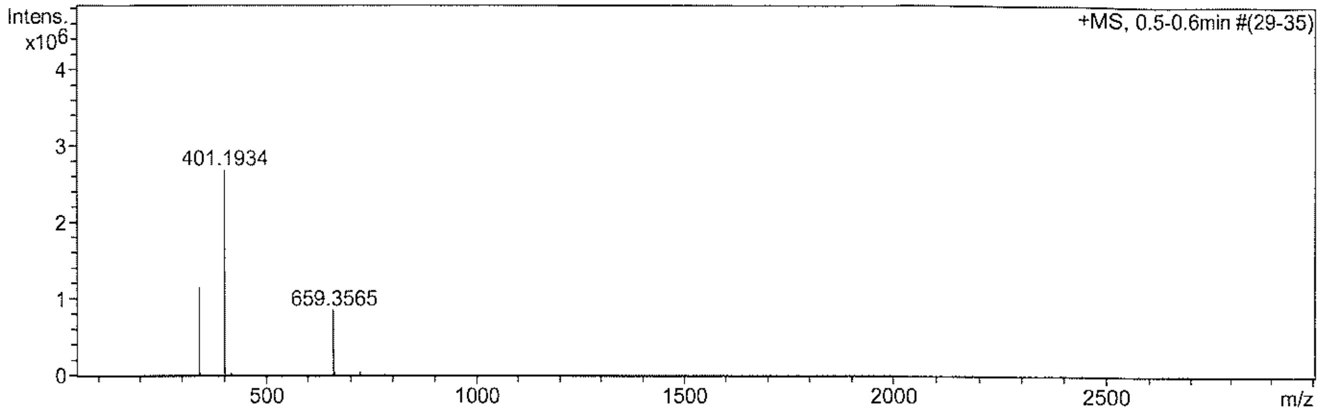


| Formula | Meas. m/z | m/z | err [ppm] | Mean err [ppm] |
|---|---|---|---|---|
| | 401.1934 | | | |
| C 21 H 30 Na O 6 | | 401.1935 | 0.3 | 1.7 |

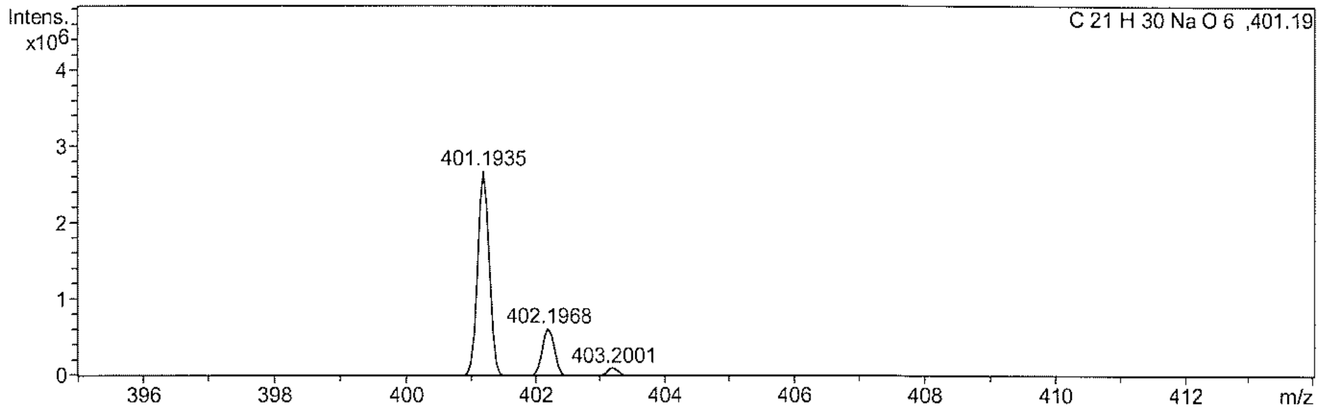


| Formula | Meas. m/z | m/z | err [ppm] | Mean err [ppm] |
|---|---|---|---|---|

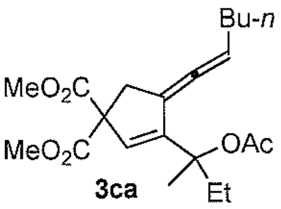

**3ca**

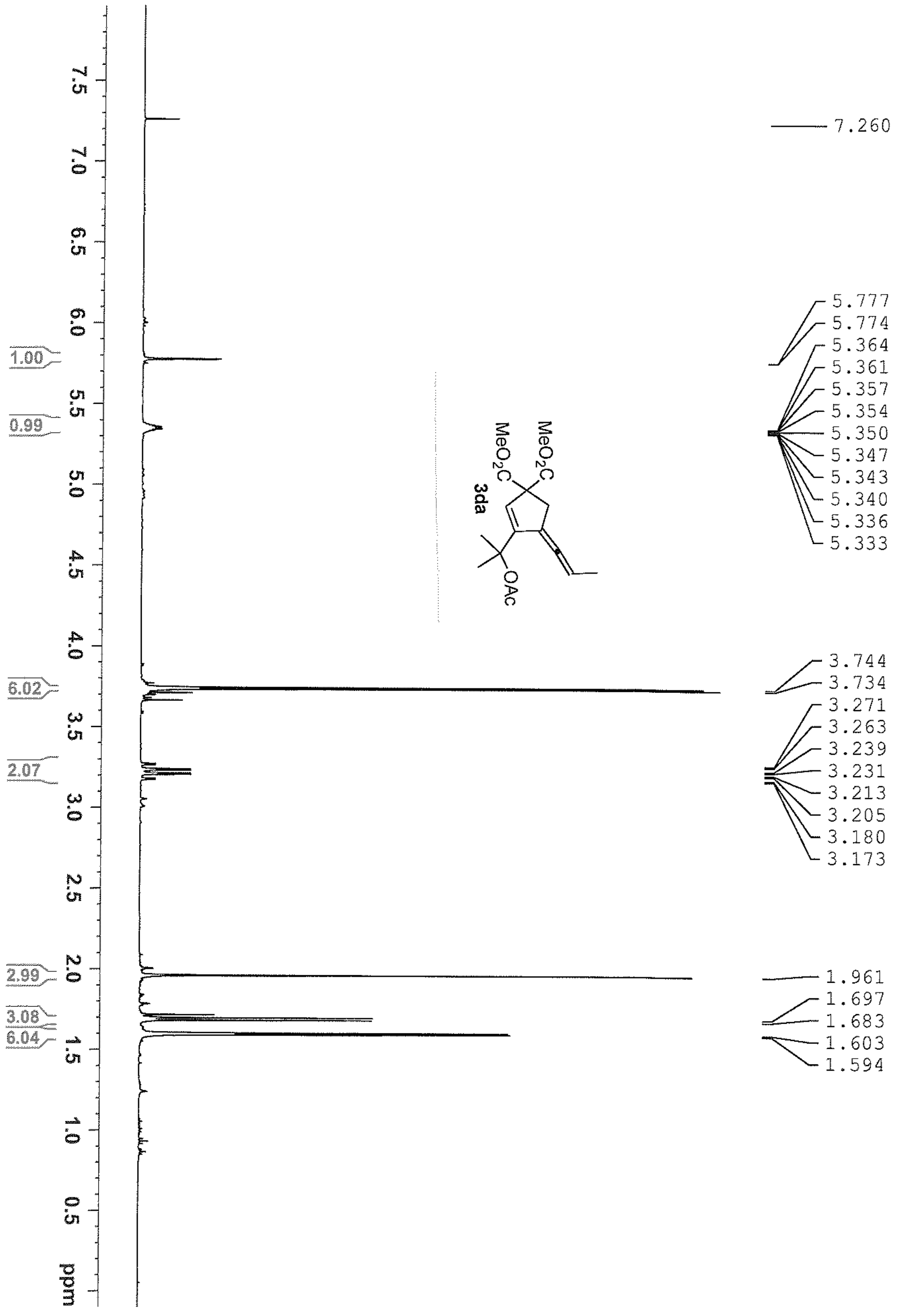
7.260
5.777
5.774
5.364
5.361
5.357
5.354
5.350
5.347
5.343
5.340
5.336
5.333
3.744
3.734
3.271
3.263
3.239
3.231
3.213
3.205
3.180
3.173
1.961
1.697
1.683
1.603
1.594
MeO2C
MeO2C
3da
OAc
1.00
0.99
6.02
2.07
2.99
3.08
6.04
7.5
7.0
6.5
6.0
5.5
5.0
4.5
4.0
3.5
3.0
2.5
2.0
1.5
1.0
0.5
ppm

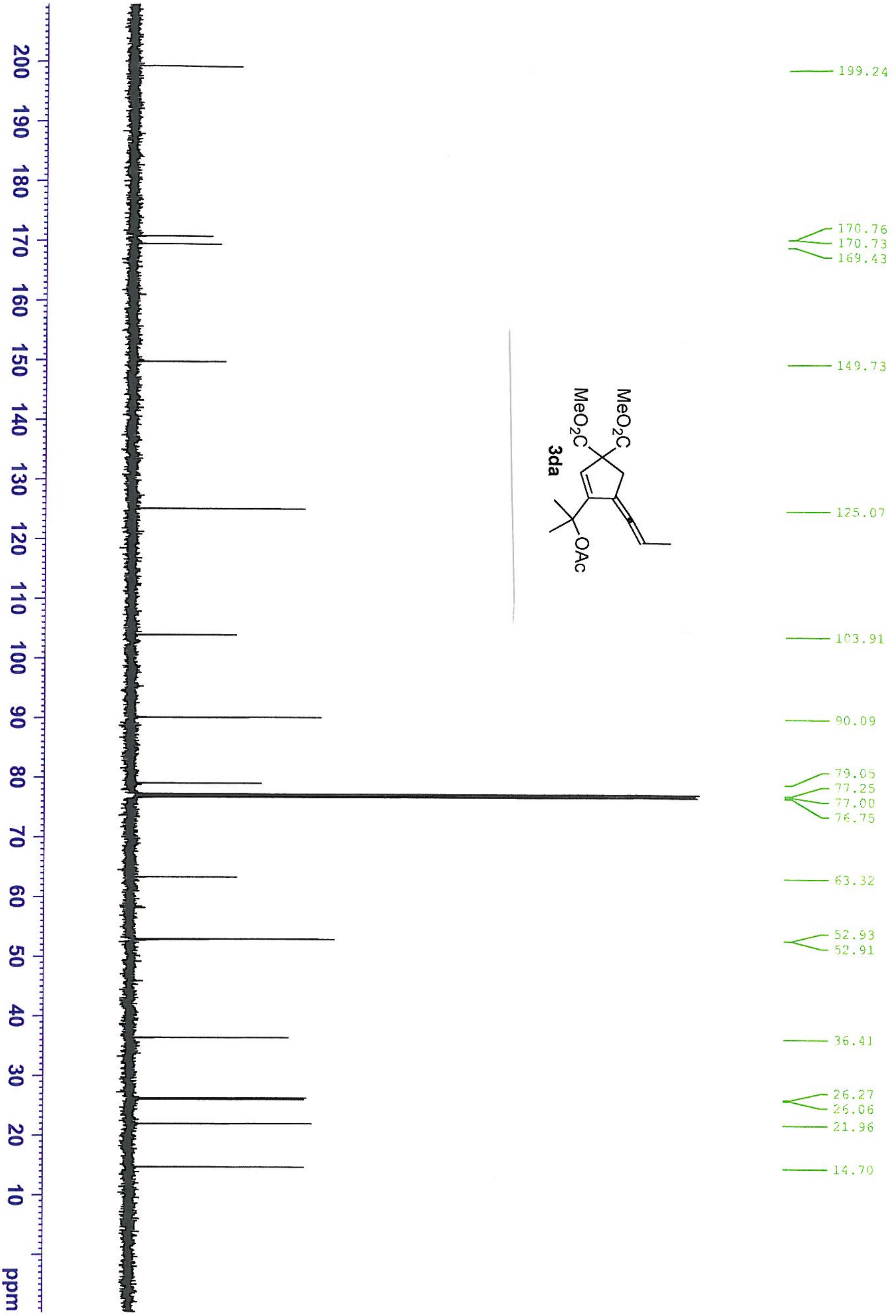
199.24
170.76
170.73
169.43
149.73
125.07
103.91
90.09
79.05
77.25
77.00
76.75
63.32
52.93
52.91
36.41
26.27
26.06
21.96
14.70
MeO2C
MeO2C
OAc
3da
ppm

# Mass Spectrum SmartFormula Report

**Analysis Info**

| | |
|---|---|
| Analysis Name | H:\Data2\Youqian\dyq-4-30000001.d |
| Method | tune_wide_dirk.m |
| Sample Name | dyq-4-30 |
| Comment | |
| Acquisition Date | 2012-06-29 18:14:58 |
| Operator | pia |
| Instrument / Ser# | micrOTOF 125 |

**Acquisition Parameter**

| | | | | | |
|---|---|---|---|---|---|
| Source Type | ESI | Ion Polarity | Positive | Set Nebulizer | 0.4 Bar |
| Focus | Not active | | | Set Dry Heater | 180 °C |
| Scan Begin | 50 m/z | Set Capillary | 4500 V | Set Dry Gas | 4.0 l/min |
| Scan End | 3000 m/z | Set End Plate Offset | -500 V | Set Divert Valve | Source |

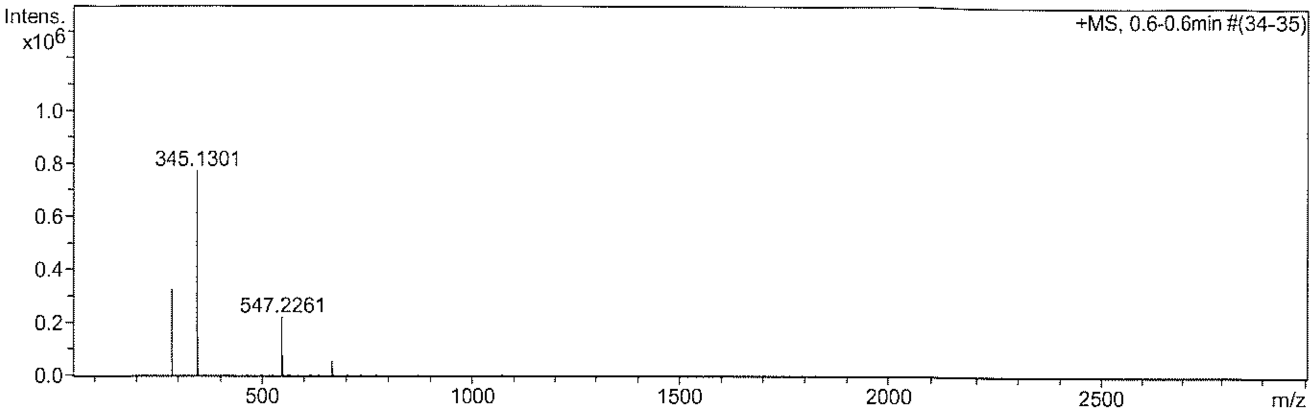


| Formula | Meas. m/z | m/z | err [ppm] | Mean err [ppm] |
|---|---|---|---|---|
| | 345.1301 | | | |
| C 17 H 22 Na O 6 | | 345.1309 | 2.1 | 2.4 |

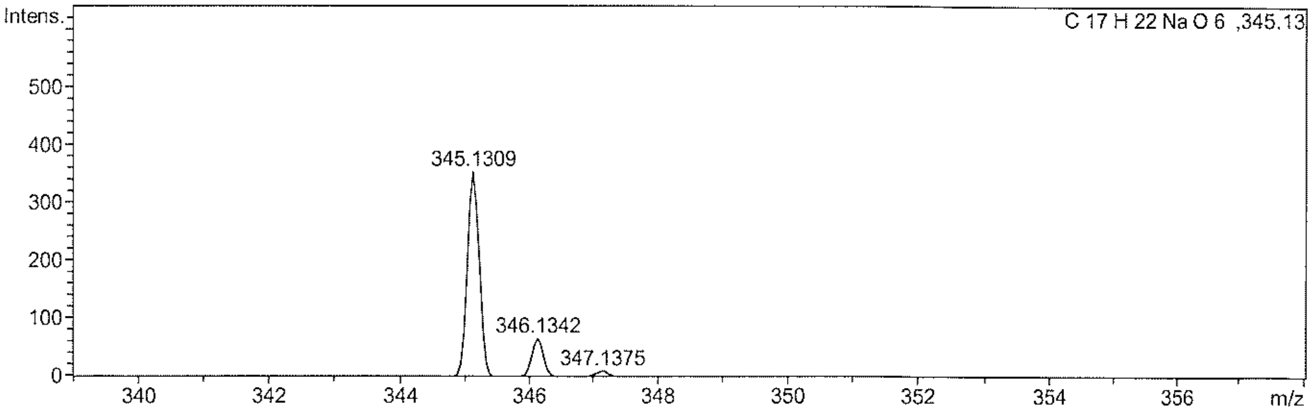


| Formula | Meas. m/z | m/z | err [ppm] | Mean err [ppm] |
|---|---|---|---|---|

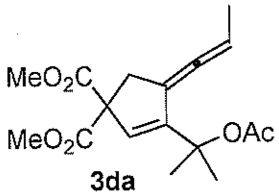


**3da**

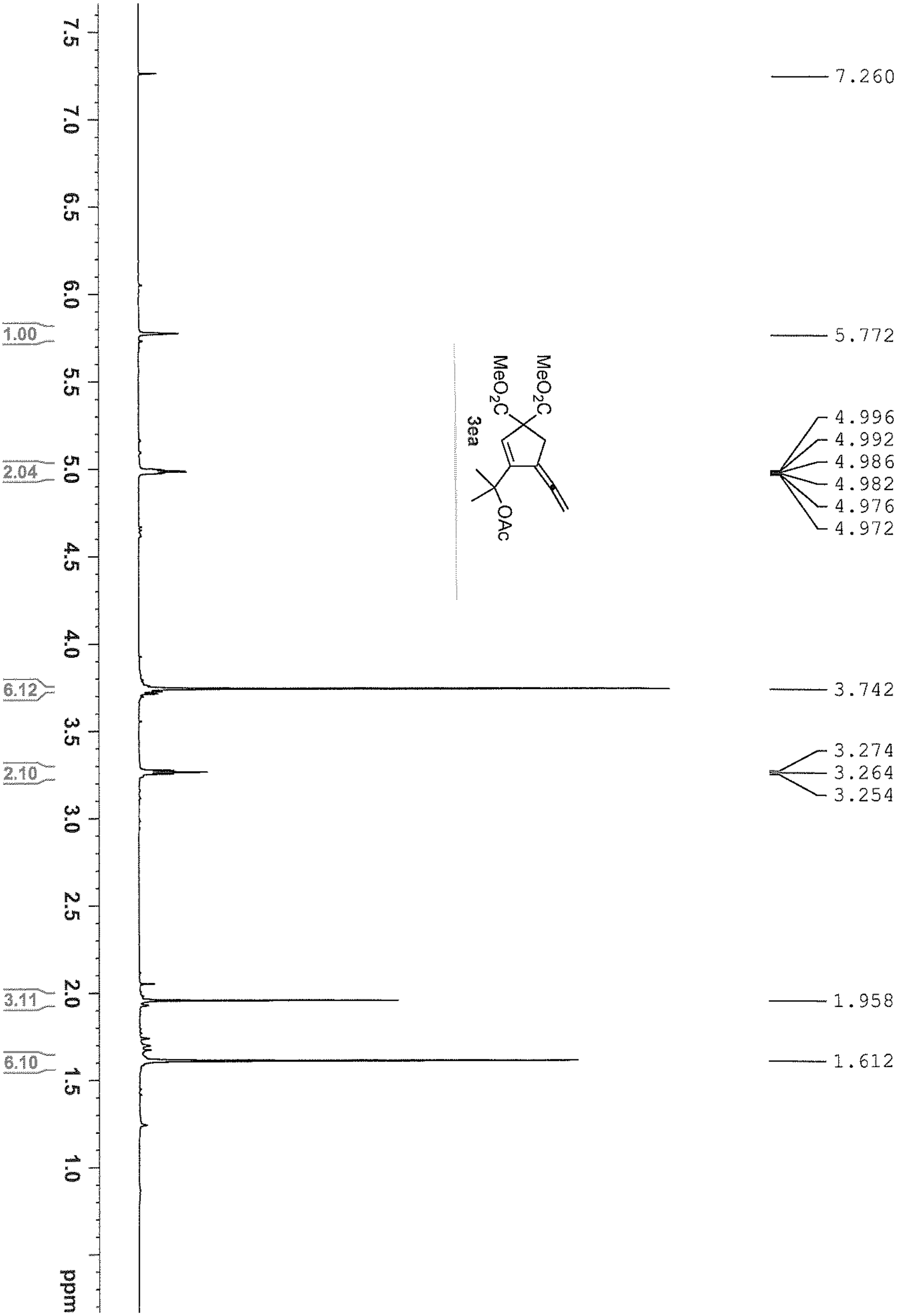

7.260
5.772
4.996
4.992
4.986
4.982
4.976
4.972
3.742
3.274
3.264
3.254
1.958
1.612
1.00
2.04
6.12
2.10
3.11
6.10
7.5
7.0
6.5
6.0
5.5
5.0
4.5
4.0
3.5
3.0
2.5
2.0
1.5
1.0
ppm
MeO2C
MeO2C
OAc
3ea

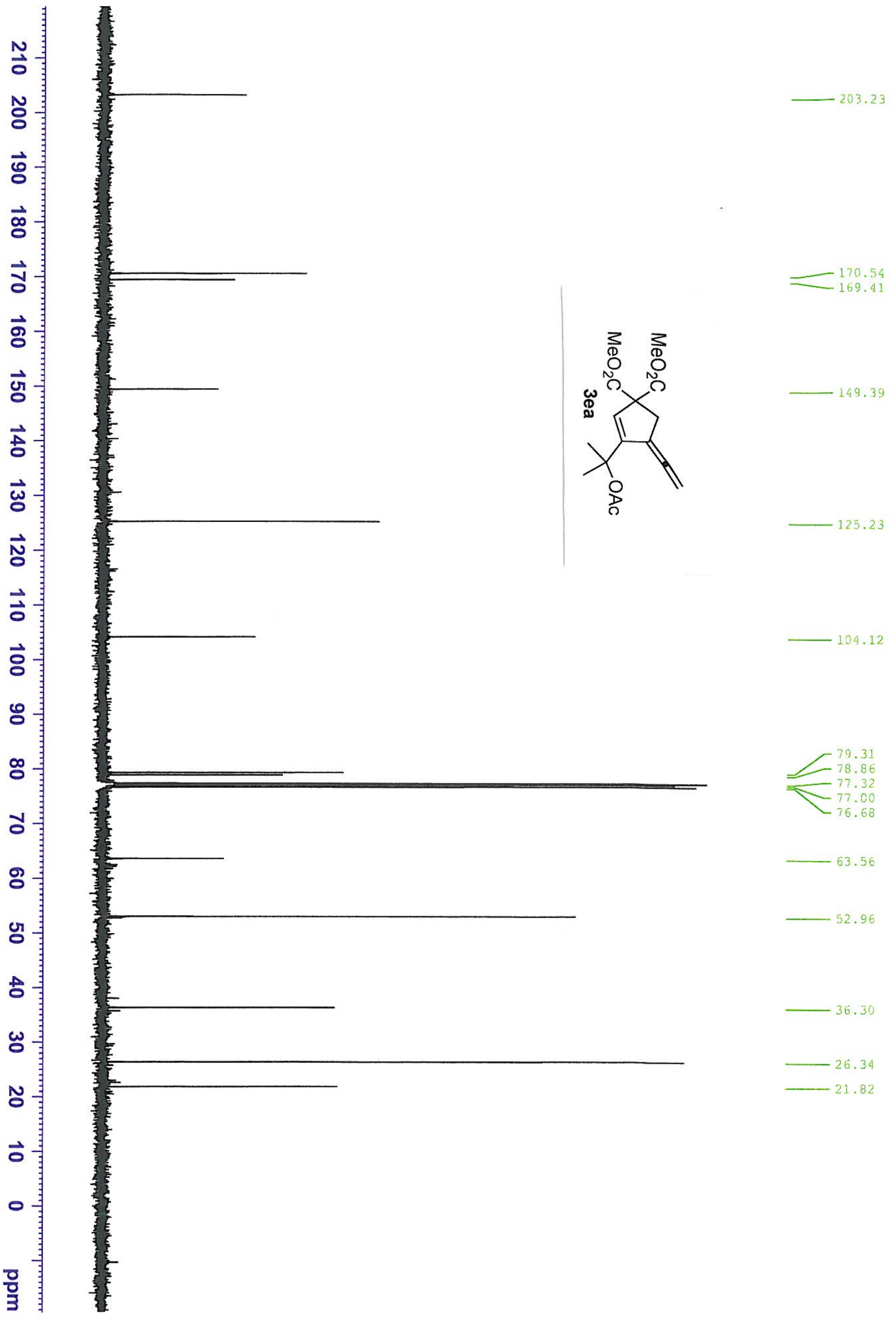
203.23
170.54
169.41
149.39
125.23
104.12
79.31
78.86
77.32
77.00
76.68
63.56
52.96
36.30
26.34
21.82
MeO2C
MeO2C
OAc
3ea
210 200 190 180 170 160 150 140 130 120 110 100 90 80 70 60 50 40 30 20 10 0
ppm

# Mass Spectrum SmartFormula Report

**Analysis Info**

| | |
|---|---|
| Analysis Name | H:\Data2\Youqian\dyq-3-172000001.d |
| Method | tune_wide_dirk.m |
| Sample Name | dyq-3-172 |
| Comment | |
| Acquisition Date | 2012-04-27 18:54:55 |
| Operator | pia |
| Instrument / Ser# | micrOTOF 125 |

**Acquisition Parameter**

| | | | | | |
|---|---|---|---|---|---|
| Source Type | ESI | Ion Polarity | Positive | Set Nebulizer | 0.4 Bar |
| Focus | Not active | | | Set Dry Heater | 180 °C |
| Scan Begin | 50 m/z | Set Capillary | 4500 V | Set Dry Gas | 4.0 l/min |
| Scan End | 3000 m/z | Set End Plate Offset | -500 V | Set Divert Valve | Source |

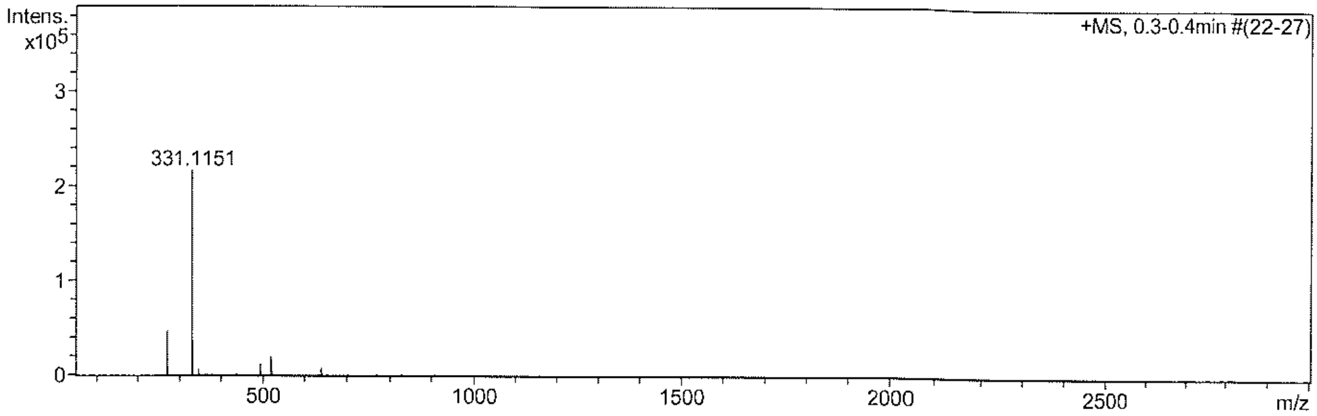


| Formula | Meas. m/z | m/z | err [ppm] | Mean err [ppm] |
|---|---|---|---|---|
| | 331.1151 | | | |
| C 16 H 20 Na O 6 | | 331.1152 | 0.2 | -0.0 |

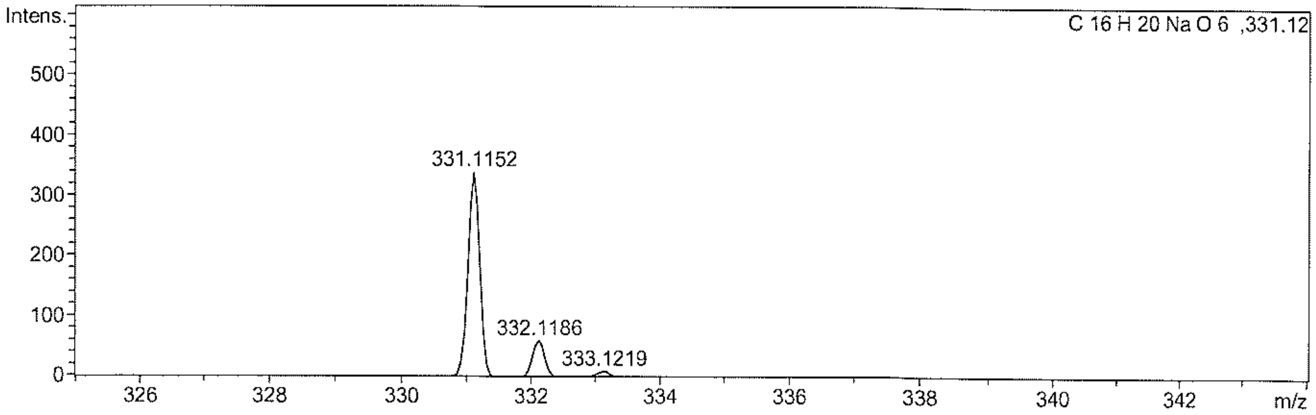


| Formula | Meas. m/z | m/z | err [ppm] | Mean err [ppm] |
|---|---|---|---|---|

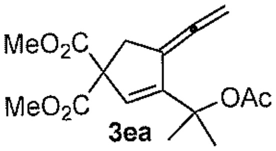


**3ea**

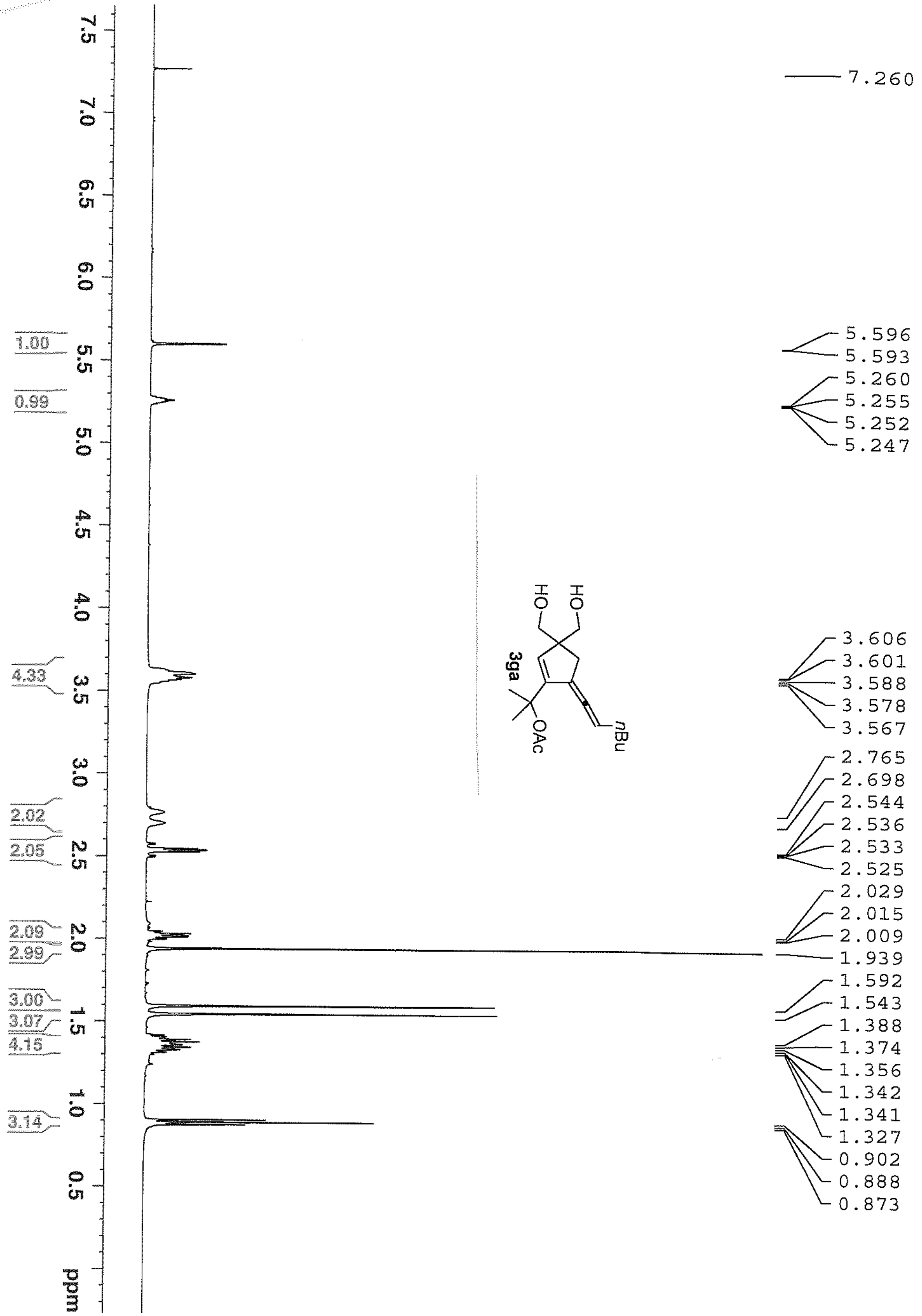
7.260
5.596
5.593
5.260
5.255
5.252
5.247
3.606
3.601
3.588
3.578
3.567
2.765
2.698
2.544
2.536
2.533
2.525
2.029
2.015
2.009
1.939
1.592
1.543
1.388
1.374
1.356
1.342
1.341
1.327
0.902
0.888
0.873
1.00
0.99
4.33
2.02
2.05
2.09
2.99
3.00
3.07
4.15
3.14
ppm
HO
HO
3ga
OAc
nBu

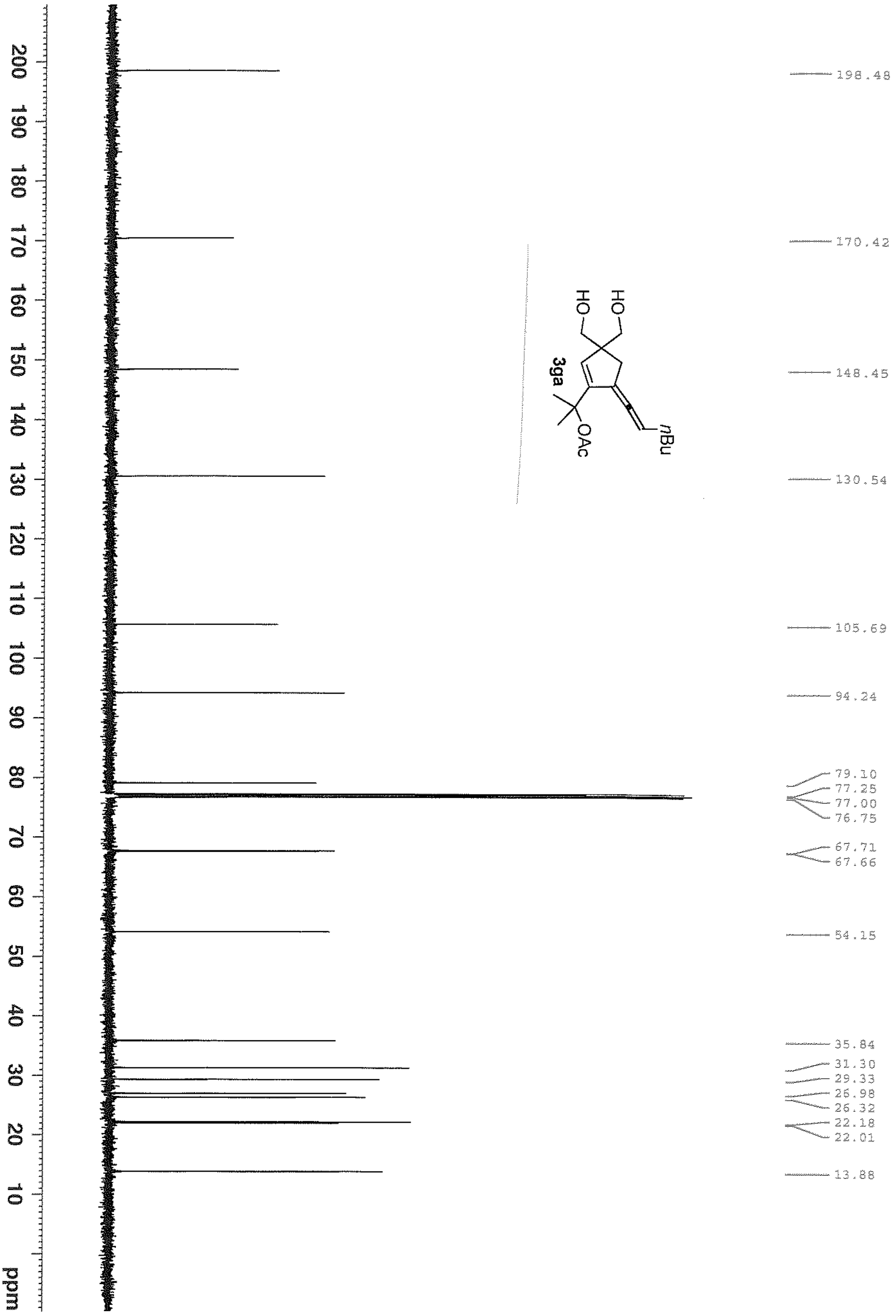
198.48
170.42
148.45
130.54
105.69
94.24
79.10
77.25
77.00
76.75
67.71
67.66
54.15
35.84
31.30
29.33
26.98
26.32
22.18
22.01
13.88
ppm
HO
HO
OAc
nBu
3ga

# Mass Spectrum SmartFormula Report

**Analysis Info**

| | | | |
|---|---|---|---|
| Analysis Name | E:\Data2\Youqian\dyq-4-199000001.d | Acquisition Date | 2012-12-11 21:08:50 |
| Method | Tune_low_pos.m | Operator | Carin Larsson |
| Sample Name | dyq-4-199 | Instrument / Ser# | micrOTOF 125 |
| Comment | | | |

**Acquisition Parameter**

| | | | | | |
|---|---|---|---|---|---|
| Source Type | ESI | Ion Polarity | Positive | Set Nebulizer | 0.4 Bar |
| Focus | Not active | | | Set Dry Heater | 180 °C |
| Scan Begin | 50 m/z | Set Capillary | 4000 V | Set Dry Gas | 4.0 l/min |
| Scan End | 1000 m/z | Set End Plate Offset | -500 V | Set Divert Valve | Source |

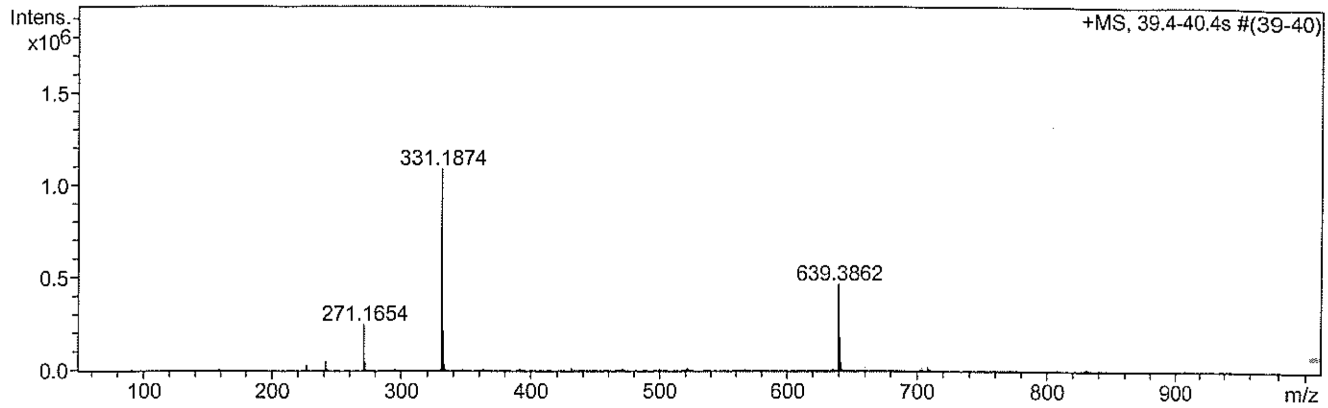


| Formula | Meas. m/z | m/z | err [ppm] | Mean err [ppm] |
|---|---|---|---|---|
| C 18 H 28 Na O 4 | 331.1874 | 331.1880 | 1.8 | 1.8 |

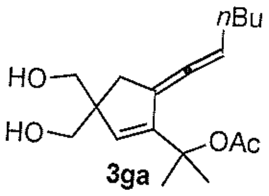


**3ga**

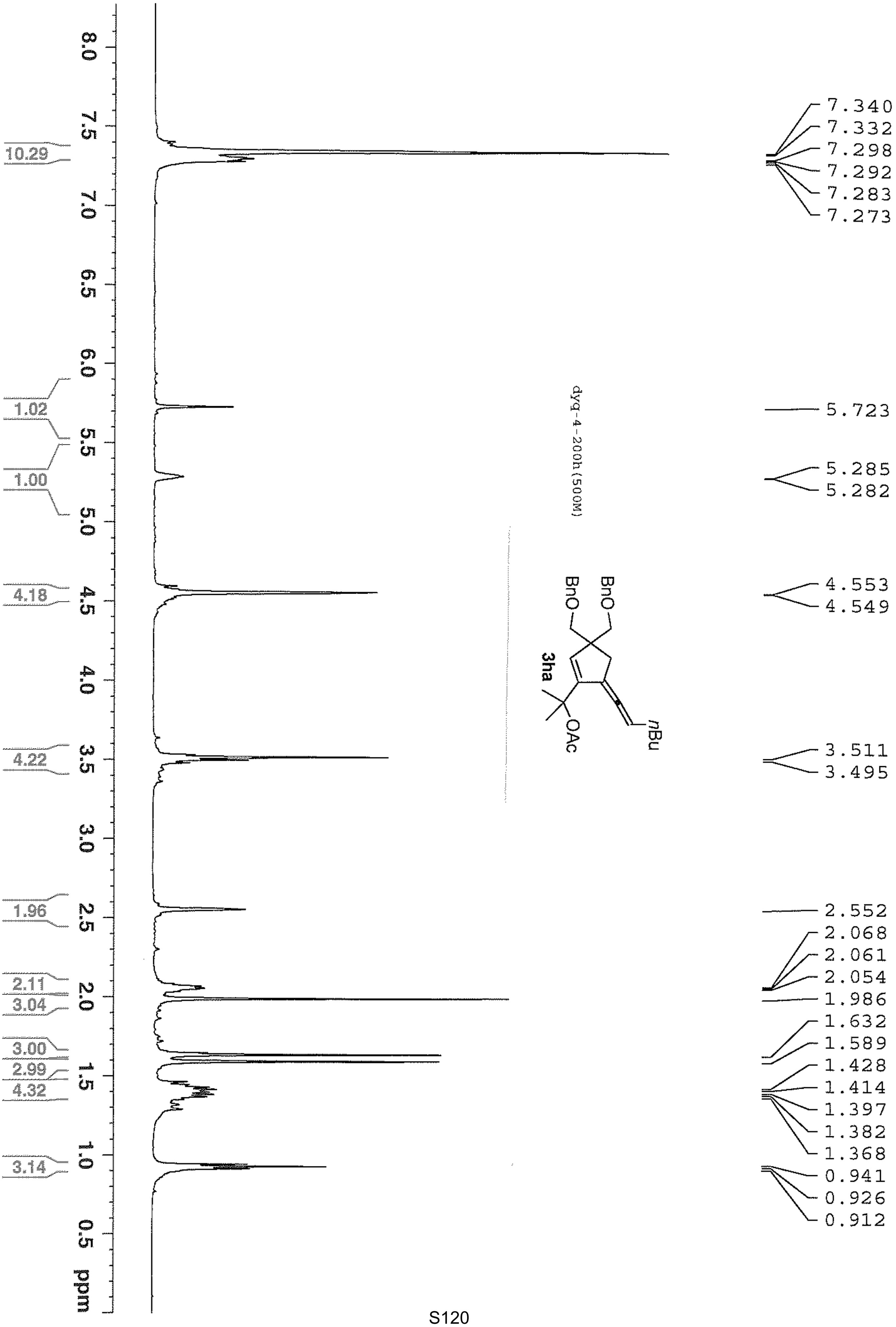
dyq-4-200h(500M)
BnO
BnO
nBu
OAc
3ha
7.340
7.332
7.298
7.292
7.283
7.273
5.723
5.285
5.282
4.553
4.549
3.511
3.495
2.552
2.068
2.061
2.054
1.986
1.632
1.589
1.428
1.414
1.397
1.382
1.368
0.941
0.926
0.912
10.29
1.02
1.00
4.18
4.22
1.96
2.11
3.04
3.00
2.99
4.32
3.14
8.0
7.5
7.0
6.5
6.0
5.5
5.0
4.5
4.0
3.5
3.0
2.5
2.0
1.5
1.0
0.5
ppm

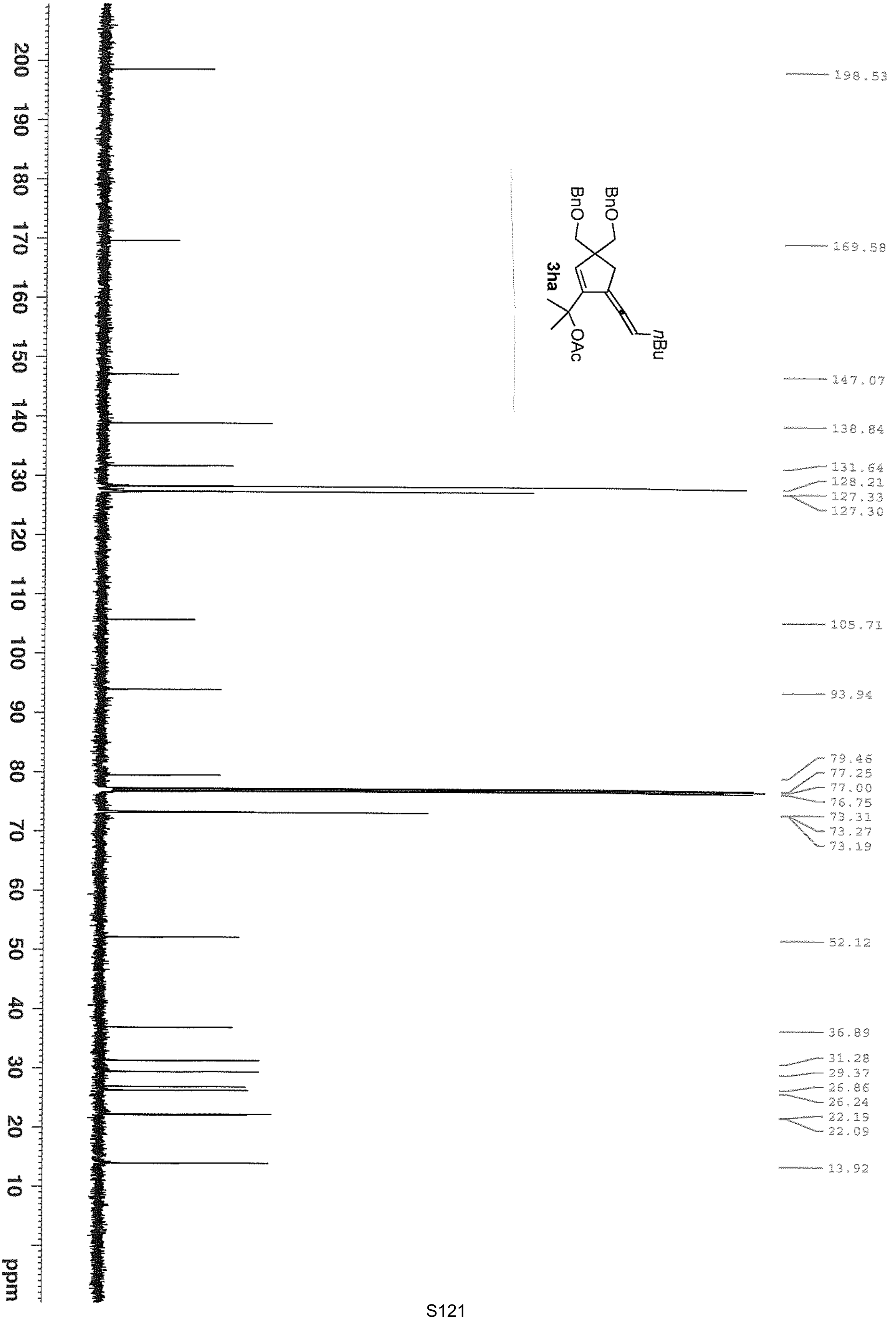
198.53
169.58
147.07
138.84
131.64
128.21
127.33
127.30
105.71
93.94
79.46
77.25
77.00
76.75
73.31
73.27
73.19
52.12
36.89
31.28
29.37
26.86
26.24
22.19
22.09
13.92
BnO
BnO
nBu
OAc
3ha
ppm

# Mass Spectrum SmartFormula Report

**Analysis Info**

| | | | |
|---|---|---|---|
| Analysis Name | E:\Data2\Youqian\dyq-4-200000001.d | Acquisition Date | 2012-12-11 21:13:14 |
| Method | Tune_low_pos.m | Operator | Carin Larsson |
| Sample Name | dyq-4-200 | Instrument / Ser# | micrOTOF 125 |
| Comment | | | |

**Acquisition Parameter**

| | | | | | |
|---|---|---|---|---|---|
| Source Type | ESI | Ion Polarity | Positive | Set Nebulizer | 0.4 Bar |
| Focus | Not active | | | Set Dry Heater | 180 °C |
| Scan Begin | 50 m/z | Set Capillary | 4000 V | Set Dry Gas | 4.0 l/min |
| Scan End | 1000 m/z | Set End Plate Offset | -500 V | Set Divert Valve | Source |

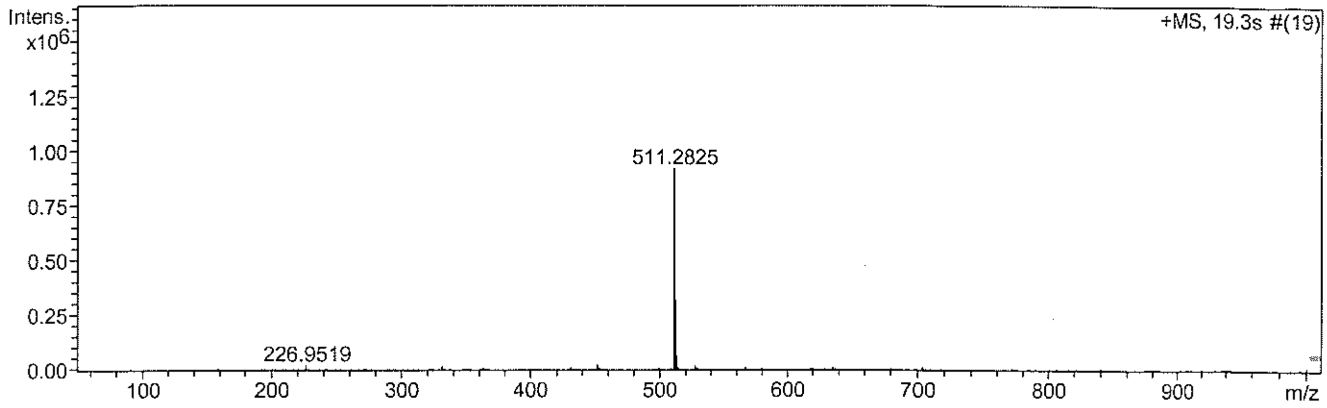


| Formula | Meas. m/z | m/z | err [ppm] | Mean err [ppm] |
|---|---|---|---|---|
| C 32 H 40 Na O 4 | 511.2825 | 511.2819 | -1.2 | -0.9 |

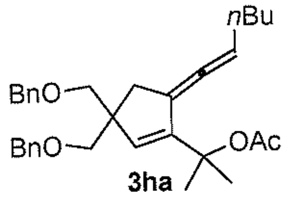


**3ha**

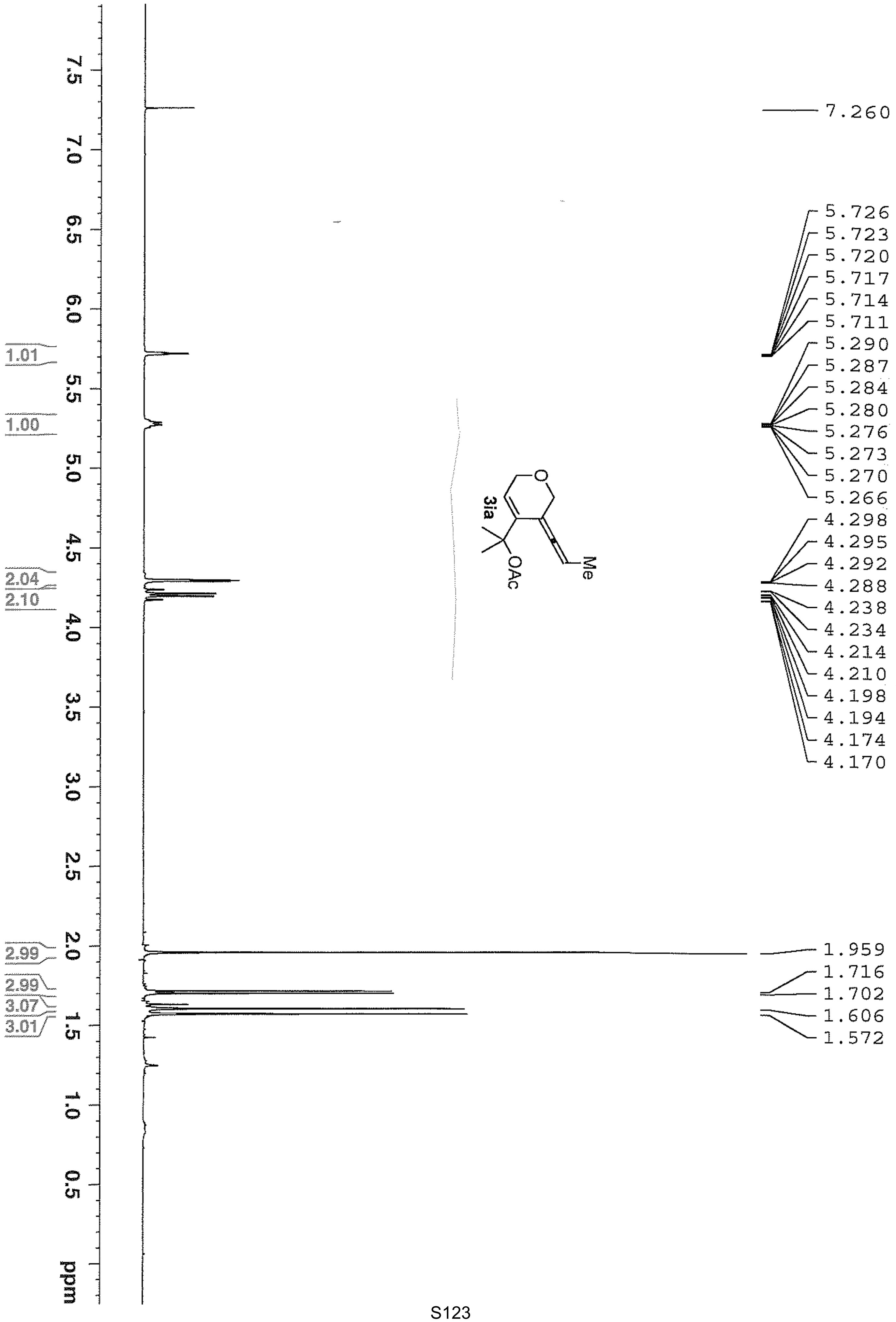
3ia
OAc
Me
O
7.260
5.726
5.723
5.720
5.717
5.714
5.711
5.290
5.287
5.284
5.280
5.276
5.273
5.270
5.266
4.298
4.295
4.292
4.288
4.238
4.234
4.214
4.210
4.198
4.194
4.174
4.170
1.959
1.716
1.702
1.606
1.572
1.01
1.00
2.04
2.10
2.99
2.99
3.07
3.01
7.5
7.0
6.5
6.0
5.5
5.0
4.5
4.0
3.5
3.0
2.5
2.0
1.5
1.0
0.5
ppm

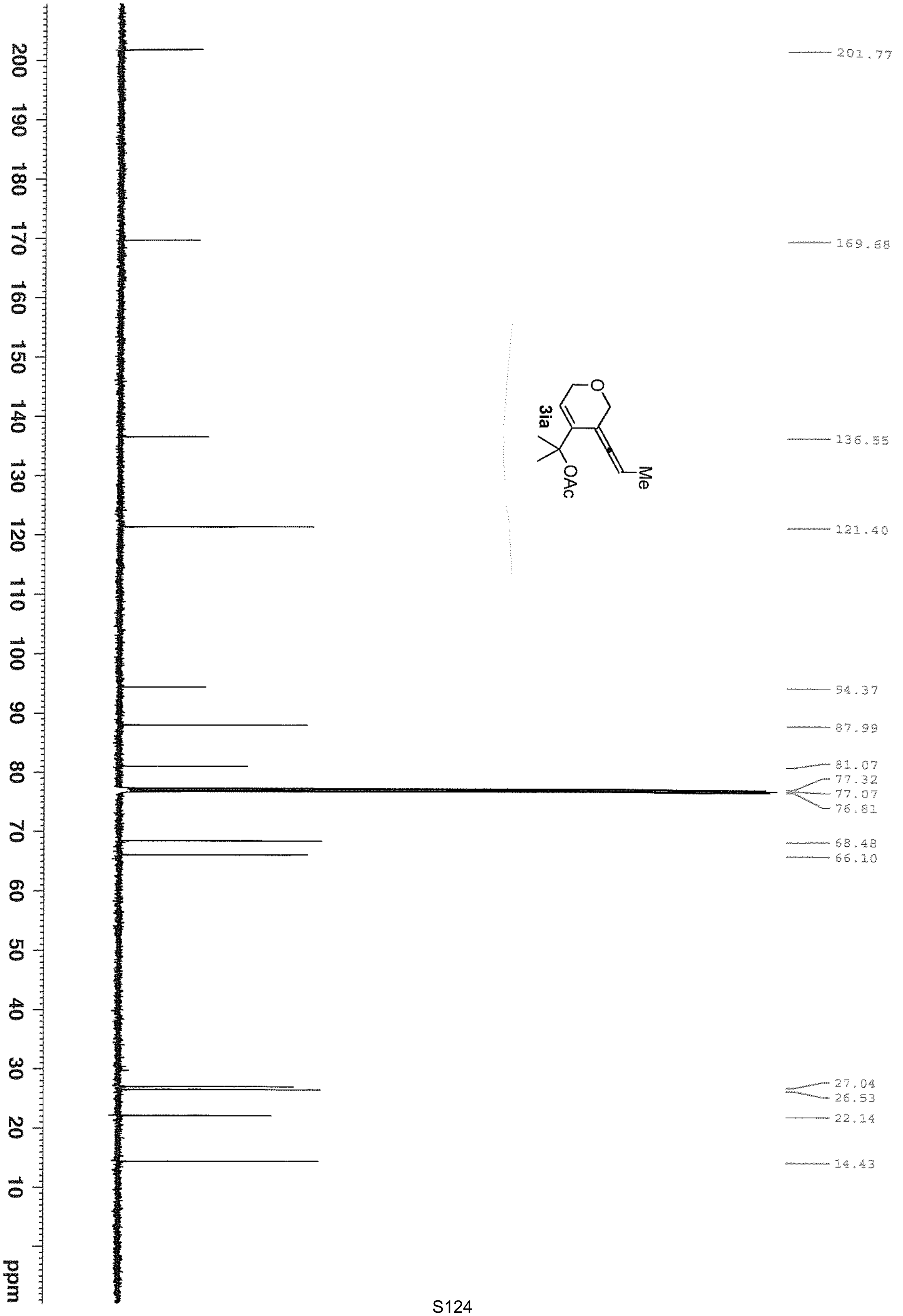
201.77
169.68
136.55
121.40
94.37
87.99
81.07
77.32
77.07
76.81
68.48
66.10
27.04
26.53
22.14
14.43
3ia
Me
OAc
ppm

# Mass Spectrum SmartFormula Report

**Analysis Info**

| | | | |
|---|---|---|---|
| Analysis Name | E:\Data2\Youqian\dyq-4-171000002.d | Acquisition Date | 2012-11-29 16:24:30 |
| Method | Tune_low_pos.m | Operator | Carin Larsson |
| Sample Name | dyq-4-171 | Instrument / Ser# | micrOTOF 125 |
| Comment | | | |

**Acquisition Parameter**

| | | | | | |
|---|---|---|---|---|---|
| Source Type | ESI | Ion Polarity | Positive | Set Nebulizer | 0.4 Bar |
| Focus | Not active | | | Set Dry Heater | 180 °C |
| Scan Begin | 50 m/z | Set Capillary | 4000 V | Set Dry Gas | 4.0 l/min |
| Scan End | 1000 m/z | Set End Plate Offset | -500 V | Set Divert Valve | Source |

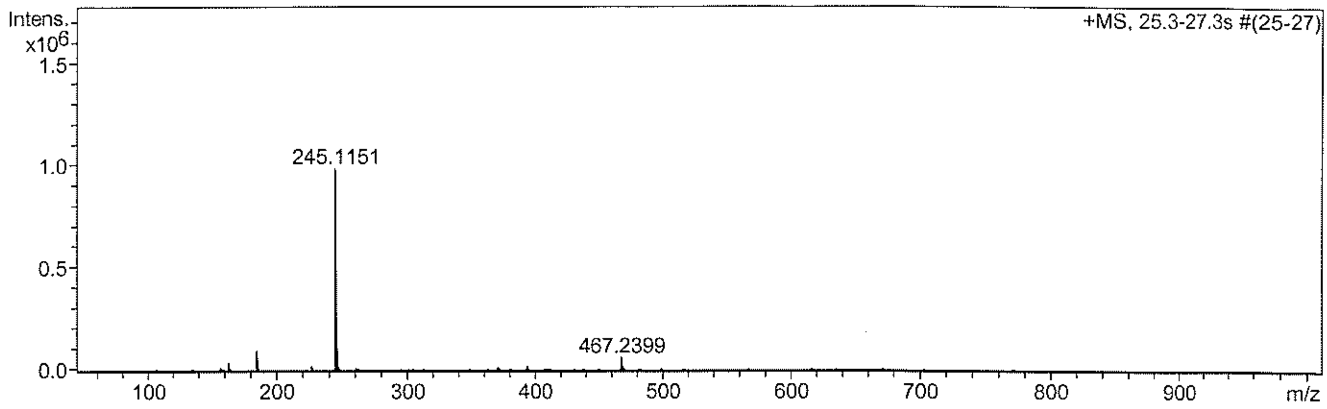


| Formula | Meas. m/z | m/z | err [ppm] | Mean err [ppm] |
|---|---|---|---|---|
| C 13 H 18 Na O 3 | 245.1151 | 245.1148 | -1.1 | -0.7 |

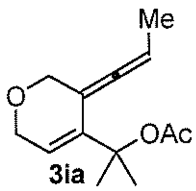


**3ia**

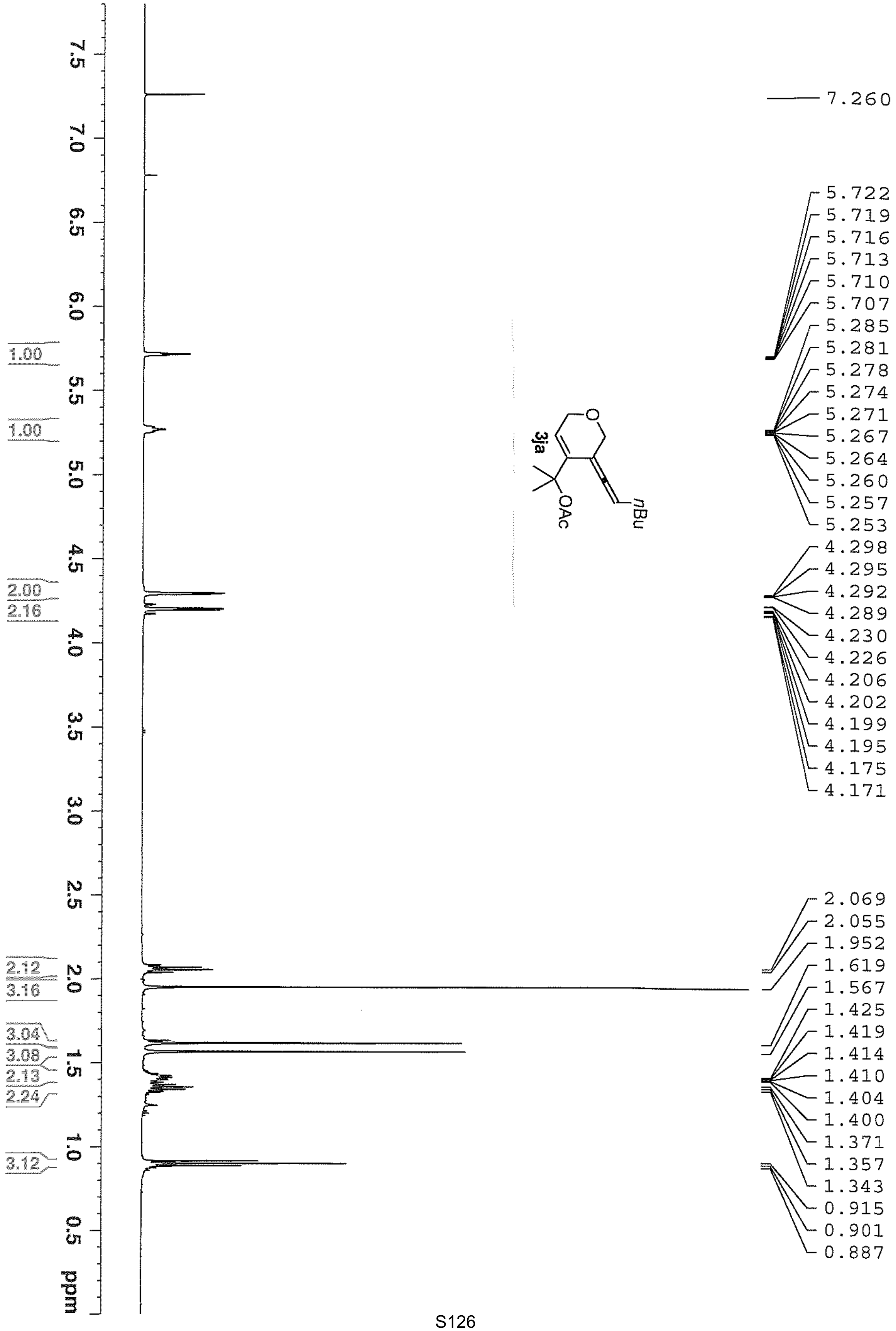
3ja
O
nBu
OAc
7.260
5.722
5.719
5.716
5.713
5.710
5.707
5.285
5.281
5.278
5.274
5.271
5.267
5.264
5.260
5.257
5.253
4.298
4.295
4.292
4.289
4.230
4.226
4.206
4.202
4.199
4.195
4.175
4.171
2.069
2.055
1.952
1.619
1.567
1.425
1.419
1.414
1.410
1.404
1.400
1.371
1.357
1.343
0.915
0.901
0.887
1.00
1.00
2.00
2.16
2.12
3.16
3.04
3.08
2.13
2.24
3.12
7.5
7.0
6.5
6.0
5.5
5.0
4.5
4.0
3.5
3.0
2.5
2.0
1.5
1.0
0.5
ppm

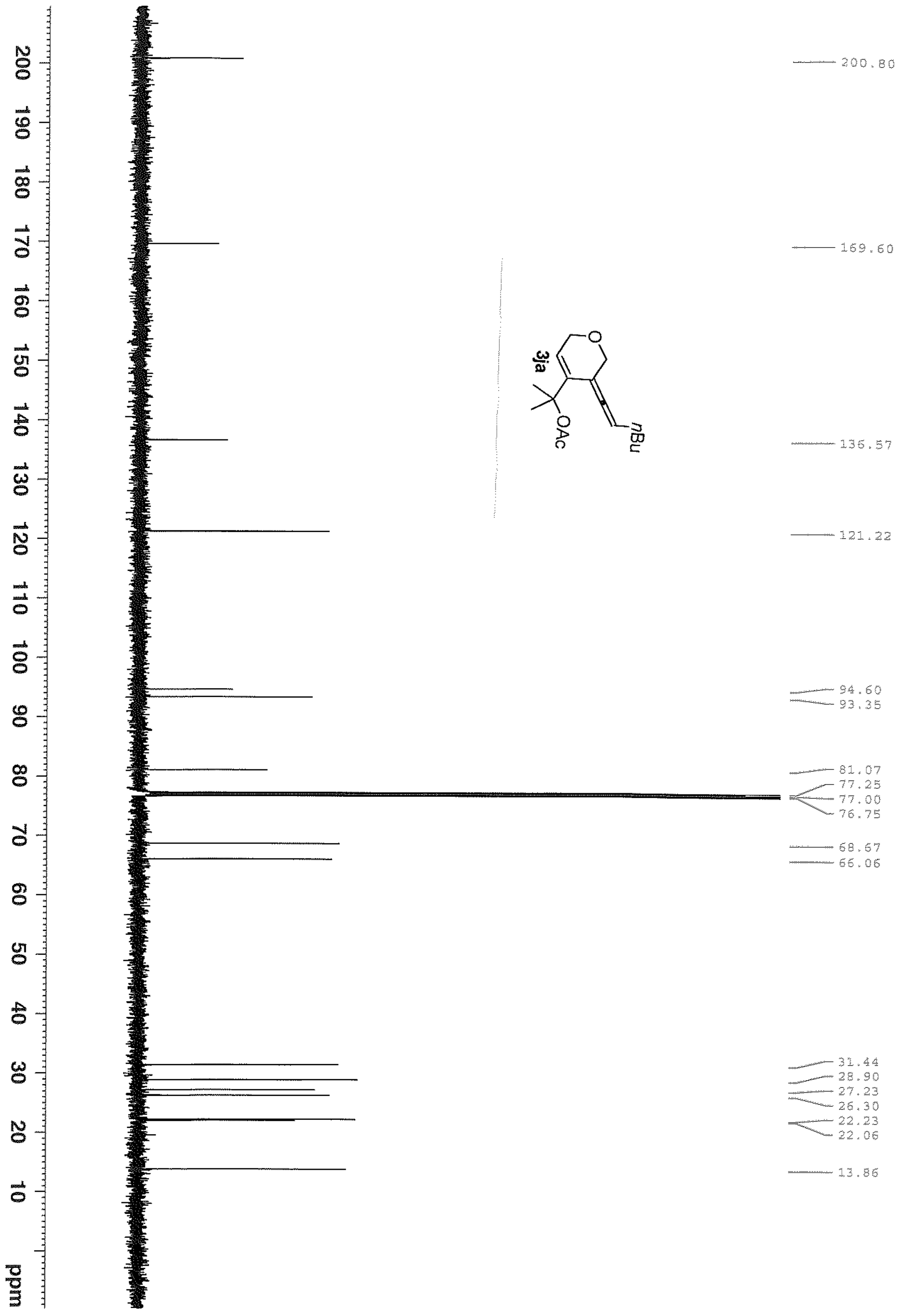
200.80
169.60
136.57
121.22
94.60
93.35
81.07
77.25
77.00
76.75
68.67
66.06
31.44
28.90
27.23
26.30
22.23
22.06
13.86
3ja
OAc
nBu
ppm

# Mass Spectrum SmartFormula Report

**Analysis Info**

| | | | |
|---|---|---|---|
| Analysis Name | E:\Data2\Youqian\dyq-4-191000001.d | Acquisition Date | 2012-12-07 21:41:53 |
| Method | Tune_low_pos.m | Operator | Carin Larsson |
| Sample Name | dyq-4-191 | Instrument / Ser# | micrOTOF 125 |
| Comment | | | |

**Acquisition Parameter**

| | | | | | |
|---|---|---|---|---|---|
| Source Type | ESI | Ion Polarity | Positive | Set Nebulizer | 0.4 Bar |
| Focus | Not active | | | Set Dry Heater | 180 °C |
| Scan Begin | 50 m/z | Set Capillary | 4000 V | Set Dry Gas | 4.0 l/min |
| Scan End | 1000 m/z | Set End Plate Offset | -500 V | Set Divert Valve | Source |

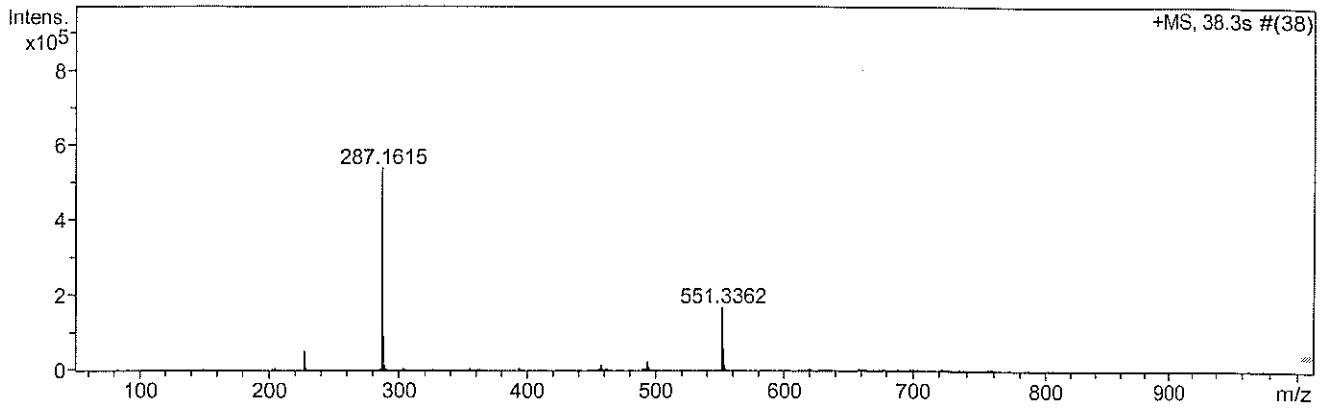


| Formula | Meas. m/z | m/z | err [ppm] | Mean err [ppm] |
|---|---|---|---|---|
| C 16 H 24 Na O 3 | 287.1615 | 287.1618 | 0.8 | 0.8 |

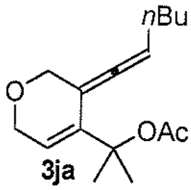


**3ja**

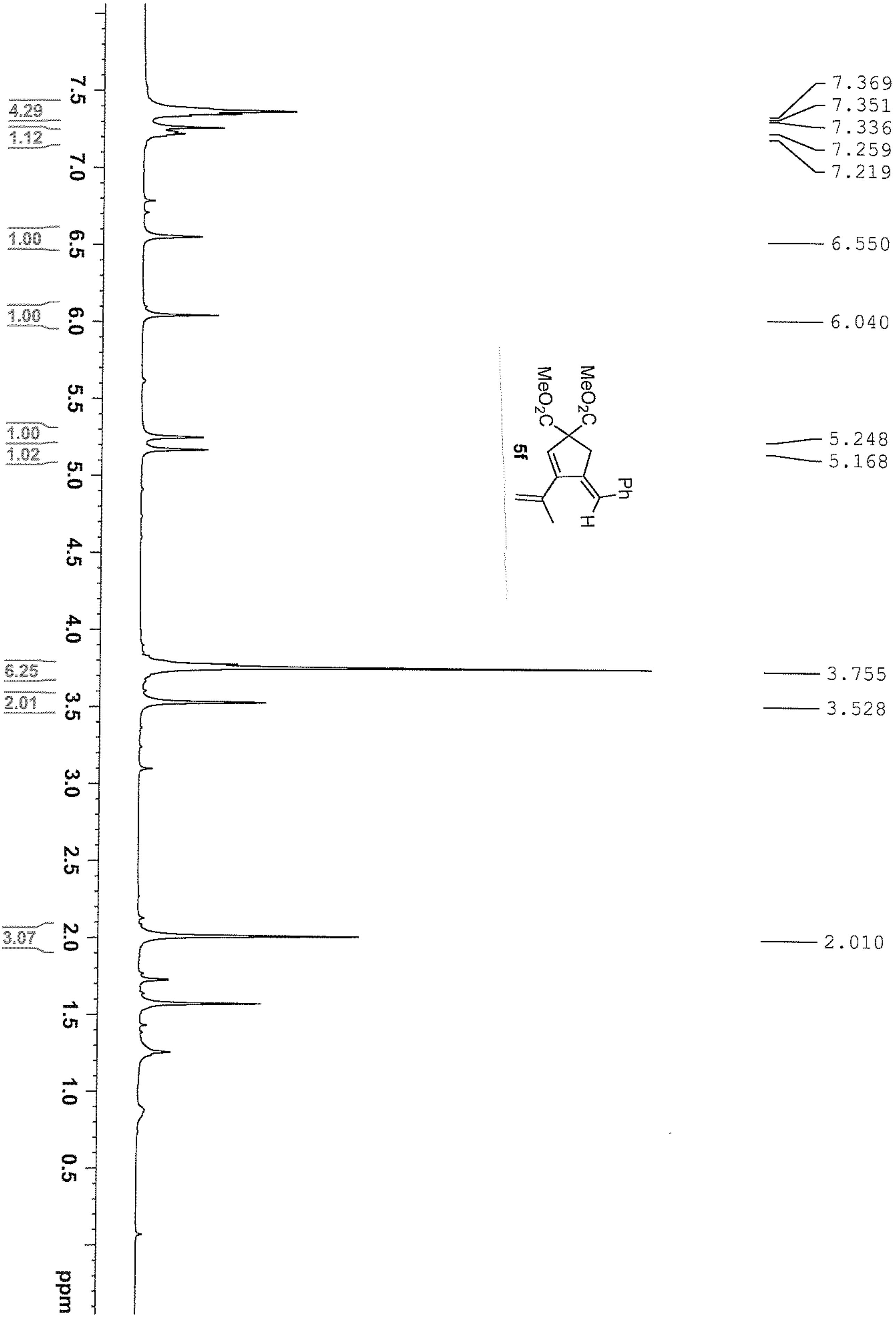
7.369
7.351
7.336
7.259
7.219
6.550
6.040
5.248
5.168
3.755
3.528
2.010
4.29
1.12
1.00
1.00
1.00
1.02
6.25
2.01
3.07
7.5
7.0
6.5
6.0
5.5
5.0
4.5
4.0
3.5
3.0
2.5
2.0
1.5
1.0
0.5
ppm
MeO2C
MeO2C
Ph
H
5f

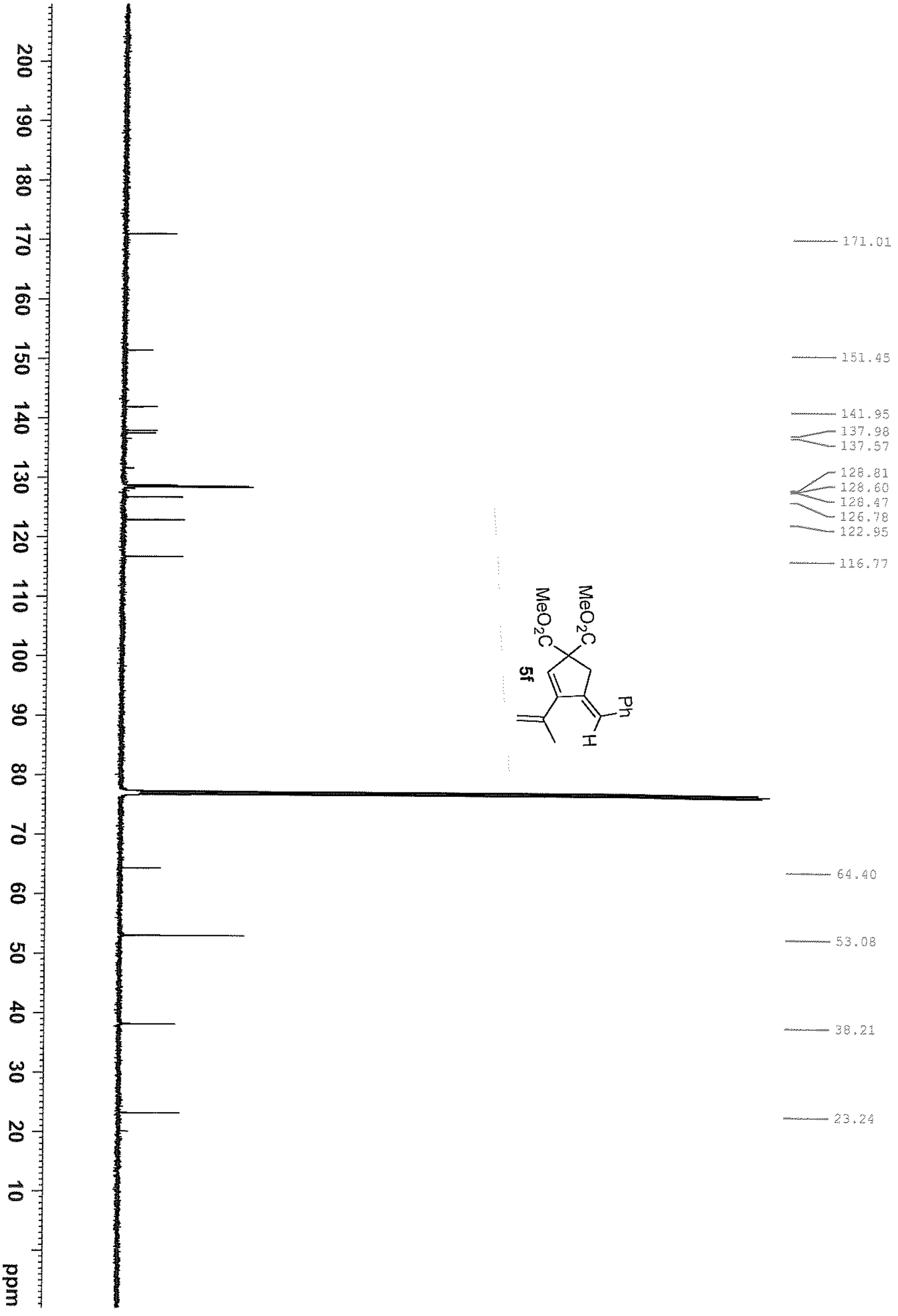
171.01
151.45
141.95
137.98
137.57
128.81
128.60
128.47
126.78
122.95
116.77
64.40
53.08
38.21
23.24
MeO2C
MeO2C
Ph
H
5f
200
190
180
170
160
150
140
130
120
110
100
90
80
70
60
50
40
30
20
10
ppm

# Mass Spectrum SmartFormula Report

**Analysis Info**

| | |
|---|---|
| Analysis Name | H:\Data2\Youqian\dyq-4-80-1000001.d |
| Method | tune_low_dirk.m |
| Sample Name | dyq-4-80-1 |
| Comment | |

| | |
|---|---|
| Acquisition Date | 2012-09-24 15:18:12 |
| Operator | pia |
| Instrument / Ser# | micrOTOF 125 |

**Acquisition Parameter**

| | | | | | |
|---|---|---|---|---|---|
| Source Type | ESI | Ion Polarity | Positive | Set Nebulizer | 0.4 Bar |
| Focus | Not active | | | Set Dry Heater | 170 °C |
| Scan Begin | 50 m/z | Set Capillary | 4500 V | Set Dry Gas | 4.0 l/min |
| Scan End | 1000 m/z | Set End Plate Offset | -500 V | Set Divert Valve | Source |

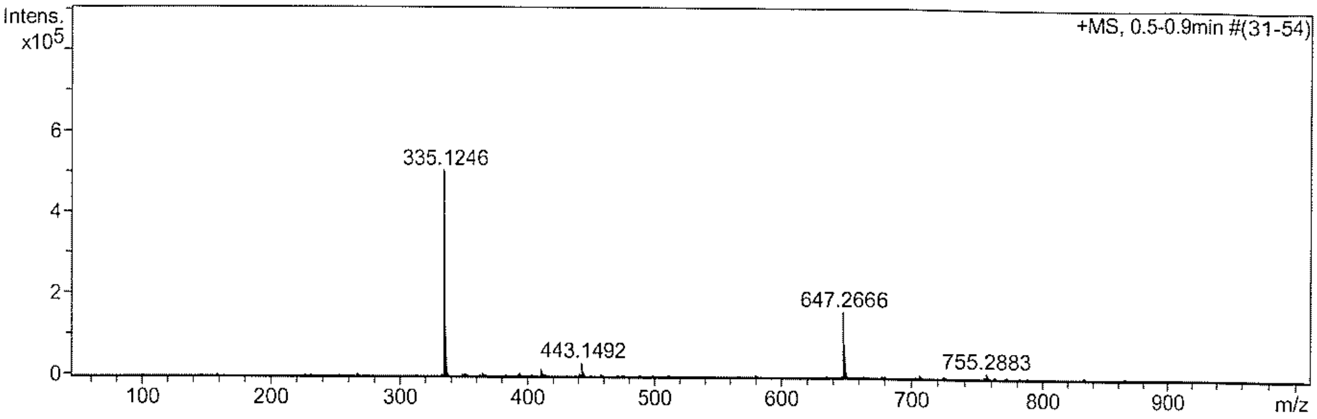


| Formula | Meas. m/z | m/z | err [ppm] | Mean err [ppm] |
|---|---|---|---|---|
| | 335.1246 | | | |
| C 19 H 20 Na O 4 | | 335.1254 | 2.2 | 2.6 |

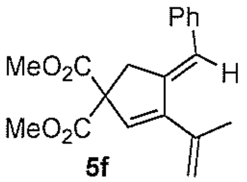


**5f**

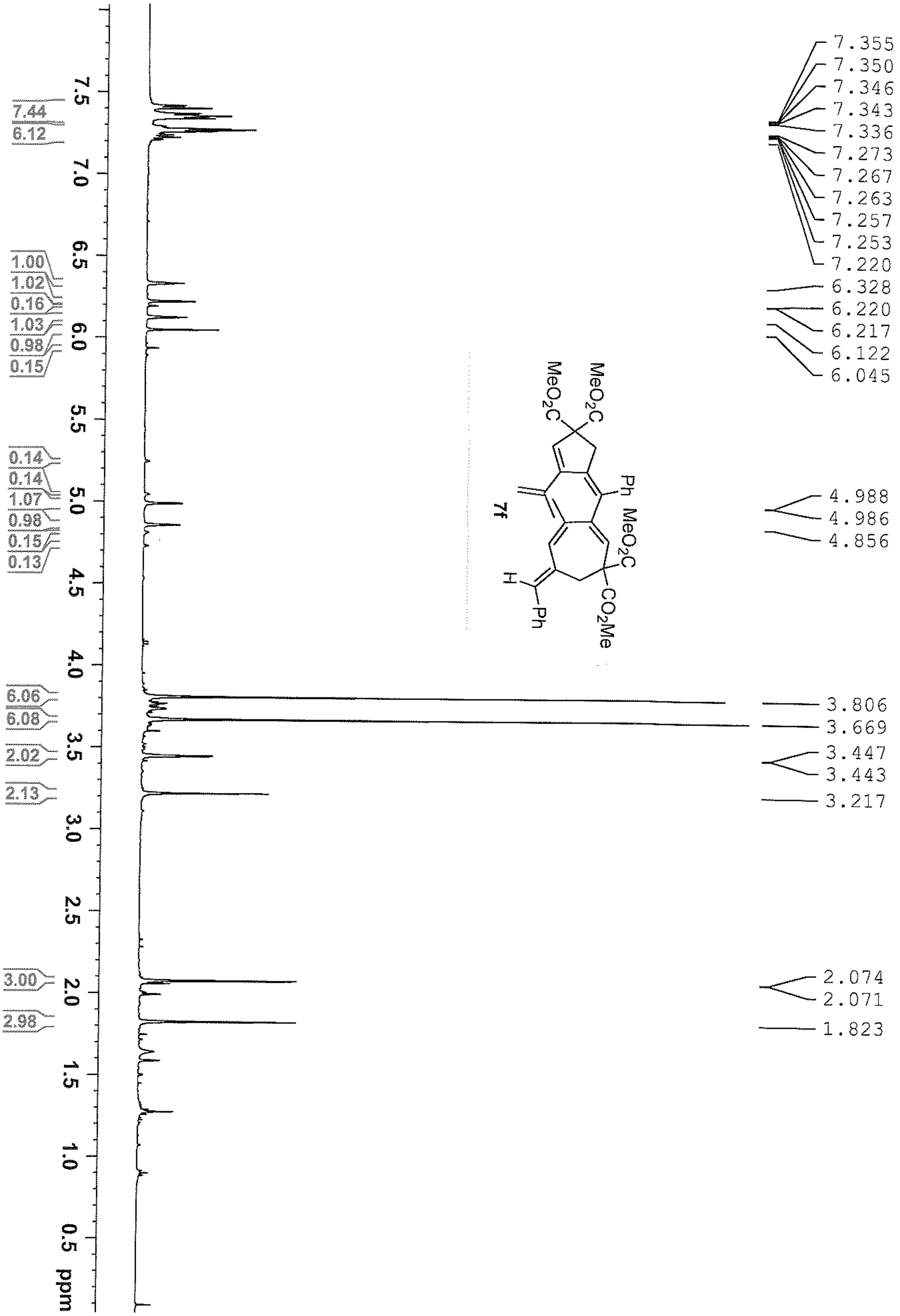
7.355
7.350
7.346
7.343
7.336
7.273
7.267
7.263
7.257
7.253
7.220
6.328
6.220
6.217
6.122
6.045
4.988
4.986
4.856
3.806
3.669
3.447
3.443
3.217
2.074
2.071
1.823
7.44
6.12
1.00
1.02
0.16
1.03
0.98
0.15
0.14
0.14
1.07
0.98
0.15
0.13
6.06
6.08
2.02
2.13
3.00
2.98
7.5
7.0
6.5
6.0
5.5
5.0
4.5
4.0
3.5
3.0
2.5
2.0
1.5
1.0
0.5 ppm
MeO2C
MeO2C
Ph
MeO2C
CO2Me
H
Ph
7f

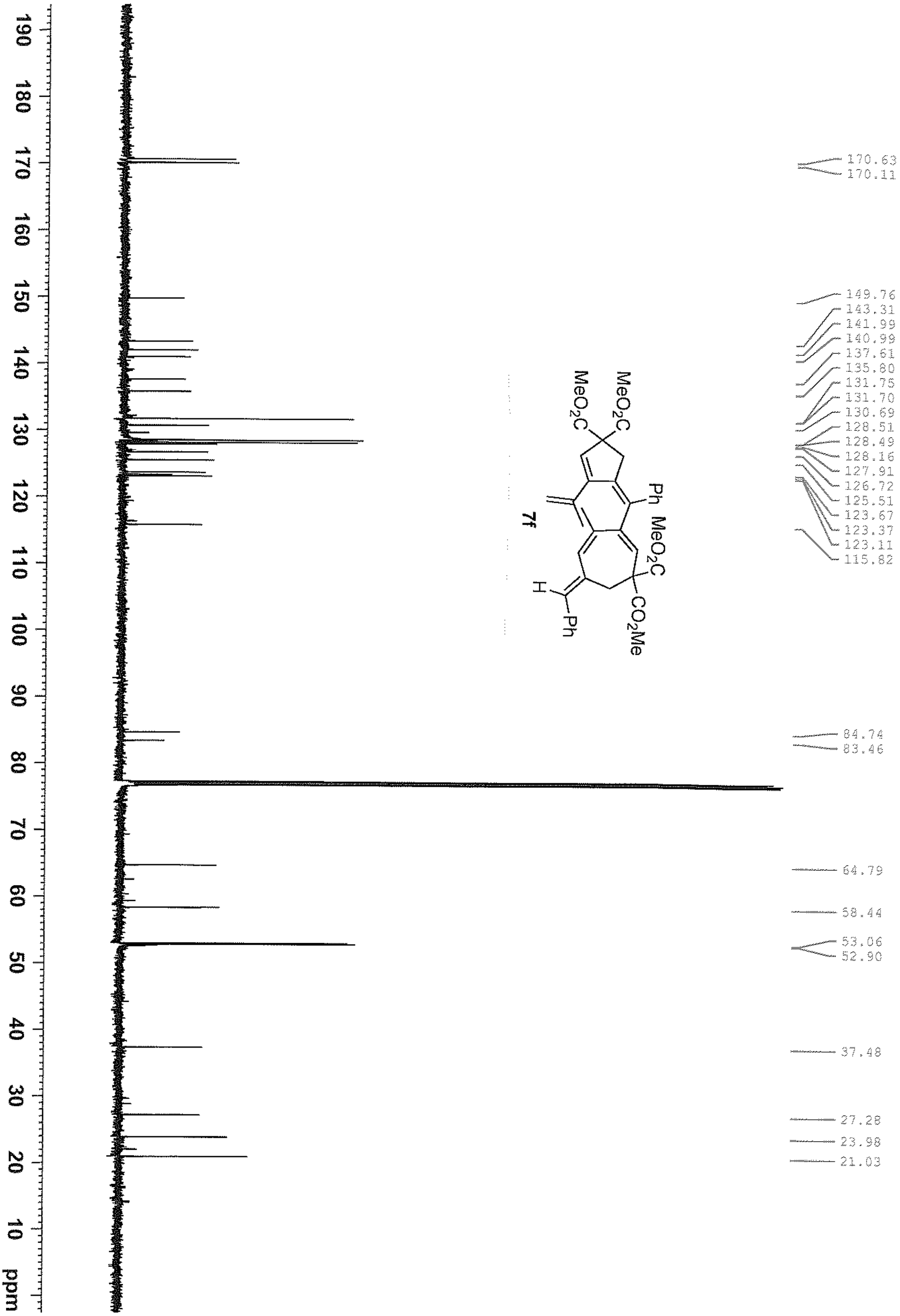
170.63
170.11
149.76
143.31
141.99
140.99
137.61
135.80
131.75
131.70
130.69
128.51
128.49
128.16
127.91
126.72
125.51
123.67
123.37
123.11
115.82
84.74
83.46
64.79
58.44
53.06
52.90
37.48
27.28
23.98
21.03
7f
ppm

# Mass Spectrum SmartFormula Report

**Analysis Info**

| | | | |
|---|---|---|---|
| Analysis Name | H:\Data2\Youqian\dyq-4-80-2000001.d | Acquisition Date | 2012-09-24 15:23:45 |
| Method | tune_low_dirk.m | Operator | pia |
| Sample Name | dyq-4-80-2 | Instrument / Ser# | micrOTOF 125 |
| Comment | | | |

**Acquisition Parameter**

| | | | | | |
|---|---|---|---|---|---|
| Source Type | ESI | Ion Polarity | Positive | Set Nebulizer | 0.4 Bar |
| Focus | Not active | | | Set Dry Heater | 170 °C |
| Scan Begin | 50 m/z | Set Capillary | 4500 V | Set Dry Gas | 4.0 l/min |
| Scan End | 1000 m/z | Set End Plate Offset | -500 V | Set Divert Valve | Source |

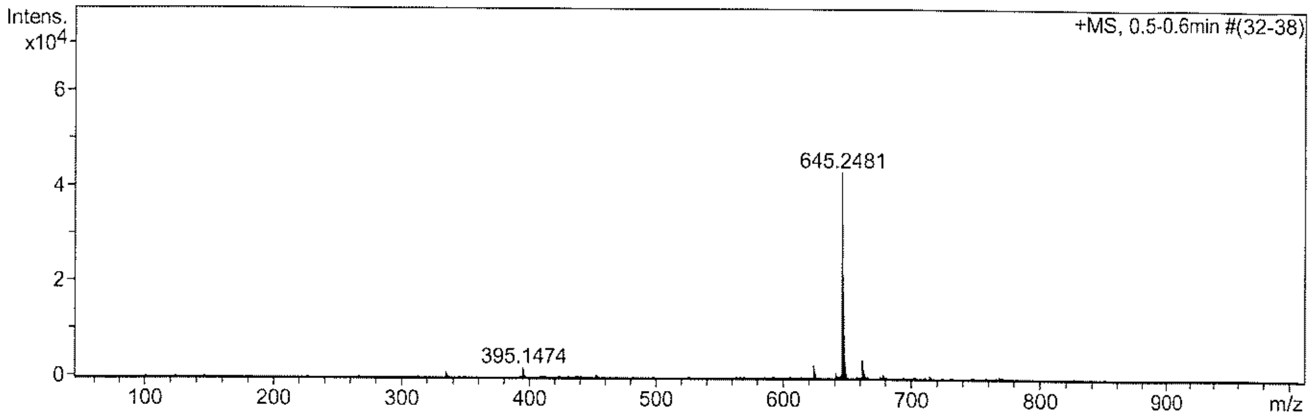


| Formula | Meas. m/z | m/z | err [ppm] | Mean err [ppm] |
|---|---|---|---|---|
| | 645.2481 | | | |
| C 38 H 38 Na O 8 | | 645.2459 | -3.4 | -3.4 |

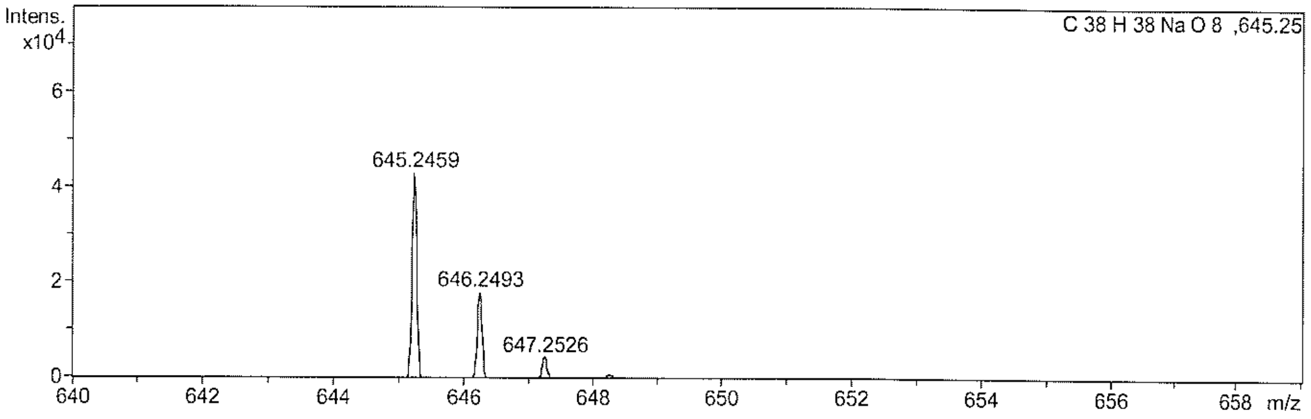


| Formula | Meas. m/z | m/z | err [ppm] | Mean err [ppm] |
|---|---|---|---|---|

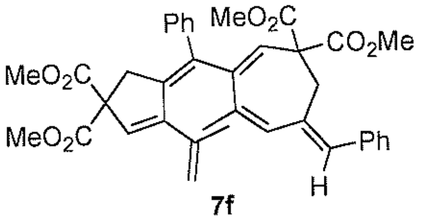


**7f**

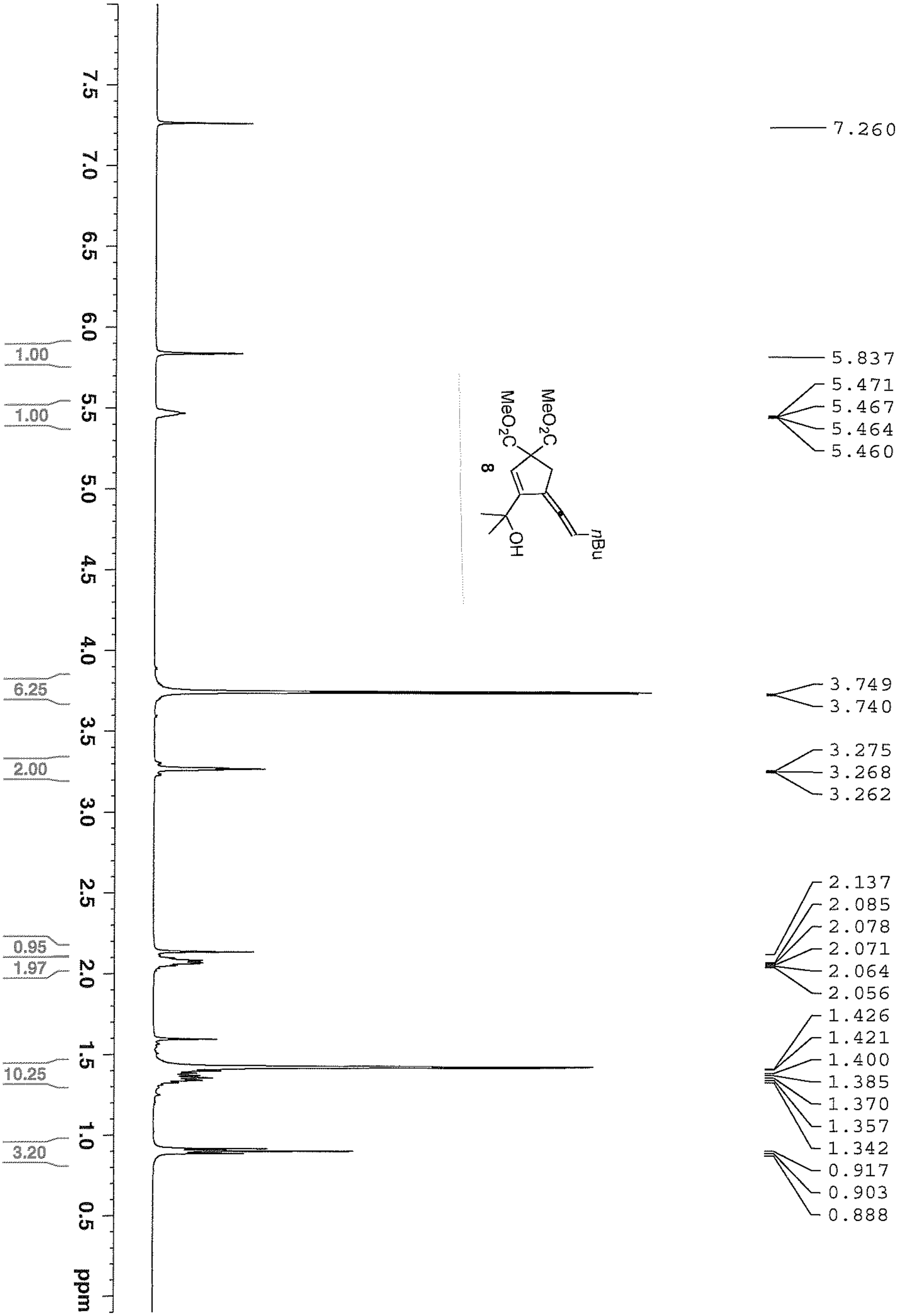
7.260
5.837
5.471
5.467
5.464
5.460
3.749
3.740
3.275
3.268
3.262
2.137
2.085
2.078
2.071
2.064
2.056
1.426
1.421
1.400
1.385
1.370
1.357
1.342
0.917
0.903
0.888
MeO2C
MeO2C
8
nBu
OH
1.00
1.00
6.25
2.00
0.95
1.97
10.25
3.20
7.5
7.0
6.5
6.0
5.5
5.0
4.5
4.0
3.5
3.0
2.5
2.0
1.5
1.0
0.5
ppm

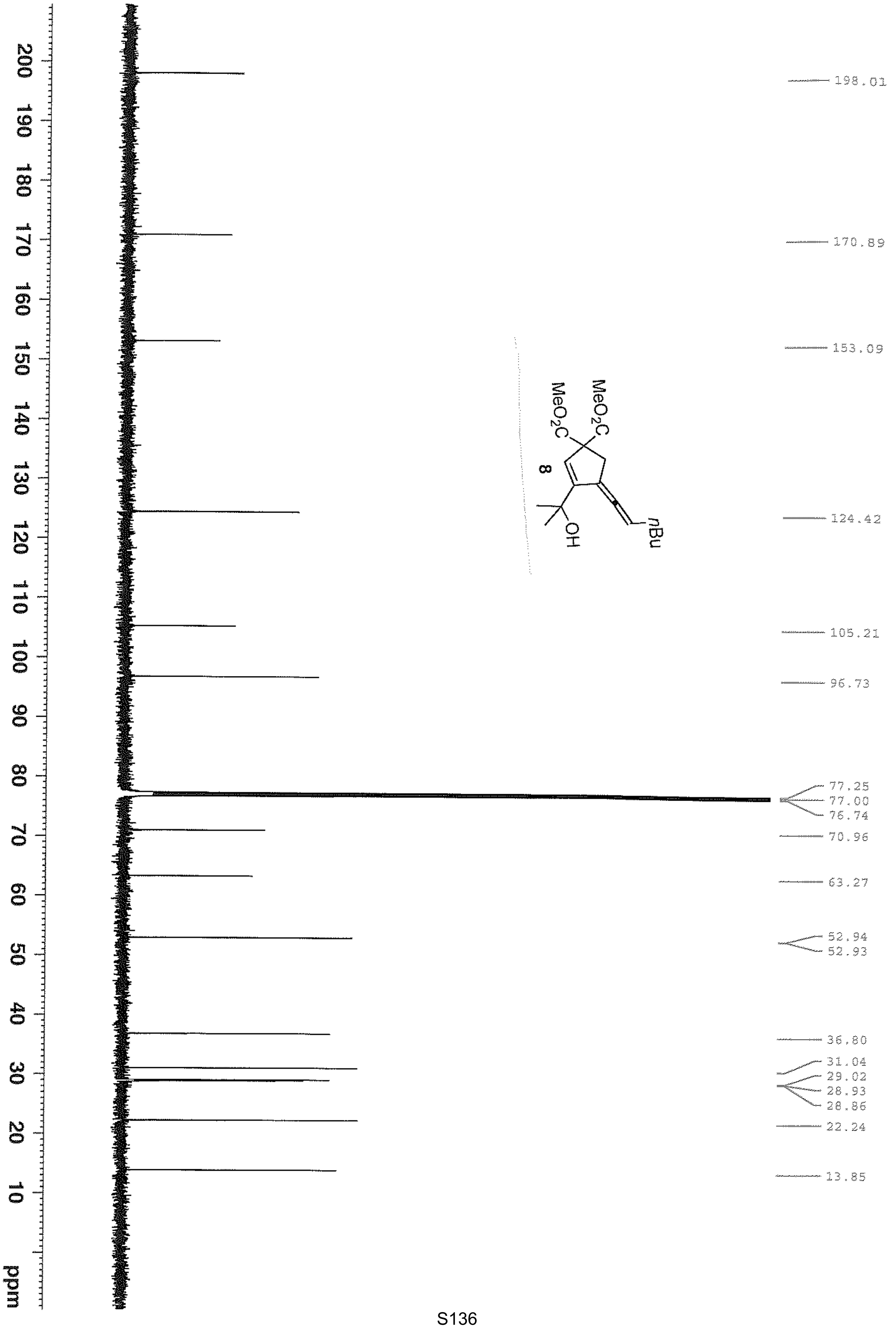

198.01
170.89
153.09
124.42
105.21
96.73
77.25
77.00
76.74
70.96
63.27
52.94
52.93
36.80
31.04
29.02
28.93
28.86
22.24
13.85
MeO2C
MeO2C
nBu
OH
8
ppm

# Mass Spectrum SmartFormula Report

**Analysis Info**

| | | | |
|---|---|---|---|
| Analysis Name | H:\Data2\Youqian\dyq-4-120000001.d | Acquisition Date | 2012-10-08 10:28:19 |
| Method | tune_wide_dirk.m | Operator | pia |
| Sample Name | dyq-4-120 | Instrument / Ser# | micrOTOF 125 |
| Comment | | | |

**Acquisition Parameter**

| | | | | | |
|---|---|---|---|---|---|
| Source Type | ESI | Ion Polarity | Positive | Set Nebulizer | 0.4 Bar |
| Focus | Not active | | | Set Dry Heater | 180 °C |
| Scan Begin | 50 m/z | Set Capillary | 4500 V | Set Dry Gas | 4.0 l/min |
| Scan End | 3000 m/z | Set End Plate Offset | -500 V | Set Divert Valve | Source |

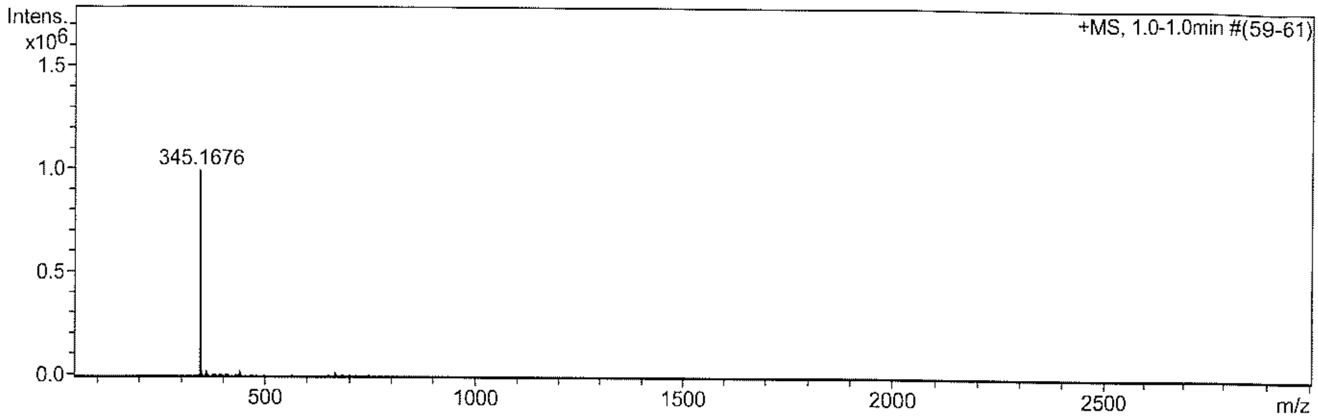


| Formula | Meas. m/z | m/z | err [ppm] | Mean err [ppm] |
|---|---|---|---|---|
| | 345.1676 | | | |
| C 18 H 26 Na O 5 | | 345.1672 | -1.1 | -0.7 |

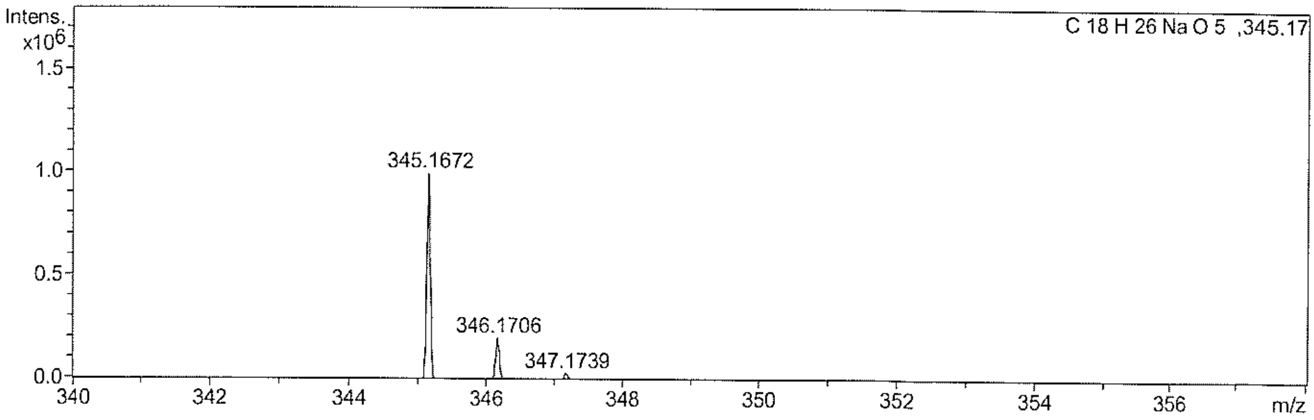


| Formula | Meas. m/z | m/z | err [ppm] | Mean err [ppm] |
|---|---|---|---|---|

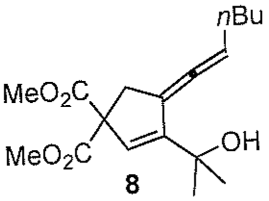

**8**

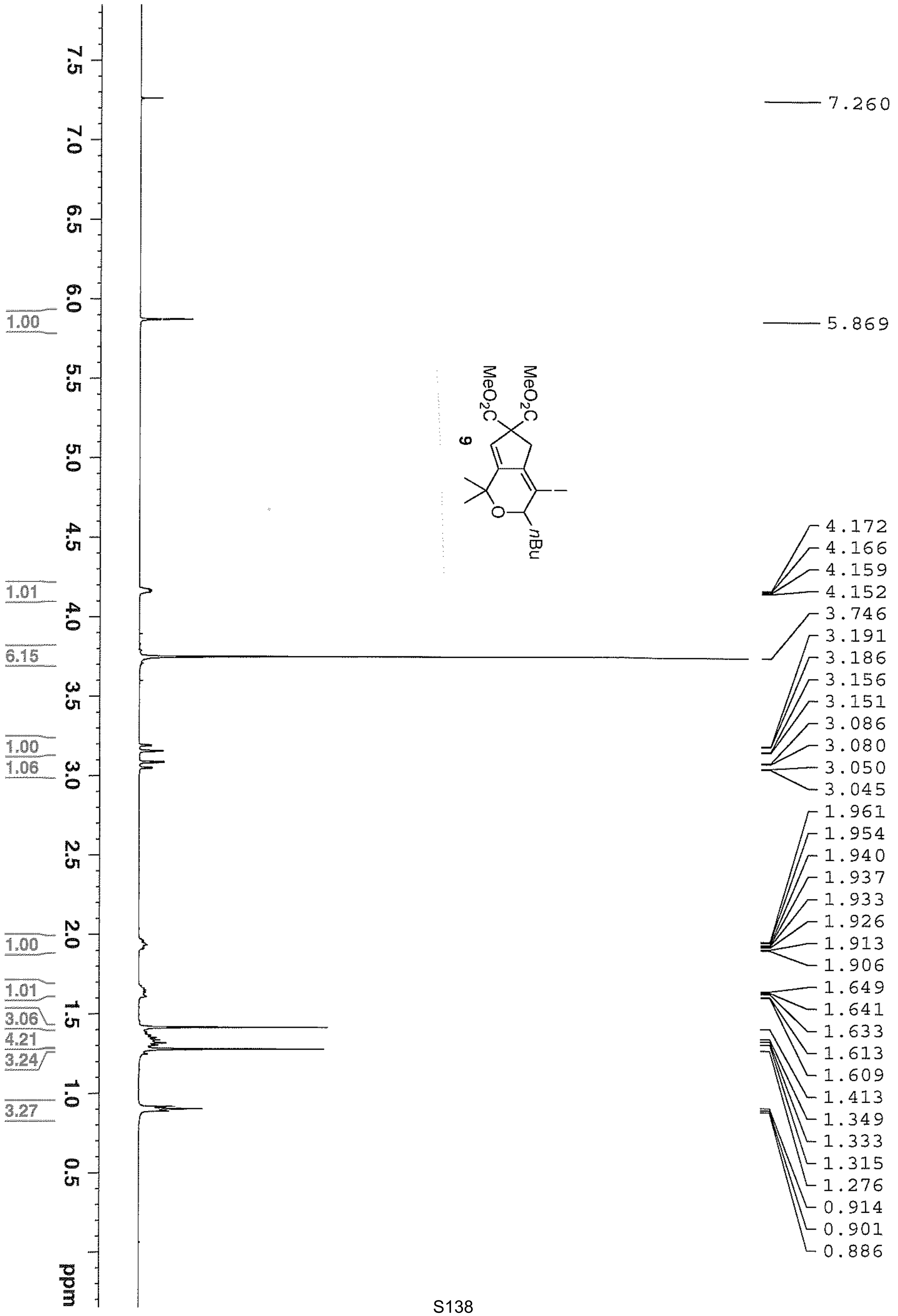
MeO2C
MeO2C
9
nBu
O
7.260
5.869
4.172
4.166
4.159
4.152
3.746
3.191
3.186
3.156
3.151
3.086
3.080
3.050
3.045
1.961
1.954
1.940
1.937
1.933
1.926
1.913
1.906
1.649
1.641
1.633
1.613
1.609
1.413
1.349
1.333
1.315
1.276
0.914
0.901
0.886
1.00
1.01
6.15
1.00
1.06
1.00
1.01
3.06
4.21
3.24
3.27
7.5
7.0
6.5
6.0
5.5
5.0
4.5
4.0
3.5
3.0
2.5
2.0
1.5
1.0
0.5
ppm

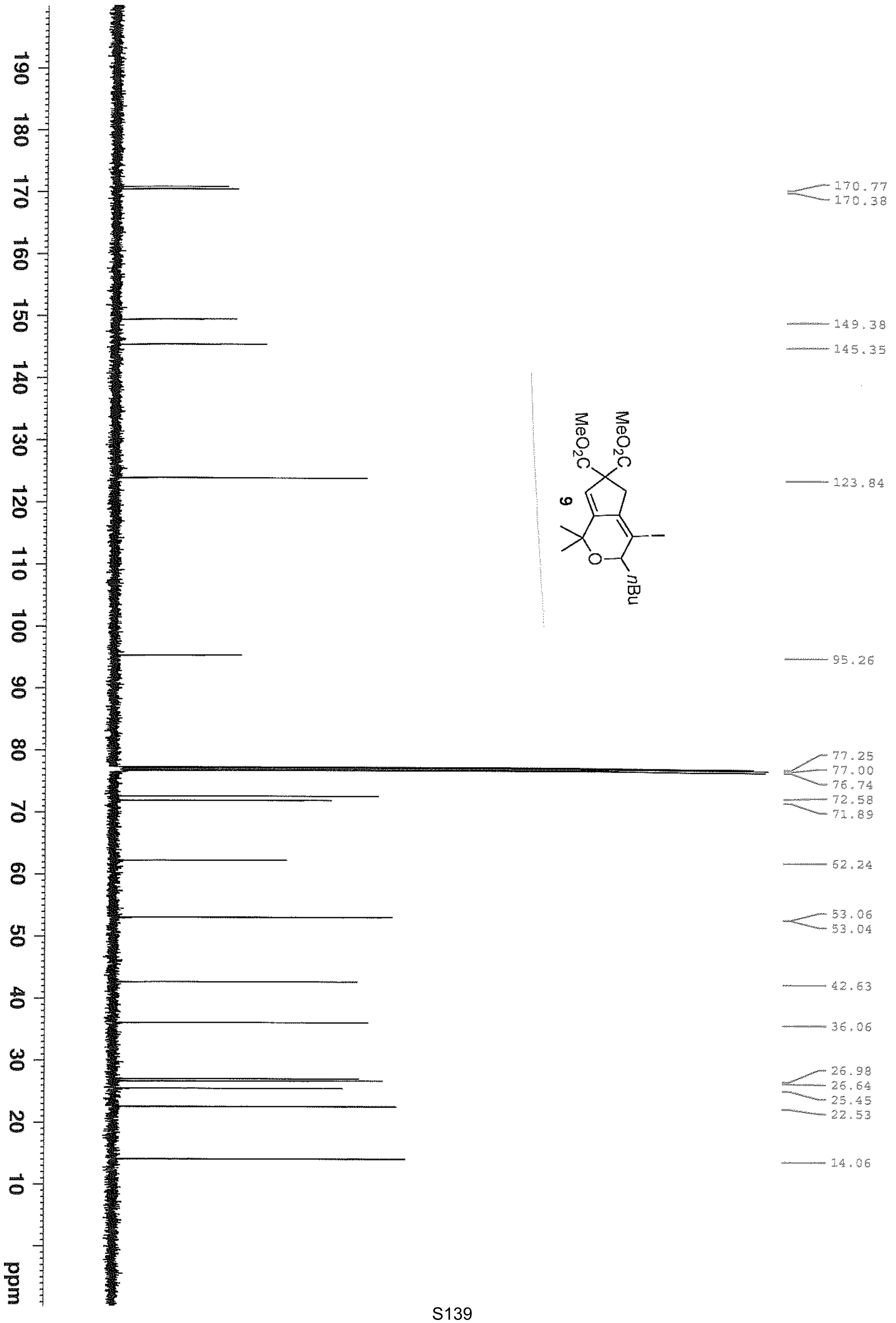
170.77
170.38
149.38
145.35
123.84
95.26
77.25
77.00
76.74
72.58
71.89
62.24
53.06
53.04
42.63
36.06
26.98
26.64
25.45
22.53
14.06
MeO2C
MeO2C
9
nBu
ppm

# Mass Spectrum SmartFormula Report

**Analysis Info**

| | | | |
|---|---|---|---|
| Analysis Name | H:\Data2\Youqian\dyq-4-123000001.d | Acquisition Date | 2012-10-08 10:58:37 |
| Method | tune_wide_dirk.m | Operator | pia |
| Sample Name | dyq-4-123 | Instrument / Ser# | micrOTOF 125 |
| Comment | | | |

**Acquisition Parameter**

| | | | | | |
|---|---|---|---|---|---|
| Source Type | ESI | Ion Polarity | Positive | Set Nebulizer | 0.4 Bar |
| Focus | Not active | | | Set Dry Heater | 180 °C |
| Scan Begin | 50 m/z | Set Capillary | 4500 V | Set Dry Gas | 4.0 l/min |
| Scan End | 3000 m/z | Set End Plate Offset | -500 V | Set Divert Valve | Source |

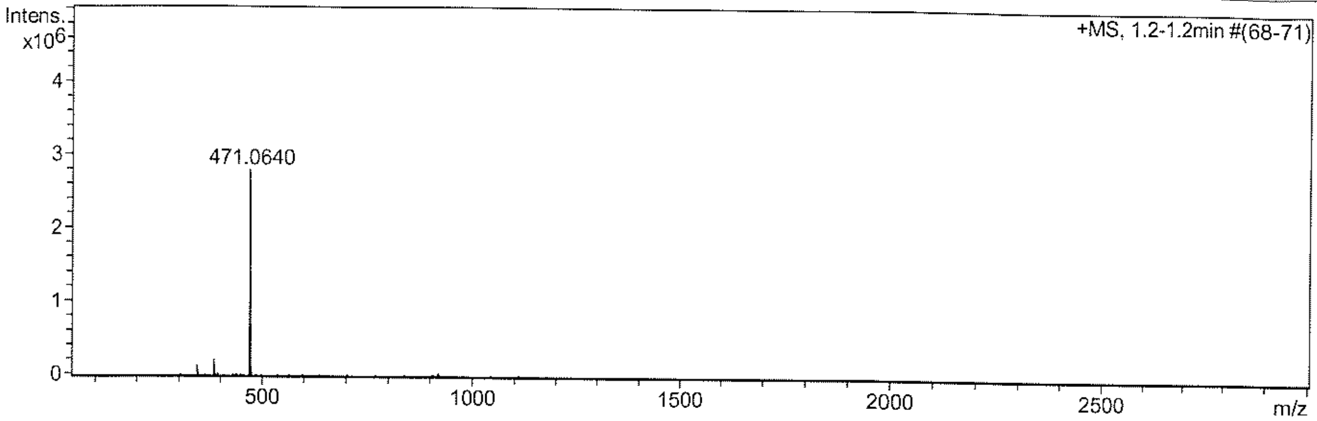


| Formula | Meas. m/z | m/z | err [ppm] | Mean err [ppm] |
|---|---|---|---|---|
| | 471.0640 | | | |
| C 18 H 25 I Na O 5 | | 471.0639 | -0.2 | 0.7 |

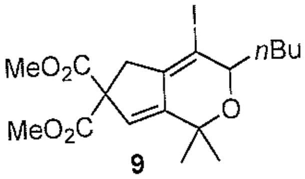


**9**

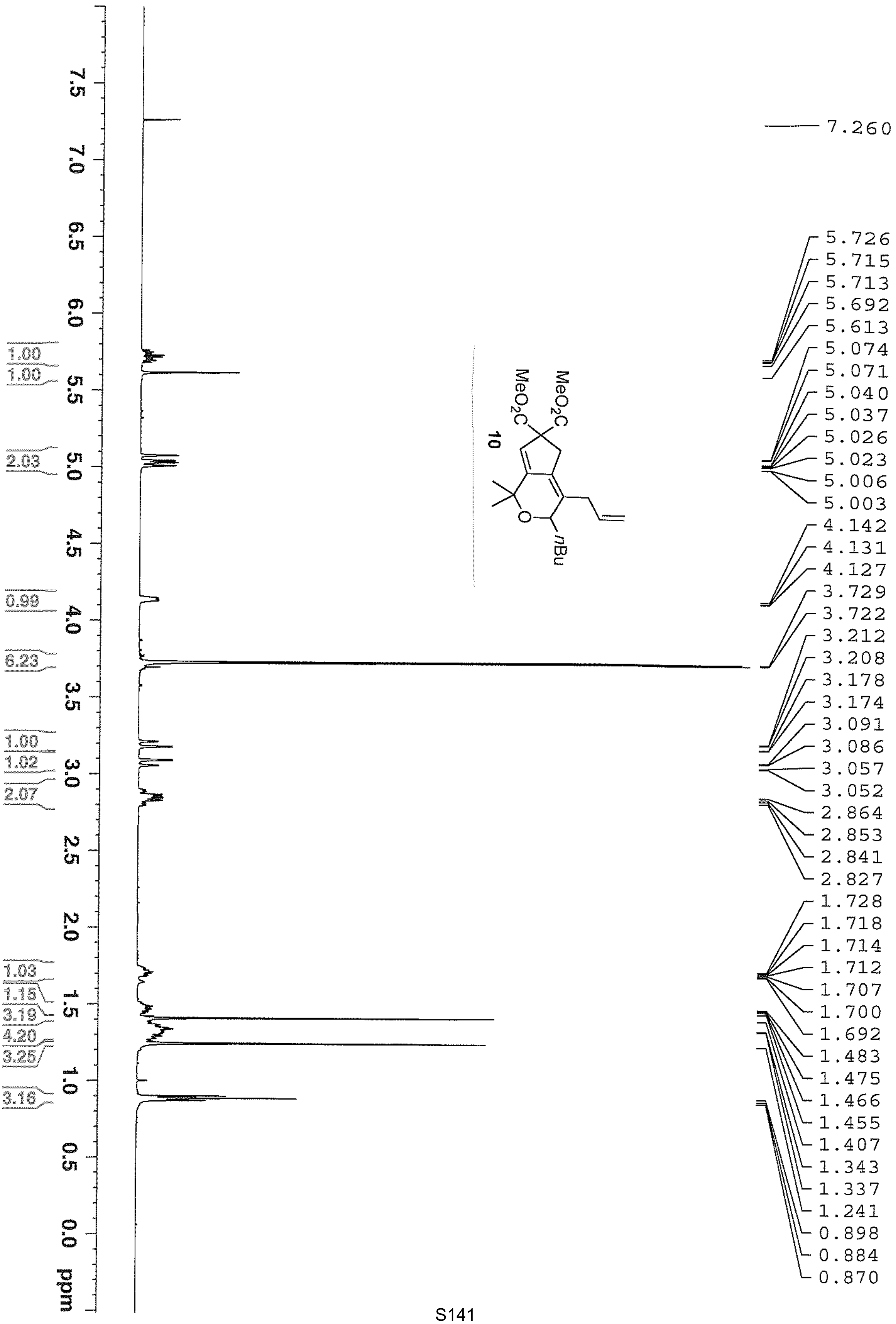
MeO2C
MeO2C
10
nBu
7.260
5.726
5.715
5.713
5.692
5.613
5.074
5.071
5.040
5.037
5.026
5.023
5.006
5.003
4.142
4.131
4.127
3.729
3.722
3.212
3.208
3.178
3.174
3.091
3.086
3.057
3.052
2.864
2.853
2.841
2.827
1.728
1.718
1.714
1.712
1.707
1.700
1.692
1.483
1.475
1.466
1.455
1.407
1.343
1.337
1.241
0.898
0.884
0.870
1.00
1.00
2.03
0.99
6.23
1.00
1.02
2.07
1.03
1.15
3.19
4.20
3.25
3.16
7.5
7.0
6.5
6.0
5.5
5.0
4.5
4.0
3.5
3.0
2.5
2.0
1.5
1.0
0.5
0.0
ppm

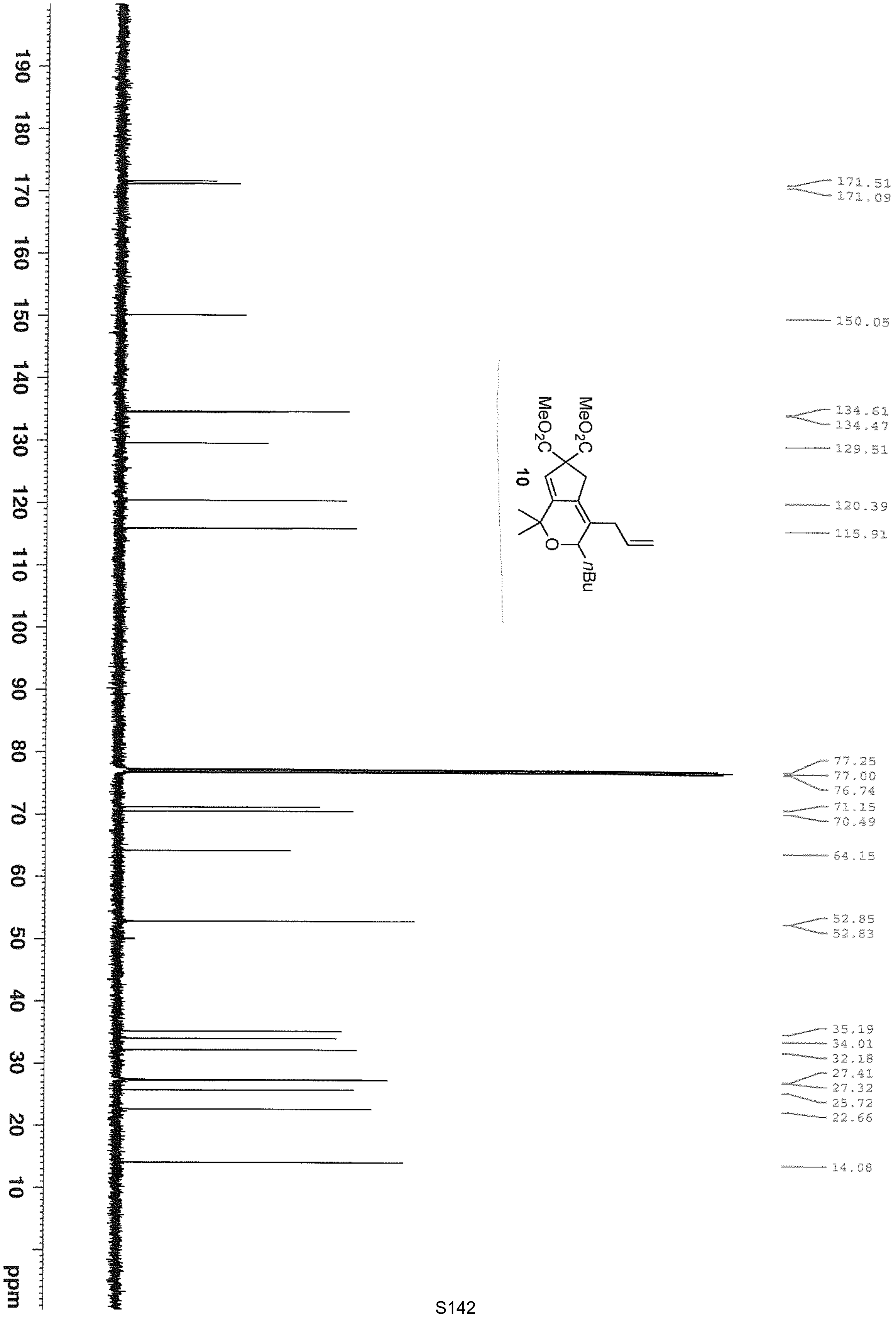
171.51
171.09
150.05
134.61
134.47
129.51
120.39
115.91
77.25
77.00
76.74
71.15
70.49
64.15
52.85
52.83
35.19
34.01
32.18
27.41
27.32
25.72
22.66
14.08
MeO2C
MeO2C
10
nBu
O
190 180 170 160 150 140 130 120 110 100 90 80 70 60 50 40 30 20 10
ppm

# Mass Spectrum SmartFormula Report

**Analysis Info**

| | | | |
|---|---|---|---|
| Analysis Name | H:\Data2\Youqian\dyq-4-124000002.d | Acquisition Date | 2012-10-08 11:47:21 |
| Method | tune_wide_dirk.m | Operator | pia |
| Sample Name | dyq-4-124 | Instrument / Ser# | micrOTOF 125 |
| Comment | | | |

**Acquisition Parameter**

| | | | | | |
|---|---|---|---|---|---|
| Source Type | ESI | Ion Polarity | Positive | Set Nebulizer | 0.4 Bar |
| Focus | Not active | | | Set Dry Heater | 180 °C |
| Scan Begin | 50 m/z | Set Capillary | 4500 V | Set Dry Gas | 4.0 l/min |
| Scan End | 3000 m/z | Set End Plate Offset | -500 V | Set Divert Valve | Source |

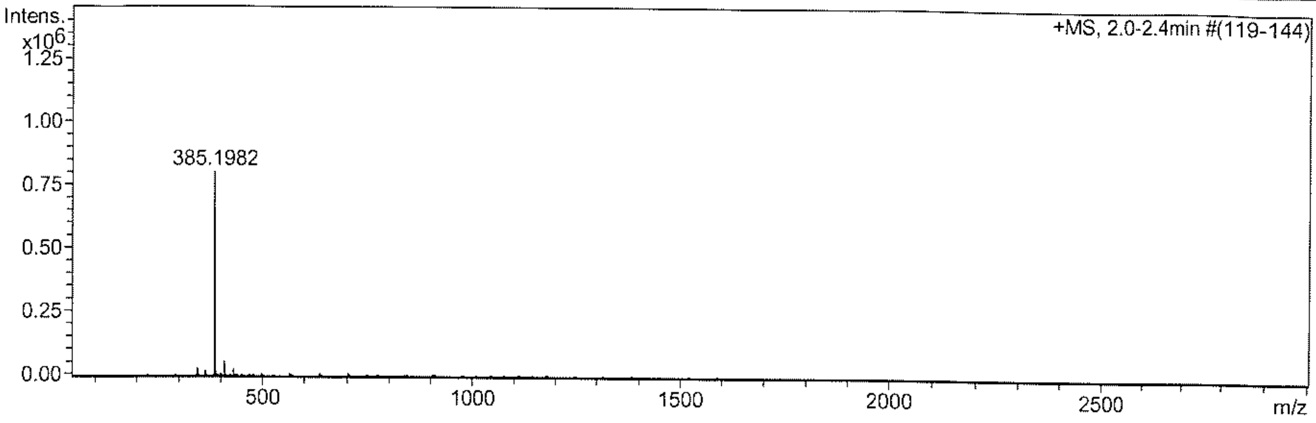


| Formula | Meas. m/z | m/z | err [ppm] | Mean err [ppm] |
|---|---|---|---|---|
| | 385.1982 | | | |
| C 21 H 30 Na O 5 | | 385.1985 | 0.8 | 1.1 |

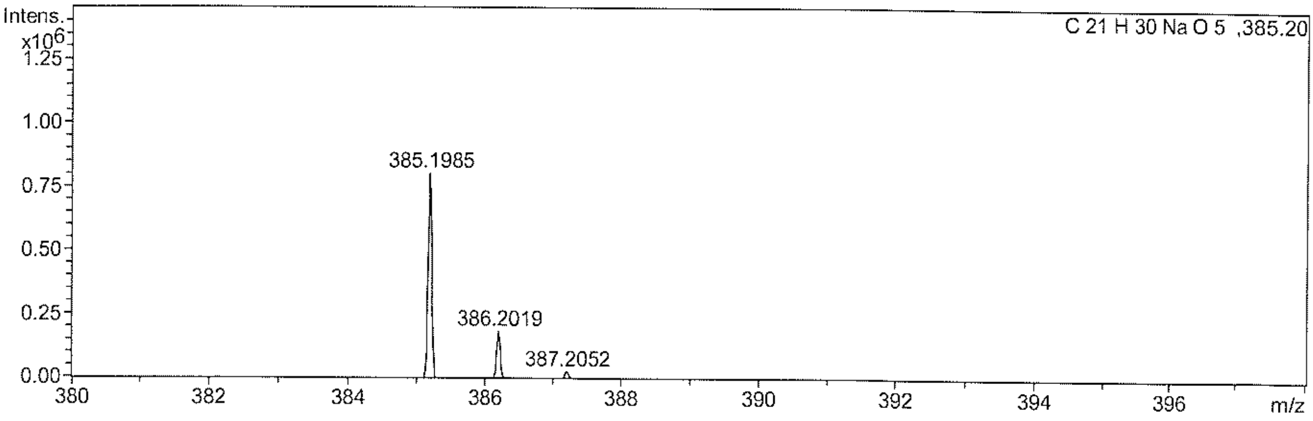


| Formula | Meas. m/z | m/z | err [ppm] | Mean err [ppm] |
|---|---|---|---|---|

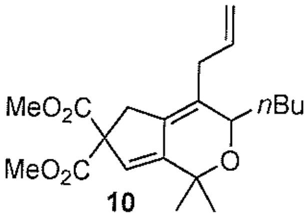


**10**

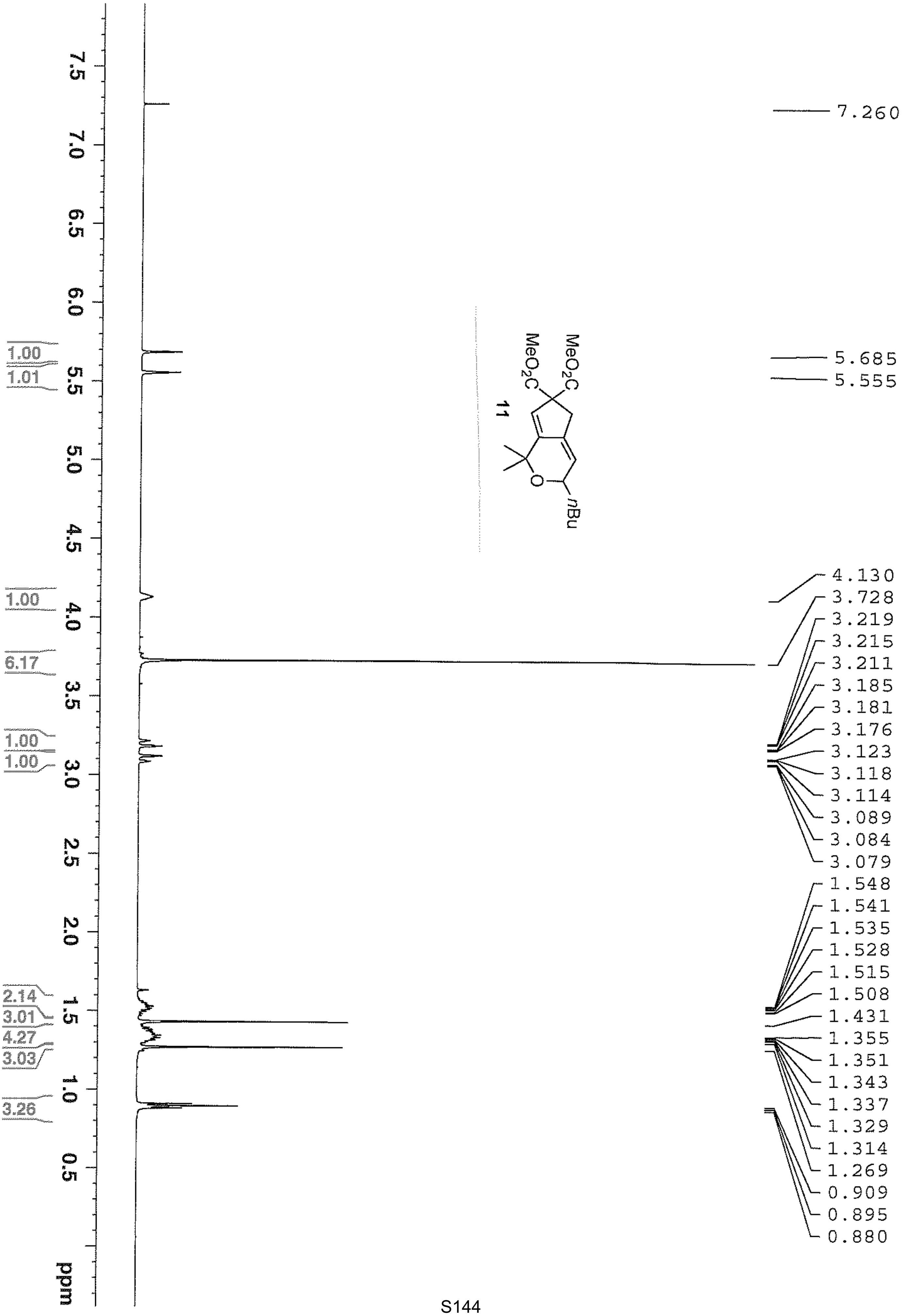
MeO2C
MeO2C
11
O
nBu
7.260
5.685
5.555
4.130
3.728
3.219
3.215
3.211
3.185
3.181
3.176
3.123
3.118
3.114
3.089
3.084
3.079
1.548
1.541
1.535
1.528
1.515
1.508
1.431
1.355
1.351
1.343
1.337
1.329
1.314
1.269
0.909
0.895
0.880
1.00
1.01
1.00
6.17
1.00
1.00
2.14
3.01
4.27
3.03
3.26
7.5
7.0
6.5
6.0
5.5
5.0
4.5
4.0
3.5
3.0
2.5
2.0
1.5
1.0
0.5
ppm

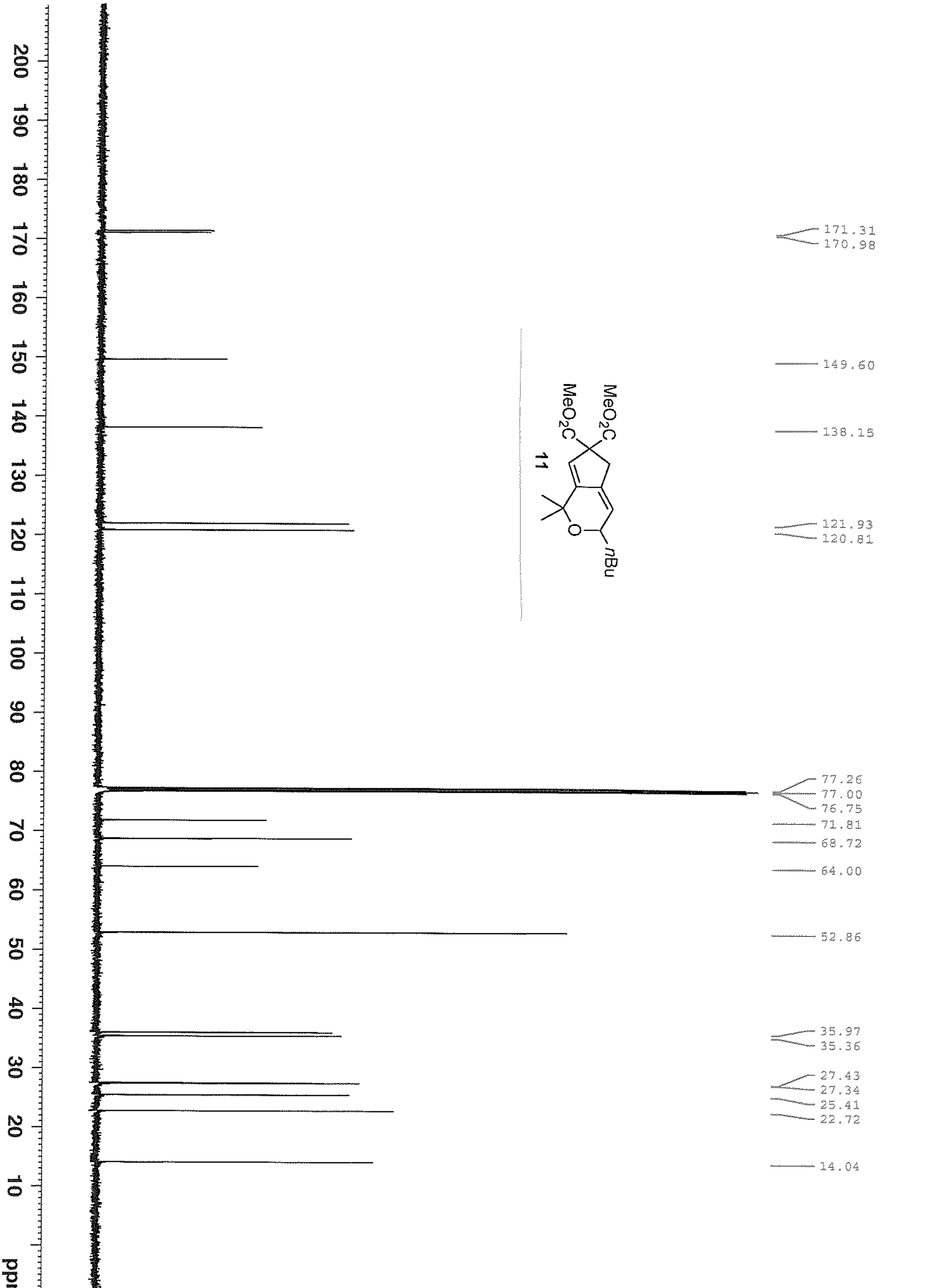
200
190
180
170
160
150
140
130
120
110
100
90
80
70
60
50
40
30
20
10
ppm
171.31
170.98
149.60
138.15
121.93
120.81
77.26
77.00
76.75
71.81
68.72
64.00
52.86
35.97
35.36
27.43
27.34
25.41
22.72
14.04
MeO2C
MeO2C
nBu
O
11

# Mass Spectrum SmartFormula Report

**Analysis Info**

| | |
|---|---|
| Analysis Name | H:\Data2\Youqian\dyq-4-128000003.d |
| Method | tune_wide_dirk.m |
| Sample Name | dyq-4-128 |
| Comment | |

| | |
|---|---|
| Acquisition Date | 2012-10-08 10:49:10 |
| Operator | pia |
| Instrument / Ser# | micrOTOF 125 |

**Acquisition Parameter**

| | | | | | |
|---|---|---|---|---|---|
| Source Type | ESI | Ion Polarity | Positive | Set Nebulizer | 0.4 Bar |
| Focus | Not active | | | Set Dry Heater | 180 °C |
| Scan Begin | 50 m/z | Set Capillary | 4500 V | Set Dry Gas | 4.0 l/min |
| Scan End | 3000 m/z | Set End Plate Offset | -500 V | Set Divert Valve | Source |

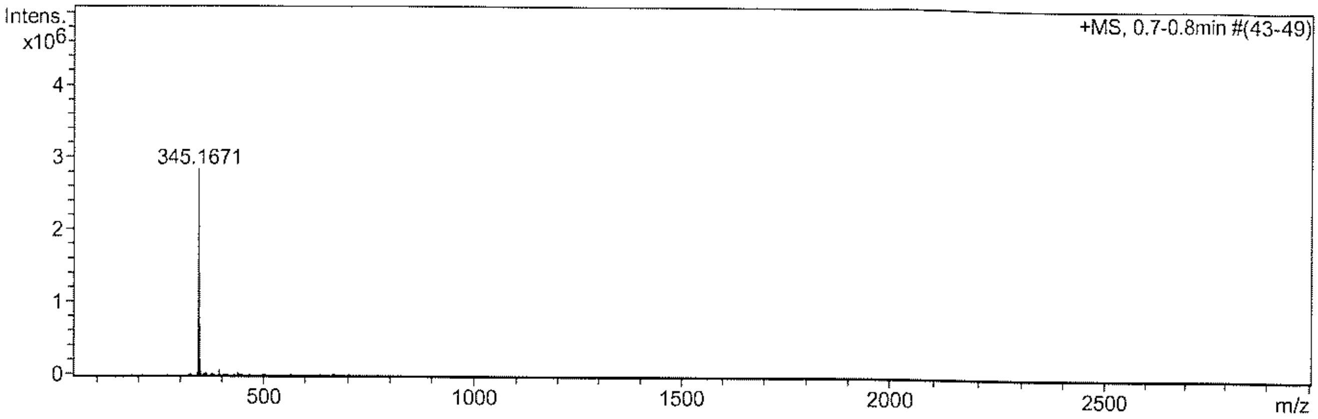


| Formula | Meas. m/z | m/z | err [ppm] | Mean err [ppm] |
|---|---|---|---|---|
| | 345.1671 | | | |
| C 18 H 26 Na O 5 | | 345.1672 | 0.5 | 2.5 |

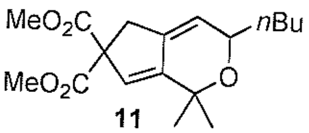


**11**
